# Spatial Control Over Tether‐Tunable Coordination on Palladium for Divergent Synthesis of Bicyclo[n.1.0]alkanes via Oxidative Cyclopropanation of Enynes

**DOI:** 10.1002/advs.202522444

**Published:** 2026-01-30

**Authors:** Ting Yuan, Lei Shi

**Affiliations:** ^1^ School of Science (Shenzhen) School of Chemistry and Chemical Engineering Harbin Institute of Technology Harbin China

**Keywords:** cyclic diacyl peroxides, oxidative cyclopropanation, Pd(II)/Pd(IV)catalysis

## Abstract

Bicyclo[n.1.0]alkanes are highly valuable scaffolds in drug discovery and synthetic chemistry. However, their efficient and stereocontrolled construction remains a desirable yet challenging task. Herein, we report a palladium‐catalyzed oxidative cyclopropanation of 1,n‐enynes using cyclic malonyl peroxides as modular, tether‐tunable oxidants. This ligand‐free method enables divergent synthesis of three classes of bicyclo[n.1.0]alkanes with broad substrate scope, encompassing both oxygen‐ and nitrogen‐linked systems as well as sterically hindered trisubstituted alkenes. The transformation proceeds under mild conditions with high efficiency and stereospecificity, and an asymmetric variant achieves moderate yet promising enantiocontrol. Green metrics and biocompatibility assessments suggest that merging transition metal catalysis with cyclic malonyl peroxides may open avenues for sustainable and bio‐relevant synthetic strategies.

## Graphical abstract

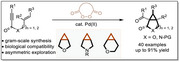

## 1 Introduction

**SCHEME 1:**
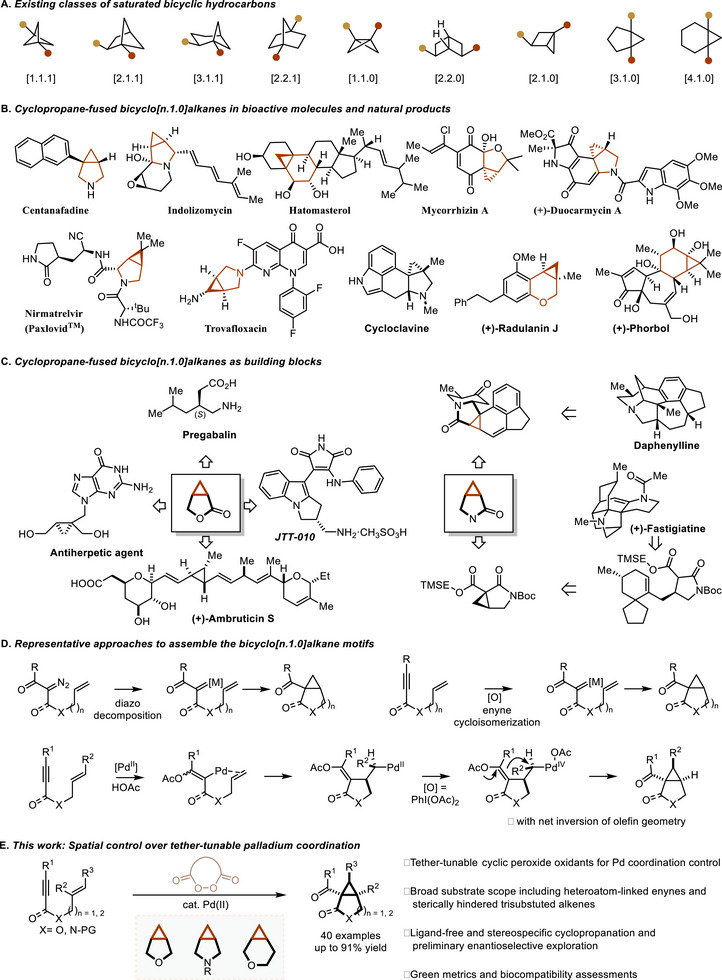
Background and synopsis of the current work.

In recent years, substantial efforts have been devoted to the design and development of saturated bicyclic hydrocarbons (Scheme 1A). These architectures present a range of attractive features, such as serving as non‐aromatic bioisosteres, enabling conformational restraint of flexible structures, and increasing both the fraction of sp^3^‐hybridized carbons (F_sp_3) and the number of stereogenic centers (F_Cstereo_) [1, 2, 3]. Among these scaffolds, cyclopropane‐fused bicyclo[n.1.0]alkanes have emerged as privileged structural motifs, frequently encountered in natural products and widely utilized in medicinal chemistry for the discovery and optimization of synthetic drug molecules (Scheme 1B) [4, 5, 6, 7, 8, 9, 10, 11, 12, 13, 14, 15, 16, 17, 18, 19]. Furthermore, they have also served as versatile building blocks to undergo diverse transformations for further chemical elaboration (Scheme 1C). Importantly, the incorporation of cyclopropane moiety imparts unique physicochemical characteristics, such as bond angle strain, coplanarity of ring carbons, shortened C─C bonds with enhanced π‐character, and stronger C─H bonds [20, 21, 22]. Taken together, these properties contribute to enhanced metabolic stability, improved binding affinity, and superior pharmacokinetic profiles, rendering such frameworks highly valuable in contemporary drug discovery campaigns aimed at achieving desired biological functions.

Over the past several years, several strategies have been developed to assemble the bicyclo[n.1.0]alkane core with the installation of distinct functionalities (Scheme 1D). For example, the transition metal‐catalyzed decomposition of diazo compounds followed by intermolecular [23, 24] or intramolecular [12, 25, 26, 27, 28, 29, 30] alkene insertion represents a well‐established and reliable method. In parallel, enyne cycloisomerization via metal‐catalyzed alkyne activation [31, 32, 33, 34, 35, 36, 37, 38, 39, 40, 41, 42, 43, 44, 45, 46, 47, 48], which leverages the reactive capacity of metal carbenoids species (e. g., Au [34, 35, 36, 37], Pt [38, 39], Ir [39, 40], Ru [41, 42], Rh [43, 44, 45, 46], Cu [47], or Ag [48] complexes), has emerged as another powerful alternative while avoiding the use of potentially hazardous diazo precursors. Other notable approaches include *α*‐cyclopropanation of aldehydes [49], annulation or 1,3‐dipolar cycloaddition of cyclopropenes [50, 51], Pd‐catalyzed C(sp^3^)–H functionalization of cyclopropanes [52, 53, 54], Lewis‐acid‐catalyzed [55, 56] or Pd‐catalyzed [57, 58] bicyclization of enynols via allenyl intermediates [59, 60], reductive amination of dicarbonyls [61], organoelectrocatalytic cyclopropanation of active methylene componds [62], and Cu‐catalyzed intramolecular reductive isocyanide‐alkene cycloaddition [63]. These seminal contributions have laid a solid foundation for the rapid construction of bicyclo[n.1.0]alkane moieties by expanding the substrate scope and understanding individual mechanistic insights.

In addition to the aforementioned strategies, palladium‐catalyzed oxidative cyclization of 1,n‐enynes constitutes a mechanistically distinct and synthetically valuable route. Early contributions by both Tse [64] and Sanford [65] in 2007 established that 1,n‐enynes underwent efficient cyclization under a Pd^II^/Pd^IV^ catalytic manifold with PhI(OAc)_2_ as an oxidant, delivering bicyclo[n.1.0]alkane‐based products with high regio‐ and diastereocontrol. A landmark advance in enantioselective catalysis was achieved by Sasai and co‐workers in 2009 through the deployment of a tailored chiral spiro bis(isoxazoline) (SPRIX) ligand in the Pd^II^/Pd^IV^ catalysis [66], enabling the construction of such bicyclic lactones with good yields and enantioselectivities, albeit to a very limited extent. The success of these reactions was critically dependent on the substitution patterns on the alkene unit of a given substrate and on the structural modulation of the palladium coordination sphere through the selection of appropriate oxidant and ligand. Notably, unlike related metal‐catalyzed cyclopropanation processes of enynes via metallocarbene or allene intermediates [31, 32, 33, 34, 35, 36, 37, 38, 39, 40, 41, 42, 43, 44, 45, 46, 47, 48, 55 ^–^ 60], these transformations proceed with net inversion of configuration at the carbon atom bound to Pd(IV), relative to the starting alkene geometry [67, 68, 69, 70]. This stereochemical outcome is determined by a key cyclopropane‐forming step, which involves an intramolecular nucleophilic attack of a tethered olefin onto the C–Pd(IV) intermediate. This general mechanistic paradigm would allow us to access skeletally diverse [bicyclo[n.1.0]alkanes with varied substitution patterns and tether units. Nevertheless, existing methods typically require a large excess of PhI(OAc)_2_ and/or high reaction temperatures and are limited in substrate generality and ligand‐controlled enantioselective processes [64, 65, 66, 67, 68, 69, 70].

Recently, as part of our ongoing interest in peroxide‐mediated transformations [71, 72, 73, 74, 75, 76], we discovered that employing cyclic diacyl peroxides as bystanding oxidants unlocks a powerful platform for selective reductive elimination (RE) from high‐valent Pd(IV) intermediates, realizing C─H functionalization reactions in a substrate‐ or oxidant‐independent fashion [77, 78]. Conceptually, this strategy is significant because the modular nature of the cyclic diacyl peroxide oxidant allows for precise tuning of the palladium coordination sphere. This control, manifested as a confined intramolecularity through spatial bisanion coordination [79, 80], not only suppresses competing RE pathways but also presents a unique opportunity to manipulate other fundamental steps in Pd^II^/Pd^IV^ catalysis. Consequently, we wondered whether such spatial control over tether‐tunable palladium coordination could be harnessed as an alternative yet distinct approach to prepare bicyclo[n.1.0]alkane scaffolds. Herein, we describe the discovery and development of a palladium‐catalyzed oxidative cyclization of enynes that employs cyclic malonyl peroxides as bystanding two‐electron oxidants under ligand‐free conditions, providing direct access to three classes of bicyclo[n.1.0]alkane scaffolds with broad substrate generality and functional group tolerance (Scheme 1E). The synthetic utility of this protocol is further highlighted by its favorable green metrics, demonstrated biocompatibility with biomolecules such as pUC 19 and DNase I, and the preliminary success of an asymmetric variant, which provides products in good yields with moderate enantiocontrol (up to 78%).

## 2 Results and Discussion

### 2.1 Optimization of the Reaction Conditions

We commenced our investigation of the Pd^II^‐catalyzed oxidative cyclization using 2‐methylallyl phenylpropiolate (**1a**) as the model substrate (Table 1, for details∖, see Supporting information). Under an Ar atmosphere, treatment of **1a** with 10 mol% of Pd(OAc)_2_ in AcOH/MeCN (0.1 m, v/v = 1:1) at 50°C in the presence of 2.0 equiv of MPO‐1 afforded 1‐benzoyl‐5‐methyl‐3‐oxabicyclo[3.1.0]hexan‐2‐one (**1**) in 73% yield (entry 1). A survey of structurally varied cyclic diacyl peroxides (MPO‐2 to MPO‐8 and PPO) established the generality of this scaffold, with bicyclic product 1 isolated in 43%–68% yield (entry 2). Conversely, the use of a peracid (DPPA, 2,2' ‐diperoxydiphenic acid) [72, 75] or common open‐chain peroxides, including BPO, LPO, DTPB, TBPB, TBHP, and H_2_O_2_, was ineffective, resulting in no or only trace formation of **1** (entry 3). This stark contrast underscores the criticality of the constrained dicarboxylate framework—derived from the cyclic malonyl peroxide scaffold—for achieving productive catalysis. We posit that the confined intramolecularity imparted by spatial bisanion coordination favors the formation of unique Pd coordination geometries that are pivotal for an efficient Pd^II^/Pd^IV^ catalytic cycle [77, 78, 79, 80]. Optimization of the solvent system revealed that increasing the AcOH/MeCN ratio to 4:1 improved the yield to 84% (entry 4). Alternative palladium(II) catalysts such as Pd(TFA)_2_, Pd(MeCN)_2_Cl_2_ and PdCl_2_ proved less effective than Pd(OAc)_2_, affording slightly lower yields (entry 5). The reaction efficiency could be further improved by shortening the reaction time, while further reducing the oxidant loading to 1.8 equiv of MPO‐1 increased the yield to 91% (entries 6 and 7). However, decreasing the catalyst loading to 5 mol % Pd(OAc)_2_, even with a prolonged reaction time of 12 h, led to a significantly diminished yield of 61% (entry 8), indicating that a 10 mol% catalyst loading is necessary to achieve maximum conversion and yield. To verify the preparative utility of this method, a gram‐scale reaction proceeded with comparable efficiency to deliver the desired product **1** in 86% yield. As anticipated, control experiments confirmed that both the Pd source and the cyclic peroxide are essential for the reaction to occur (entry 9). The lack of reactivity in the absence of cyclic peroxide reinforces its dual function as a sacrificial two‐electron oxidant and a coordinating bidentate ligand. To further demonstrate the practical robustness of this optimized protocol, a condition‐based sensitivity assessment was conducted (Figure S1) [81, 82]. The transformation was shown to be remarkably tolerable toward perturbations in the concentration (*c*), oxygen level, light intensity (*I*), and scale. A more discernible yield reduction was observed with the introduction of water, while temperature variations showed a moderate effect on the reaction yield, thus identifying the key parameters for ensuring reproducible performance under standard laboratory conditions.

**TABLE 1:** Optimization of the reaction conditions.^a^

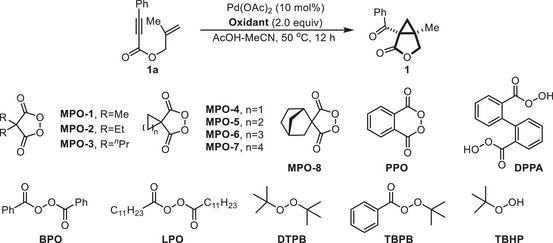		
Entry	Deviation from standard conditions	Yield of 1 (%)^b)^
1	None	73
2	MPO‐2 ∼ MPO‐8/PPO instead of MPO‐1	43 ∼ 68
3	DPPA/BPO/LPO/DTPB/TBPB/TBHP/H_2_O_2_ instead of MPO‐1	trace/N.D.
4	AcOH/MeCN (0.1 m, v/v = 4:1)	84
5	Pd(OCOCF_3_)_2_/Pd(MeCN)_2_Cl_2_/PdCl_2_ instead of Pd(OAc)_2_	79^c)^ /68^c)^ / 63^c)^
6	Reaction for 5 h	90^c)^
7	Reaction for 5 h with MPO‐1(1.8 equiv)	91^c)^, (86)^c^, ^d^
8	With Pd(OAc)_2_ (5 mol%)	61^c^, ^e^
9	No Pd(OAc)_2_/MPO‐1	0^c)^

^a^ Reaction conditions: **1a** (0.1 mmol, 1.0 equiv.), Pd(OAc)_2_ (0.01 mmol, 10 mol%), MPO‐1 (0.2 mmol, 2.0 equiv), AcOH/MeCN (0.1 m, v/v = 1:1), 50°C, under Ar atmosphere,

^b^ Isolated yield,

^c^ AcOH/MeCN (0.1 m, v/v = 4:1),

^d^ Reaction performed at 5 mmol scale,

^e^ 12 h. BPO = Benzoyl peroxide. LPO = Dilauroyl peroxide. DTPB = Di‐*tert*‐butyl peroxide. TBPB = *tert*‐Butyl peroxybenzoate. TBHP = tert‐Butyl hydroperoxide. N.D. = No desired product was detected.

### 2.2 Substrate Scope of This Oxidative Cyclization

**SCHEME 2:**
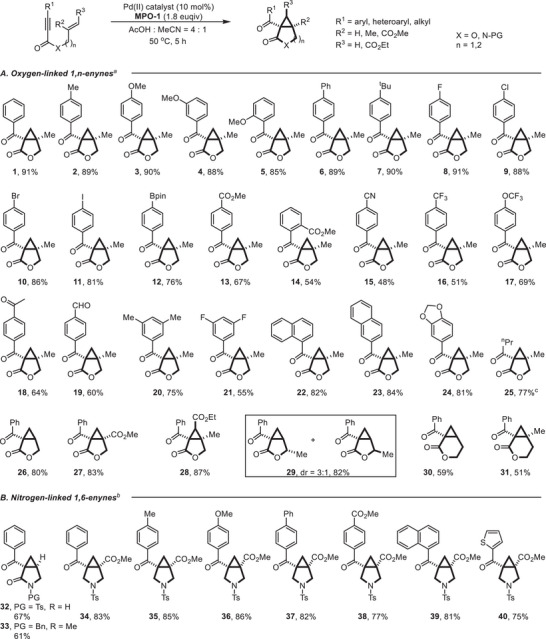
Substrate Scope of Pd^II^‐catalyzed oxidative cyclization of 1,n‐enynes. (a)Reaction conditions: substrate (0.10 mmol, 1.0 equiv), Pd(OAc)_2_ (0.01 mmol, 10 mol%), **MPO‐1** (0.18 mmol, 1.8 equiv), AcOH/MeCN (0.1 m, v/v=4:1), 50°C, 5 h, under Ar atmosphere; Yield of isolated product. (b)Reaction conditions: substrate (0.10 mmol, 1.0 equiv), Pd(TFA)_2_ (0.01 mmol, 10 mol%), **MPO‐1** (0.20 mmol, 2.0 equiv), AcOH/MeCN (0.1 m, v/v=4:1), 50°C, 12 h, under Ar atmosphere; Yield of isolated product. (c)Reaction was performed for 12 h.

With the optimized reaction conditions, we proceeded to evaluate the scope of this oxidative cyclization with respect to a variety of oxygen‐linked 1,n‐enynes (Scheme 2A). Aryl substituents bearing electron‐donating groups, halogen atoms (F, Cl, Br, I) or boronic ester group (Bpin) at the alkyne terminus (R^1^) were well tolerated, affording the corresponding bicyclo[3.1.0] lactones (**1**–**12**) in good to excellent yields. Notably, the position of a methoxy group on the aryl ring—whether *ortho*, *meta*, or *para*—had little influence on the reaction efficiency, as demonstrated by the consistently high yields obtained for products **3**–**5**. On the other hand, substrates with strong electron‐withdrawing substituents on the aromatic rings, including esters (**13**, **14**), cyano (**15**), trifluoromethyl (**16**), trifluoromethoxy (**17**), acetyl (**18**), and aldehyde (**19**) groups, also underwent smooth cyclization under the standard conditions, resulting the corresponding products in moderate yields. Furthermore, oxygen‐linked 1,6‐enynes containing multisubstituted phenyl rings (**20**, **21**), 1‐naphthyl (**22**), 2‐naphthyl (**23**), piperonyl (**24**), and n‐propyl (**25**) substituents were also compatible, delivering the 3,5‐fused bicyclic products in 55%–84% yields. Enynes featuring a mono‐, di‐, and tri‐substituted alkenes can be effectively transformed into multi‐substituted bicyclo[3.1.0] hexane derivatives (**26**–**28**) in high yields. Particularly noteworthy is the successful cyclization of a congested, tri‐substituted (*E*)‐alkene substrate (**28a**), which proceeded smoothly to provide cyclopropane‐fused γ‐lactone **28** in 87% yield. This result stands in marked contrast to prior Pd^II^/Pd^IV^ systems [66, 68], where analogous trisubstituted enynes failed to react due to prohibitive steric hindrance. Moreover, this transformation exhibited clean inversion of the starting olefin geometry—a stereospecific outcome that further distinguishes it from metallocarbene or allene‐based pathways [31, 32, 33, 34, 35, 36, 37, 38, 39, 40, 41, 42, 43, 44, 45, 46, 47, 48, 55, 56, 57, 58, 59, 60]. These findings underscore the unique capacity of our peroxide‐based strategy to access previously elusive, sterically congested bicyclo[n.1.0]alkane architectures, thereby significantly expanding the structural diversity accessible via Pd‐catalyzed enyne oxidative cyclization. When *γ*‐substituted enyne **29a** was subjected to the reaction conditions, the desired lactone **29** was obtained in 82% yield with 3:1 diastereoselectivity. This stereochemical outcome is consistent with that observed in previously reported Pd^II^/Pd^IV^ catalytic systems, wherein the *endo*/*exo* insertion pathway is governed by the electronic character of the alkene moiety [66]. In addition, the methodology was successfully applied to 1,7‐enynes, enabling the synthesis of bicyclo[4.1.0]hexane‐based lactones **30** and **31**, thereby demonstrating the utility of this approach for accessing structurally distinct bicyclic frameworks.

We next sought to explore the generality of this oxidative cyclization by extending the substrate class to nitrogen‐linked 1,6‐enynes (Scheme 2B). Following a minor reoptimization of the reaction conditions (Table S7), a range of nitrogen‐tethered enynes—varying in both the alkynyl substituent and the presence or absence of a carbonyl group adjacent to the nitrogen atom—were found to be suitable substrates. This structural modification of enynes enabled the efficient synthesis of nitrogen‐containing bicyclo[3.1.0] lactams and pyrrolidines (**32**–**40**) in yields ranging from 61% to 86%. The effective construction of these nitrogen heterocycles significantly broadens the applicability of this method, offering practical access to rigid, polycyclic nitrogen‐containing scaffolds that hold potential relevance in medicinal chemistry and related fields.

### 2.3 Asymmetric Exploration of This Oxidative Cyclization

**SCHEME 3:**
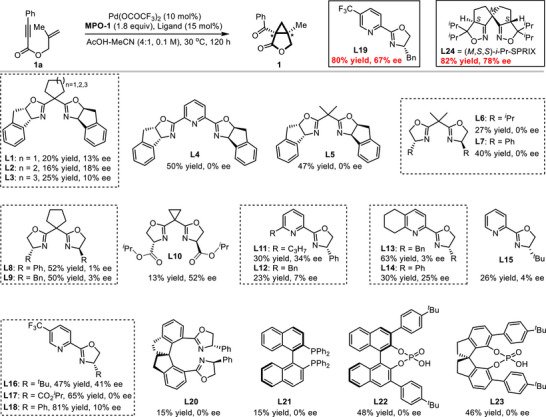
Catalytic asymmetric investigation. (a)Reaction conditions: **1a** (0.10 mmol, 1.0 equiv), Pd(TFA)_2_ (0.01 mmol, 10 mol%), Ligand (0.015 mmol, 15 mol%), MPO‐1 (0.18 mmol, 1.8 equiv), AcOH/MeCN (0.1 m, v/v=4:1), 30°C, 120 h, under Ar atmosphere; Yield of isolated product. (b)The ee values were determined by HPLC.

We also briefly examined the catalytic asymmetric version of this oxidative cyclization using **1a** as the template substrate (Scheme 3, for details, see, Table S10). An initial evaluation of chiral bisoxazoline ligands (**L1**–**L9**) with Pd(OCOCF_3_)_2_ afforded the product **1** in low yields with poor enantiocontrol. While a serine‐derived bisoxazoline (**L10**) with ancillary coordination sites [83] improved the enantiomeric excess (ee) to 52%, the chemical yield was merely 13%. A systematic survey of chiral pyridine‐ and quinoline‐oxazoline ligands (**L11**–**L19** and others in Table S10) subsequently identified **L19** as the optimal ligand, furnishing the bicyclic lactone 1 in 80% yield and 67% ee. In contrast, other ligand classes, including SpiroBOX (**L20**), phosphine (**L21**), and phosphoric acid derivatives (**L22** and **L23**), were found to be ineffective under the standard conditions. Notably, when the SPRIX ligand (**L24**)—which stands as the only privileged scaffold to have previously achieved high enantioselectivity (82% ee for **1**) in related Pd^II^/Pd^IV^‐catalyzed enyne oxidative cyclizations using 4 equivalents of PhI(OAc)_2_ as the oxidant [66, 84]—was deployed in combination with our unique oxidant system, the product **1** was obtained in 82% yield with 78% ee. The marked difference in enantioselectivity achieved with the same SPRIX ligand implies that the oxidant structure serves as an important and previously overlooked factor in stereocontrol. Our results reveal that the tether‐tunable oxidant can modulate the palladium coordination sphere during the Pd^II^/Pd^IV^ catalysis, thereby exerting a measurable and distinct influence on enantioselectivity. This tether‐tunable strategy represents a hitherto unappreciated dimension for stereochemical intervention in high‐valent palladium catalysis.

## 3 Mechanism Analysis

**SCHEME 4:**
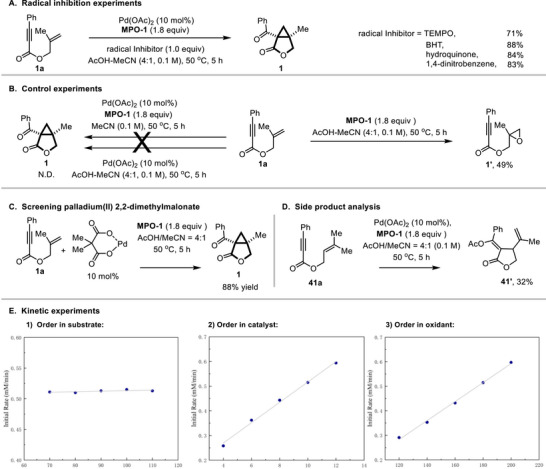
Mechanistic studies.

To elucidate the mechanism of the Pd(II)‐catalyzed oxidative cyclization, a comprehensive mechanistic investigation was undertaken (Scheme 4). Radical inhibition experiments employing stoichiometric quantities of scavengers such as 2,2,6,6‐tetramethyl‐1‐piperidinyloxy (TEMPO), butylated hydroxytoluene (BHT), hydroquinone, or 1,4‐dinitrobenzene revealed no significant suppression of product formation, with yields remaining between 71% and 88% (Scheme 4A, for details, see Table S11). These results decisively exclude the involvement of radical intermediates in the catalytic cycle. A set of control experiments was conducted to delineate the essential roles of the reaction components (Scheme 4B). The reaction failed to proceed in the absence of acetic acid, indicating that AcOH acts not only as the solvent but also as an essential reagent for the incorporation of acetoxy group in a key acetoxypalladation step [85]. The indispensability of both palladium and oxidant was further confirmed by the absence of cyclopropane formation when either component was excluded from the reaction mixture. Notably, the epoxide byproduct **1'** was not detected under our standard conditions. This observation underscores the efficacy of the Pd/MPO‐1 system in diverting the pathway toward the desired cyclopropanation, effectively suppressing a competing epoxidation. Moreover, the use of palladium(II) 2,2‐dimethylmalonate as the catalyst afforded the product in 88% yield (Scheme 4C), comparable to that achieved with Pd(OAc)_2_, supporting its identity as the operative catalytic species. Furthermore, the use of 3‐methylbut‐2‐en‐1‐yl 3‐phenylpropiolate (**41a**) yielded exclusively the *β*‐hydride elimination product (**41'**) (Scheme 4D), consistent with the intermediacy of a Pd(II)‐alkyl species formed via acetoxypalladation and subsequent olefin insertion. Kinetic analysis through initial rate measurements established a zero‐order dependence on the substrate and a first‐order dependence on both the Pd(II) catalyst and the oxidant (Scheme 4E), indicating that oxidation to form a Pd^IV^ intermediate is the turnover‐limiting step (TLS).

**SCHEME 5:**
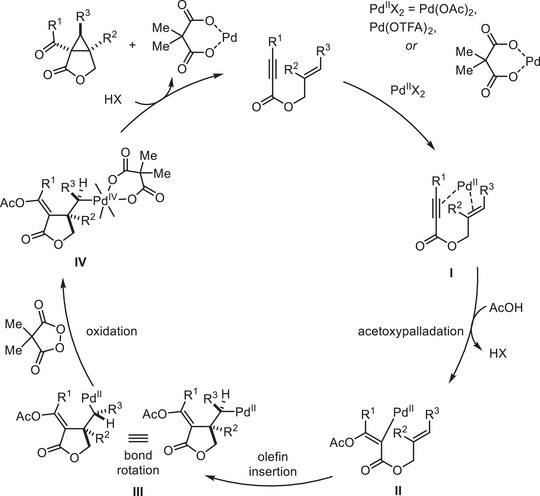
Proposed mechanism.

Based on literature precedents [64, 65, 66, 67, 68, 69, 70] and our experimental observations, a plausible mechanism for the Pd^II^‐catalyzed oxidative cyclization is proposed (Scheme 5). The catalytic cycle begins with acetoxypalladation of the alkyne moiety (**I**), generating a vinyl–Pd^II^ intermediate (**II**). Then, intramolecular olefin insertion affords an alkyl–Pd(II) species (**III**). Subsequent oxidation of **III** by the cyclic diacyl peroxide yields a Pd^IV^ intermediate (**IV**), in which the constrained structure of the coordinated dicarboxylate unit preorganizes the Pd^IV^ center in a conformation that disfavors competing C–OAc reductive elimination. Instead, the electron‐rich tethered vinyl acetate moiety attacks the Pd^IV^‐bound carbon in the S_N_2‐type fashion, leading to cyclopropane formation with inversion of configuration and concomitant regeneration of the Pd^II^ catalyst. Hydrolysis of the resulting bicyclic intermediate finally furnishes the observed bicyclo[n.1.0]alkane product. The diminished yields observed with electron‐withdrawing aryl substituents at the alkyne terminus are consistent with reduced nucleophilicity of the vinyl acetate moiety, further supporting the S_N_2 pathway for cyclopropanation. Notably, while related systems often report trace amounts of acetoxylated byproducts arising from competing C–OAc reductive elimination from the Pd^IV^ intermediate [64, 65, 66, 67, 68, 69, 70], our reaction exclusively affords the cyclopropane product without any detectable C–OAc coupling. This high selectivity is attributed to the conformational constraints and tether‐directed coordination imposed by the dicarboxylate unit, which disfavors the alternative C–OAc bond‐forming pathway [77, 78]. Nevertheless, given the documented complexities in analogous systems, [67, 68] further mechanistic studies are warranted to fully elucidate the operative pathway.

## 4 Greenness and Biocompatibility Evaluation

**SCHEME 6:**
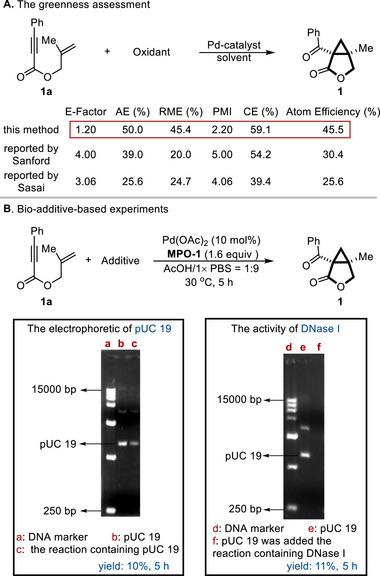
The greenness and biocompatibility of this method.

To assess the environmental impact [86, 87] and biocompatibility [88] of our developed protocol, we conducted a comprehensive evaluation using six established green metrics [89, 90, 91, 92, 93, 94] alongside a series of bio‐additive‐based experiments [95, 96]. The calculated values for environmental factor (E‐factor) [89], atom economy (AE) [90], reaction mass efficiency (RME) [91], process mass intensity (PMI) [92], carbon efficiency (CE) [93], and atom efficiency [94] collectively demonstrated a superior sustainability profile of our method relative to conventional approaches (Scheme 6A), underscoring its reduced waste generation and enhanced resource utilization. We next evaluated the biocompatibility under physiologically relevant conditions. When the reaction was performed in a solvent system of acetic acid and phosphate‐buffered saline (AcOH/PBS = 1:9) with oscillation over 5 h in the presence of plasmid pUC19, the target product was obtained in 10% yield. Agarose gel electrophoresis of the post‐reaction mixture showed that the plasmid remained intact (lane c, Scheme 6B), with no detectable fragmentation. In a separate experiment, DNase I was introduced into the reaction mixture at the outset. After 5 h, the product was isolated in 11% yield. Subsequent addition of plasmid pUC19 to this post‐reaction mixture resulted in rapid degradation, as evidenced by agarose gel electrophoresis (lane f, Scheme 6B). This confirms that DNase I retained its full enzymatic activity throughout the synthetic transformation. These findings, which combine rigorous green chemistry metrics with direct biocompatibility testing, position our methodology as a sustainable strategy with prospective utility in interdisciplinary contexts spanning organic synthesis and chemical biology.

## 5 Conclusion

In summary, we have developed a Pd‐catalyzed oxidative cyclopropanation of 1,n‐enynes employing cyclic diacyl peroxides as bifunctional reagents that serve as both tether‐tunable oxidants and spatially controlled dicarboxylate anions. This strategy affords efficient and divergent access to structurally diverse bicyclo[n.1.0]alkanes, including sterically congested and heteroatom‐embedded variants, with broad functional group tolerance. Preliminary enantioselective studies using a SPRIX ligand and a trifluoromethyl‐substituted pyridine‐oxazoline ligand afforded the corresponding product in up to 78% and 67% ee, respectively, highlighting the potential for further asymmetric optimization. Mechanistic studies support a Pd^II^/Pd^IV^ pathway wherein theconformational constraint and tether‐directed coordination imposed by the cyclic malonyl peroxide effectively suppresses competing C–OAc reductive elimination, thereby favoring stereospecific cyclopropanation. The method also exhibits favorable green chemistry metrics and retains biocompatibility with biomolecules such as plasmid pUC 19 and DNase I. We anticipate the present study will not only enable the preparation and exploration of highly functionalized bicyclic cyclopropanes in medicinal chemistry and related fields, but also establish a general platform for the use of structurally defined, tether‐tunable peroxides in high‐valent palladium catalysis, inspiring further innovation in selective transformations. Further applications of this strategy are currently underway in our laboratory.

## Funding

The Shenzhen Medical Research Fund (No. D2501008), the National Natural Science Foundation of China (No. 22271069), and the Shenzhen Science and Technology Program (Nos. JCYJ20240813105110014 and GXWD20231130100539001).

## Conflicts of Interest

The authors declare no conflict of interest.

## Supporting information




**Supporting File**: advs74077‐sup‐0001‐SuppMat.pdf.

## Data Availability

The data that support the findings of this study are available in the supplementary material of this article.
