# Supplementary material for: Spatial Control Over Tether‐Tunable Coordination on Palladium for Divergent Synthesis of Bicyclo[n.1.0]alkanes via Oxidative Cyclopropanation of Enynes
Source: Adv Sci (Weinh). 2026 Jan 30;13(19):e22444. doi: 10.1002/advs.202522444 (PMC13045495; doi:10.1002/advs.202522444)
Supplement: Supplementary file 1 — Supporting File: advs74077‐sup‐0001‐SuppMat.pdf. [file ADVS-13-e22444-s001.pdf]

Supporting Information

Spatial Control over Tether-Tunable Coordination on Palladium for Divergent Synthesis of Bicyclo[n.1.0]alkanes via Oxidative Cyclopropanation of Enynes

*Ting Yuan and Lei Shi**

School of Science (Shenzhen), School of Chemistry and Chemical Engineering,

Harbin Institute of Technology, Harbin, 150001, China

E-mail: lshi@hit.edu.cn

Table of Contents

1. General Information S1
2. Condition Optimization S2
Table S1. Optimization on oxidants. S2
Table S2. Optimization on solvents S3
Table S3. Optimization on solvents ratio and concentration. S4
Table S4. Optimization on palladium catalysts. S5
Table S5. Optimization on catalyst loading and reaction time. S6
Table S6. Optimization on oxidant amount and temperature. S7
Table S7. Optimization of the synthesis of 3-azabicyclo[3.1.0]hexanes. S8
3. Preparation of substrates S8
3.1 General procedure for the synthesis of aryl alkynyl carboxylic acids from aryl iodides. S8
3.2 General procedure A for the preparation of enyne derivatives. S9
3.3 General procedure B for the preparation of *N*-Ts-enyne derivatives S9
4 General procedure for the oxidative cyclization of enynes. S15
4.1 General procedure for the synthesis of bicycle[3.1.0]hexanes. S15
4.2 General procedure for the synthesis of aza-bicycle[3.1.0]hexanes. S21
5 Gram-scale synthesis S24
6 Sensitivity assessment S24
7 Catalytic asymmetric investigation S26
8 Mechanism experiments S28
8.1 Effect of radical inhibitors S28
8.2 Control experiments S28
8.3 Screening palladium(II) 2,2-dimethylmalonate S31
8.4 Side product analysis S31
8.5 Kinetic experiments S33
9 Biological compatibility experiments S37
9.1 The stability of nucleic acid in the oxidation S37
9.2 The activity of DNase I in the reaction S38
10. Green chemistry metrics analysis S39
11 Reference S42
12 Copies of NMR spectra S43

1. General Information

Unless otherwise noted, all experiments were carried out under Ar atmosphere. Commercially available reagents and starting materials were used without further purification. All the solvents including *N*,*N*-dimethylformamide (DMF), 1,2-dimethoxyethane (DME), dimethyl sulfoxide (DMSO), 1,2-dichloroethane (DCE), dichloromethane (DCM), acetonitrile (MeCN), toluene (PhMe), acetic acid (AcOH), tetrahydrofuran (THF), ethyl acetate (EA), petroleum ether (PE) and acetone were used as received without purification. Flash column chromatography was performed using 200-300 mesh silica gel. Schlenk tubes were purchased from Synthware.

All reactions were monitored by TLC and visualized by UV lamp (254 nm). ^{1}H NMR (400 MHz) and ^{13}C NMR (101 MHz) spectra were obtained on Zhongke-Niujin ((Quantum-I Plus 400M)) nuclear magnetic resonance spectrometers. Coupling constants are reported in Hertz (Hz). Data for ^{1}H-NMR spectra were reported as follows: chemical shift (ppm, referenced to protium; s = singlet, br s= broad singlet, d = doublet, t = triplet, q = quartet, p = pentet, dd = doublet of doublets, td = triplet of doublets, m = multiplet, coupling constant (Hz), and integration). Data for ^{1}H-NMR were reported in (ppm) relative to residual solvent peak ($CDCl_3$: 7.26 ppm, $(CD_3)_2SO$: 2.50 ppm) and $(CD_3)_2CO$: 2.05 ppm). Data for ^{13}C-NMR were reported in (ppm) relative to residual solvent peak ($CDCl_3$: 77.16 ppm, $(CD_3)_2SO$: 39.52 ppm and $(CD_3)_2CO$: 29.84 ppm). HRMS (ESI) was recorded using an Agilent 6520 accurate-Mass Q-TOF spectrometer. The enantiomeric excesses were determined by HPLC analysis using an Agilent 1260 Infinity II LC system (column Daicel Co. CHIRALCEL AS-H; eluent: hexane/2-propanol).

2. Condition Optimization

Table S1. Optimization on oxidants.

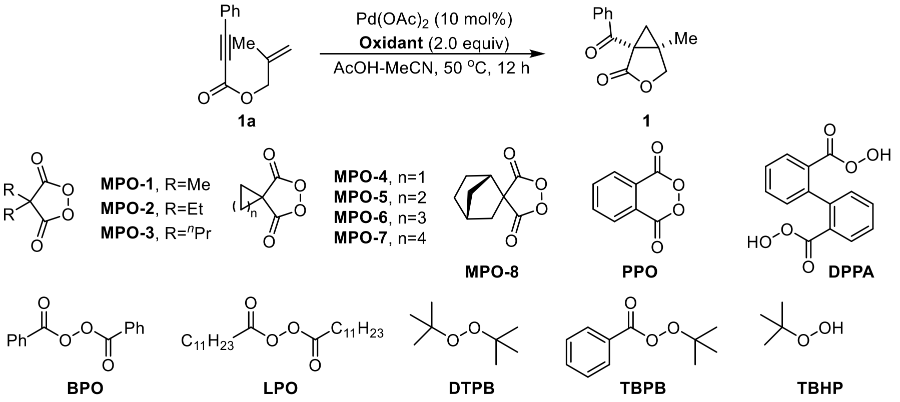

Entry	Oxidant	Yield (**1**, %)	Entry	Oxidant	Yield (**1**, %)
**1**	**MPO-1**	**73**	9	PPO	43
2	MPO-2	68	10	DPPA	trace
3	MPO-3	64	11	BPO	trace
4	MPO-4	60	12	LPO	N.D.
5	MPO-5	61	13	DTPB	N.D.
6	MPO-6	65	14	TBPB	N.D.
7	MPO-7	55	15	TBHP	N.D.
8	MPO-8	51	16	H_2O_2	N.D.

[a]Reaction conditions: **1a** (0.1 mmol, 1.0 equiv.), $Pd(OAc)_2$ (0.01 mmol, 10 mol%), Oxidant (0.2 mmol, 2.0 equiv) and AcOH/MeCN (0.1 M, v/v = 1:1), 50 °C, 12h, under Ar atmosphere. [b]isolated yield.

Table S2. Optimization on solvents.

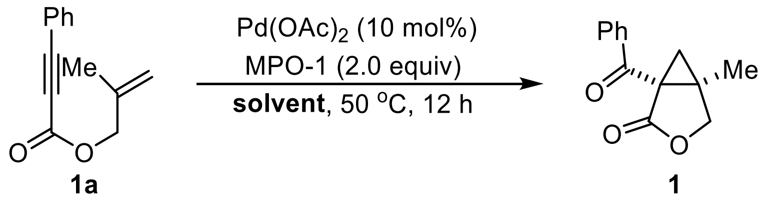

Entry	Solvent	Yield (**1**, %)
1	AcOH	69
2	AcOH-CH_2Cl_2 (1:1)	55
3	AcOH-toluene (1:1)	31
4	AcOH-EtOAc (1:1)	47
5	AcOH-THF (1:1)	33
6	AcOH-DMF (1:1)	51
**7**	**AcOH-MeCN (1:1)**	**73**
8	AcOH-acetone (1:1)	43
9	AcOH-CF_3CH_2OH (1:1)	61
10	AcOH-$CF_3CH(OH)CF_3$ (1:1)	63

[a]Reaction conditions: **1a** (0.1 mmol, 1.0 equiv.), $Pd(OAc)_2$ (0.01 mmol, 10 mol%), MPO-1 (0.2 mmol, 2.0 equiv) and solvent (0.1 M), 50 ^oC, 12h, under Ar atmosphere. [b]isolated yield.

Table S3. Optimization on solvents ratio and concentration.

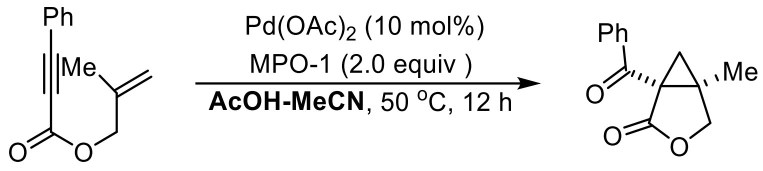

Entry	c (M)	AcOH/MeCN (v / v)	Yield (**1**, %)
1	0.1	1 : 1	73
2	0.1	1 : 2	65
3	0.1	2 : 1	76
**4**	**0.1**	**4 : 1**	**84**
5	0.1	9 : 1	81
6	0.1	14 : 1	80
7	0.2	4 : 1	80
8	0.05	4 : 1	77

[a]Reaction conditions: **1a** (0.1 mmol, 1.0 equiv.), $Pd(OAc)_2$ (0.01 mmol, 10 mol%), MPO-1 (0.2 mmol, 2.0 equiv) and AcOH/MeCN (0.1 M), 50 °C, 12h, under Ar atmosphere. [b]isolated yield.

Table S4. Optimization on palladium catalysts.

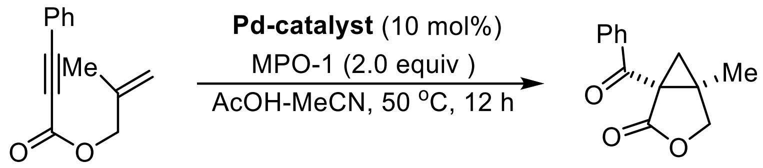

1

Entry	Pd-catalyst	Yield (**1**, %)
**1**	**$Pd(OAc)_2$**	**84**
2	$PdCl_2$	63
3	$Pd(TFA)_2$	79
4	$Pd(MeCN)_2Cl_2$	68

[a]Reaction conditions: **1a** (0.1 mmol, 1.0 equiv.), catalyst (0.01 mmol, 10 mol%), MPO-1 (0.2 mmol, 2.0 equiv) and AcOH/MeCN (0.1 M, v/v=4:1), 50 °C, 12h, under Ar atmosphere. [b]isolated yield.

Table S5. Optimization on catalyst loading and reaction time.

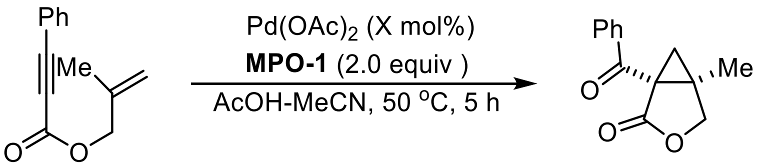

1a

1

Entry	X mol%	Time (h)	Yield (**1**, %)
1	10	12	84
2	10	6	83
**3**	**10**	**5**	**90**
4	10	4	75
5	5	5	29
6	5	12	61
7	7	12	72
8	9	12	80
10	12	12	83

[a]Reaction conditions: **1a** (0.1 mmol, 1.0 equiv.), $Pd(OAc)_2$, MPO-1 (0.2 mmol, 2.0 equiv) and AcOH/MeCN (0.1 M, v/v=4:1), 50 °C, under Ar atmosphere. [b]isolated yield.

Table S6. Optimization on oxidant amount and temperature.

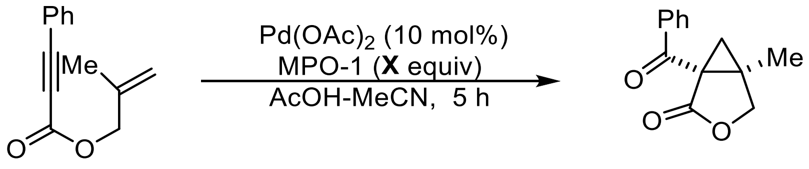

1a **1**

Entry	X equiv	Temp. (°C)	Con. (%)	Yield (**1**, %)
1	2.0	50	>99	90
2	1.0	50	51	43
3	1.2	50	69	57
4	1.5	50	90	79
5	1.6	50	>99	90
**6**	**1.8**	50	**>99**	**91**
7	1.8	70	>99	82
8	1.8	30	88	75

[a]Reaction conditions: **1a** (0.1 mmol, 1.0 equiv.), $Pd(OAc)_2$ (0.01 mmol, 10 mol%), MPO-1 and AcOH/MeCN (0.1 M, v/v=4:1), 5h, under Ar atmosphere. [b]isolated yield.

Table S7. Optimization of the synthesis of 3-azabicyclo[3.1.0]hexanes.

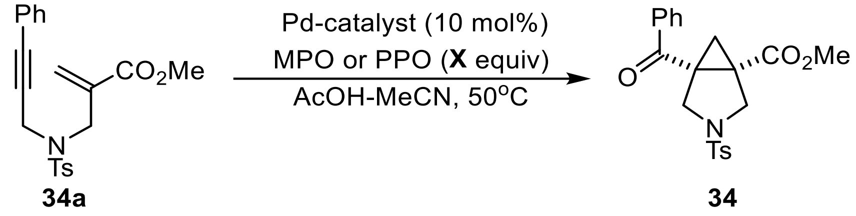

Entry	Pd-catalyst	MPO or PPO	X equiv	Time (h)	Yield (**34**, %)
1	$Pd(OAc)_2$	MPO-1	2.0	5	11
2	$PdCl_2$	MPO-1	2.0	5	<5
3	$Pd(TFA)_2$	MPO-1	2.0	5	51
4	$Pd(TFA)_2$	MPO-6	2.0	5	32
5	$Pd(OAc)_2$	PPO	2.0	5	<5
7	$Pd(TFA)_2$	MPO-1	2.0	12	83
8	$Pd(TFA)_2$	MPO-1	1.8	12	78

[a]Reaction conditions: **34a** (0.1 mmol, 1.0 equiv.), Pd-catalyst (0.01 mmol, 10 mol%), MPO or PPO and AcOH/MeCN (0.1 M, v/v=4:1), 50°C, under Ar atmosphere. [b]isolated yield.

3. Preparation of substrates

3.1 General procedure for the synthesis of aryl alkynyl carboxylic acids from aryl iodides.

A dry round bottom flask flushed with argon and equipped with a magnetic stirrer bar and a septum was charged with aryl iodide (5.0 mmol), 1,5-diazabicyclo(5,4,0)undec-5-ene (1.83 g, 12 mmol, 2.4 equiv.) and $Pd(PPh_3)_4$ (144 mg, 0.13 mmol, 2.5 mol%) in DMSO (6 mL). The solution of propiolic acid (420 mg, 6.0 mmol, 1.2 equiv.) in DMSO (6 mL) was added and the mixture was stirred at 25 °C for 12 h. The reaction mixture was diluted with EA (25.0 mL), and extracted with $NaHCO_3$ (sat. aq.). The aqueous layer was separated, acidified to pH < 2.0 by adding cold HCl (1 N), and extracted with CH_2Cl_2. The combined organic layers were dried with anhydrous Na_2SO_4, filtered, and concentrated under reduced pressure. The resulting crude product was purified by column chromatography on silica gel.[1]

3.2 General procedure A for the preparation of enyne derivatives.

A dry round bottom flask equipped with a magnetic stirrer bar and a septum was charged with propiolic acid (1.0 equiv) and allyl alcohol or amide (1.4 equiv) in CH_2Cl_2. A solution of 4-dimethylaminopyridine (10 mol %) and *N,N'*-diisopropylcarbodiimide (1.2 equiv) in CH_2Cl_2 (total: 0.25 M) was added at 0 ℃. The reaction mixture was stirred for 6-8 h at 25 ℃ and filtered through a short plug of silica gel, which was rinsed with PE/EA = 2:1. The filtrate was concentrated under reduced pressure and the crude product was purified by column chromatography on silica gel (PE/EA).[2]

3.3 General procedure B for the preparation of *N*-Ts-enyne derivatives.

A dry round bottom flask equipped with a magnetic stirrer bar and a septum was charged with 2-propyn-1-amine (1.0 equiv) and triethylamine (2.0 equiv) in CH_2Cl_2 (0.25 M). Tosyl chloride (1.2 equiv) was added slowly at 0 ℃ and then the reaction mixture was stirred for 8 h at 25 ℃. The crude product was purified by column chromatography on silica gel (PE/EA). A dry round bottom flask equipped with a magnetic stirrer bar and a septum was charged with the above obtained amide (1.0 equiv), 2-(bromomethyl)acrylate (1.2 equiv) and K_2CO_3 (3.0 equiv.) in acetone (0.1 M). The reaction mixture was heated to reflux for 2 h and after cooling to room temperature, the mixture was filtered and the solid was washed with acetone. The filtrate was concentrated and the residue was purified by silica gel column chromatography to afford the propargylated product. A dry round bottom flask flushed with argon and equipped with a magnetic stirrer bar and a septum was charged with CuI (0.01 equiv), $Pd(PPh_3)_2Cl_2$ (0.02 equiv) and the propargylated product (1.0 equiv.) in triethylamine (0.25 M). After stirring for 5 min at 25 ℃, aryl iodide (1.5 equiv) was added and the reaction mixture was stirred for 8 h at 25 ℃. The formed ammonium salt was removed by filtration and washed with Et_2O several times. The combined organic layer was dried with anhydrous Na_2SO_4 and concentrated under reduced pressure, and the residue was purified by flash chromatography on silica gel (PE/EA).[3]

The compounds **1a, 3a, 5a, 9a, 23a, 25a-26a, 28a-30a, 32a-34a** are known in previous reports.[2-7]

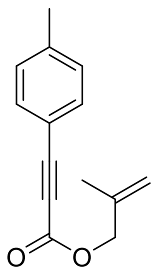

2-Methylallyl 3-(*p*-tolyl)propiolate (2a): yellow oil; 78% yield; **^{1}H NMR (400 MHz, $CDCl_3$)** δ 7.48 (d, *J* = 8.2 Hz, 2H), 7.17 (d, *J* = 8.2 Hz, 2H), 5.09 – 5.03 (m, 1H), 5.01 – 4.98 (m, 1H), 4.64 (s, 2H), 2.37 (s, 3H), 1.81 (s, 3H); **^{13}C NMR (101 MHz, $CDCl_3$)** δ 154.02, 141.44, 139.16, 133.10, 129.45, 116.51, 114.13, 87.17, 80.22, 69.15, 21.79, 19.60; **HRMS (ESI)** m/z calcd for $C_{14}H_{14}NaO_2^+$ [M+Na]$^+$: 237.0886, found 237.0884.

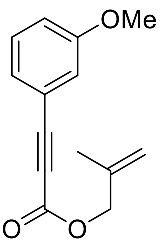

2-Methylallyl 3-(3-methoxyphenyl)propiolate (4a): colorless oil; 66% yield; **^{1}H NMR (400 MHz, CDCl$_3$)** δ 7.27 (t, *J* = 7.9 Hz, 1H), 7.18 (dt, *J* = 7.6, 1.2 Hz, 1H), 7.10 (dd, *J* = 2.8, 1.4 Hz, 1H), 6.99 (ddd, *J* = 8.3, 2.7, 1.1 Hz, 1H), 5.06 (q, *J* = 1.2 Hz, 1H), 4.98 (td, *J* = 1.6, 0.8 Hz, 1H), 4.65 (s, 2H), 3.80 (d, *J* = 1.0 Hz, 3H), 1.81 (d, *J* = 1.1 Hz, 3H); **^{13}C NMR (101 MHz, CDCl$_3$)** δ 159.45, 153.87, 139.12, 129.80, 125.63, 120.57, 117.68, 117.52, 114.24, 86.56, 80.25, 69.29, 55.46, 19.61; **HRMS (ESI)** m/z calcd for $C_{14}H_{14}NaO_3^+$ [M+Na]$^+$: 253.0835, found 253.0834.

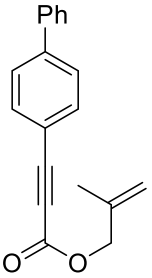

2-Methylallyl 3-([1,1'-biphenyl]-4-yl)propiolate (6a): yellow oil; 89% yield; **^{1}H NMR (400 MHz, CDCl$_3$)** δ 7.67 (d, *J* = 8.2 Hz, 2H), 7.63 – 7.57 (m, 4H), 7.46 (t, *J* = 7.5 Hz, 2H), 7.42 – 7.36 (m, 1H), 5.09 (d, *J* = 1.9 Hz, 1H), 5.03 – 5.01 (m, 1H), 4.68 (s, 2H), 1.84 (s, 3H); **^{13}C NMR (101 MHz, CDCl$_3$)** δ 153.95, 143.56, 139.86, 139.14, 133.62, 129.07, 128.26, 127.32, 127.22, 118.37, 114.23, 86.69, 81.18, 69.28, 19.63; **HRMS (ESI)** m/z calcd for $C_{19}H_{16}NaO_2^+$ [M+Na]$^+$: 299.1043, found 299.1044.

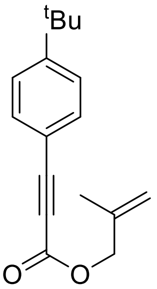

2-Methylallyl 3-(4-(*tert*-butyl)phenyl)propiolate (7a): colorless oil; 82% yield; **^{1}H NMR (400 MHz, CDCl$_3$)** δ 7.56 – 7.50 (m, 2H), 7.42 – 7.36 (m, 2H), 5.07 – 5.05 (m, 1H), 5.01 – 4.98 (m, 1H), 4.65 (s, 2H), 1.81 (t, *J* = 1.1 Hz, 3H), 1.31 (s, 9H); **^{13}C NMR (101 MHz, CDCl$_3$)** δ 154.47, 154.07, 139.19, 133.02, 125.74, 116.56, 114.15, 87.18, 80.21, 69.18, 35.13, 31.13, 19.62; **HRMS (ESI)** m/z calcd for $C_{17}H_{20}NaO_2^+$ [M+Na]$^+$: 279.1356, found 279.1353.

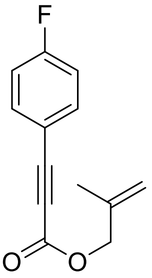

2-Methylallyl 3-(4-fluorophenyl)propiolate (8a): colorless oil; 51% yield; **^{1}H NMR (400 MHz, CDCl$_3$)** δ 7.62 – 7.53 (m, 2H), 7.11 – 7.01 (m, 2H), 5.06 – 5.04 (m, 1H), 4.98 (t, *J* = 1.5 Hz, 1H), 4.64 (s, 2H), 1.80 (d, *J* = 1.2 Hz, 3H); **^{13}C NMR (101 MHz, CDCl$_3$)** δ 163.99 (d, *J* = 253.8 Hz), 153.74, 139.05, 135.41, 135.33, 116.32, 116.10, 115.74 (d, *J* = 3.7 Hz), 114.23, 85.52, 80.43, 69.26, 19.56; **^{19}F NMR (376 MHz, CDCl$_3$)** δ -106.24, -106.26; **HRMS (ESI)** m/z calcd for $C_{13}H_{11}FNaO_2^+$ [M+Na]$^+$: 241.0635, found 241.0633.

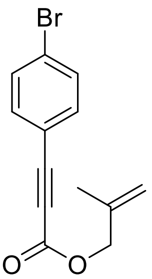

2-Methylallyl 3-(4-bromophenyl)propiolate (10a): yellow solid; 53% yield; **^{1}H NMR (400 MHz, CDCl$_3$)** δ 7.55 – 7.48 (m, 2H), 7.47 – 7.40 (m, 2H), 5.06 – 5.04 (m, 1H), 4.98 (t, *J* = 1.5 Hz, 1H), 4.64 (s, 2H), 1.80 (s, 3H); **^{13}C NMR (101 MHz, CDCl$_3$)** δ 153.67, 139.00, 134.40, 132.09, 125.60, 118.58, 114.35, 85.32, 81.46, 69.38, 19.61; **HRMS (ESI)** m/z calcd for $C_{13}H_{11}BrNaO_2^+$ [M+Na]$^+$: 300.9835, found 300.9835.

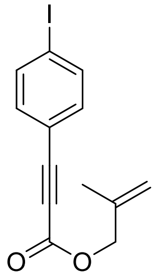

2-Methylallyl 3-(4-iodophenyl)propiolate (11a): white solid; 61% yield; **^{1}H NMR (400 MHz, $CDCl_3$)** δ 7.77 – 7.70 (m, 2H), 7.33 – 7.26 (m, 2H), 5.07 – 5.05 (m, 1H), 5.00 (t, *J* = 1.5 Hz, 1H), 4.65 (s, 2H), 1.81 (t, *J* = 1.2 Hz, 3H); **^{13}C NMR (101 MHz, $CDCl_3$)** δ 153.73, 139.04, 138.02, 134.34, 119.16, 114.39, 97.72, 85.52, 81.70, 69.43, 19.65; **HRMS (ESI)** m/z calcd for $C_{13}H_{11}INaO_2^+$ [M+Na]$^+$: 348.9696, found 348.9697.

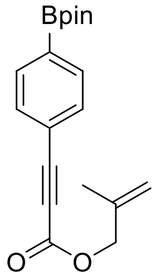

2-Methylallyl-3-(4-(4,4,5,5-tetramethyl-1,3,2-dioxaborolan-2-yl)phenyl)propiolate (12a): colorless oil; 61% yield; **^{1}H NMR (400 MHz, $CDCl_3$)** 7.80 (d, *J* = 8.2 Hz, 2H), 7.60 – 7.56 (m, 2H), 5.07 – 5.05 (m, 1H), 4.99 (t, *J* = 1.5 Hz, 1H), 4.65 (s, 2H), 1.81 (s, *J* = 1.1 Hz, 3H), 1.34 (s, 12H)**; ^{13}C NMR (100 MHz, $CDCl_3$)** δ 153.88, 139.12, 134.80, 132.16, 122.14, 114.30, 86.58, 84.33, 81.42, 69.36, 24.99, 19.65; **HRMS (ESI)** m/z calcd for $C_{19}H_{23}BNaO_4^+$ [M+Na]$^+$: 349.1582, found 349.1584.

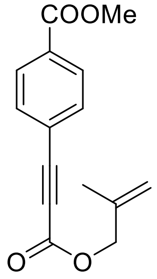

Methyl 4-(3-((2-methylallyl)oxy)-3-oxoprop-1-yn-1-yl)benzoate (13a): colorless oil; 80% yield; **^{1}H NMR (400 MHz, $CDCl_3$)** δ 8.07 – 8.01 (m, 2H), 7.68 – 7.62 (m, 2H), 5.06 – 5.04 (m, 1H), 5.00 (t, *J* = 1.5 Hz, 1H), 4.66 (s, 2H), 3.93 (s, 3H), 1.81 (t, *J* = 1.2 Hz, 3H); **^{13}C NMR (101 MHz, $CDCl_3$)** δ 166.20, 153.57, 138.99, 132.97, 131.84, 129.75, 124.19, 114.44, 85.11, 82.53, 69.52, 52.58, 19.63; **HRMS (ESI)** m/z calcd for $C_{15}H_{14}NaO_4^+$ [M+Na]$^+$: 281.0784, found 281.0785.

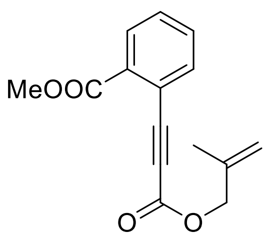

Methyl 2-(3-((2-methylallyl)oxy)-3-oxoprop-1-yn-1-yl)benzoate (14a): yellow oil; 61% yield; **^{1}H NMR (400 MHz, $CDCl_3$)** δ 8.02 (dd, *J* = 7.6, 1.7 Hz, 1H), 7.76 – 7.59 (m, 1H), 7.60 – 7.43 (m, 2H), 5.08 – 5.05 (m, 1H), 4.99 (d, *J* = 2.0 Hz, 1H), 4.66 (s, 2H), 3.95 (s, 3H), 1.81 (d, *J* = 1.5 Hz, 3H); **^{13}C NMR (101 MHz, $CDCl_3$)** δ 165.94, 153.87, 139.14, 135.28, 133.23, 132.11, 130.91, 130.31, 120.32, 114.16, 85.00, 84.59, 69.32, 52.55, 19.65; **HRMS (ESI)** m/z calcd for $C_{15}H_{14}NaO_4^+$ [M+Na]$^+$: 281.0784, found 281.0784.

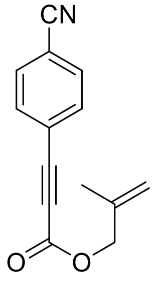

2-Methylallyl 3-(4-cyanophenyl)propiolate (15a): white solid; 72% yield; **^{1}H NMR (400 MHz, $CDCl_3$)** δ 7.68 (s, 4H), 5.07 (t, *J* = 1.2 Hz, 1H), 5.03 – 5.00 (m, 1H), 4.67 (s, 2H), 1.81 (d, J = 1.1 Hz, 3H).; **^{13}C NMR (101 MHz, $CDCl_3$)** δ 153.26, 138.85, 133.45, 132.37, 124.54, 117.97, 114.62, 114.20, 83.68, 83.57, 69.70, 19.63; **HRMS (ESI)** m/z calcd for $C_{14}H_{11}NNaO_2^+$ [M+Na]$^+$: 248.0682, found 248.0684.

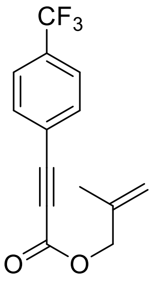

2-Methylallyl 3-(4-(trifluoromethyl)phenyl)propiolate (16a): yellow oil; 86% yield; **^{1}H NMR (400 MHz, CDCl$_3$)** δ 7.70 (d, *J* = 8.4 Hz, 2H), 7.64 (d, *J* = 8.6 Hz, 2H), 5.07 (t, *J* = 1.2 Hz, 1H), 5.01 (t, *J* = 1.5 Hz, 1H), 4.67 (s, 2H), 1.82 (t, *J* = 1.1 Hz, 3H); **^{13}C NMR (101 MHz, CDCl$_3$)** δ 153.48, 138.96, 133.33, 132.86, 132.54, 132.21, 125.71, 125.67, 123.66 (q, *J* = 272.4 Hz), 123.56, 114.52, 84.40, 82.18, 69.59, 19.64; **^{19}F NMR (376 MHz, CDCl$_3$)** δ -63.16; **HRMS (ESI)** m/z calcd for $C_{14}H_{11}F_3NaO_2^+$ [M+Na]$^+$: 291.0603, found 291.0606.

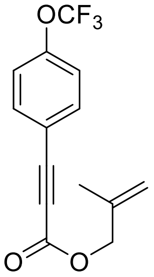

2-Methylallyl 3-(4-(trifluoromethoxy)phenyl)propiolate (17a): yellow oil; 82% yield; **^{1}H NMR (400 MHz, CDCl$_3$)** δ 7.67 – 7.59 (m, 2H), 7.25 – 7.19 (m, 2H), 5.07 – 5.05 (m, 1H), 5.02 – 4.99 (m, 1H), 4.68 – 4.63 (m, 2H), 1.81 (t, *J* = 1.2 Hz, 3H); **^{13}C NMR (101 MHz, CDCl$_3$)** δ 153.66, 150.78, 139.04, 134.87, 120.99, 120.39 (q, *J* = 258.7 Hz), 118.32, 114.37, 84.85, 81.20, 77.48, 77.16, 76.84, 69.44, 19.61; **^{19}F NMR (376 MHz, CDCl$_3$)** δ -57.73; **HRMS (ESI)** m/z calcd for $C_{14}H_{11}F_3NaO_3^+$ [M+Na]$^+$: 307.0552, found 307.0550.

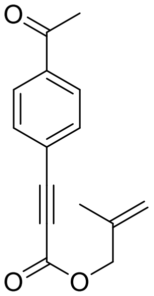

2-Methylallyl 3-(4-acetylphenyl)propiolate (18a): colorless oil; 80% yield; **^{1}H NMR (400 MHz, CDCl$_3$)** δ 7.96 (dd, J = 8.4, 1.5 Hz, 2H), 7.68 (dd, J = 8.4, 1.5 Hz, 2H), 5.09 – 5.06 (m, 1H), 5.02 – 5.00 (m, 1H), 4.67 (s, 2H), 2.62 (d, J = 1.5 Hz, 3H), 1.82 (d, J = 1.1 Hz, 3H); **^{13}C NMR (101 MHz, CDCl$_3$)** δ 197.25, 153.59, 138.98, 138.19, 133.24, 128.44, 124.33, 114.50, 85.04, 82.76, 69.57, 26.87, 19.66; **HRMS (ESI)** m/z calcd for $C_{15}H_{14}NaO_3^+$ [M+Na]$^+$: 265.0835, found 265.0831.

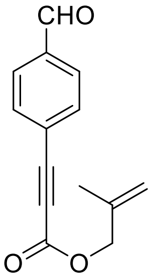

2-Methylallyl 3-(4-formylphenyl)propiolate (19a): green oil; 61% yield; **^{1}H NMR (400 MHz, CDCl$_3$)** δ 10.01 (s, 1H), 7.86 (dd, *J* = 8.1, 1.7 Hz, 2H), 7.70 (dd, *J* = 8.3, 1.7 Hz, 2H), 5.05 – 5.02 (m, 1H), 4.99 – 4.96 (m, 1H), 4.63 (s, 2H), 1.78 (s, 3H); **^{13}C NMR (100 MHz, CDCl$_3$)** δ 191.17, 153.29, 138.84, 137.11, 133.44, 129.57, 125.49, 114.39, 84.57, 83.08, 69.46, 19.52; **HRMS (ESI)** m/z calcd for $C_{14}H_{12}NaO_3^+$ [M+Na]$^+$: 251.0679, found 251.0681.

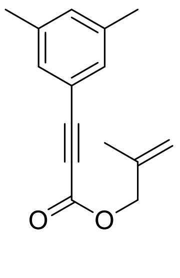

2-Methylallyl 3-(3,5-dimethylphenyl)propiolate (20a): yellow oil; 86% yield; **^{1}H NMR (400 MHz, CDCl$_3$)** δ 7.58 – 7.32 (m, 3H), 5.25 (t, *J* = 1.4 Hz, 1H), 5.19 – 5.17 (m, 1H), 4.83 (s, 2H), 2.49 (s, 6H), 2.00 (s, 3H); **^{13}C NMR (101 MHz, CDCl$_3$)** δ 154.00, 139.19, 138.34, 132.76, 130.78, 119.25, 114.09, 87.26, 80.00, 76.84, 69.16, 21.12, 19.60; **HRMS (ESI)** m/z calcd for $C_{15}H_{16}NaO_2^+$ [M+Na]$^+$: 251.1043, found 251.1043.

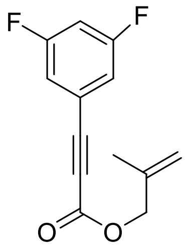

2-Methylallyl 3-(3,5-difluorophenyl)propiolate (21a): yellow oil; 76% yield; **^{1}H NMR (400 MHz, CDCl$_3$)** δ 7.11 (dt, *J* = 5.9, 2.3 Hz, 2H), 6.93 (tt, *J* = 8.8, 2.4 Hz, 1H), 5.06 (s, 1H), 5.01 (d, *J* = 1.5 Hz, 1H), 4.66 (s, 2H), 1.81 (s, 3H); **^{13}C NMR (101 MHz, CDCl$_3$)** δ 164.02 (d, *J* = 12.9 Hz), 161.53 (d, *J* = 12.9 Hz), 153.30, 138.89, 122.36 (t, *J* = 11.6 Hz), 116.16 (d, *J* = 7.9 Hz), 115.96 (d, *J* = 8.3 Hz), 114.57, 107.16 (t, *J* = 25.1 Hz), 83.34, 81.70, 77.48, 77.16, 76.84, 69.64, 19.64; **^{19}F NMR (376 MHz, CDCl$_3$)** δ -108.06, -108.08, -108.10; **HRMS (ESI)** m/z calcd for $C_{13}H_{10}F_2NaO_2^+$ [M+Na]$^+$: 259.0541, found 259.0540.

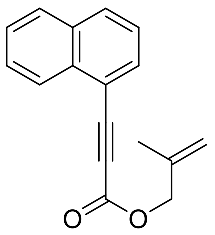

2-Methylallyl 3-(naphthalen-1-yl)propiolate (22a): yellow solid; 63% yield; **^{1}H NMR (400 MHz, CDCl$_3$)** δ 8.38 – 8.31 (m, 1H), 7.95 (d, *J* = 8.3 Hz, 1H), 7.87 (ddd, *J* = 7.1, 5.4, 1.0 Hz, 2H), 7.63 (ddd, *J* = 8.3, 6.8, 1.5 Hz, 1H), 7.56 (ddd, *J* = 8.2, 6.8, 1.4 Hz, 1H), 7.47 (dd, *J* = 8.3, 7.1 Hz, 1H), 5.12 (t, *J* = 1.2 Hz, 1H), 5.03 (t, *J* = 1.4 Hz, 1H), 4.72 (s, 2H), 1.86 (t, *J* = 1.1 Hz, 3H); **^{13}C NMR (101 MHz, CDCl$_3$)** δ 154.05, 139.25, 133.77, 133.22, 133.12, 131.51, 128.61, 127.80, 127.05, 125.88, 125.21, 117.26, 114.21, 85.22, 85.05, 69.33, 19.68; **HRMS (ESI)** m/z calcd for $C_{17}H_{14}NaO_2^+$ [M+Na]$^+$: 273.0886, found 273.0885.

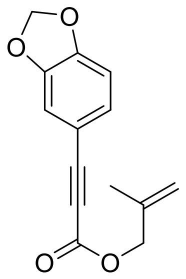

2-Methylallyl 3-(benzo[*d*][1,3]dioxol-5-yl)propiolate (24a): white solid; 69% yield; **^{1}H NMR (400 MHz, CDCl$_3$)** δ 7.14 (dt, J = 8.1, 1.3 Hz, 1H), 6.98 (t, J = 1.3 Hz, 1H), 6.78 (d, J = 8.1 Hz, 1H), 5.04 (d, J = 1.0 Hz, 1H), 5.01 - 4.93 (m, 1H), 4.62 (s, 2H), 1.79 (d, J = 1.0 Hz, 3H).; **^{13}C NMR (101 MHz, CDCl$_3$)** δ 153.92, 150.10, 147.67, 139.15, 128.95, 114.40, 114.07, 112.54, 108.79, 101.84, 87.07, 79.48, 69.09, 19.56; **HRMS (ESI)** m/z calcd for $C_{14}H_{12}NaO_4^+$ [M+Na]$^+$: 267.0628, found 267.0627.

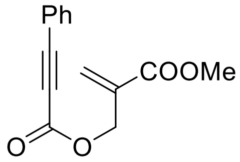

Methyl 2-(((3-phenylpropioloyl)oxy)methyl)acrylate (27a): colorless oil; 57% yield; **^{1}H NMR (400 MHz, CDCl$_3$)** δ 7.62 – 7.56 (m, 2H), 7.49 – 7.43 (m, 1H), 7.41 – 7.35 (m, 2H), 6.44 (d, *J* = 0.9 Hz, 1H), 5.96 (d, *J* = 1.0 Hz, 1H), 4.97 (t, *J* = 1.2 Hz, 2H), 3.81 (s, 3H); **^{13}C NMR (101 MHz, CDCl$_3$)** δ 165.60, 153.58, 134.48, 133.20, 130.94, 128.74, 119.54, 87.20, 80.36, 63.83, 52.33; **HRMS (ESI)** m/z calcd for $C_{14}H_{12}NaO_4^+$ [M+Na]$^+$: 267.0628, found 267.0627.

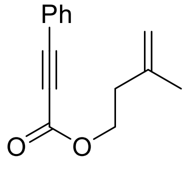

3-Methylbut-3-en-1-yl 3-phenylpropiolate (31a): colorless oil; 61% yield; **^{1}H NMR (400 MHz, CDCl$_3$)** δ 7.58 (d, *J* = 7.5 Hz, 2H), 7.44 (t, *J* = 7.4 Hz, 1H), 7.36 (t, *J* = 7.6 Hz, 2H), 4.85 (s, 1H), 4.79 (s, 1H), 4.35 (t, *J* = 7.0 Hz, 2H), 2.43 (t, *J* = 7.0 Hz, 2H), 1.78 (s, 3H); **^{13}C NMR (101 MHz, CDCl$_3$)** δ 154.15, 141.21, 133.09, 130.73, 128.66, 119.73, 112.76, 86.37, 80.72, 64.34, 36.55, 22.62; **HRMS (ESI)** m/z calcd for $C_{14}H_{14}NaO_2^+$ [M+Na]$^+$: 237.0886, found 237.0885.

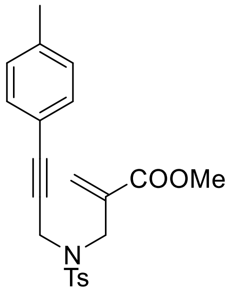

Methyl 2-(((4-methyl-*N*-(3-(p-tolyl)prop-2-yn-1-yl)phenyl)sulfonamido)methyl)acrylate (35a): brown yellow solid; 81% yield; **^{1}H NMR (400 MHz, $CDCl_3$)** δ 7.83 (d, *J* = 8.0 Hz, 2H), 7.39 – 7.23 (m, 2H), 7.12 – 6.93 (m, 4H), 6.46 (d, *J* = 1.5 Hz, 1H), 6.10 – 5.98 (m, 1H), 4.34 (s, 2H), 4.18 (s, 2H), 3.80 (s, 3H), 2.39 (s, 3H), 2.36 (s, 3H); **^{13}C NMR (101 MHz, $CDCl_3$)** δ 166.38, 143.74, 138.75, 136.06, 135.02, 131.53, 129.72, 128.99, 127.95, 127.77, 119.08, 86.16, 81.04, 52.19, 47.04, 37.93, 21.56; **HRMS (ESI)** m/z calcd for $C_{22}H_{23}NNaO_4S^+$ [M+Na]$^+$:420.1240, found 420.1238.

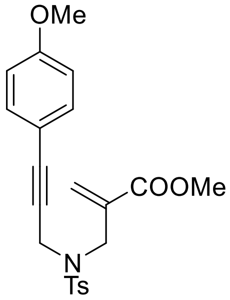

Methyl 2-(((*N*-(3-(4-methoxyphenyl)prop-2-yn-1-yl)-4-methylphenyl)sulfonamido)methyl)acrylate (36a): brown yellow oil; 83% yield; **^{1}H NMR (400 MHz, $CDCl_3$)** δ 7.83 (d, *J* = 8.0 Hz, 2H), 7.37 – 7.25 (m, 2H), 7.05 (d, *J* = 8.7 Hz, 2H), 6.79 (d, *J* = 8.6 Hz, 2H), 6.46 (s, 1H), 6.03 (s, 1H), 4.33 (s, 2H), 4.18 (s, 2H), 4.33 (s, 3H), 4.18 (s, 3H), 2.40 (s, 3H); **^{13}C NMR (101 MHz, $CDCl_3$)** δ 166.41, 159.82, 143.72, 136.13, 135.05, 133.12, 129.70, 127.97, 127.75, 114.27, 113.86, 85.96, 80.35, 55.42, 52.20, 47.03, 37.98, 21.61; **HRMS (ESI)** m/z calcd for $C_{22}H_{23}NNaO_5S^+$ [M+Na]$^+$:436.1189, found 436.1188.

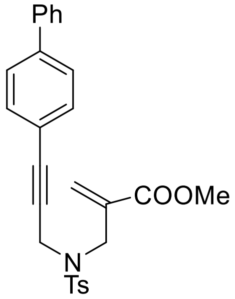

Methyl 2-(((*N*-(3-([1,1'-biphenyl]-4-yl)prop-2-yn-1-yl)-4-methylphenyl)sulfonamido)methyl)acrylate (37a): brown yellow oil; 87% yield; **^{1}H NMR (400 MHz, $CDCl_3$)** δ 7.84 – 7.80 (m, 2H), 7.59 – 7.54 (m, 2H), 7.51 – 7.42 (m, 4H), 7.40 – 7.34 (m, 1H), 7.29 (d, *J* = 8.0 Hz, 2H), 7.19 – 7.10 (m, 2H), 6.45 (d, *J* = 1.4 Hz, 1H), 6.03 (d, *J* = 1.4 Hz, 1H), 4.35 (s, 2H), 4.18 (s, 2H), 3.78 (s, 3H), 2.36 (s, 3H); **^{13}C NMR (101 MHz, $CDCl_3$)** δ 166.34, 143.78, 141.33, 140.20, 136.01, 134.97, 132.03, 129.73, 128.99, 127.93, 127.86, 127.81, 127.06, 126.88, 120.98, 85.88, 82.41, 52.19, 47.09, 37.94, 21.57; **HRMS (ESI)** m/z calcd for $C_{27}H_{25}NNaO_4S^+$ [M+Na]$^+$:482.1397, found 482.1398.

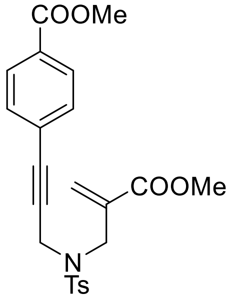

Methyl 4-(3-((*N*-(2-(methoxycarbonyl)allyl)-4-methylphenyl)sulfonamido)prop-1-yn-1-yl)benzoate (38a): pale yellow solid; 83% yield; **^{1}H NMR (400 MHz, $CDCl_3$)** δ 7.93 (dd, *J* = 8.3, 1.6 Hz, 2H), 7.82 (dd, *J* = 8.3, 1.7 Hz, 2H), 7.41 – 7.23 (m, 2H), 7.14 (dd, *J* = 8.4, 1.6 Hz, 2H), 6.45 (s, 1H), 6.03 (s, 1H), 4.36 (d, *J* = 1.5 Hz, 2H), 4.17 (d, *J* = 2.0 Hz, 2H), 3.94 (s, 3H), 3.79 (s, 3H), 2.36 (s, 3H); **^{13}C NMR (101 MHz, $CDCl_3$)** δ 166.43, 166.31, 143.89, 135.96, 134.91, 131.51, 129.87, 129.73, 129.36, 127.93, 127.87, 126.75, 85.18, 84.94, 52.39, 52.21, 47.18, 37.85, 21.56; **HRMS (ESI)** m/z calcd for $C_{23}H_{23}NNaO_6S^+$ [M+Na]$^+$:464.1138, found 464.1136.

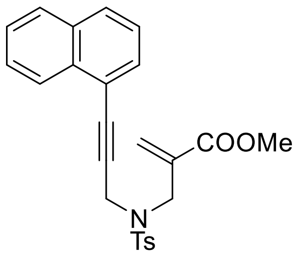

Methyl 2-(((4-methyl-*N*-(3-(naphthalen-1-yl)prop-2-yn-1-yl)phenyl)sulfonamido)methyl)acrylate (39a): 85% yield; brown yellow oil; **^{1}H NMR (400 MHz, $CDCl_3$)** δ 7.92 – 7.73 (m, 5H), 7.53 – 7.44 (m, 2H), 7.38 – 7.28 (m, 2H), 7.16 (d, *J* = 7.9 Hz, 2H), 6.46 (s, 1H), 6.06 (s, 1H), 4.49 (s, 2H), 4.25 (s, 2H), 3.77 (s, 3H), 2.14 (s, 3H); **^{13}C NMR (101 MHz, $CDCl_3$)** δ 166.39, 143.90, 135.97, 135.04, 133.10, 133.04, 130.76, 129.76, 129.08, 128.34, 128.00, 127.85, 126.85, 126.53, 125.95, 125.08, 119.87, 86.60, 84.31, 52.23, 47.16, 38.13, 21.41; **HRMS (ESI)** m/z calcd for $C_{25}H_{23}NNaO_4S^+$ $[M+Na]^+$:456.1240, found 456.1239.

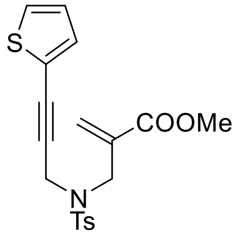

Methyl 2-(((4-methyl-*N*-(3-(thiophen-2-yl)prop-2-yn-1-yl)phenyl)sulfonamido)methyl)acrylate (40a): 81% yield; brown yellow oil; **^{1}H NMR (400 MHz, $CDCl_3$)** δ 7.80 (d, *J* = 8.3 Hz, 2H), 7.31 (d, *J* = 8.3 Hz, 2H), 7.26 – 7.18 (m, 1H), 7.01 – 6.86 (m, 2H), 6.44 (s, 1H), 6.01 (s, 1H), 4.35 (s, 2H), 4.14 (s, 2H), 3.79 (s, 3H), 2.39 (s, 3H); **^{13}C NMR (101 MHz, $CDCl_3$)** δ 166.22, 143.85, 135.73, 134.89, 132.41, 129.73, 127.77, 127.75, 127.43, 126.83, 121.91, 85.68, 79.19, 52.11, 47.09, 37.97, 21.58; **HRMS (ESI)** m/z calcd for $C_{19}H_{19}NNaO_4S_2^+$ $[M+Na]^+$:412.0648, found 412.0649.

4 General procedure for the oxidative cyclization of enynes.

4.1 General procedure for the synthesis of bicycle[3.1.0]hexanes.

A dry Schlenk tube flushed with argon equipped with a magnetic stirrer bar and a septum was charged with substrate (0.1 mmol, 1.0 equiv) and $Pd(OAc)_2$ (0.01 mmol, 10 mol%) in AcOH (0.8 mL) and MeCN (0.2 mL). **MPO-1** (0.18 mmol, 1.8 equiv) was added and the mixture was stirred at 50 ℃ for 5 h. The residue was then purified by flash chromatography on silica gel (PE/EA).

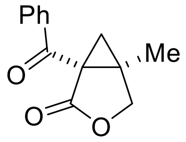

1-Benzoyl-5-methyl-3-oxabicyclo[3.1.0]hexan-2-one (1): white solid; 91% yield; **^{1}H NMR (400 MHz, $CDCl_3$)** δ 7.75 – 7.68 (m, 2H), 7.61 – 7.54 (m, 1H), 7.47 (dd, J = 8.4, 7.0 Hz, 2H), 4.40 (d, J = 2.2 Hz, 2H), 2.19 (d, J = 4.8 Hz, 1H), 1.42 (d, J = 4.9 Hz, 1H), 1.31 (s, 3H); **^{13}C NMR (100 MHz, $CDCl_3$)** δ 191.96, 174.06, 136.59, 133.60, 128.84, 128.60, 72.73, 40.32, 37.08, 22.85, 14.39.

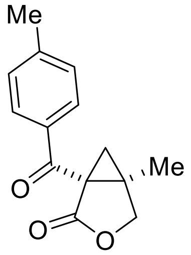

5-Methyl-1-(4-methylbenzoyl)-3-oxabicyclo[3.1.0]hexan-2-one (2): white solid; 89% yield; **^{1}H NMR (400 MHz, $CDCl_3$)** δ 7.75 – 7.64 (m, 2H), 7.41 – 7.24 (m, 2H), 4.53 – 4.40 (m, 2H), 2.46 (s, 3H), 2.24 (d, J = 4.9 Hz, 1H), 1.46 (d, J = 4.8 Hz, 1H), 1.36 (s, 3H); **^{13}C NMR (100 MHz, $CDCl_3$)** δ 191.37, 174.24, 144.71, 134.13, 129.60, 128.87, 72.79, 40.27, 36.74, 22.80, 21.85, 14.49. **HRMS (ESI)** m/z calcd for $C_{14}H_{14}NaO_3^+$ $(M+Na)^+$ 253.0835, found 253.0834.

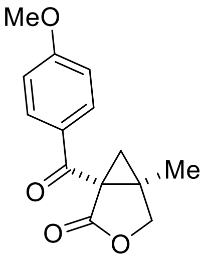

1-(4-Methoxybenzoyl)-5-methyl-3-oxabicyclo[3.1.0]hexan-2-one (3): white solid; 90% yield; **^{1}H NMR (400 MHz, $CDCl_3$)** δ 7.77 – 7.69 (m, 2H), 7.01 – 6.91 (m, 2H), 4.42 (d, *J* = 9.5 Hz, 1H), 4.37 (d, *J* = 9.5 Hz, 1H), 3.87 (s, 3H), 2.17 (d, J = 4.9 Hz, 1H), 1.40 (d, J = 4.9 Hz, 1H), 1.31 (s, 3H); **^{13}C NMR (100 MHz, $CDCl_3$)** δ 189.89, 174.42, 164.06, 131.21, 129.48, 114.18, 72.82, 55.69, 40.00, 36.29, 22.71, 14.54.

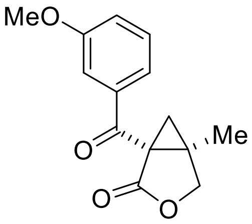

1-(3-Methoxybenzoyl)-5-methyl-3-oxabicyclo[3.1.0]hexan-2-one (4): pale yellow solid; 88% yield; **^{1}H NMR (400 MHz, $CDCl_3$)** δ 7.38 (t, J = 7.9 Hz, 1H), 7.30 (t, J = 2.3 Hz, 1H), 7.22 (ddd, J = 7.6, 1.7, 0.9 Hz, 1H), 7.16 – 7.09 (m, 1H), 4.46 – 4.36 (m, 2H), 3.85 (s, 3H), 2.20 (d, J = 4.9 Hz, 1H), 1.43 (d, J = 4.9 Hz, 1H), 1.33 (s, 3H); **^{13}C NMR (101 MHz, $CDCl_3$)** δ 191.85, 174.02, 160.05, 138.01, 129.84, 121.20, 120.07, 113.15, 72.78, 55.58, 40.53, 37.05, 22.87, 14.51; **HRMS (ESI)** m/z calcd for $C_{14}H_{14}NaO_4^+$ $(M+Na)^+$ 269.0784, found 269.0784.

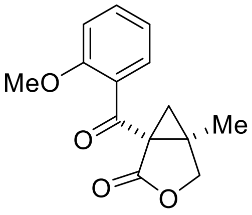

1-(2-Methoxybenzoyl)-5-methyl-3-oxabicyclo[3.1.0]hexan-2-one (5): pale yellow solid; 85% yield; **^{1}H NMR (400 MHz, $CDCl_3$)** δ 7.67 (dt, J = 7.6, 1.5 Hz, 1H), 7.51 (ddd, J = 9.0, 7.3, 1.7 Hz, 1H), 7.07 (td, J = 7.5, 1.1 Hz, 1H), 6.94 (d, J = 8.4 Hz, 1H), 4.36 (d, J = 9.0 Hz, 1H), 4.18 (d, J = 9.0 Hz, 1H), 3.87 (d, J = 1.2 Hz, 3H), 2.31 (d, J = 4.6 Hz, 1H), 1.38 (d, J = 4.8 Hz, 1H), 1.33 (s, 3H); **^{13}C NMR (101 MHz, $CDCl_3$)** δ 192.46, 174.56, 158.20, 134.56, 131.02, 127.16, 121.59, 111.46, 72.93, 55.49, 43.12, 38.65, 23.23, 14.03; **HRMS (ESI)** m/z calcd for $C_{14}H_{14}NaO_4^+$ $(M+Na)^+$ 269.0784, found 269.0781.

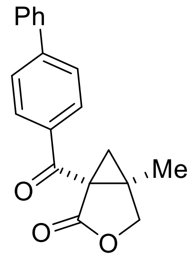

1-([1,1'-Biphenyl]-4-carbonyl)-5-methyl-3-oxabicyclo[3.1.0]hexan-2-one (6): pale yellow solid; 89% yield; **^{1}H NMR (400 MHz, $CDCl_3$)** δ 7.85 – 7.77 (m, 2H), 7.73 – 7.67 (m, 2H), 7.64 – 7.58 (m, 2H), 7.51 – 7.45 (m, 2H), 7.44 – 7.38 (m, 1H), 4.53 – 4.37 (m, 2H), 2.25 (d, *J* = 4.8 Hz, 1H), 1.47 (d, *J* = 4.9 Hz, 1H), 1.36 (s, 3H); **^{13}C NMR (101 MHz, $CDCl_3$)** δ 191.40, 174.25, 146.53, 139.87, 135.27, 129.36, 129.13, 128.51, 127.62, 127.48, 72.87, 40.39, 37.10, 22.99, 14.55; **HRMS (ESI)** m/z calcd for $C_{19}H_{16}NaO_3^+$ $(M+Na)^+$ 315.0992, found 315.0992.

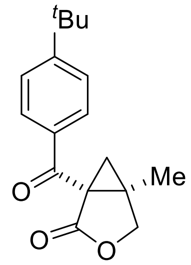

1-(4-(*Tert*-Butyl)benzoyl)-5-methyl-3-oxabicyclo[3.1.0]hexan-2-one (7): white solid; 90% yield; **^{1}H NMR (400 MHz, $CDCl_3$)** δ 7.71 – 7.64 (m, 2H), 7.52 – 7.45 (m, 2H), 4.45 – 4.35 (m, 2H), 2.18 (d, J = 4.9 Hz, 1H), 1.41 (d, J = 4.9 Hz, 1H), 1.32 (d, J = 8.2 Hz, 9H); **^{13}C NMR (101 MHz, $CDCl_3$)** δ 191.25, 174.31, 157.50, 133.87, 128.71, 125.88, 72.79, 40.24, 36.69, 35.28, 31.11, 22.62, 14.46; **HRMS (ESI)** m/z calcd for $C_{17}H_{20}NaO_3^+$ $(M+Na)^+$ 295.1305, found 295.1305.

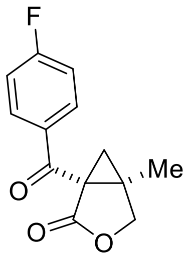

1-(4-Fluorobenzoyl)-5-methyl-3-oxabicyclo[3.1.0]hexan-2-one (8): white solid; 91% yield; **^{1}H NMR (400 MHz, $CDCl_3$)** δ 7.80 – 7.71 (m, 2H), 7.19 – 7.09 (m, 2H), 4.45 – 4.34 (m, 2H), 2.19 (d, J = 4.9 Hz, 1H), 1.44 (d, J = 4.9 Hz, 1H), 1.32 (s, 3H); **^{13}C NMR (100 MHz, $CDCl_3$)** δ 190.31, 173.99, 165.99 (d, J = 255.8 Hz), 133.01, 132.98, 131.42, 131.33, 116.21, 115.99, 72.78, 40.18, 37.07, 23.15, 14.42; **^{19}F NMR (376 MHz, CDCl3)** δ -103.92; **HRMS (ESI)** m/z calcd for $C_{13}H_{11}FNaO_3^+$ (M+Na)$^+$ 257.0584, found 257.0586.

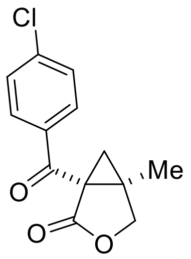

1-(4-Chlorobenzoyl)-5-methyl-3-oxabicyclo[3.1.0]hexan-2-one (9): white solid; 88% yield;**^{1}H NMR (400 MHz, $CDCl_3$)** δ 7.70 – 7.62 (m, 2H), 7.49 – 7.41 (m, 2H), 4.46 – 4.34 (m, 2H), 2.21 (d, *J* = 4.9 Hz, 1H), 1.46 (d, *J* = 4.9 Hz, 1H), 1.33 (s, 3H); **^{13}C NMR (100 MHz, $CDCl_3$)** δ 190.82, 173.88, 140.17, 134.97, 130.06, 129.23, 72.79, 40.27, 37.35, 23.28, 14.44.

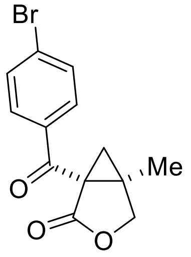

1-(4-Bromobenzoyl)-5-methyl-3-oxabicyclo[3.1.0]hexan-2-one (10): pale yellow solid; 86% yield; **^{1}H NMR (400 MHz, $CDCl_3$)** δ 7.65 – 7.54 (m, 4H), 4.42 (d, *J* = 9.7 Hz, 1H), 4.37 (d, *J* = 9.5 Hz, 1H), 2.20 (d, J = 4.9 Hz, 1H), 1.45 (d, J = 4.9 Hz, 1H), 1.32 (s, J = 1.0 Hz, 3H); **^{13}C NMR (100 MHz, $CDCl_3$)** δ 191.04, 173.84, 135.38, 132.20, 130.11, 128.86, 72.78, 40.26, 37.38, 23.28, 14.42; **HRMS (ESI)** m/z calcd for $C_{13}H_{11}BrNaO_3^+$ (M+Na)$^+$ 316.9784, found 316.9785.

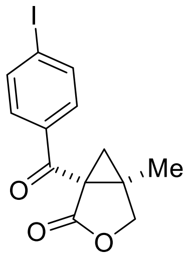

1-(4-Iodobenzoyl)-5-methyl-3-oxabicyclo[3.1.0]hexan-2-one (11): pale yellow solid; 81% yield; **^{1}H NMR (400 MHz, $CDCl_3$)** δ 7.87 – 7.81 (m, 2H), 7.46 – 7.39 (m, 2H), 4.42 (m, J = 9.6, 1.7 Hz, 2H), 2.21 (dd, J = 4.9, 1.5 Hz, 1H), 1.45 (dd, J = 4.9, 1.7 Hz, 1H), 1.33 (s, J = 1.7 Hz, 3H).; **^{13}C NMR (100 MHz, $CDCl_3$)** δ 191.38, 173.84, 138.22, 135.95, 129.96, 101.72, 72.80, 40.27, 37.44, 23.30, 14.46. **HRMS (ESI)** m/z calcd for $C_{13}H_{11}INaO_3^+$ (M+Na)$^+$ 364.9645, found 364.9641.

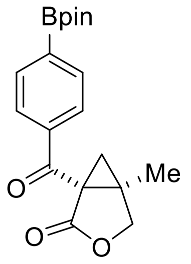

5-Methyl-1-(4-(4, 4, 5, 5-tetramethyl-1, 3, 2-dioxaborolan-2-yl)benzoyl)-3-oxabicyclo[3.1.0]hexan-2-one (12): colorless oil; 76% yield; **^{1}H NMR (400 MHz, $CDCl_3$)** δ 7.94 (d, *J* = 8.2 Hz, 2H), 7.71 (d, *J* = 8.3 Hz, 2H), 4.50 – 4.40 (m, 2H), 2.26 (d, *J* = 4.9 Hz, 1H), 1.48 (d, *J* = 4.9 Hz, 1H), 1.37 (d, *J* = 6.2 Hz, 15H); **^{13}C NMR (101 MHz, $CDCl_3$)** δ 192.42, 173.99, 138.60, 135.17, 127.58, 84.38, 72.77, 40.50, 37.34, 24.99, 24.95, 23.03, 14.46; **HRMS (ESI)** m/z calcd for $C_{19}H_{23}BNaO_5^+$ (M+Na)$^+$ 365.1531, found 365.1527.

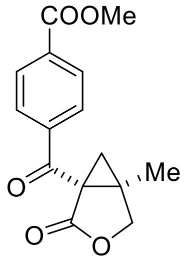

Methyl 4-(5-methyl-2-oxo-3-oxabicyclo[3.1.0]hexane-1-carbonyl)benzoate (13): white solid; 67% yield; **^{1}H NMR (400 MHz, CDCl$_3$)** δ 8.17 – 8.11 (m, 2H), 7.79 – 7.73 (m, 2H), 4.49 – 4.38 (m, 2H), 3.95 (d, *J* = 1.2 Hz, 3H), 2.27 (d, *J* = 4.8 Hz, 1H), 1.50 (d, *J* = 4.8 Hz, 1H), 1.37 (s, 3H); **^{13}C NMR (101 MHz, CDCl$_3$)** δ 191.98, 173.75, 166.15, 140.06, 134.29, 130.09, 128.49, 72.82, 52.70, 40.65, 38.03, 23.54, 14.48; **HRMS (ESI)** m/z calcd for $C_{15}H_{14}NaO_5^+$ 297.0733, found 297.0733.

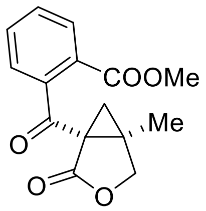

Methyl 2-(5-methyl-2-oxo-3-oxabicyclo[3.1.0]hexane-1-carbonyl)benzoate (14): white solid; 54% yield; **^{1}H NMR (400 MHz, CDCl$_3$)** δ 7.98 (dd, *J* = 7.8, 1.3 Hz, 1H), 7.63 (td, *J* = 7.5, 1.3 Hz, 1H), 7.52 (td, *J* = 7.7, 1.3 Hz, 1H), 7.29 (dd, *J* = 7.6, 1.3 Hz, 1H), 4.26 (d, *J* = 9.0 Hz, 1H), 4.19 (d, *J* = 9.0 Hz, 1H), 3.88 (s, 3H), 2.27 (d, *J* = 4.2 Hz, 1H), 1.63 (s, 3H), 1.59 (s, 1H); **^{13}C NMR (101 MHz, CDCl$_3$)** δ 198.69, 173.62, 166.98, 142.50, 133.04, 130.11, 129.55, 128.21, 127.57, 72.08, 52.66, 40.97, 40.60, 29.22, 13.35; **HRMS (ESI)** m/z calcd for $C_{15}H_{14}NaO_5^+$ $(M+Na)^+$ 297.0733, found 297.0735.

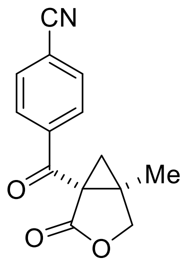

4-(5-Methyl-2-oxo-3-oxabicyclo[3.1.0]hexane-1-carbonyl)benzonitrile (15): white solid; 48% yield; **^{1}H NMR (400 MHz, CDCl$_3$)** δ 7.79 (s, 4H), 4.57 – 4.21 (m, 2H), 2.29 (d, *J* = 4.9 Hz, 1H), 1.54 (d, *J* = 4.9 Hz, 1H), 1.38 (s, 3H); **^{13}C NMR (101 MHz, CDCl$_3$)** δ 194.48, 173.53, 139.95, 132.70, 128.97, 117.88, 116.78, 72.85, 40.60, 38.64, 24.08, 14.45; **HRMS (ESI)** m/z calcd for $C_{14}H_{14}NNaO_3^+$ $(M+Na)^+$ 264.0631, found 264.0629.

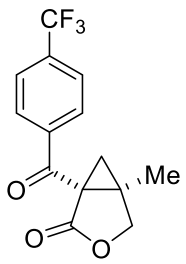

5-Methyl-1-(4-(trifluoromethyl)benzoyl)-3-oxabicyclo[3.1.0]hexan-2-one(16): white solid; 51% yield; **^{1}H NMR (400 MHz, CDCl$_3$)** δ 7.82 (d, J = 8.6 Hz, 2H), 7.75 (d, J = 8.3 Hz, 2H), 4.44 (q, J = 9.7 Hz, 2H), 2.28 (d, J = 4.9 Hz, 1H), 1.52 (d, J = 4.9 Hz, 1H), 1.37 (s, 3H); **^{13}C NMR (101 MHz, CDCl$_3$)** δ 191.54, 173.70, 139.51, 134.82 (d, J = 33.2 Hz), 128.93, 128.30, 126.00, 125.97, 124.83, 122.22, 119.42, 72.85, 40.60, 38.16, 23.69, 14.45; **^{19}F NMR (376 MHz, CDCl$_3$)** δ -63.19; **HRMS (ESI)** m/z calcd for $C_{14}H_{11}F_3NaO_3^+$ $(M+Na)^+$ 307.0552, found 307.0551.

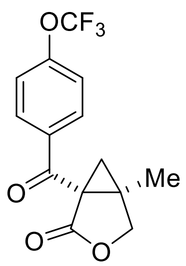

5-Methyl-1-(4-(trifluoromethoxy)benzoyl)-3-oxabicyclo[3.1.0]hexan-2-one (17): yellow oil; 69% yield; **^{1}H NMR (400 MHz, Chloroform-*d*)** δ 7.82 – 7.74 (m, 2H), 7.33 – 7.27 (m, 2H), 4.41 (q, *J* = 9.7 Hz, 2H), 2.22 (d, *J* = 4.9 Hz, 1H), 1.47 (d, *J* = 4.9 Hz, 1H), 1.34 (s, 3H); **^{13}C NMR (101 MHz, CDCl$_3$)** δ 190.53, 173.90, 152.97, 134.79, 130.75, 120.61, 120.37 (q, *J* = 259.1 Hz), 77.48, 77.16, 76.84, 72.83, 40.30, 37.46, 23.35, 14.43; **^{19}F NMR (376 MHz, CDCl$_3$)** δ -57.57; **HRMS (ESI)** m/z calcd for $C_{14}H_{11}F_3NaO_4^+$ $(M+Na)^+$ 323.0502, found 323.0502.

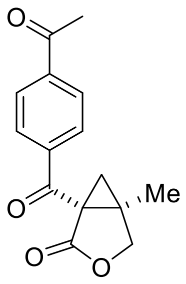

1-(4-Acetylbenzoyl)-5-methyl-3-oxabicyclo[3.1.0]hexan-2-one (18): colorless oil; 64% yield; **^{1}H NMR (400 MHz, CDCl$_3$)** δ 8.07 – 7.93 (m, 2H), 7.81 – 7.75 (m, 2H), 4.48 – 4.38 (m, 2H), 2.64 (s, 3H), 2.26 (d, *J* = 4.8 Hz, 1H), 1.50 (d, *J* = 4.9 Hz, 1H), 1.37 (s, 3H); **^{13}C NMR (101 MHz, CDCl$_3$)** δ 197.40, 191.94, 173.78, 140.44, 140.03, 128.77, 128.72, 72.83, 40.68, 38.10, 27.04, 23.58, 14.45; **HRMS (ESI)** m/z calcd for $C_{15}H_{14}NaO_4^+$ $(M+Na)^+$ 281.0784, found 281.0785.

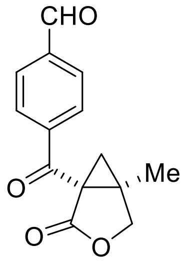

4-(5-Methyl-2-oxo-3-oxabicyclo[3.1.0]hexane-1-carbonyl)benzaldehyde (19): colorless oil; 60% yield; **^{1}H NMR (400 MHz, CDCl$_3$)** δ 10.14 (d, *J* = 1.1 Hz, 1H), 8.03 (dd, *J* = 8.3, 1.3 Hz, 2H), 7.91 – 7.85 (m, 2H), 4.54 – 4.41 (m, 2H), 2.33 (d, *J* = 4.8 Hz, 1H), 1.56 (d, *J* = 4.9 Hz, 1H), 1.42 (s, 3H); **^{13}C NMR (101 MHz, CDCl$_3$)** δ 192.04, 191.50, 173.69, 141.19, 139.27, 130.01, 129.08, 72.84, 40.75, 38.39, 23.80, 14.47; **HRMS (ESI)** m/z calcd for $C_{14}H_{12}NaO_4^+$ $(M+Na)^+$ 267.0628, found 267.0627.

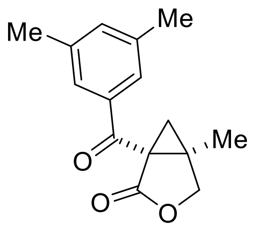

1-(3, 5-Dimethylbenzoyl)-5-methyl-3-oxabicyclo[3.1.0]hexan-2-one (20): pale yellow oil; 75% yield; **^{1}H NMR (400 MHz, CDCl$_3$)** δ 7.29 (d, *J* = 1.7 Hz, 2H), 7.22 (d, *J* = 0.8 Hz, 1H), 4.41 (d, *J* = 3.3 Hz, 2H), 2.36 (s, 6H), 2.20 (d, *J* = 4.8 Hz, 1H), 1.41 (d, *J* = 4.9 Hz, 1H), 1.32 (s, 3H); **^{13}C NMR (101 MHz, CDCl$_3$)** δ 192.35, 174.19, 138.54, 136.84, 135.44, 126.36, 72.73, 40.39, 36.99, 22.87, 21.42, 14.46; **HRMS (ESI)** m/z calcd for $C_{15}H_{16}NaO_3^+$ $(M+Na)^+$ 267.0992, found 267.0992.

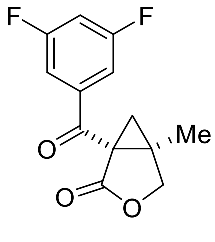

1-(3, 5-Difluorobenzoyl)-5-methyl-3-oxabicyclo[3.1.0]hexan-2-one(21): white solid; 55% yield; **^{1}H NMR (400 MHz, CDCl$_3$)** δ 7.25 – 7.16 (m, 2H), 7.04 (tt, *J* = 8.6, 2.5 Hz, 1H), 4.48 – 4.37 (m, 2H), 2.23 (d, *J* = 4.9 Hz, 1H), 1.51 (d, *J* = 5.0 Hz, 1H), 1.37 (s, 3H); **^{13}C NMR (101 MHz, CDCl$_3$)** δ 190.07, 173.41, 164.31 (d, J = 11.6 Hz), 161.75, 139.57, 111.74 (d, J = 7.5 Hz), 111.55 (d, J = 7.5 Hz), 108.99 (t, J = 25.1 Hz), 72.80, 40.49, 38.05, 23.68, 14.47.; **^{19}F NMR (376 MHz, CDCl$_3$)** δ -107.27, -107.29, -107.30; **HRMS (ESI)** m/z calcd for $C_{13}H_{10}F_2NaO_3^+$ $(M+Na)^+$ 275.0490, found 275.0487.

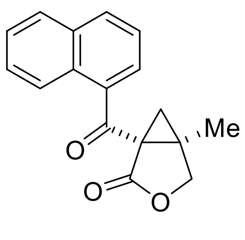

1-(1-Naphthoyl)-5-methyl-3-oxabicyclo[3.1.0]hexan-2-one(22): pale yellow solid; 82% yield; **^{1}H NMR (400 MHz, CDCl$_3$)** δ 8.48 (d, *J* = 8.4 Hz, 1H), 8.05 (td, *J* = 5.0, 2.2 Hz, 1H), 7.93 (d, *J* = 8.1 Hz, 1H), 7.70 – 7.46 (m, 4H), 4.53 – 4.39 (m, 2H), 2.44 (dd, *J* = 4.8, 2.1 Hz, 1H), 1.57 (dd, *J* = 4.8, 2.2 Hz, 1H), 1.52 (d, *J* = 2.3 Hz, 3H); **^{13}C NMR (101 MHz, CDCl$_3$)** δ 194.65, 173.66, 135.09, 134.22, 133.30, 130.53, 128.71, 128.28, 127.63, 126.89, 125.44, 124.34, 72.53, 42.19, 38.99, 24.40, 14.41; **HRMS (ESI)** m/z calcd for $C_{17}H_{14}NaO_3^+$ $(M+Na)^+$ 289.0835, found 289.0831.

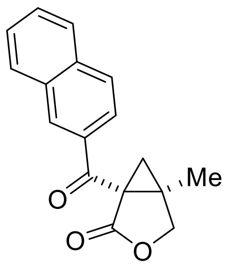

1-(2-Naphthoyl)-5-methyl-3-oxabicyclo[3.1.0]hexan-2-one (23): pale yellow solid; 84% yield; **^{1}H NMR (400 MHz, $CDCl_3$)** δ 8.29 – 8.16 (m, 1H), 7.92 (td, *J* = 15.8, 15.2, 8.0 Hz, 3H), 7.80 (dd, *J* = 8.6, 1.8 Hz, 1H), 7.59 (dddd, *J* = 21.4, 8.1, 6.9, 1.3 Hz, 2H), 4.48 (d, *J* = 1.1 Hz, 2H), 2.27 (d, *J* = 4.9 Hz, 1H), 1.49 (d, *J* = 4.8 Hz, 1H), 1.38 (s, 3H); **^{13}C NMR (101 MHz, $CDCl_3$)** δ 191.84, 174.19, 135.87, 133.97, 132.49, 130.57, 129.83, 128.97, 128.88, 127.97, 127.14, 124.17, 72.87, 40.45, 37.09, 23.13, 14.57.

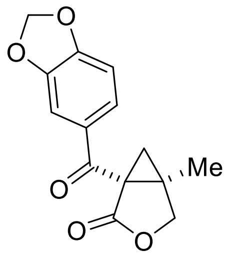

1-(Benzo[*d*][1,3]dioxole-5-carbonyl)-5-methyl-3-oxabicyclo[3.1.0]hexan-2-one (24): white solid; 81% yield; **^{1}H NMR (400 MHz, $CDCl_3$)** δ 7.34 – 7.26 (m, 2H), 6.90 (d, J = 8.1 Hz, 1H), 6.09 (q, J = 1.3 Hz, 2H), 4.48 – 4.37 (m, 2H), 2.18 (d, J = 4.9 Hz, 1H), 1.44 (d, J = 4.9 Hz, 1H), 1.35 (s, 3H); **^{13}C NMR (100 MHz, $CDCl_3$)** δ 189.62, 174.20, 152.46, 148.49, 131.32, 125.35, 108.56, 108.26, 102.21, 72.78, 40.11, 36.35, 22.82, 14.54. **HRMS (ESI)** m/z calcd for $C_{14}H_{12}NaO_5^+$ $(M+Na)^+$ 283.0577, found 283.0575.

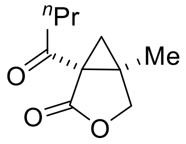

1-Butyryl-5-methyl-3-oxabicyclo[3.1.0]hexan-2-one (25): colorless oil; 77% yield; **^{1}H NMR (400 MHz, $CDCl_3$)** δ 4.24 (d, J = 9.4 Hz, 1H), 4.10 (d, J = 9.3 Hz, 1H), 3.03 (dt, J = 17.4, 7.3 Hz, 1H), 2.69 (dt, J = 17.4, 7.3 Hz, 1H), 2.12 (d, J = 4.3 Hz, 1H), 1.60 (h, J = 7.3 Hz, 2H), 1.37 (d, J = 4.3 Hz, 1H), 1.33 (s, 3H), 0.92 (t, J = 7.4 Hz, 3H); **^{13}C NMR (100 MHz, $CDCl_3$)** δ 201.70, 174.05, 72.32, 44.28, 40.35, 38.99, 26.59, 17.09, 13.76, 13.49.

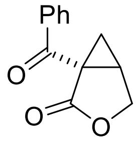

1-Benzoyl-3-oxabicyclo[3.1.0]hexan-2-one (26): white solid; 80% yield; **^{1}H NMR (400 MHz, $CDCl_3$)** δ 7.96 – 7.85 (m, 2H), 7.68 – 7.56 (m, 1H), 7.49 (dd, J = 8.5, 7.0 Hz, 2H), 4.59 (dd, J = 9.6, 4.7 Hz, 1H), 4.36 (d, J = 9.6 Hz, 1H), 2.84 (dt, J = 7.9, 4.9 Hz, 1H), 2.13 (dd, J = 7.9, 4.8 Hz, 1H), 1.46 (t, J = 5.0 Hz, 1H); **^{13}C NMR (101 MHz, $CDCl_3$)** δ 192.26, 172.91, 135.72, 133.87, 129.37, 128.69, 68.10, 35.96, 27.26, 19.40.

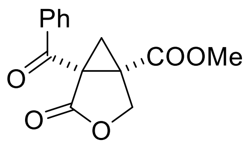

Methyl 5-benzoyl-4-oxo-3-oxabicyclo[3.1.0]hexane-1-carboxylate (27): white solid; 83% yield; **^{1}H NMR (400 MHz, $CDCl_3$)** δ 7.83 (dt, J = 8.3, 1.2 Hz, 2H), 7.67 – 7.57 (m, 1H), 7.49 (t, J = 7.8 Hz, 2H), 5.08 (d, J = 10.0 Hz, 1H), 4.45 (d, J = 10.0 Hz, 1H), 3.57 (s, 3H), 2.72 (d, J = 5.1 Hz, 1H), 1.73 (d, J = 5.1 Hz, 1H); **^{13}C NMR (101 MHz, $CDCl_3$)** δ 187.91, 170.82, 167.06, 135.03, 134.35, 129.11, 128.98, 68.23, 52.99, 43.00, 37.79, 21.92; **HRMS (ESI)** m/z calcd for $C_{14}H_{12}NaO_5^+$ $(M+Na)^+$ 283.0577, found 283.0574.

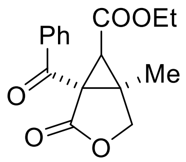

Ethyl 1-benzoyl-5-methyl-2-oxo-3-oxabicyclo[3.1.0]hexane-6-carboxylate (28): pale yellow oil; 87% yield; **^{1}H NMR (400 MHz, CDCl$_3$)** δ 7.80 – 7.73 (m, 2H), 7.63 – 7.51 (m, 1H), 7.47 (t, *J* = 7.8 Hz, 2H), 4.66 (d, *J* = 10.3 Hz, 1H), 4.47 (d, *J* = 10.3 Hz, 1H), 4.26 – 4.16 (m, 2H), 3.09 (s, 1H), 1.39 (s, 3H), 1.28 (t, *J* = 7.2 Hz, 3H); **^{13}C NMR (101 MHz, CDCl$_3$)** δ 190.22, 171.09, 166.62, 135.93, 134.05, 128.98, 128.77, 70.34, 62.03, 46.47, 42.44, 34.10, 16.06, 14.08; **HRMS (ESI)** m/z calcd for $C_{16}H_{16}NaO_5^+$ $(M+Na)^+$ 311.0890, found 311.0888.

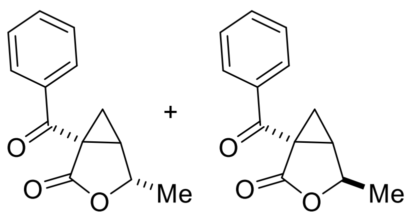

dr = 3:1

1-Benzoyl-4-methyl-3-oxabicyclo[3.1.0]hexan-2-one (29): yellow solid; 66% yield; **^{1}H NMR (400 MHz, CDCl$_3$)** δ 8.04 – 7.87 (m, 2H), 7.64 – 7.56 (m, 1H), 7.53 – 7.44 (m, 2H), 4.57 (q, J = 6.4 Hz, 1H), 2.65 (dd, J = 8.0, 5.2 Hz, 1H), 2.10 (dd, J = 8.0, 4.6 Hz, 1H), 1.59 (d, J = 6.5 Hz, 3H), 1.49 (d, J = 4.8 Hz, 1H).; **^{13}C NMR (100 MHz, CDCl$_3$)** δ 192.34, 172.28, 135.84, 133.78, 129.51, 128.57, 76.54, 36.62, 33.14, 22.38, 20.67; **HRMS (ESI)** m/z calcd for $C_{13}H_{12}NaO_3^+$ $(M+Na)^+$ 239.0679, found 239.0681.

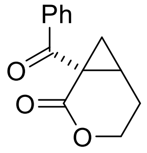

1-Benzoyl-3-oxabicyclo[4.1.0]heptan-2-one (30): colorless oil; 59% yield; **^{1}H NMR (400 MHz, CDCl$_3$)** δ 7.84 (dd, J = 8.1, 1.7 Hz, 2H), 7.55 (td, J = 7.2, 1.5 Hz, 1H), 7.45 (td, J = 7.7, 1.7 Hz, 2H), 4.45 (m, J = 12.1, 6.0, 1.5 Hz, 1H), 4.32 – 4.21 (m, 1H), 2.51 (m, J = 14.7, 5.9, 2.8 Hz, 1H), 2.14 (m, J = 21.6, 11.9, 3.6, 1.8 Hz, 3H), 1.86 (dt, J = 7.1, 3.5 Hz, 1H); **^{13}C NMR (100 MHz, CDCl$_3$)** δ 193.83, 168.92, 136.07, 133.29, 128.72, 128.68, 64.92, 33.83, 24.05, 20.43, 13.48.

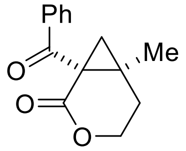

1-Benzoyl-6-methyl-3-oxabicyclo[4.1.0]heptan-2-one (31): colorless oil; 51% yield; **^{1}H NMR (400 MHz, CDCl$_3$)** δ 7.81 – 7.75 (m, 2H), 7.59 – 7.52 (m, 1H), 7.51 – 7.39 (m, 2H), 4.44 (ddd, *J* = 12.3, 6.1, 1.3 Hz, 1H), 4.23 (ddd, *J* = 13.4, 12.2, 3.7 Hz, 1H), 2.41 (td, *J* = 14.0, 13.4, 6.0 Hz, 1H), 2.13 (ddd, *J* = 14.8, 3.6, 1.4 Hz, 1H), 1.98 (d, *J* = 6.2 Hz, 1H), 1.92 (d, *J* = 6.2 Hz, 1H), 1.14 (s, 3H);**^{13}C NMR (101 MHz, CDCl$_3$)** δ 193.23, 169.23, 136.85, 133.28, 128.82, 128.51, 39.65, 30.01, 27.14, 19.60, 18.23; **HRMS (ESI)** m/z calcd for $C_{14}H_{14}NaO_3^+$ $(M+Na)^+$ 253.0835, found 253.0838.

4.2 General procedure for the synthesis of aza-bicycle[3.1.0]hexanes.

A dry Schlenk tube flushed with argon equipped with a magnetic stirrer bar and a septum was charged with substrate (0.1 mmol, 1.0 equiv) and Pd(TFA)$_2$ (0.01 mmol, 10 mol%) in AcOH (0.8 mL) and MeCN (0.2 mL). **MPO-1** (0.20 mmol, 2.0 equiv) was added and the mixture was stirred at 50 ℃ for 12 h. The residue was then purified by flash chromatography on silica gel (PE/EA).

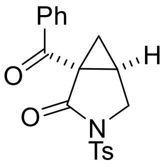

1- Benzoyl-3-tosyl-3-azabicyclo[3.1.0]hexan-2-one (32): white solid; 61% yield; **^{1}H NMR (400 MHz, CDCl$_3$)** δ 7.93 – 7.87 (m, 2H), 7.73 – 7.67 (m, 2H), 7.57 – 7.44 (m, 1H), 7.33 (dt, J = 7.7, 3.7 Hz, 4H), 4.17 – 4.00 (m, 2H), 2.54 (m, J = 10.3, 5.1, 2.5 Hz, 1H), 2.45 (s, 3H), 1.98 (dd, J = 7.9, 5.0 Hz, 1H), 1.36 (t, J = 5.0 Hz, 1H); **^{13}C NMR (100 MHz, CDCl$_3$)** δ 192.38, 169.44, 145.61, 135.59, 134.58, 133.74, 129.93, 129.38, 128.49, 128.28, 47.44, 38.74, 21.82, 21.57, 19.37.

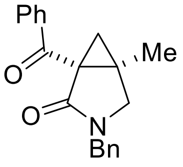

1-Benzoyl-3-benzyl-5-methyl-3-azabicyclo[3.1.0]hexan-2-one (33): colorless oil; 55% yield; **^{1}H NMR (400 MHz, CDCl$_3$)** δ 7.70 (t, J = 6.8 Hz, 2H), 7.59 (q, J = 7.2 Hz, 1H), 7.50 – 7.27 (m, 7H), 4.63 (dd, J = 14.5, 6.1 Hz, 1H), 4.28 (dd, J = 14.5, 6.2 Hz, 1H), 3.46 (qd, J = 10.6, 6.1 Hz, 2H), 2.06 (t, J = 5.4 Hz, 1H), 1.24 (d, J = 6.1 Hz, 3H), 1.19 (t, J = 5.4 Hz, 1H)**; ^{13}C NMR (100 MHz, CDCl$_3$)** δ 194.41, 172.74, 137.64, 136.71, 133.15, 128.99, 128.65, 128.07, 77.48, 77.16, 76.84, 53.04, 46.57, 43.61, 31.21, 23.05, 16.06; **HRMS (ESI)** m/z calcd for $C_{20}H_{19}NNaO_2^+$ $(M+Na)^+$ 328.1308, found 328.1311.

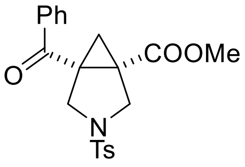

Methyl 5-benzoyl-3-tosyl-3-azabicyclo[3.1.0]hexane-1-carboxylate (34): colorless oil; 83% yield; **^{1}H NMR (400 MHz, CDCl$_3$)** δ 7.78 – 7.66 (m, 4H), 7.57 (t, *J* = 7.4 Hz, 1H), 7.45 – 7.32 (m, 4H), 3.93 – 3.66 (m, 3H), 3.51 (s, 3H), 3.39 (d, *J* = 9.5 Hz, 1H), 2.47 (s, 3H), 2.14 (d, *J* = 5.4 Hz, 1H), 1.72 (d, *J* = 5.3 Hz, 1H); **^{13}C NMR (100 MHz, CDCl$_3$)** δ 194.16, 169.28, 144.38, 135.72, 133.82, 132.67, 130.12, 128.91, 127.79, 52.62, 52.40, 49.86, 43.55, 36.36, 21.72, 20.23.

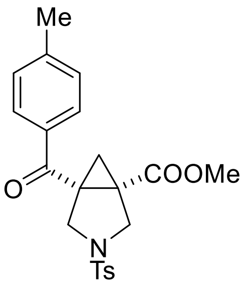

Methyl 5-(4-methylbenzoyl)-3-tosyl-3-azabicyclo[3.1.0]hexane-1-carboxylate (35): colorless oil; 85% yield; **^{1}H NMR (400 MHz, CDCl$_3$)** δ 7.68 (t, *J* = 8.0 Hz, 4H), 7.35 (d, *J* = 8.0 Hz, 2H), 7.21 (d, *J* = 8.0 Hz, 2H), 3.85 (d, *J* = 9.5 Hz, 1H), 3.79 (d, *J* = 9.9 Hz, 1H), 3.70 (d, *J* = 9.9 Hz, 1H), 3.51 (s, 3H), 3.37 (d, *J* = 9.5 Hz, 1H), 2.47 (s, 3H), 2.41 (s, 3H), 2.13 (d, *J* = 5.2 Hz, 1H), 1.71 (d, *J* = 5.3 Hz, 1H); **^{13}C NMR (100 MHz, CDCl$_3$)** δ 193.62, 169.35, 144.92, 144.35, 139.26, 133.18, 132.67, 130.12, 129.62, 129.07, 127.80, 52.71, 52.38, 49.90, 43.64, 36.08, 21.89, 21.73, 20.22.

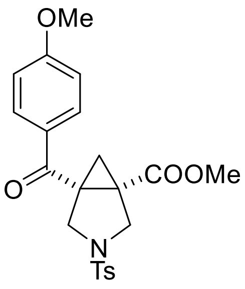

Methyl 5-(4-methoxybenzoyl)-3-tosyl-3-azabicyclo[3.1.0]hexane-1-carboxylate (36): colorless oil; 86% yield; **^{1}H NMR (400 MHz, CDCl$_3$)** δ 7.77 (d, *J* = 8.4 Hz, 2H), 7.68 (d, *J* = 8.0 Hz, 2H), 7.35 (d, *J* = 7.9 Hz, 2H), 6.88 (d, *J* = 8.5 Hz, 2H), 3.86 (s, 3H), 3.84 – 3.75 (m, 2H), 3.70 (d, *J* = 9.9 Hz, 1H), 3.51 (s, 3H), 3.35 (d, *J* = 9.6 Hz, 1H), 2.46 (s, 3H), 2.11 (d, *J* = 5.2 Hz, 1H), 1.70 (d, *J* = 5.2 Hz, 1H); **^{13}C NMR (100 MHz, CDCl$_3$)** δ 192.22, 169.37, 164.14, 144.32, 132.64, 131.35, 130.09, 128.59, 127.77, 114.13, 55.69, 52.77, 52.34, 49.87, 43.61, 35.74, 21.70, 20.21.

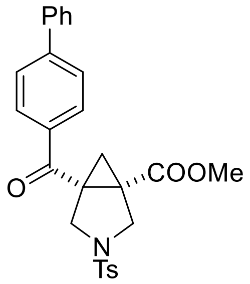

Methyl 5-([1,1'-biphenyl]-4-carbonyl)-3-tosyl-3-azabicyclo[3.1.0]hexane-1-carboxylate (37): pale yellow oil; 82% yield; **^{1}H NMR (400 MHz, $CDCl_3$)** δ 7.84 (d, *J* = 8.0 Hz, 2H), 7.71 (d, *J* = 8.1 Hz, 2H), 7.66 – 7.59 (m, 4H), 7.49 (t, *J* = 7.3 Hz, 2H), 7.43 (d, *J* = 7.1 Hz, 1H), 7.36 (d, *J* = 8.0 Hz, 2H), 3.90 (d, *J* = 9.6 Hz, 1H), 3.82 (d, *J* = 9.9 Hz, 1H), 3.73 (d, *J* = 9.9 Hz, 1H), 3.54 (s, 3H), 3.42 (d, *J* = 9.5 Hz, 1H), 2.46 (s, 3H), 2.18 (d, *J* = 5.2 Hz, 1H), 1.76 (d, *J* = 5.2 Hz, 1H); **^{13}C NMR (100 MHz, $CDCl_3$)** δ 193.68, 169.33, 146.59, 144.39, 139.68, 134.39, 132.70, 130.14, 129.53, 129.19, 128.65, 127.83, 127.55, 127.40, 52.69, 52.46, 49.90, 43.64, 36.30, 21.73, 20.31.

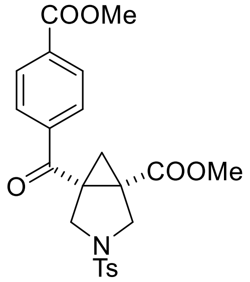

Methyl 5-(4-(methoxycarbonyl)benzoyl)-3-tosyl-3-azabicyclo[3.1.0]hexane-1-carboxylate (38): colorless oil; 77% yield; **^{1}H NMR (400 MHz, $CDCl_3$)** δ 8.05 (d, *J* = 8.1 Hz, 2H), 7.75 (d, *J* = 8.1 Hz, 2H), 7.69 (d, *J* = 8.0 Hz, 2H), 7.36 (d, *J* = 7.9 Hz, 2H), 3.95 (s, 3H), 3.84 (dd, *J* = 14.7, 9.8 Hz, 2H), 3.69 (d, *J* = 10.1 Hz, 1H), 3.51 (s, 3H), 3.39 (d, *J* = 9.6 Hz, 1H), 2.48 (s, 3H), 2.14 (d, *J* = 5.3 Hz, 1H), 1.77 (d, *J* = 5.3 Hz, 1H); **^{13}C NMR (100 MHz, $CDCl_3$)** δ 194.05, 169.07, 166.06, 144.54, 139.02, 134.37, 132.44, 130.17, 130.06, 128.67, 127.81, 52.72, 52.52, 52.44, 49.80, 43.37, 37.05, 21.72, 20.41; **HRMS (ESI)** m/z calcd for $C_{23}H_{23}NNaO_7S^+$ $(M+Na)^+$ 480.1087, found 480.1088.

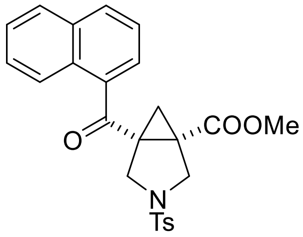

Methyl 5-(1-naphthoyl)-3-tosyl-3-azabicyclo[3.1.0]hexane-1-carboxylate (39): colorless oil; 81% yield; **^{1}H NMR (400 MHz, $CDCl_3$)** δ 8.36 (dd, *J* = 7.7, 2.0 Hz, 1H), 8.00 (d, *J* = 8.2 Hz, 1H), 7.87 (dd, *J* = 7.2, 2.1 Hz, 1H), 7.82 – 7.77 (m, 1H), 7.69 (d, *J* = 8.0 Hz, 2H), 7.54 (ddt, *J* = 10.6, 7.0, 3.5 Hz, 2H), 7.46 (t, *J* = 7.7 Hz, 1H), 7.34 (d, *J* = 8.0 Hz, 2H), 3.93 (d, *J* = 9.4 Hz, 1H), 3.81 (d, *J* = 9.8 Hz, 1H), 3.62 (d, *J* = 9.9 Hz, 1H), 3.53 (d, *J* = 9.6 Hz, 1H), 3.49 (s, 3H), 2.46 (s, 3H), 2.20 (d, *J* = 5.3 Hz, 1H), 1.86 (d, *J* = 5.4 Hz, 1H); **^{13}C NMR (100 MHz, $CDCl_3$)** δ 197.59, 169.17, 144.32, 134.08, 134.01, 133.47, 132.55, 130.43, 130.08, 129.04, 128.63, 128.24, 127.76, 126.79, 125.62, 124.21, 52.66, 52.57, 50.01, 44.81, 38.42, 21.70, 21.25.

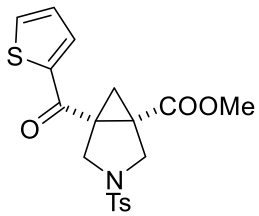

Methyl 5-(thiophene-2-carbonyl)-3-tosyl-3-azabicyclo[3.1.0]hexane-1-carboxylate (40): colorless oil; 75% yield; **^{1}H NMR (400 MHz, $CDCl_3$)** δ 7.72 – 7.65 (m, 3H), 7.58 (dd, *J* = 3.3, 1.8 Hz, 1H), 7.36 (d, *J* = 7.8 Hz, 2H), 7.09 (ddd, *J* = 5.1, 3.8, 1.4 Hz, 1H), 3.88 (dd, *J* = 9.5, 1.3 Hz, 1H), 3.78 (dd, *J* = 9.9, 1.4 Hz, 1H), 3.67 (d, *J* = 9.8 Hz, 1H), 3.52 (s, 3H), 3.42 (d, *J* = 9.5 Hz, 1H), 2.46 (s, 3H), 2.20 (d, *J* = 5.2 Hz, 1H), 1.72 (d, *J* = 5.2 Hz, 1H); **^{13}C NMR (100 MHz, $CDCl_3$)** δ 186.06, 169.13, 144.41, 142.53, 135.08, 133.51, 132.62, 130.13, 128.43, 127.80, 52.72, 52.44, 49.77, 44.12, 35.88, 21.73, 20.24.

5 Gram-scale synthesis

A dry Schlenk tube flushed with argon equipped with a magnetic stirrer bar and a septum was charged with **1a** (5 mmol, 1.0 equiv) and $Pd(OAc)_2$ (0.5 mmol, 10 mol%) in AcOH (40 mL) and MeCN (10 mL). **MPO-1** (9 mmol, 1.8 equiv) was added and the mixture was stirred at 50 ℃ for 5 h. The residue was then purified by flash chromatography on silica gel (PE/EA) to give product **1** (86% yield) as white solid.

6 Sensitivity assessment

Table S8. Parameters of sensitivity assessment.

Entry	Experiment	Description
1	High concentration	0.7 mL solvent
2	Low concentration	1.3 mL solvent
3	High H_2O	30 μL H_2O
4	High O_2	O_2
5	Low temperature	40 °C
6	High temperature	60 °C
7	Low light intensity	Dark
8	High light intensity	Blue LEDs
9	Big scale	**1a**（5 mmol，1.00 g）

Table S9. Results of the sensitivity assessment.

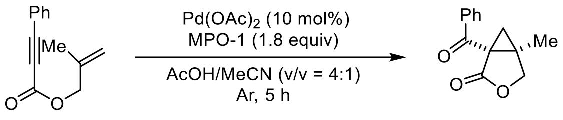

Entry	Parameter	Yield（**1**，%）	Deviation（%）
1	High concentration	90	-1
2	Low concentration	90	-1
3	H_2O	76	-15
4	High O_2	89	-2
5	Low temperature	78	-13
6	High temperature	85	-6
7	Low light intensity	90	-1
8	High light intensity	89	-2
9	Big scale	86	-5

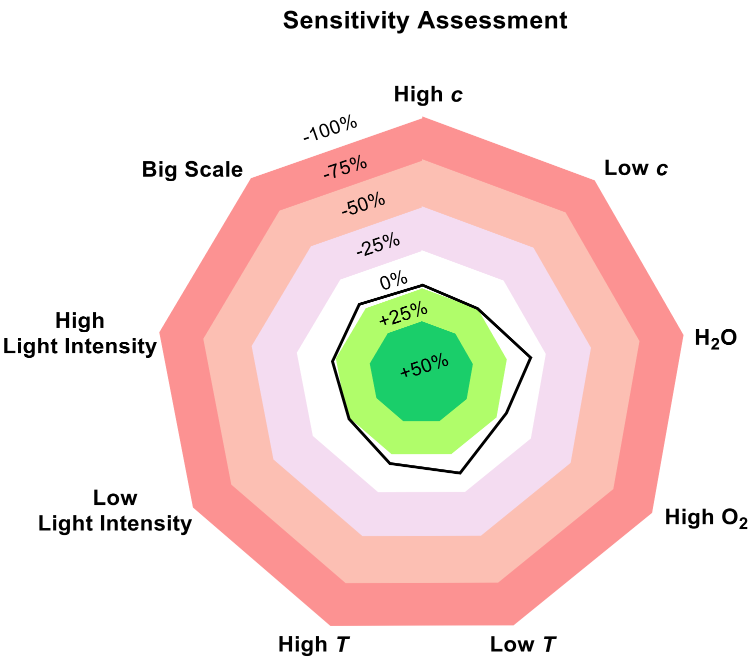

Figure S1. Radar diagram of sensitivity assessment results.

7 Catalytic asymmetric investigation

Table S10. Ligand screening.

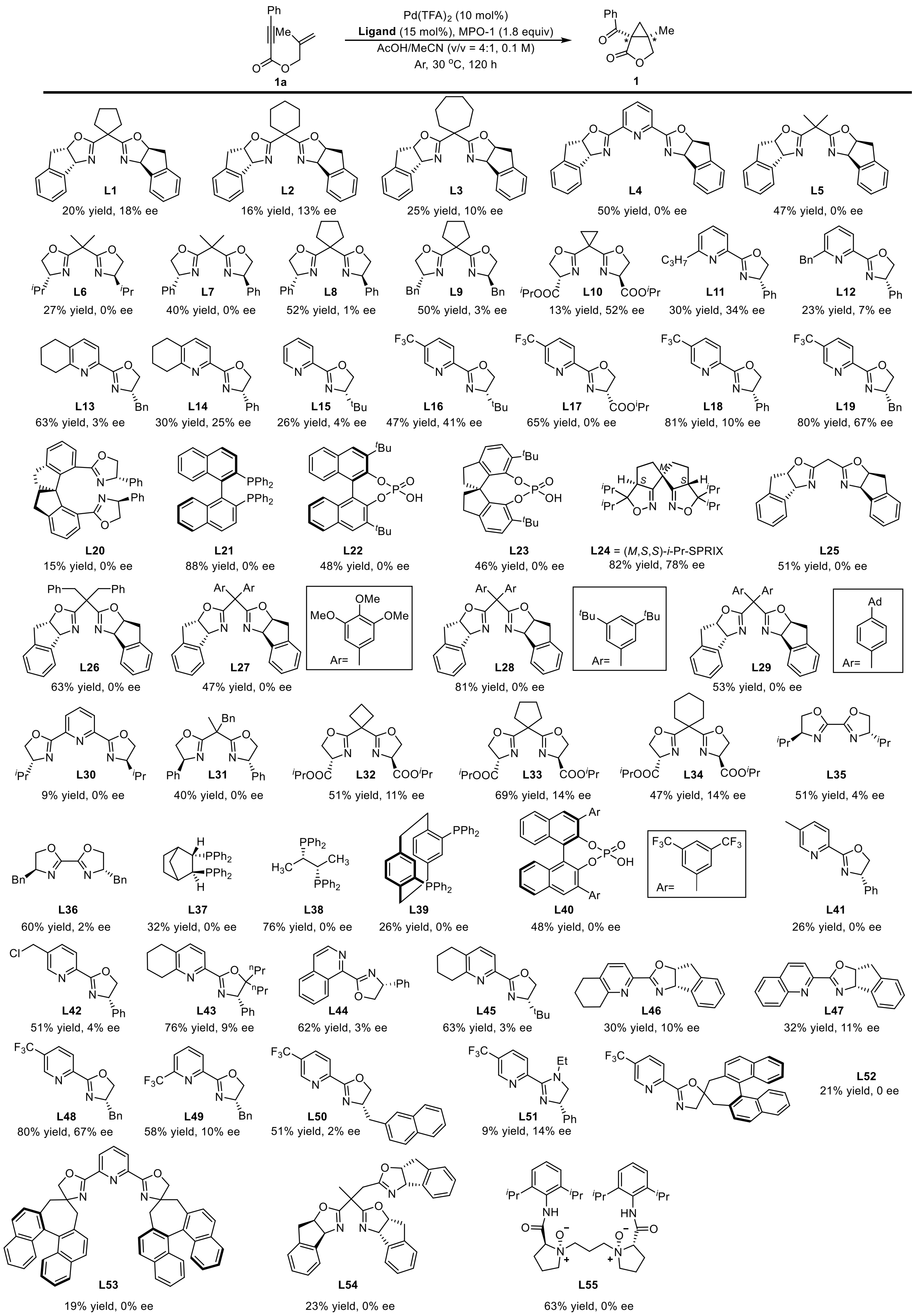

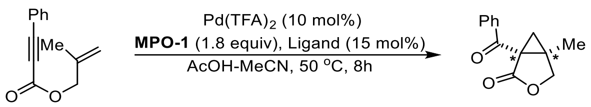

1a → **1**

70% yield, 67% ee

A dry Schlenk tube flushed with argon equipped with a magnetic stirrer bar and a septum was charged with **L19** (0.015 mmol, 15 mol%) and Pd(TFA)$_2$ (0.01 mmol, 10 mol%) in MeCN (0.2 mL), and the mixture was stirred for 1 h at room temperature. Then a solution of **1a** (0.1 mmol, 1.0 equiv) in AcOH (0.8 mL) and **MPO-1** (0.18 mmol, 1.8 equiv) was added, and the reaction mixture was stirred at 50 °C for 8 h. The residue was then purified by flash chromatography on silica gel (PE/EA) to give product **1** (80% yield, 67% ee) as white solid. The enantiomeric excess was determined by HPLC analysis: DAICEL CHIRALPAK AS-H, *n*-hexane/isopropanol = 75/25, λ = 254 nm, 0.5 mL/min.

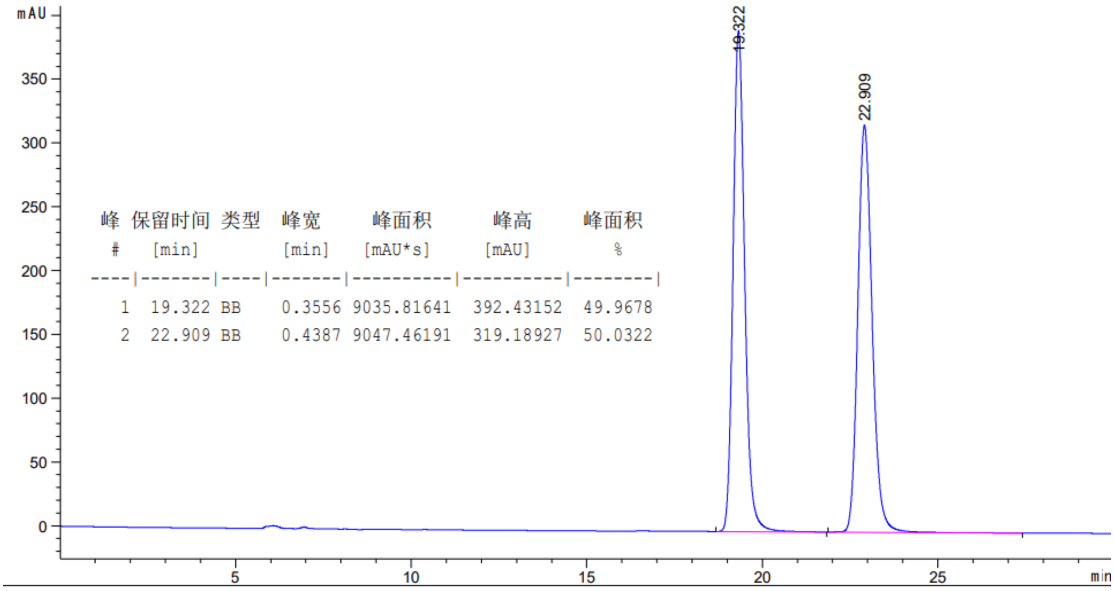

Figure S2. HPLC spectrum of racemic product **1**.

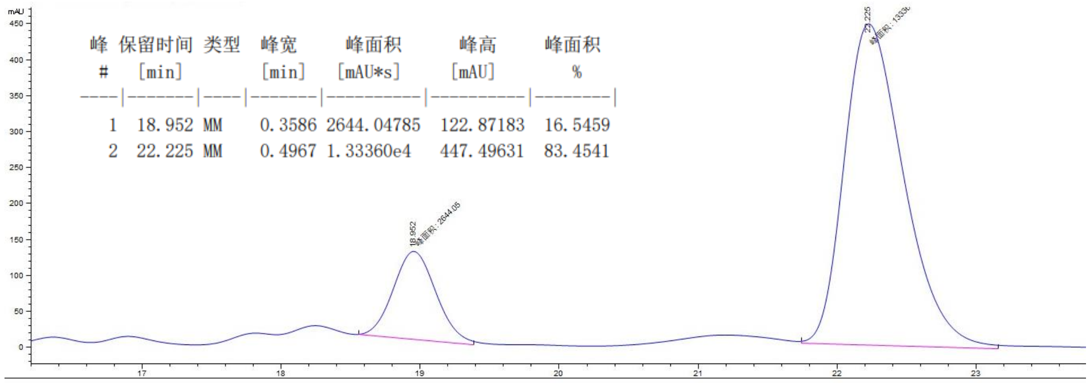

Figure S3. HPLC spectrum of enantioselective product **1** by using **L19** as the ligand.

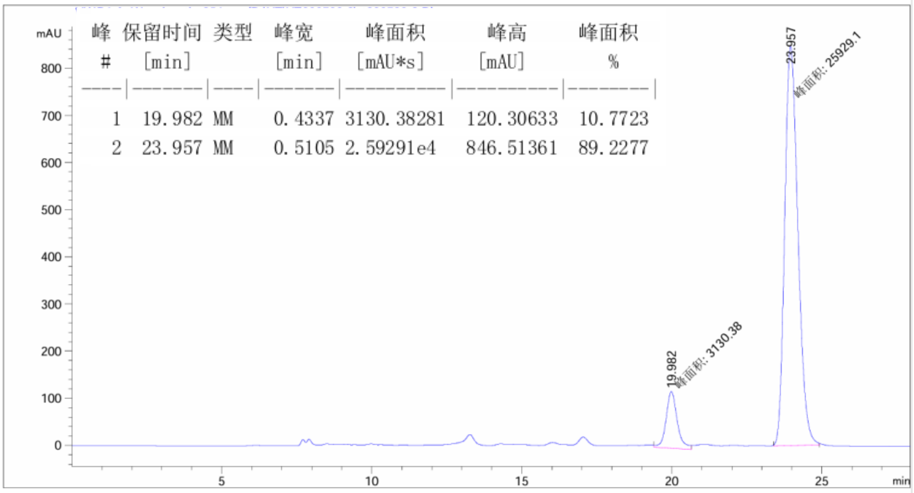

Figure S4. HPLC spectrum of enantioselective product **1** by using **L24** as the ligand.

8 Mechanism experiments

8.1 Effect of radical inhibitors

A dry Schlenk tube flushed with argon equipped with a magnetic stirrer bar and a septum was charged with **1a** (0.1 mmol, 1.0 equiv), $Pd(OAc)_2$ (0.01 mmol, 10 mol%) and additive (0.10 mmol, 1.0 equiv) in AcOH (0.8 mL) and MeCN (0.2 mL). **MPO-1** (0.18 mmol, 1.8 equiv) was added and the mixture was stirred at 50 ℃ for 5 h. The residue was then purified by flash chromatography on silica gel (PE/EA).

Table S11. Radical inhibition experiments.

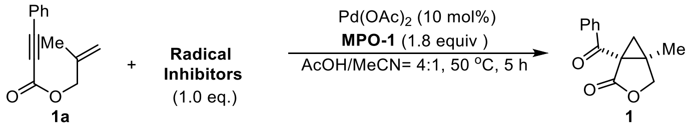

**Radical Inhibitors**	None	TEMPO	BHT	Hydroquinone	1,4-Dinitrobenzene
**Yield(1, %)**	90	71	88	84	83

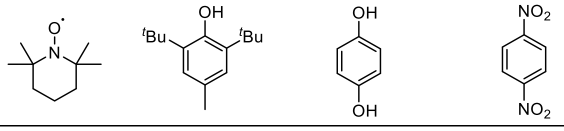

8.2 Control experiments

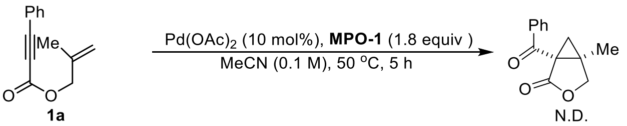

A dry Schlenk tube flushed with argon equipped with a magnetic stirrer bar and a septum was charged with **1a** (0.1 mmol, 1.0 equiv) and $Pd(OAc)_2$ (0.01 mmol, 10 mol%) in MeCN (1 mL). **MPO-1** (0.18 mmol, 1.8 equiv) was added and the mixture was stirred at 50 ℃ for 5 h. The residue was then purified by flash chromatography on silica gel (PE/EA). No desired product was produced.

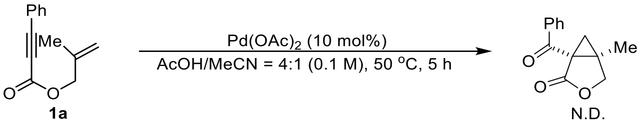

A dry Schlenk tube flushed with argon equipped with a magnetic stirrer bar and a septum was charged with **1a** (0.1 mmol, 1.0 equiv) and $Pd(OAc)_2$ (0.01 mmol, 10 mol%) in AcOH (0.8 mL) and MeCN (0.2 mL). The mixture was stirred at 50 ℃ for 5 h. The residue was then purified by flash chromatography on silica gel (PE/EA). No desired product was produced.

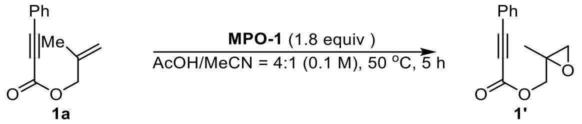

A dry Schlenk tube flushed with argon equipped with a magnetic stirrer bar and a septum was charged with **1a** (0.1 mmol, 1.0 equiv) in AcOH (0.8 mL) and MeCN (0.2 mL). **MPO-1** (0.18 mmol, 1.8 equiv) was added and the mixture was stirred at 50 ℃ for 5 h. The residue was then purified by flash chromatography on silica gel (PE/EA) to give the product as a colorless oil in 49% yield. The product was subjected to ^{1}H NMR and ^{13}C NMR.

(2-methyloxiran-2-yl)methyl 3-phenylpropiolate (1’):

^{1}H NMR (400 MHz, $CDCl_3$) δ 7.66 – 7.56 (m, 2H), 7.51 – 7.43 (m, 1H), 7.38 (ddt, *J* = 8.5, 6.8, 1.2 Hz, 2H), 4.36 (d, *J* = 11.9 Hz, 1H), 4.14 (d, *J* = 11.9 Hz, 1H), 2.84 (d, *J* = 4.7 Hz, 1H), 2.71 (d, *J* = 4.6 Hz, 1H), 1.45 (s, 3H).

^{13}C NMR (101 MHz, $CDCl_3$) δ 153.79, 133.21, 130.96, 128.75, 119.55, 87.37, 80.27, 68.69, 54.68, 52.14, 18.55.

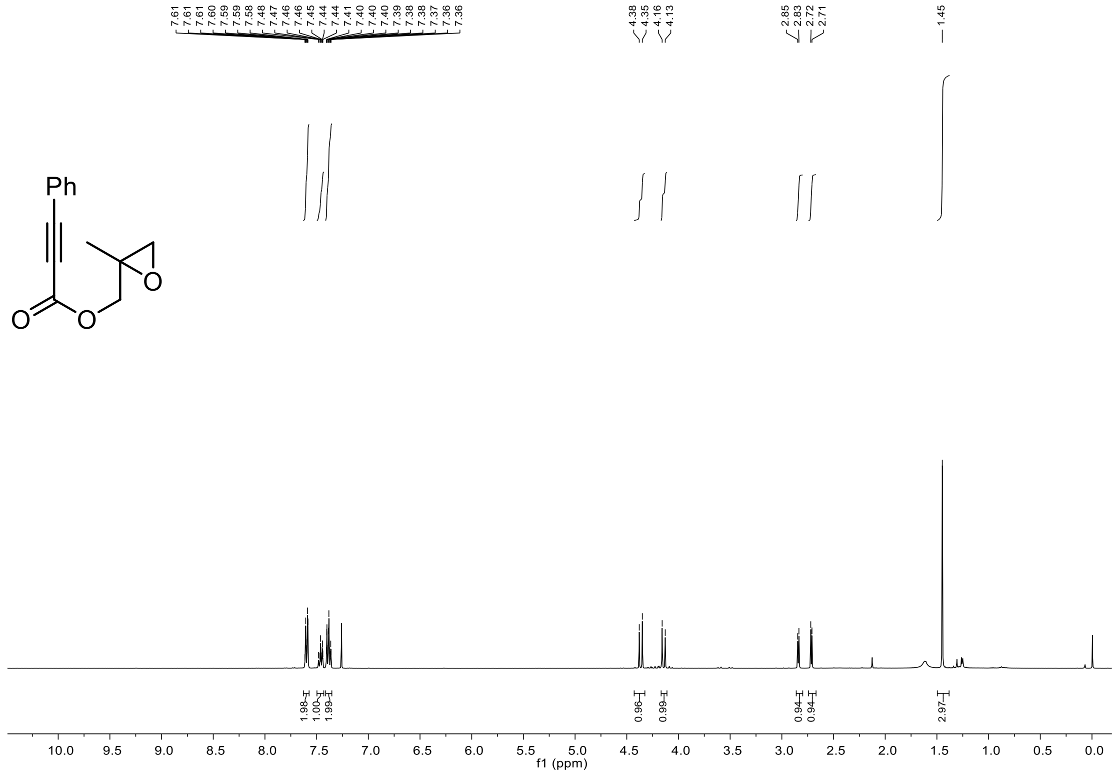
7.61 7.61 7.60 7.59 7.59 7.58 7.48 7.47 7.46 7.45 7.44 7.44 7.41 7.40 7.40 7.39 7.38 7.38 7.37 7.36 7.36
4.38 4.35 4.16 4.13
2.85 2.83 2.72 2.71
1.45
Ph
O
1.98 1.00 1.99
0.96 0.99
0.94 0.94
2.97
10.0 9.5 9.0 8.5 8.0 7.5 7.0 6.5 6.0 5.5 5.0 4.5 4.0 3.5 3.0 2.5 2.0 1.5 1.0 0.5 0.0
f1 (ppm)

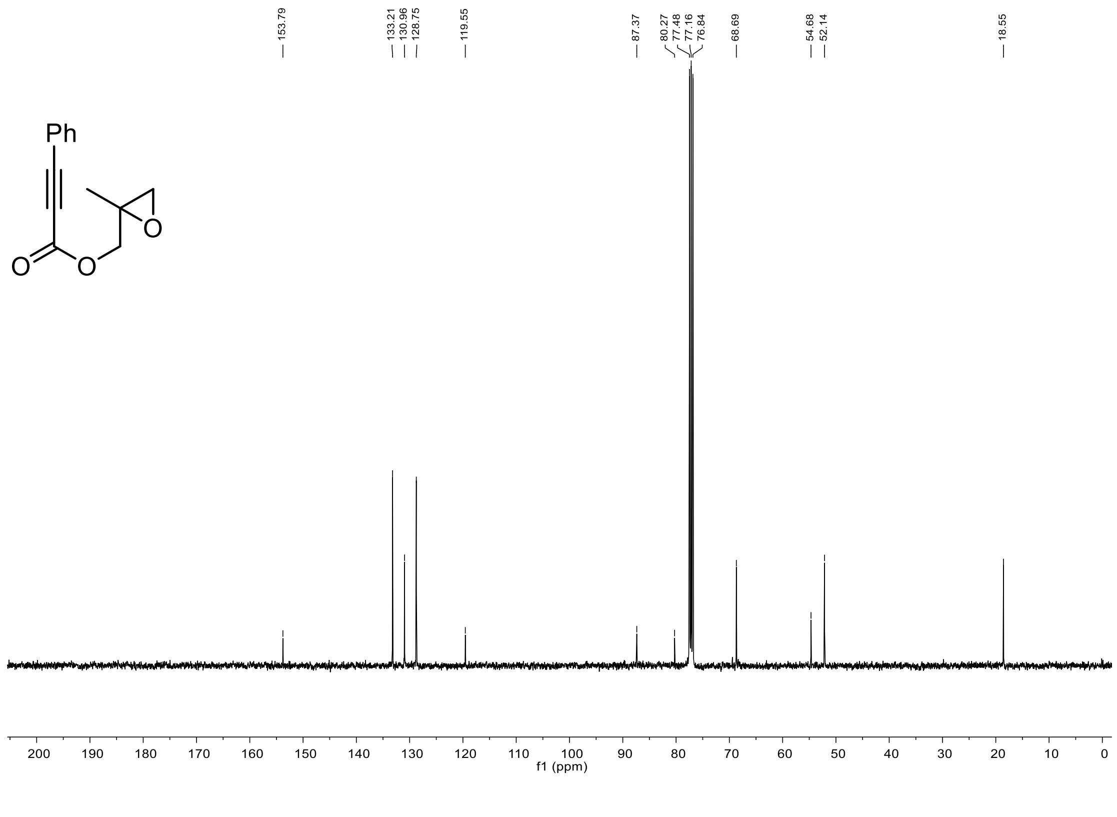
153.79
133.21 130.96 128.75
119.55
87.37
80.27 77.48 77.16 76.84
68.69
54.68 52.14
18.55
Ph
O
200 190 180 170 160 150 140 130 120 110 100 90 80 70 60 50 40 30 20 10 0
f1 (ppm)

8.3 Screening palladium(II) 2,2-dimethylmalonate

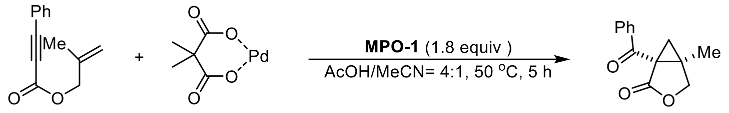

A dry Schlenk tube flushed with argon equipped with a magnetic stirrer bar and a septum was charged with **1a** (0.1 mmol, 1.0 equiv) and palladium(II) 2,2-dimethylmalonate (0.01 mmol, 10 mol%) in AcOH (0.8 mL) and MeCN (0.2 mL). **MPO-1** (0.18 mmol, 1.8 equiv) was added and the mixture was stirred at 50 ℃ for 5 h. The residue was then purified by flash chromatography on silica gel (PE/EA).

8.4 Side product analysis

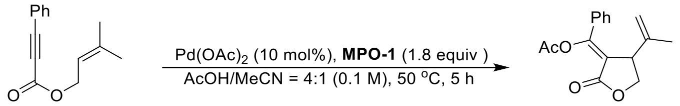

A dry Schlenk tube flushed with argon equipped with a magnetic stirrer bar and a septum was charged with **1a** (0.1 mmol, 1.0 equiv) and $Pd(OAc)_2$ (0.01 mmol, 10 mol%) in AcOH (0.8 mL) and MeCN (0.2 mL). **MPO-1** (0.18 mmol, 1.8 equiv) was added and the mixture was stirred at 50 ℃ for 5 h. The residue was then purified by flash chromatography on silica gel (PE/EA).

(Z)-(2-oxo-4-(prop-1-en-2-yl)dihydrofuran-3(2*H*)-ylidene)(phenyl)methyl acetate (41'): 32% yield; **^{1}H NMR (400 MHz, $CDCl_3$)** δ 7.55 (dd, *J* = 7.8, 1.8 Hz, 2H), 7.47 – 7.35 (m, 3H), 4.93 (d, *J* = 11.8 Hz, 2H), 4.47 (t, *J* = 9.0 Hz, 1H), 4.11 (dd, *J* = 9.2, 4.4 Hz, 1H), 4.00 (dd, *J* = 8.8, 4.4 Hz, 1H), 2.13 (s, 3H), 1.78 (s, 3H).

^{13}C NMR (101 MHz, $CDCl_3$) δ 168.45, 167.23, 158.55, 143.18, 132.36, 130.73, 129.20, 128.04, 116.82, 114.16, 68.98, 47.27, 20.63, 18.05.

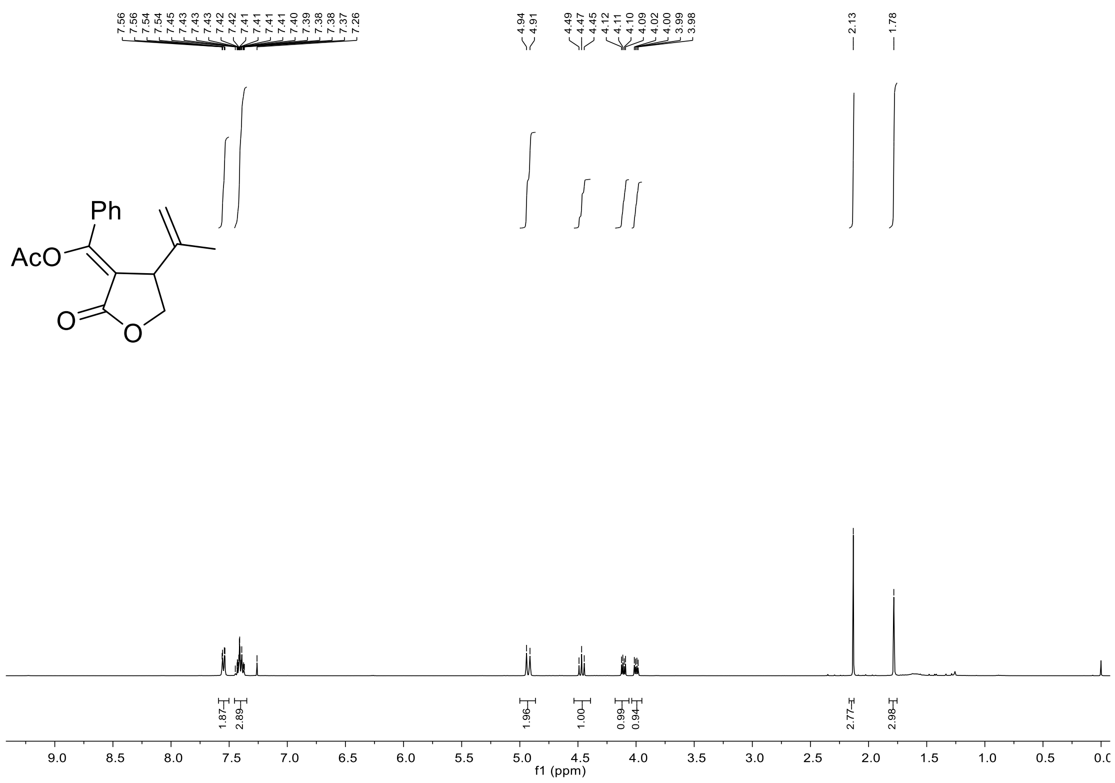

7.56
7.56
7.54
7.45
7.43
7.43
7.43
7.42
7.42
7.41
7.41
7.41
7.40
7.39
7.38
7.38
7.37
7.26
4.94
4.91
4.49
4.47
4.45
4.12
4.11
4.10
4.09
4.02
4.00
3.99
3.98
2.13
1.78
Ph
AcO
O
O
1.87
2.89
1.96
1.00
0.99
0.94
2.77
2.98
f1 (ppm)

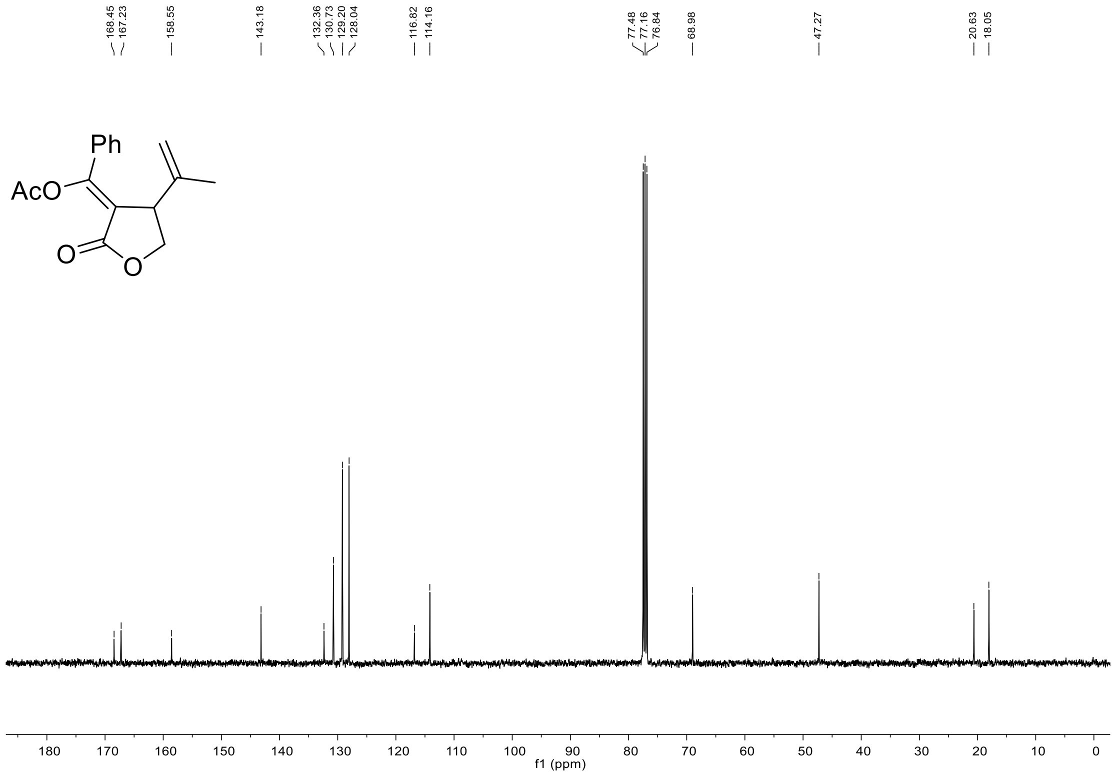

168.45
167.23
158.55
143.18
132.36
130.73
129.20
128.04
116.82
114.16
77.48
77.16
76.84
68.98
47.27
20.63
18.05
Ph
AcO
O
O
f1 (ppm)

8.5 Kinetic experiments

Reaction rate profile: A dry Schlenk tube flushed with argon equipped with a magnetic stirrer bar was charged with **1a** (0.10 mmol, 1.0 equiv) and $Pd(OAc)_2$ (0.01 mmol, 10 mol%) in MeCN (0.2 mL) and AcOH (0.8 mL). **MPO-1** (0.18 mmol, 1.8 equiv) was added, and the reaction mixture was stirred at 50 °C. Aiquots (50 μl) were taken out by syringe at every 30 minutes and immediately quenched by $Na_2S_2O_3$, diluted with EA (1 mL). Product yield was tested by LC using anthracene as an internal standard and the results were presented in Figure S3.

Initial rate profile: A dry Schlenk tube flushed with argon equipped with a magnetic stirrer bar was charged with **1a** (0.10 mmol, 1.0 equiv) and $Pd(OAc)_2$ (0.01 mmol, 10 mol%) in MeCN (1 mL) and AcOH (0.8 mL). **MPO-1** (0.18 mmol, 1.8 equiv) was added, and the reaction mixture was stirred at 50 °C. Aiquots (50 μL) were taken out by syringe at every 5 minutes and immediately quenched by $Na_2S_2O_3$, diluted with EA (1 mL). Product yield was tested by LC using anthracene as an internal standard and the results were presented in Figure S3.

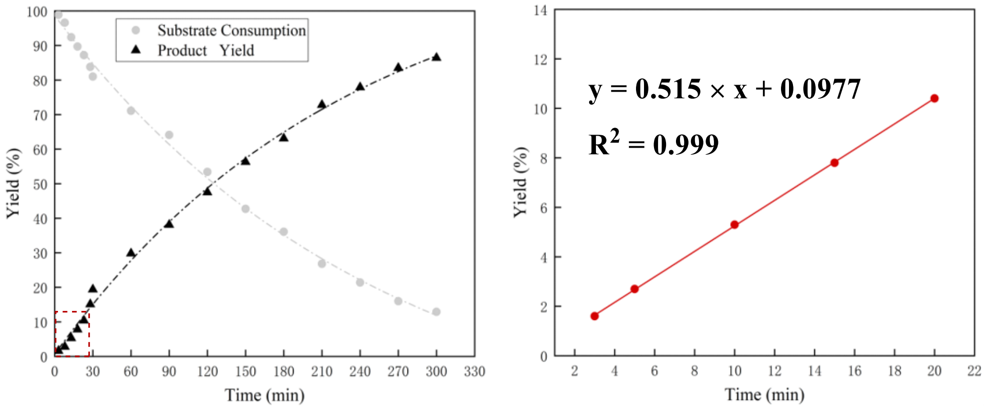

Figure S5. Reaction rate and initial rate profiles.

Order in catalyst: A dry Schlenk tube flushed with argon equipped with a magnetic stirrer bar was charged with **1a** (0.10 mmol, 1.0 equiv) and $Pd(OAc)_2$ (0. 004 ~ 0.012 mmol) in MeCN (1 mL) and AcOH (0.8 mL). **MPO-1** (0.18 mmol, 1.8 equiv) was added, and the reaction mixture was stirred at 50 °C. Aiquots (50 μL) were taken out by syringe at every 5 minutes and immediately quenched by $Na_2S_2O_3$, diluted with EA (1 mL). Product yield from the corresponding reaction was tested by LC using anthracene as an internal standard, and each reported initial rate represents an average of three experiments. The results were presented in Figure S4. A plot of initial rate versus $[Pd(OAc)_2]$ gave a straight line (R^2 = 0.994), indicating a 1st order dependence on $[Pd(OAc)_2]$. Y = 0.04125 mM/min × X + 0.1044.

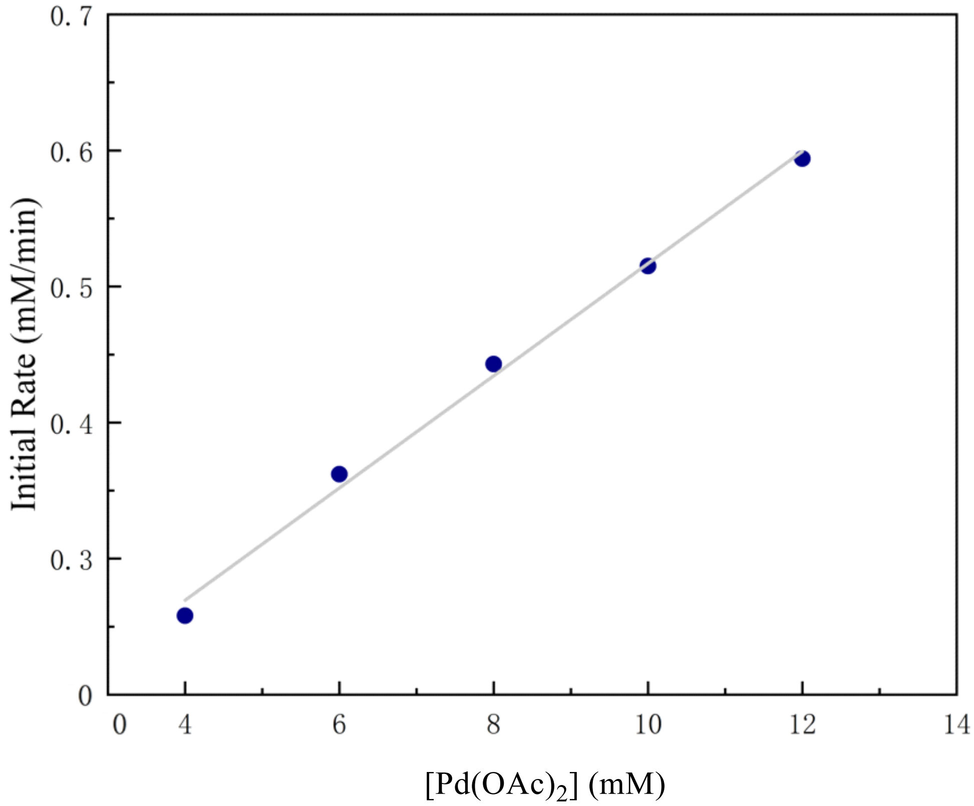

Figure S6. Dependence of the initial rate on $Pd(OAc)_2$

Order in substrate: A dry Schlenk tube flushed with argon equipped with a magnetic stirrer bar was charged with **1a** (0.07 ~ 0.11 mmol) and $Pd(OAc)_2$ (0.01 mmol, 10 mol%) in MeCN (1 mL) and AcOH (0.8 mL). **MPO-1** (0.18 mmol, 1.8 equiv) was added, and the reaction mixture was stirred at 50 °C. Aiquots (1 mL) were taken out by syringe at every 5 minutes and immediately quenched by $Na_2S_2O_3$ (0.5 mL), diluted with EA (1 mL). Product yield from the corresponding reaction was tested by LC using anthracene as an internal standard, and each reported initial rate represents an average of three experiments. The results were presented in Figure S5. A plot of initial rate versus substrate gave a flat line, indicating of a zero order dependence on [substrate]. Y = 0.00009 mM/min × X + 0.5043.

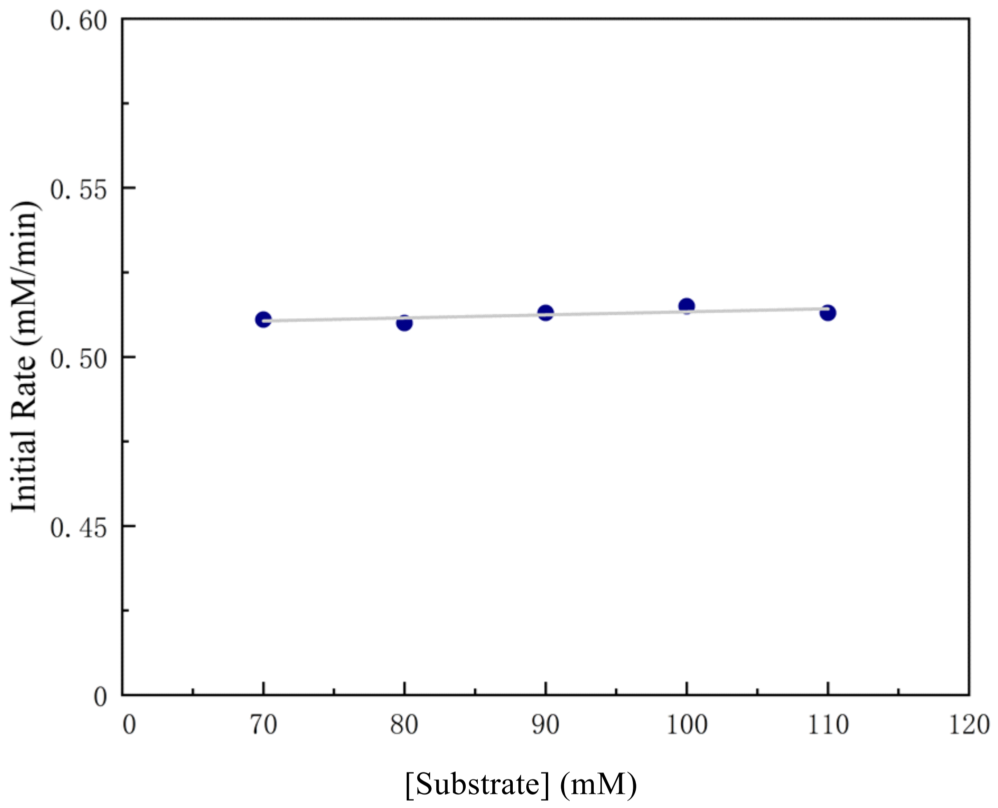

Figure S7. Dependence of the initial rate on substrate

Order in oxidant: A dry Schlenk tube flushed with argon equipped with a magnetic stirrer bar was charged with **1a** (0.10 mmol, 1.0 equiv) and $Pd(OAc)_2$ (0.01 mmol, 10 mol%) in MeCN (1 mL) and AcOH (0.8 mL). **MPO-1** (0.12 ~ 0.20 mmol) was added, and the reaction mixture was stirred at 50 ^{o}C. Aiquots (50 μL) were taken out by syringe at every 5 minutes and immediately quenched by $Na_2S_2O_3$, diluted with EA (1 mL). Product yield from the corresponding reaction was tested by LC using anthracene as an internal standard, and each reported initial rate represents an average of three experiments. The results were presented in Figure S6. A plot of initial rate versus substrate gave straight line (R^2 = 0.996) a flat line, indicating of a 1st order dependence on [MPO]. Y = 0.00387 mM/min × X - 0.1816.

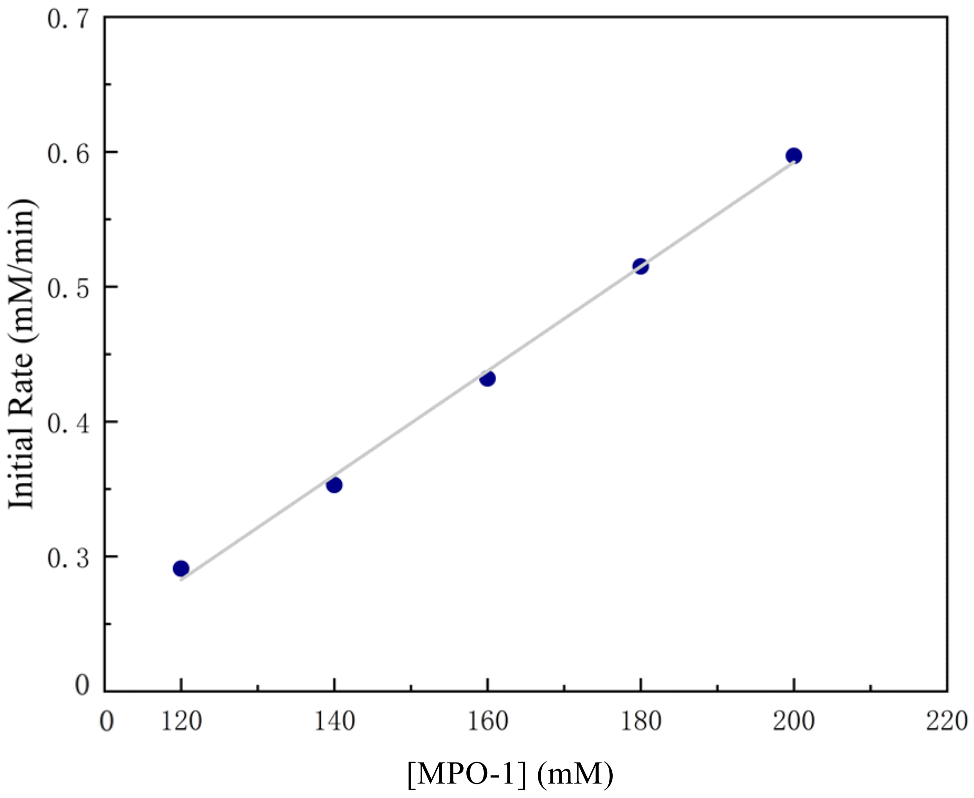

Figure S8. Dependence of the initial rate on oxidant

9 Biological compatibility experiments

9.1 The stability of nucleic acid in the oxidation

100 μM of **1a**, 10 μM of $Pd(OAc)_2$, 180 μM of **MPO-1** and 20 μL pUC 19 were oscillation in 40 μL 1×PBS-AcOH (9:1) at 30 ℃ for 5 h. The agarose gel electrophoresis was conducted to confirm the pUC 19 was not degraded.

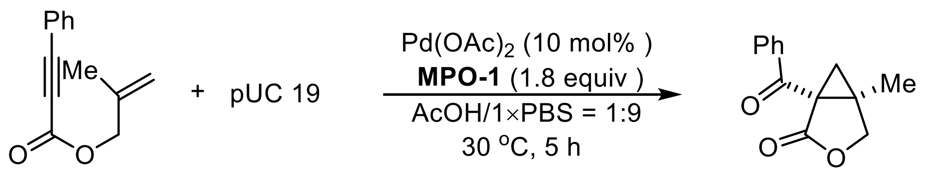

1a **1**

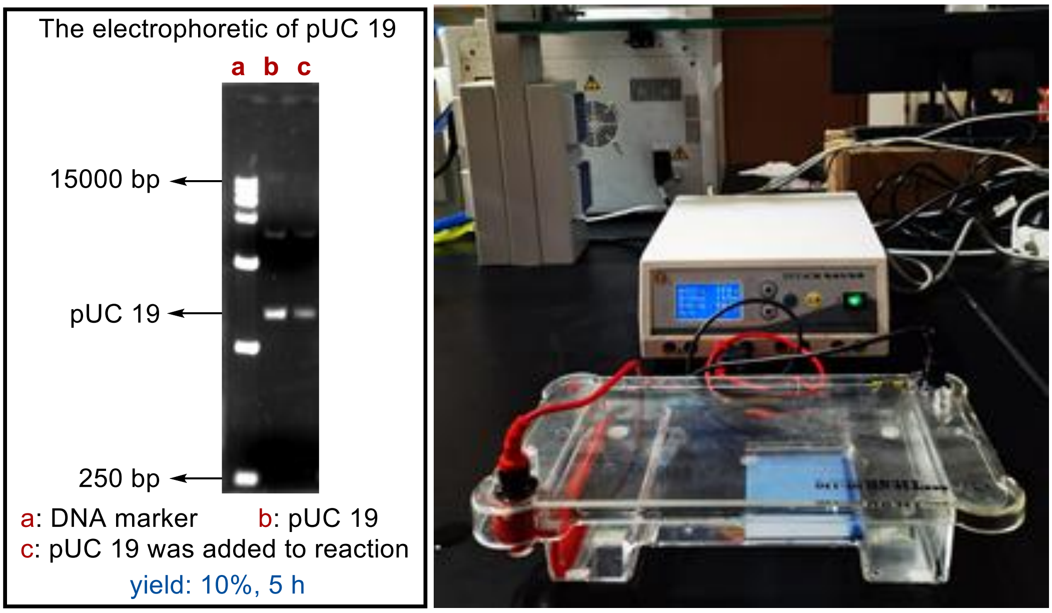

Figure S9. The electrophoretic of pUC 19.

9.2 The activity of DNase I in the reaction

100 μM of **1a**, 10 μM of $Pd(OAc)_2$, 180 μM of **MPO-1** and 0.032 μM DNase I were oscillation in 1×PBS-AcOH (9:1) at 30 ℃ for 5 h. Then, 10 μL the reaction mixture was added 10 μL pUC 19, incubated at 37 ℃ for 1 h, then examined by agarose gel electrophoresis.

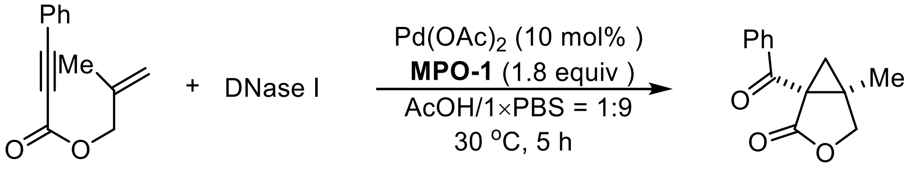

1a **1**

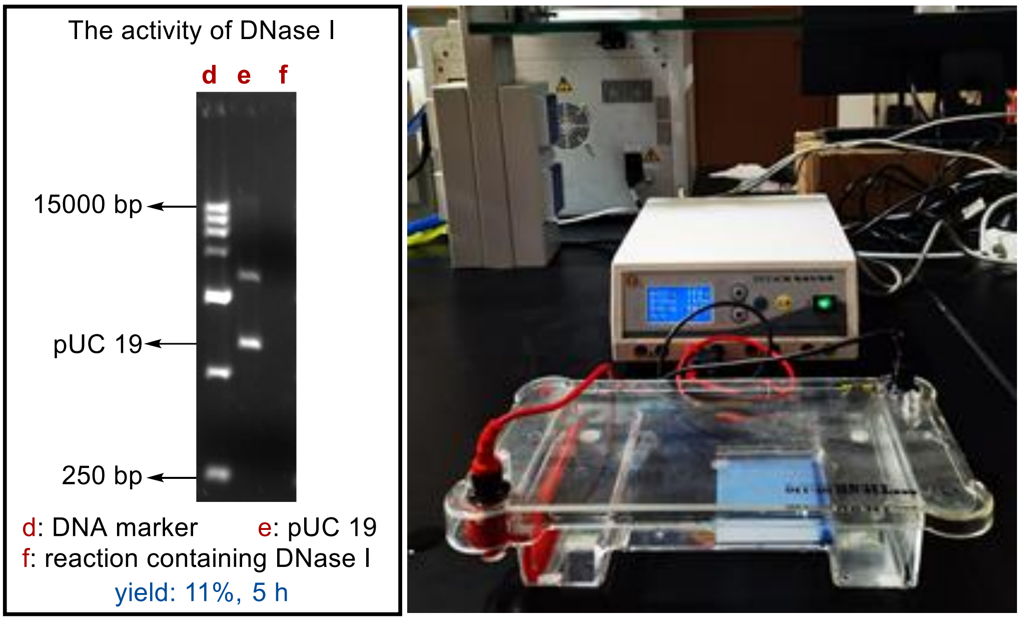

Figure S10. The activity of DNase I.

10. Green chemistry metrics analysis

Table S12. *E*-Factor, AE, RME, PMI, CE and Atom Efficiency for Bicyclo[3.1.0]hexanes Synthesis (This work).

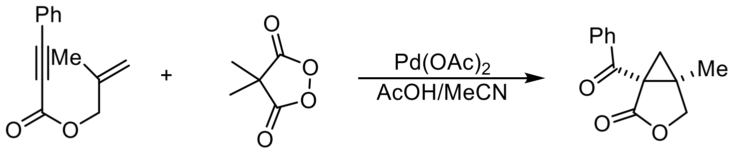

**1a**	**MPO-1** (1.8 equiv)	**1**
200.24	130.10	216.24
20.0 mg	23.4 mg	19.7 mg (91% yield)

Total amount of reactants: 20.0 mg + 23.4 mg = 43.4 mg
Amount of final product:19.7 mg
Amount of waste: 43.4 mg - 19.7 mg = 23.7 mg
E-Factor = Amount of waste/Amount of final product = 23.7/19.7 = 1.20

Molecular weight of product: 216.24
Sum of molecular weight of reagent: 200.24 + 130.10 ×1.8 = 434.42
Atom economy = Molecular weight of product/Sum of molecular weight of reagent = 216.24/434.42 = 50.0%

Mass of product: 19.7 mg
Total Mass of reagent: 20.0 mg + 23.4 mg = 43.4 mg
RME = Mass of product/Total Mass of reagent = 19.7/43.4 = 45.4%

Total Mass in process: 20.0 mg + 23.4 mg = 43.4 mg
Mass of product: 19.7 mg
PMI = Total Mass in process/Mass of product = 43.4/19.7 = 2.20

Amount of carbon in desired product: 13
Total amount of carbon presenten in all reactants: 13 + 5 ×1.8 = 22

$$\text{Carbon Efficiency (\%)} = \frac{\text{Amount of carbon in desired product}}{\text{Total amount of carbon presenten in all reactants}} \times 100\% = 59.1\%$$

Yield of product: 91%
Atom economy: 50.0%
Atom Efficiency (%) = (% Yield of product ×% Atom economy) $\times 100\% = 45.5\%$

Table S13. *E*-Factor, AE, RME, PMI, CE and Atom Efficiency for Bicyclo[3.1.0]hexanes Synthesis Using $PhI(OAc)_2$ as Oxidant (reported by Sanford).

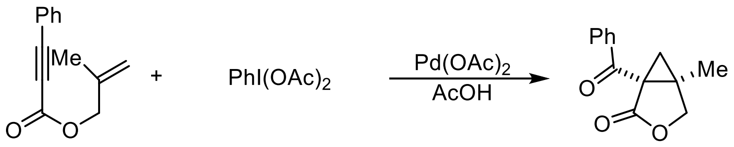

1a 1.1 equiv **1**

200.24	322.10	216.24
20.0 mg	64.4 mg	16.9 mg (78% yield)

Total amount of reactants: 20.0 mg + 64.4 mg = 84.4 mg
Amount of final product: 16.9 mg
Amount of waste: 84.4 mg - 16.9 mg = 67.5 mg
E-Factor = Amount of waste/Amount of final product = 67.5/16.9 = 4.00

Molecular weight of product: 216.24
Sum of molecular weight of reagent: 200.24 + 322.10 ×1.1 = 554.55
Atom economy = Molecular weight of product/Sum of molecular weight of reagent = 216.24/554.55 = 39.0%

Mass of product: 16.9 mg
Total Mass of reagent: 20.0 mg + 64.4 mg = 84.4 mg
RME = Mass of product/Total Mass of reagent = 16.9/84.4 = 20.0%

Total Mass in process: 20.0 mg +64.4 mg = 84.4 mg
Mass of product: 16.9 mg
PMI = Total Mass in process/Mass of product = 84.4/16.9 = 5.00

Amount of carbon in desired product: 13
Total amount of carbon presenten in all reactants: 13 + 10 ×1.1 = 24

$$\text{Carbon Efficiency (\%)} = \frac{\text{Amount of carbon in desired product}}{\text{Total amount of carbon presenten in all reactants}} \times 100\% = 54.2\%$$

Yield of product: 78%
Atom economy: 39.0%
Atom Efficiency (%) = (% Yield of product ×% Atom economy) ×100% = 30.4%

Table S14. *E*-Factor, AE, RME, PMI, CE and Atom Efficiency for Bicyclo[3.1.0]hexanes Synthesis Using $PhI(OAc)_2$ as Oxidant (reported by Sasai).

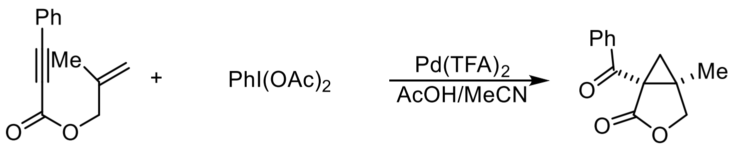

1a	2.0 equiv	1
200.24	322.10	216.24
20.0 mg	64.4 mg	20.8 mg (96% yield)

Total amount of reactants: 20.0 mg + 64.4 mg = 84.4 mg
Amount of final product:20.8 mg
Amount of waste: 84.4 mg - 20.8 mg = 63.6 mg
E-Factor = Amount of waste/Amount of final product = 63.6/20.8 = 3.06

Molecular weight of product: 216.24
Sum of molecular weight of reagent: 200.24 + 322.10 ×2.0 = 844.44
Atom economy = Molecular weight of product/Sum of molecular weight of reagent = 216.24/844.44 = 25.6%

Mass of product: 20.8 mg
Total Mass of reagent: 20.0 mg + 64.4 mg = 84.4 mg
RME = Mass of product/Total Mass of reagent = 20.8/84.4 = 24.7%

Total Mass in process: 20.0 mg +64.4 mg = 84.4 mg
Mass of product: 20.8 mg
PMI = Total Mass in process/Mass of product = 84.4/20.8 = 4.06

Amount of carbon in desired product: 13
Total amount of carbon presenten in all reactants: 13 + 10 ×2.0 = 33

$$\text{Carbon Efficiency (\%)} = \frac{\text{Amount of carbon in desired product}}{\text{Total amount of carbon presenten in all reactants}} \times 100\% = 39.4\%$$

Yield of product: 96%
Atom economy: 25.6%
Atom Efficiency (%) = (% Yield of product ×% Atom economy) ×100% = 25.6%

11 Reference

[1] K. Park, Y. Heo, S. Lee, Metal-free decarboxylative three-component coupling reaction for the synthesis of propargylamines, *Org. Let.* **2013**, *15*, 3322-3325.

[2] T. Tsujihara, K. Takenaka, K. Onitsuka, M. Hatanaka, H. Sasai, Pd^{II}/Pd^{IV} Catalytic enantioselective synthesis of bicyclo[3.1.0]hexanes *via* oxidative cyclization of enynes, *J. Am. Chem. Soc.* **2009**, *131*, 3452–3453.

[3] J. Xuan, C.-G. Daniliuc, A. Studer, Construction of polycyclic *γ*-lactams and related heterocycles *via* electron catalysis, *Org. Lett.* **2016**, *18*, 6372–6375.

[4] M. Chen, X. Zhao, C. Yang, Y. Wang, W. Xia, Further insight into the photochemical behavior of 3-aryl-*N*-(arylsulfonyl)propiolamides: tunable synthetic route to phenanthrenes, *RSC Adv.* **2017**, *7*, 12022–12026.

[5] U. S. Badu, M. K. R. Singam, M. N. Kumar, J. B. Nanubolu, M. S. Reddy, Palladium-catalyzed carbo-aminative cyclization of 1, 6-enynes: access to napthyridinone derivatives, *Org. Lett.* **2022**, *24*, 1598–1603.

[6] J. Xuan, C. G. Daniliuc, A. Studer, Construction of polycyclic *γ*-lactams and related heterocycles *via* electron catalysis, *Org. Lett.* **2016**, *18*, 6372–6375.

[7] Z. Li, J. Zheng, W. Hu, J. Li, W. Wu, H. Jiang, Synthesis of 1, 4-enyne-3-ones *via* palladium-catalyzed sequential decarboxylation and carbonylation of allyl alkynoates, *Org. Chem. Front.* **2017**, *4*, 1363–1366.

[8] S.-F. Pi, B.-X. Tang, J.-H. Li, Y.-L. Liu, Y. Liang, Palladium-catalyzed decarboxylative coupling of allylic alkynoates with arynes, *Org. Lett.* **2009**, *11*, 2309–2312.

12 Copies of NMR spectra

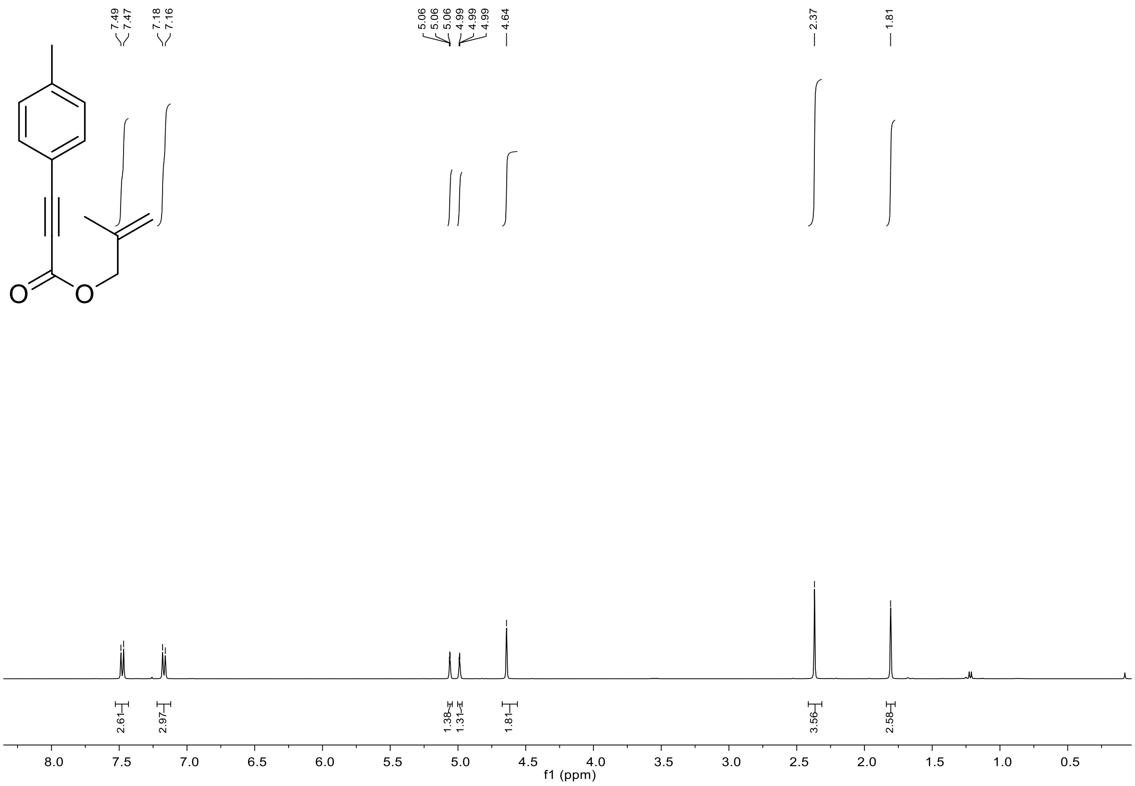

^{1}H NMR spectrum of **2a**

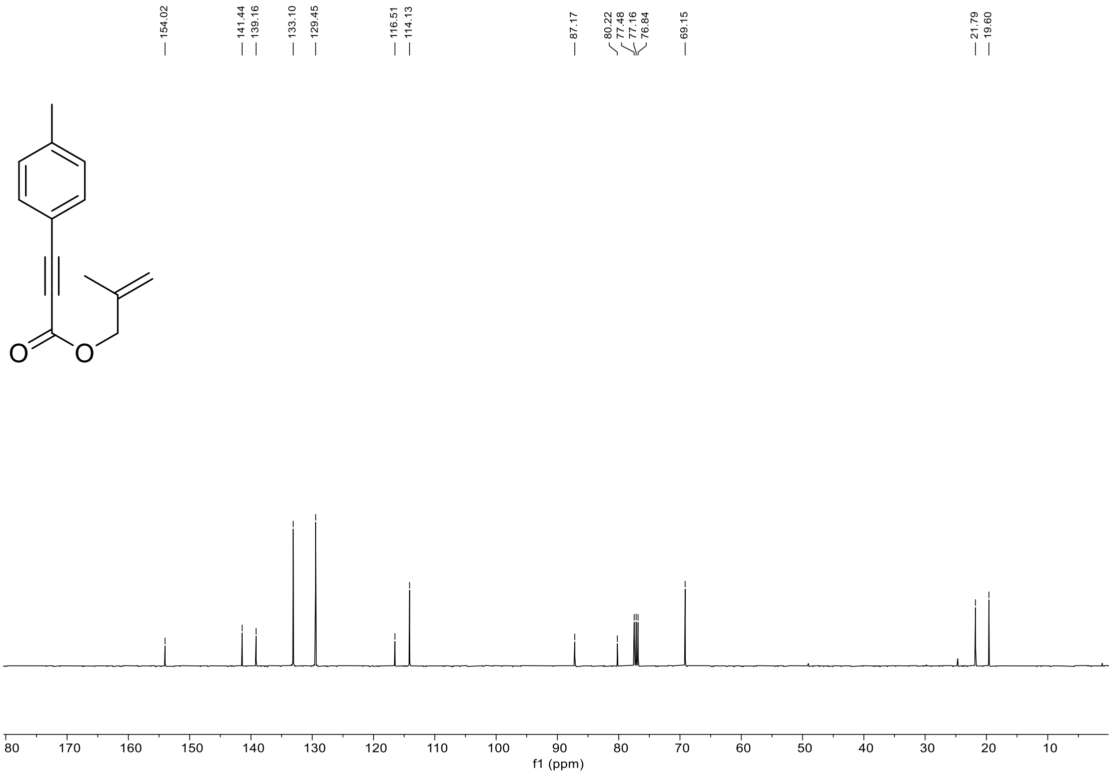

^{13}C NMR spectrum of **2a**

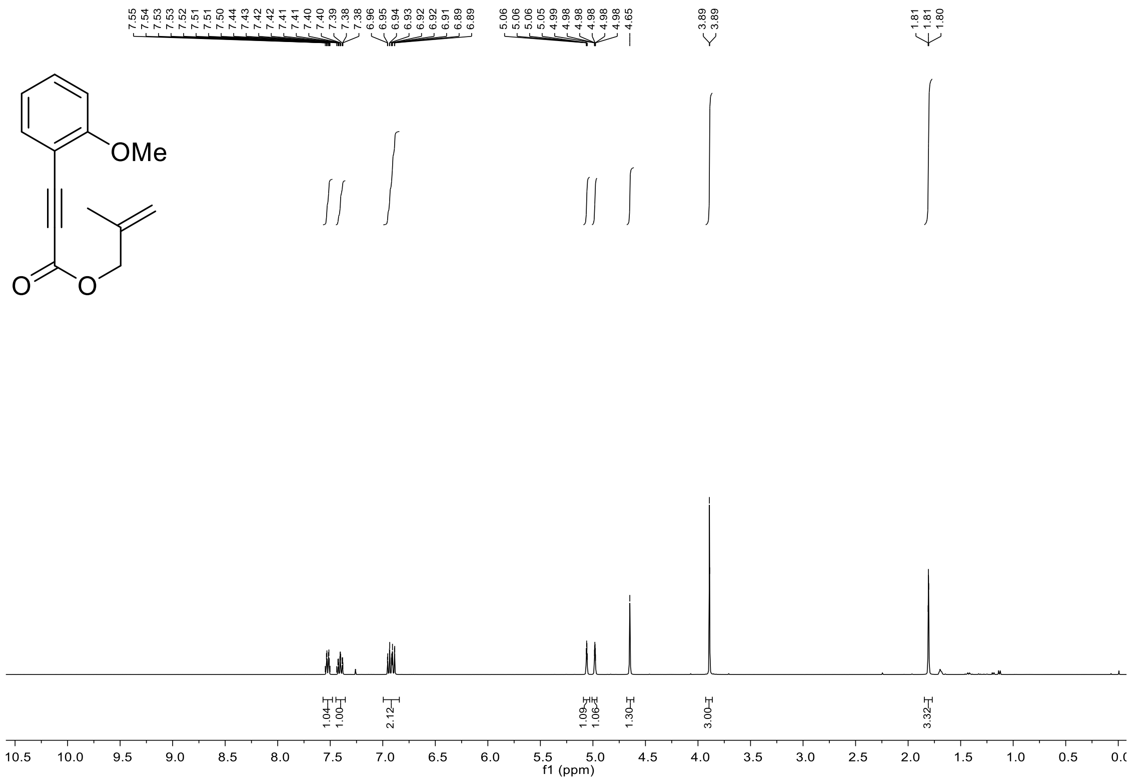

^{1}H NMR spectrum of **5a**

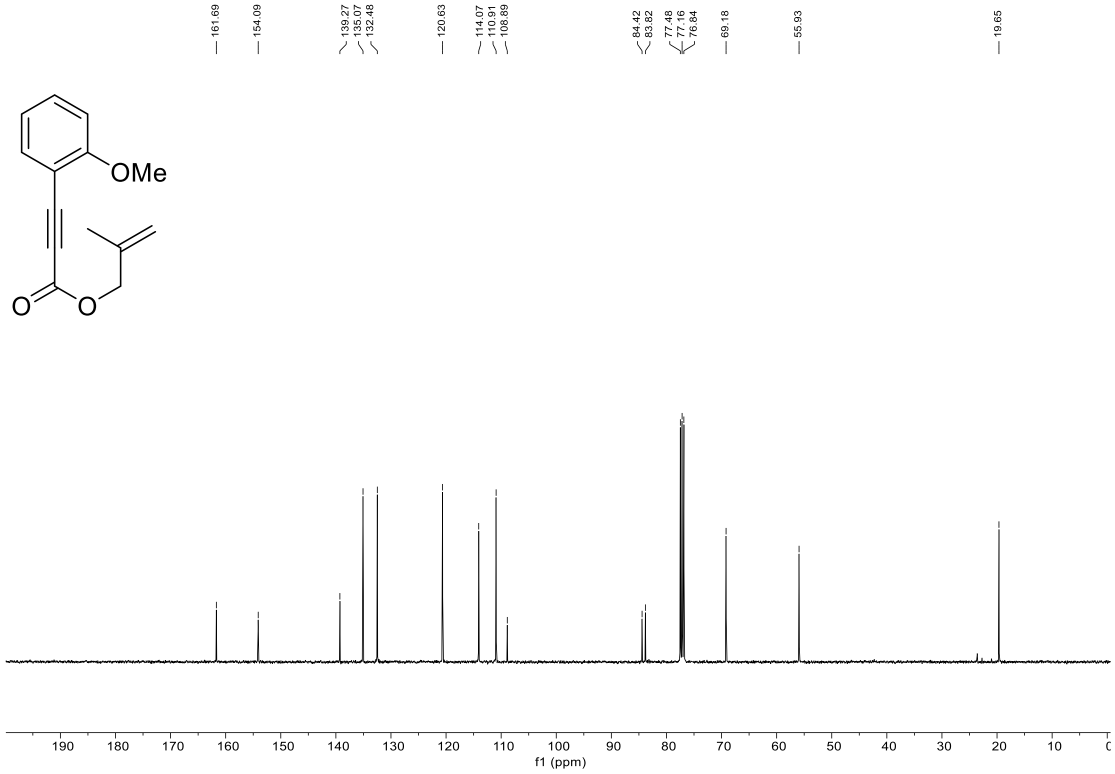

^{13}C NMR spectrum of **5a**

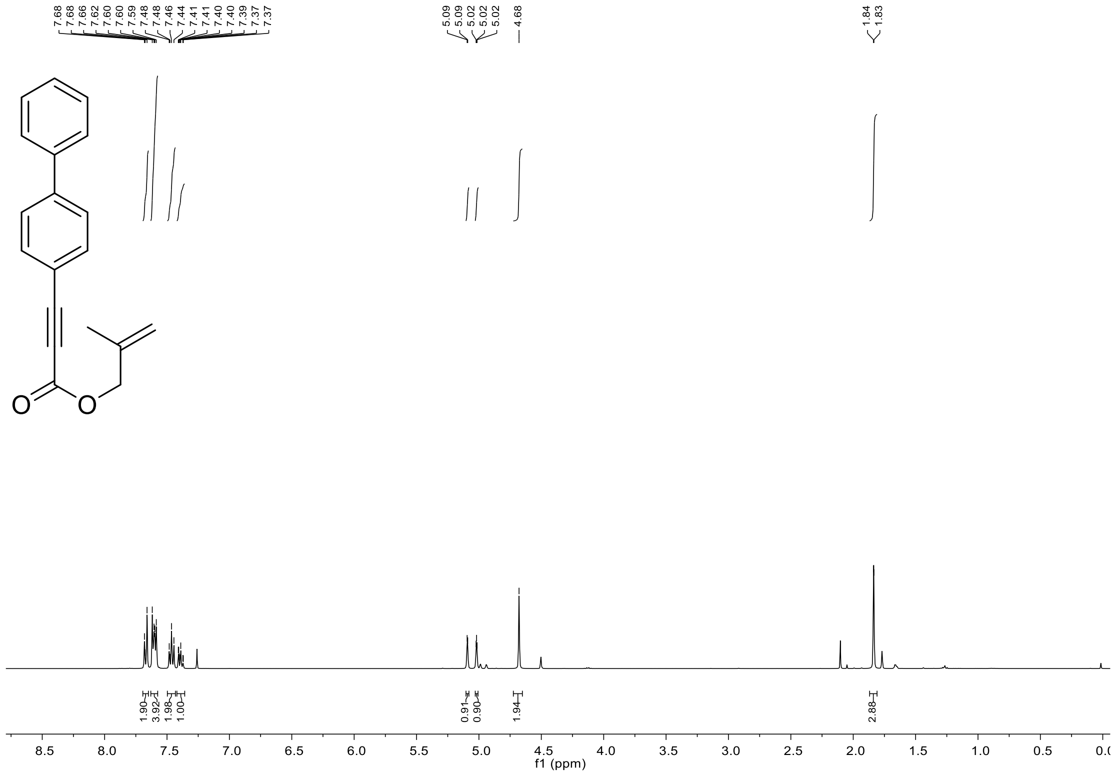

^{1}H NMR spectrum of **6a**

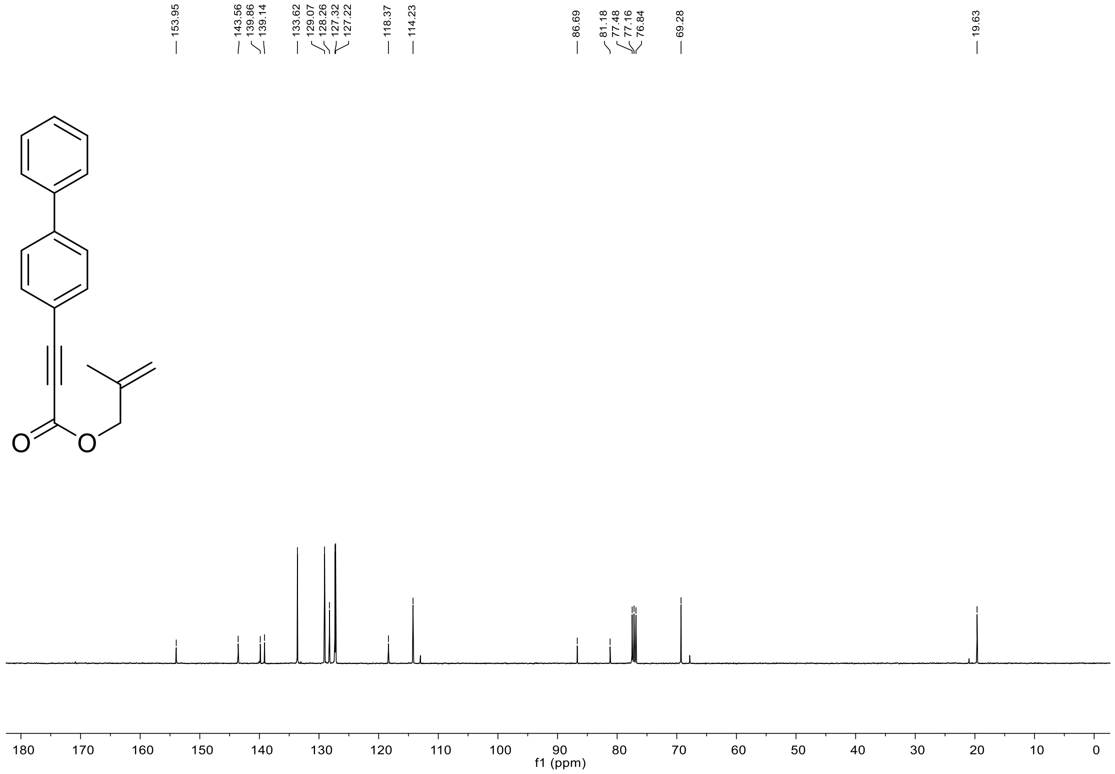

^{13}C NMR spectrum of **6a**

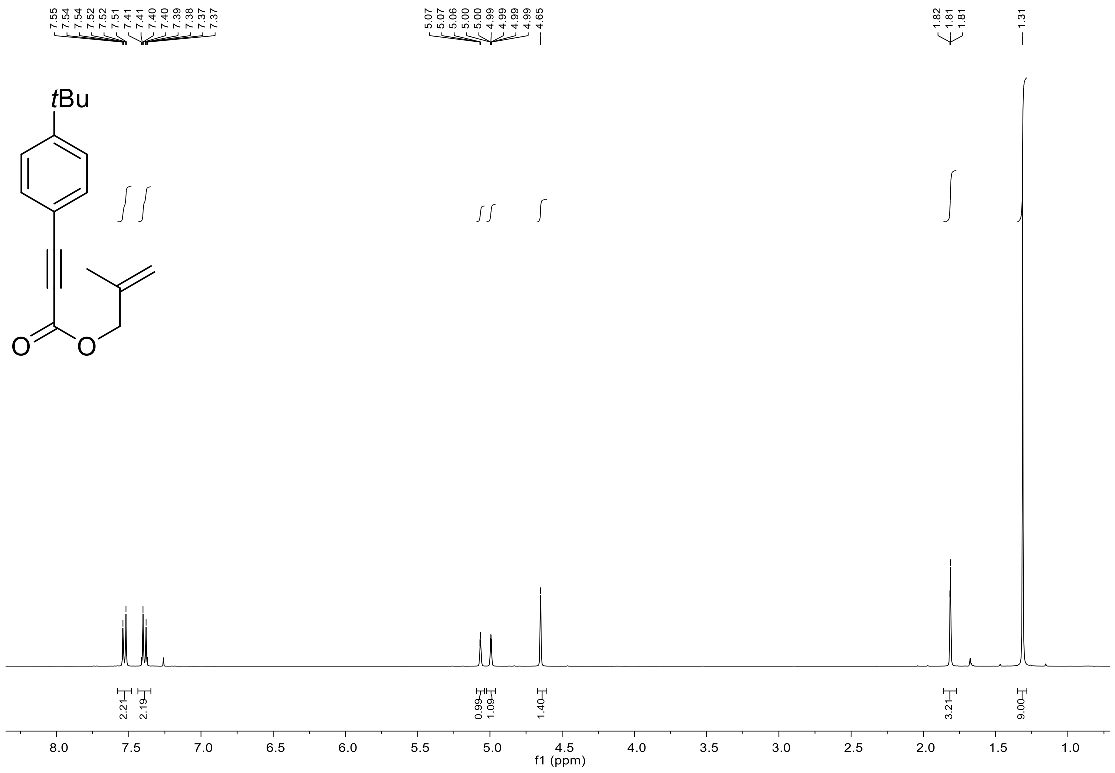

^{1}H NMR spectrum of **7a**

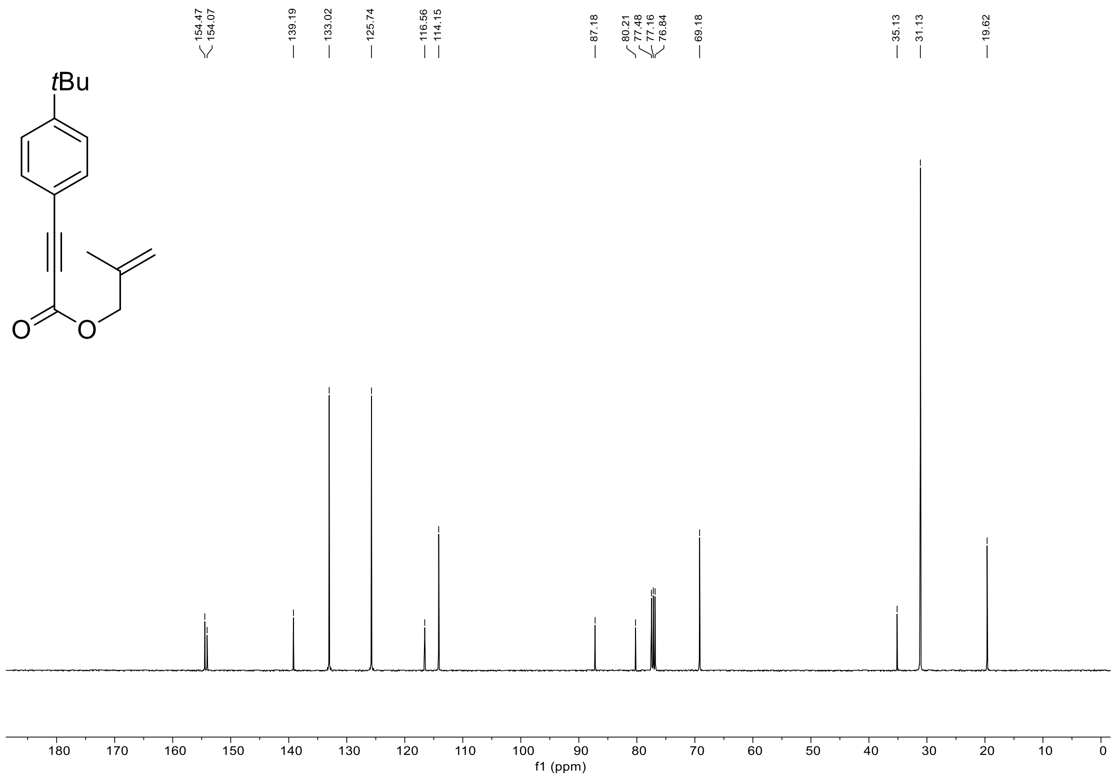

^{13}C NMR spectrum of **7a**

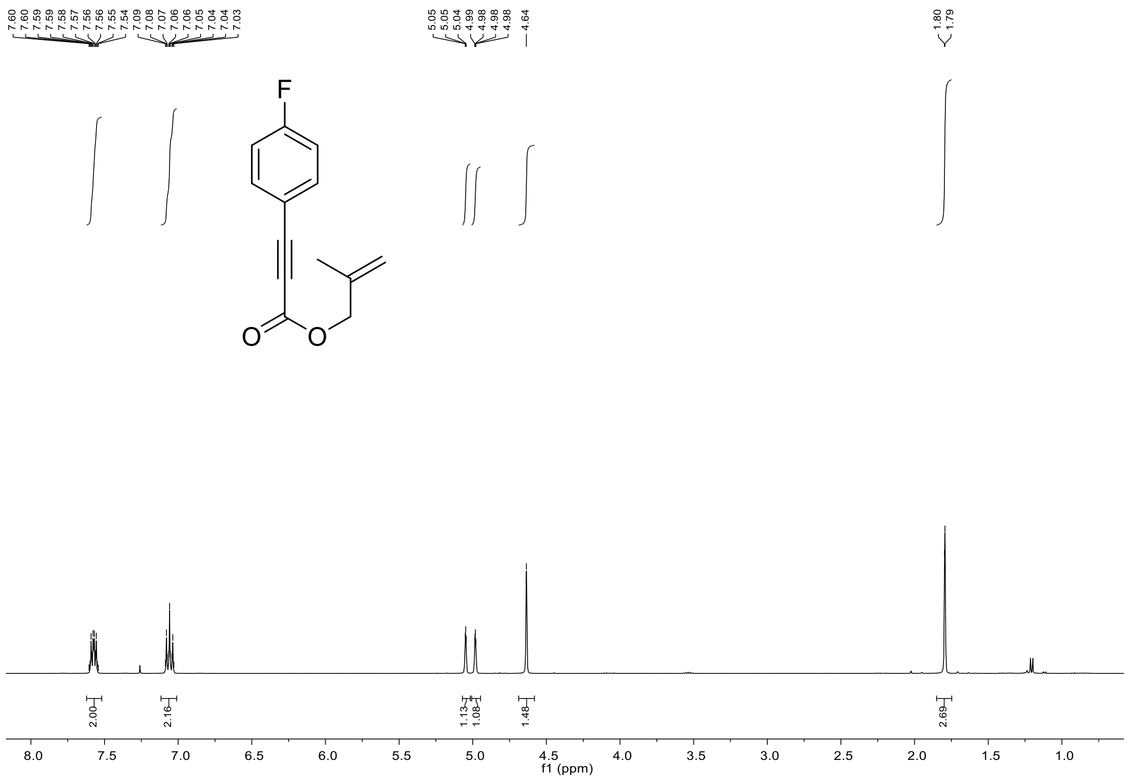

^{1}H NMR spectrum of **8a**

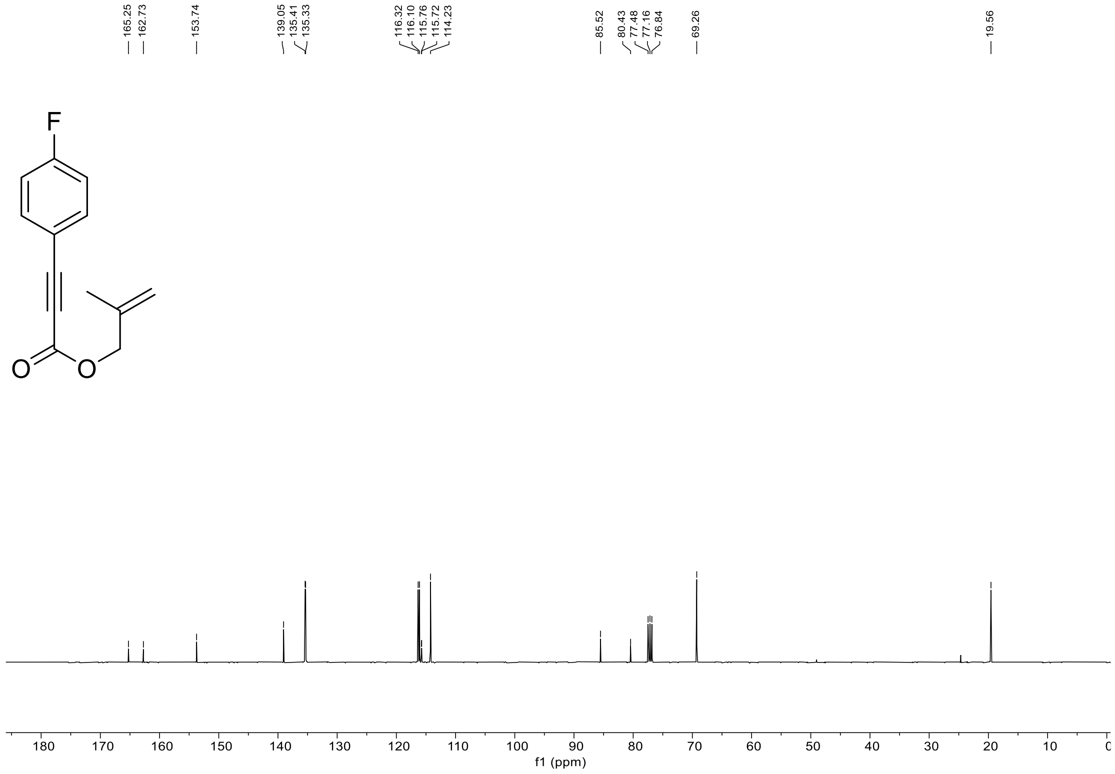

^{13}C NMR spectrum of **8a**

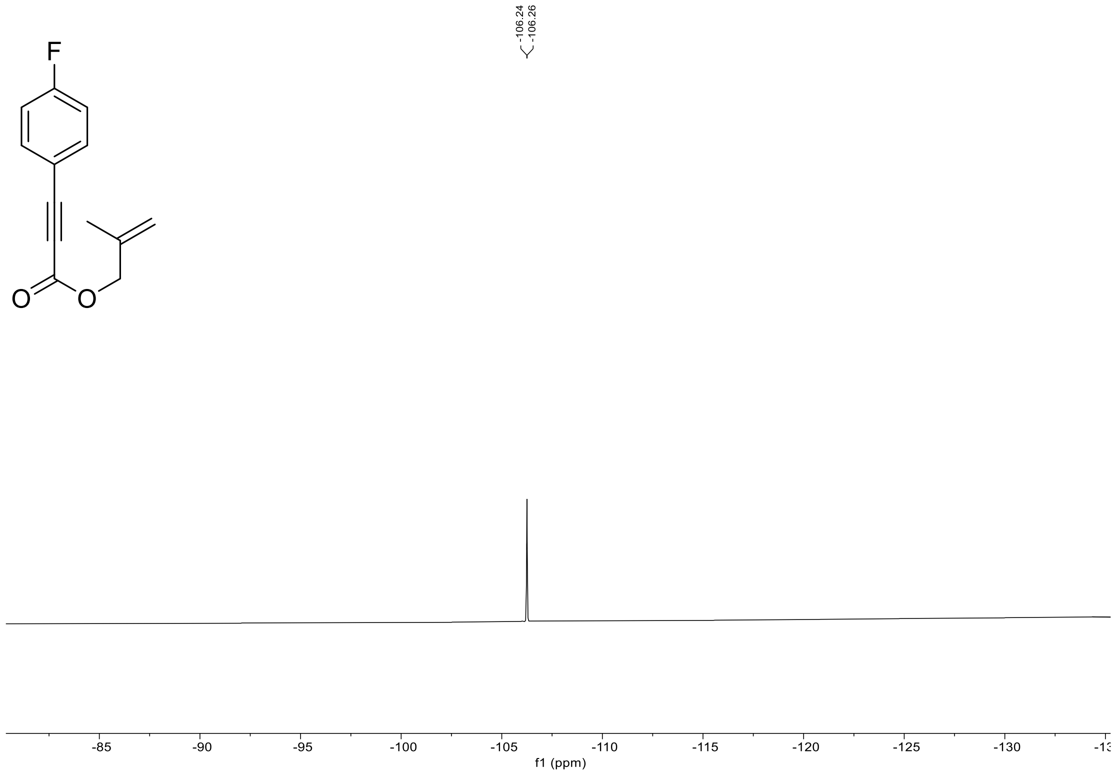

^{19}F NMR spectrum of **8a**

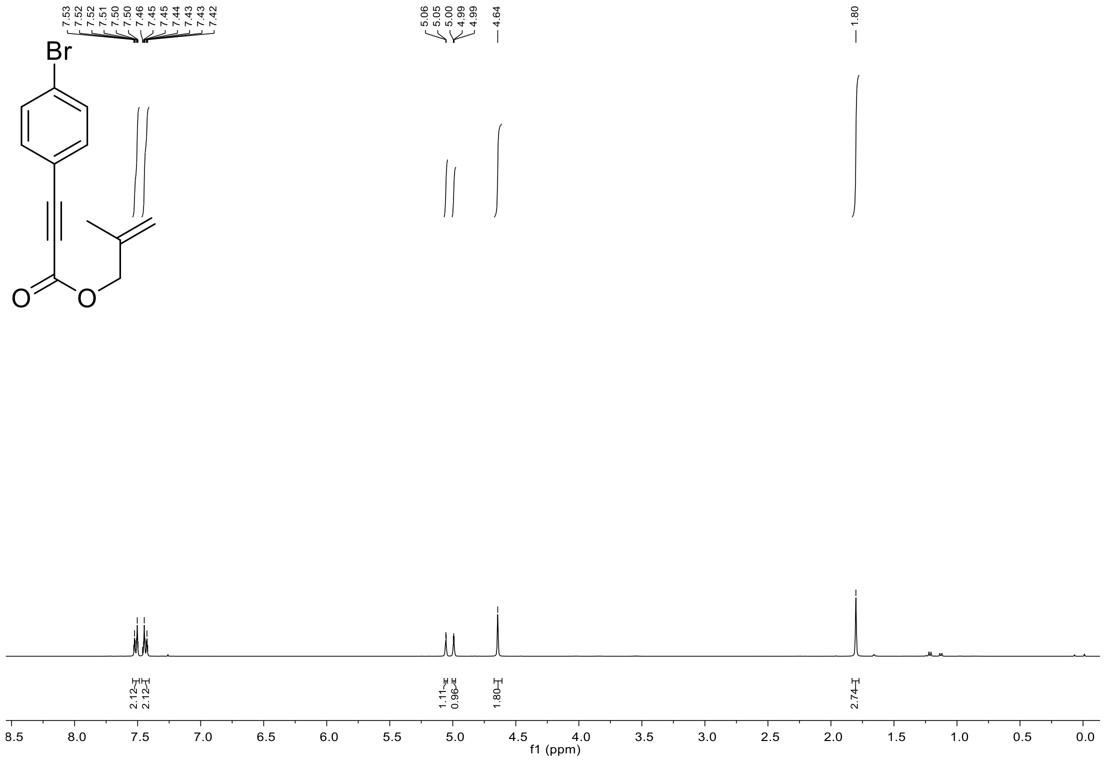

^{1}H NMR spectrum of **10a**

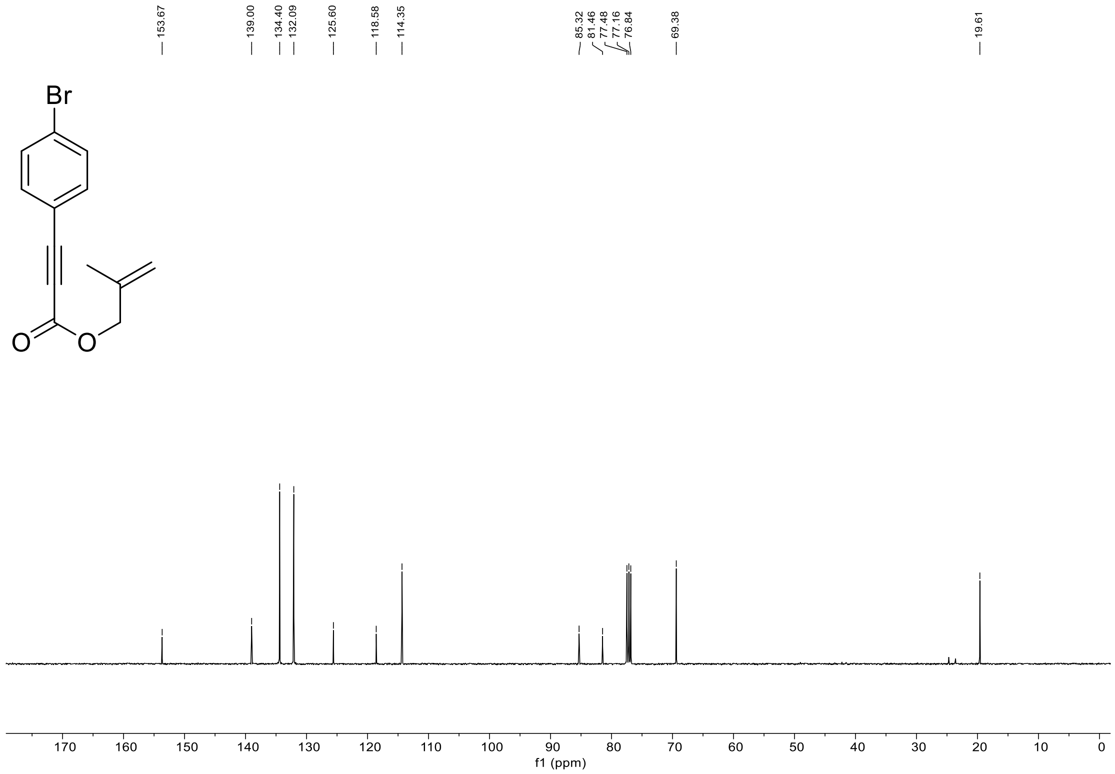

^{13}C NMR spectrum of **10a**

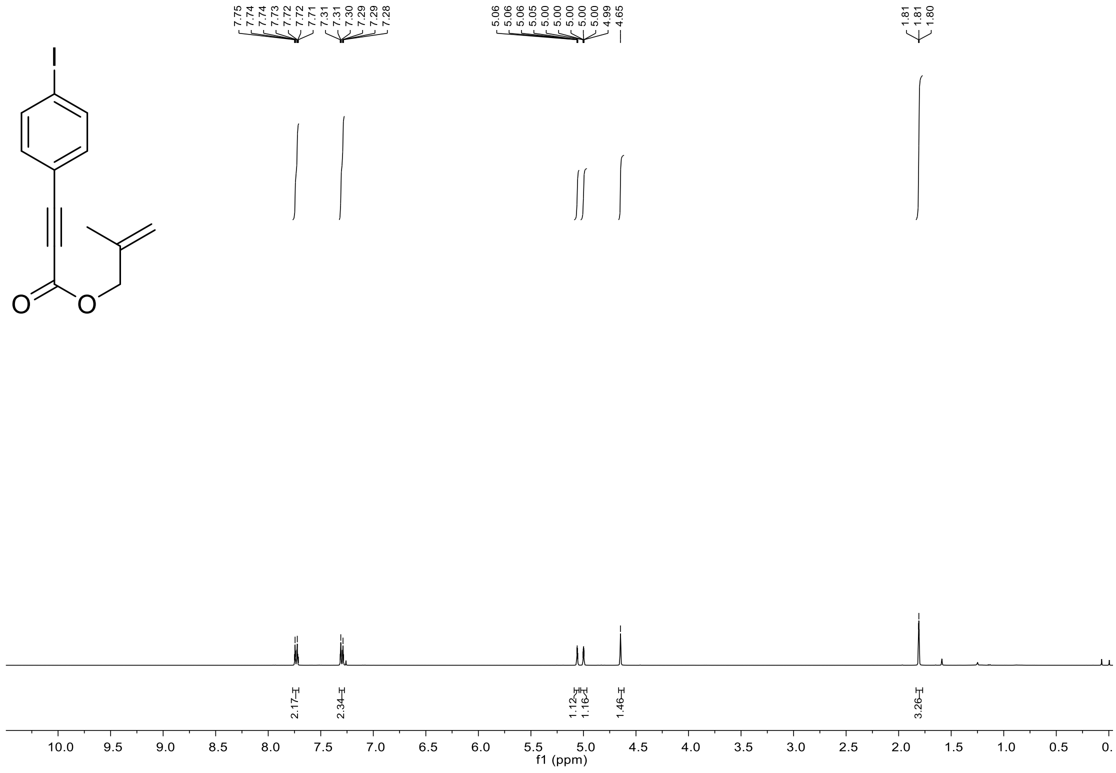

^{1}H NMR spectrum of **11a**

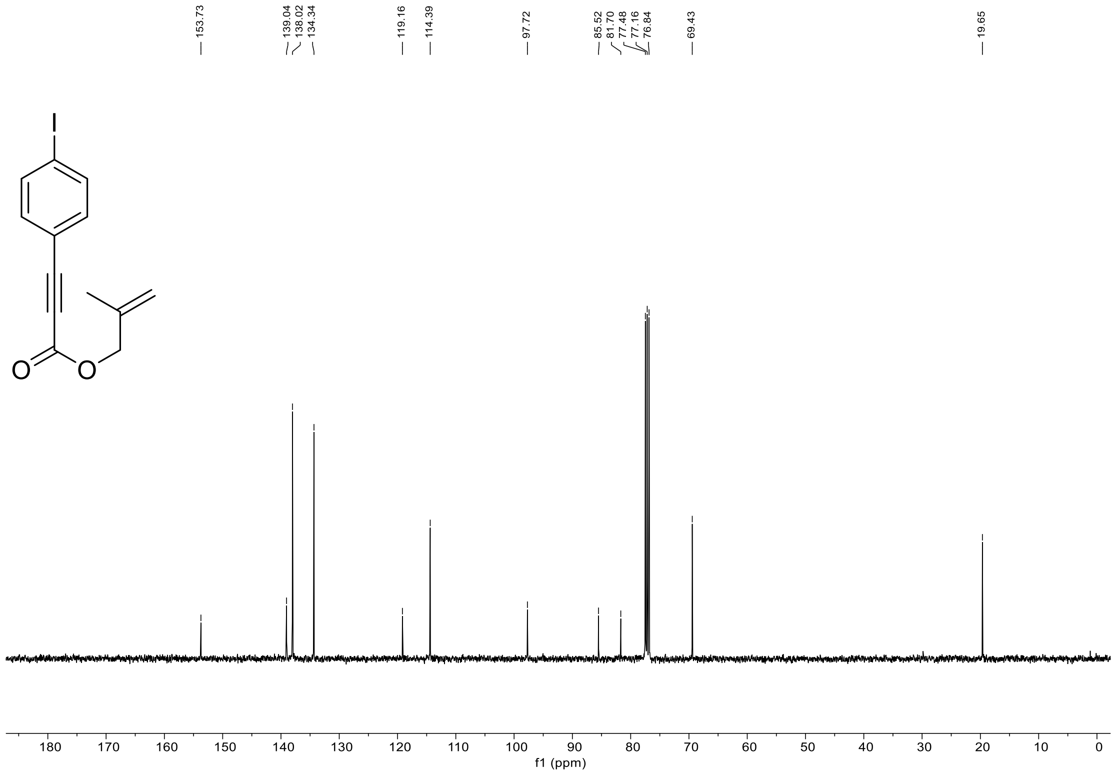

^{13}C NMR spectrum of **11a**

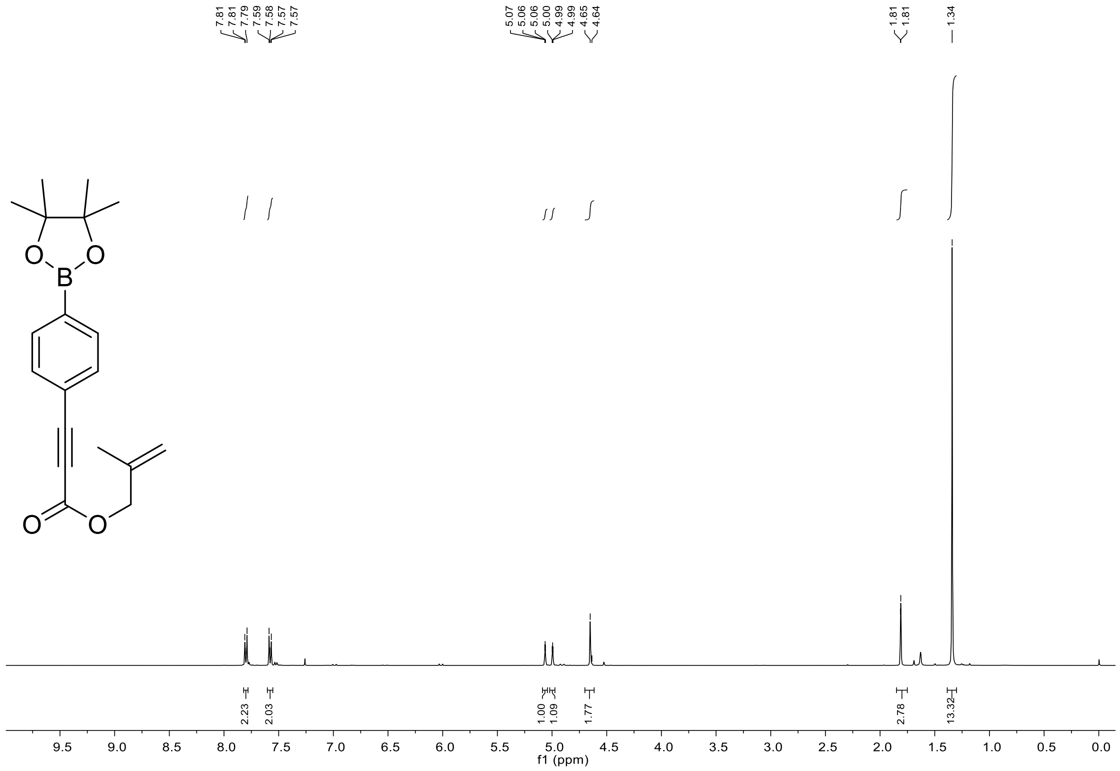

^{1}H NMR spectrum of 1**2a**

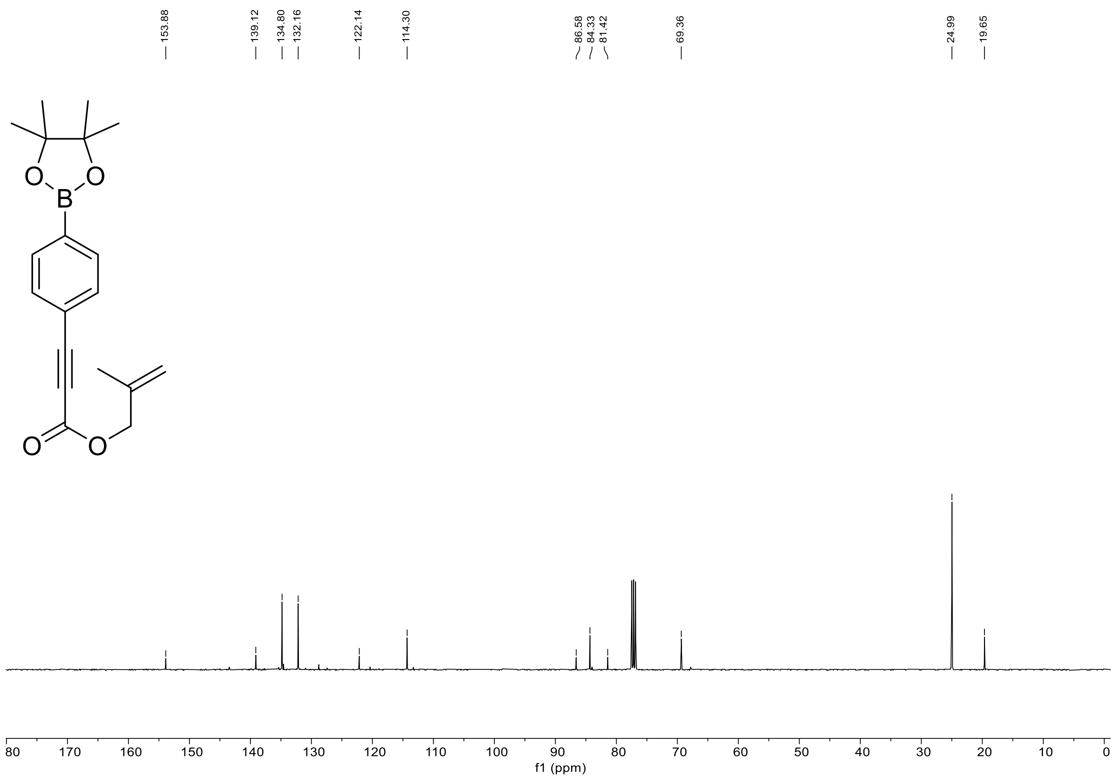

^{13}C NMR spectrum of **12a**

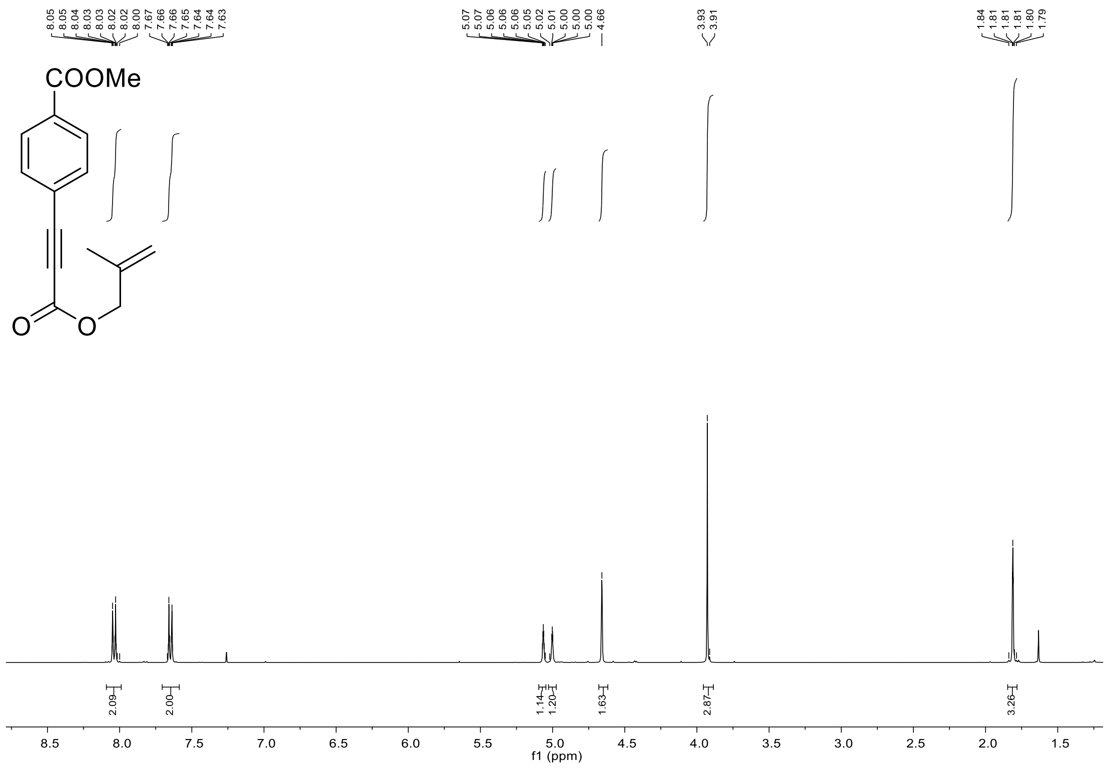

^{1}H NMR spectrum of **13a**

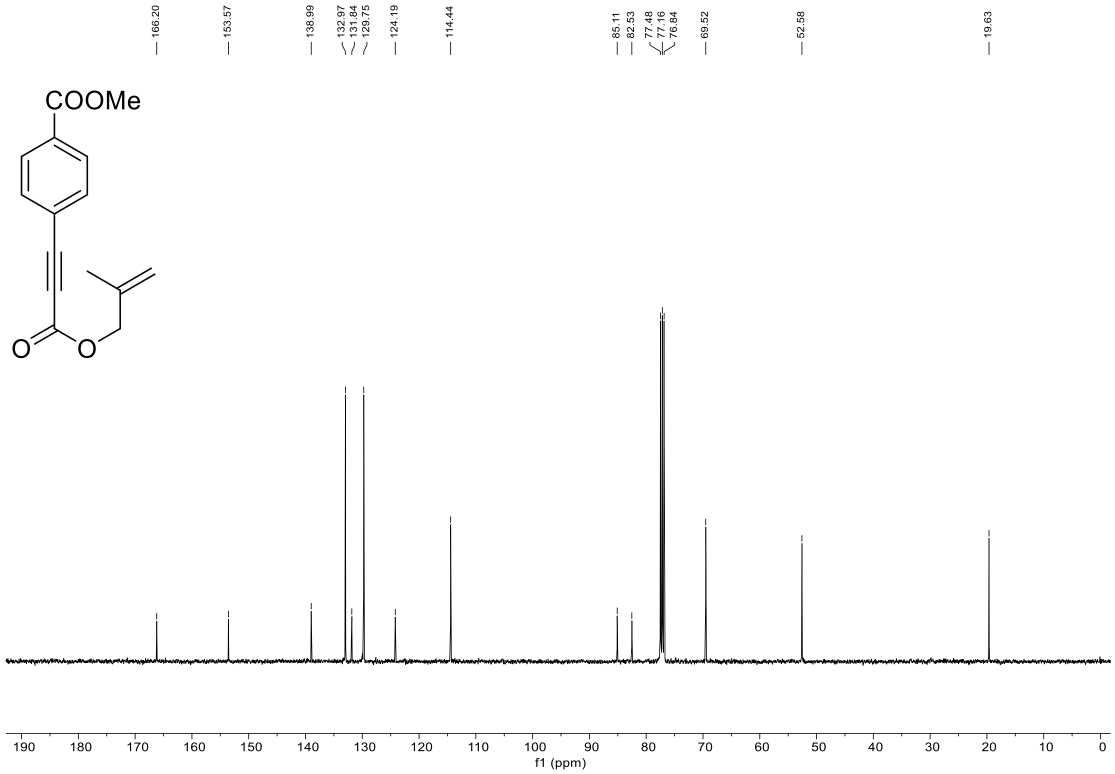

^{13}C NMR spectrum of **13a**

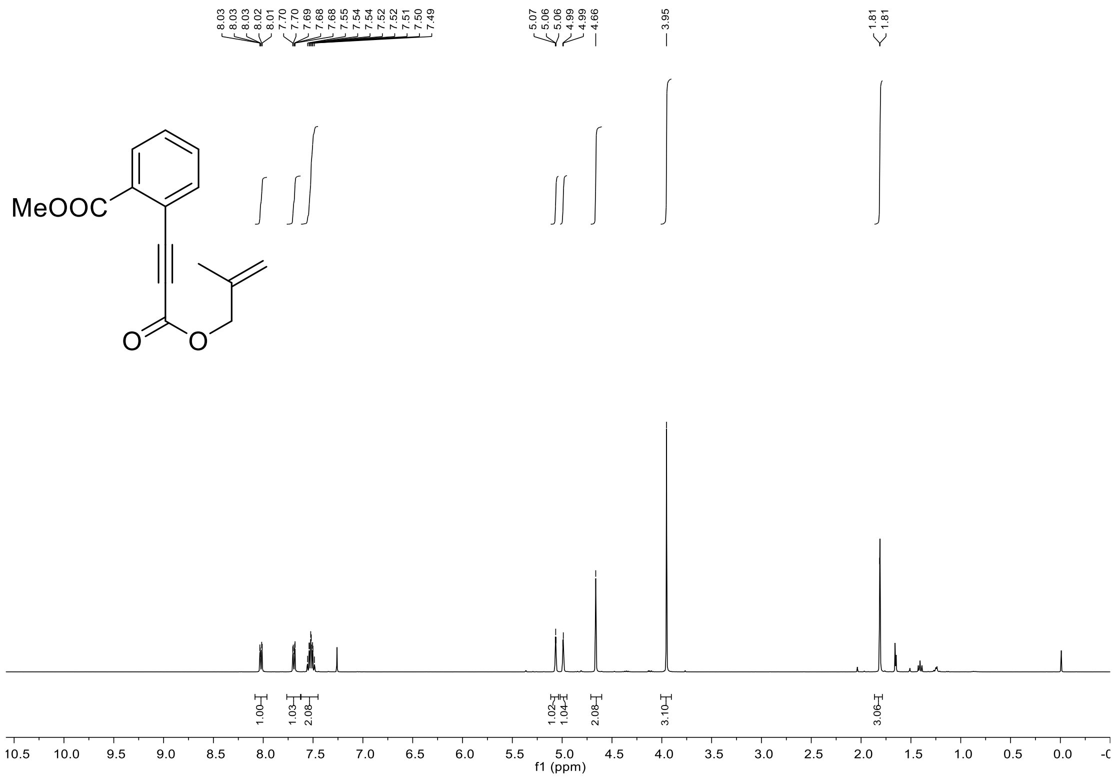

^{1}H NMR spectrum of **14a**

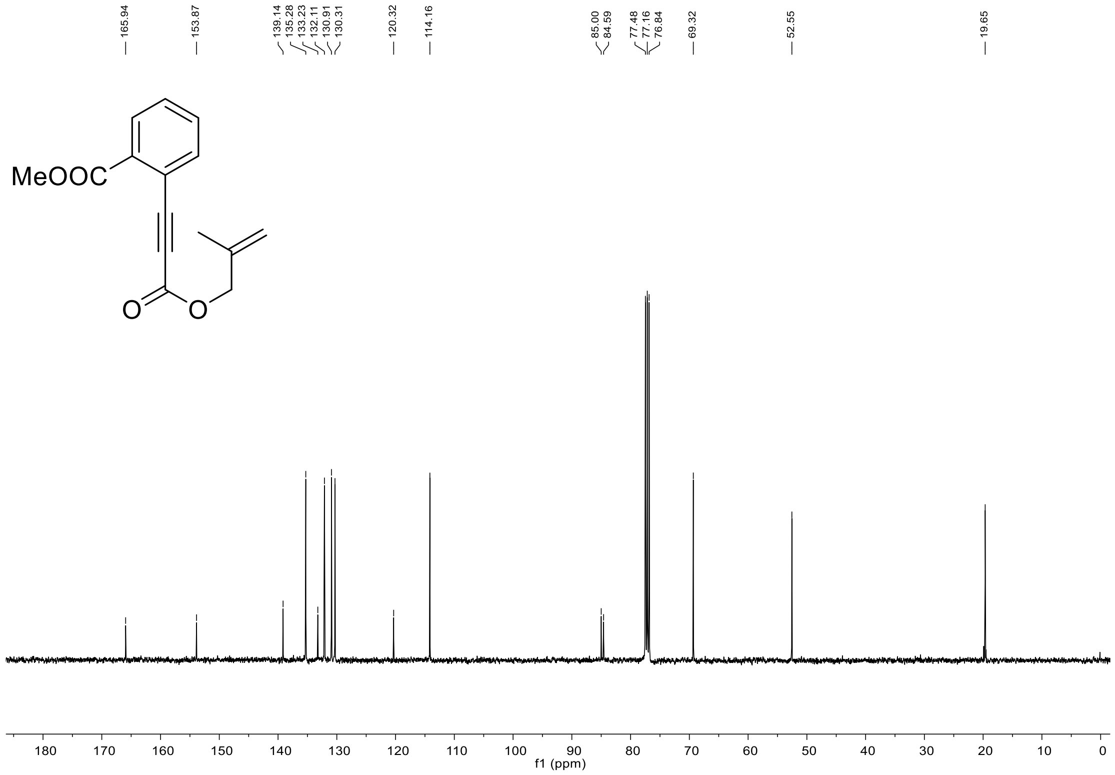

^{13}C NMR spectrum of **14a**

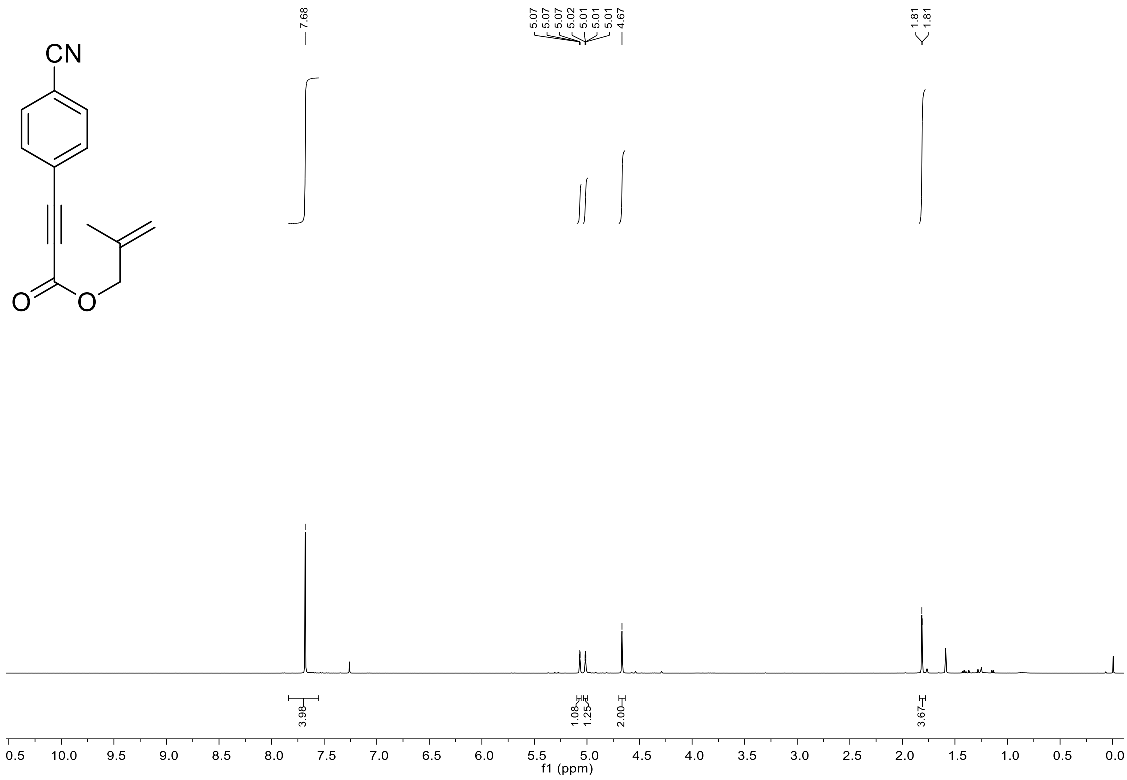

^{1}H NMR spectrum of **15a**

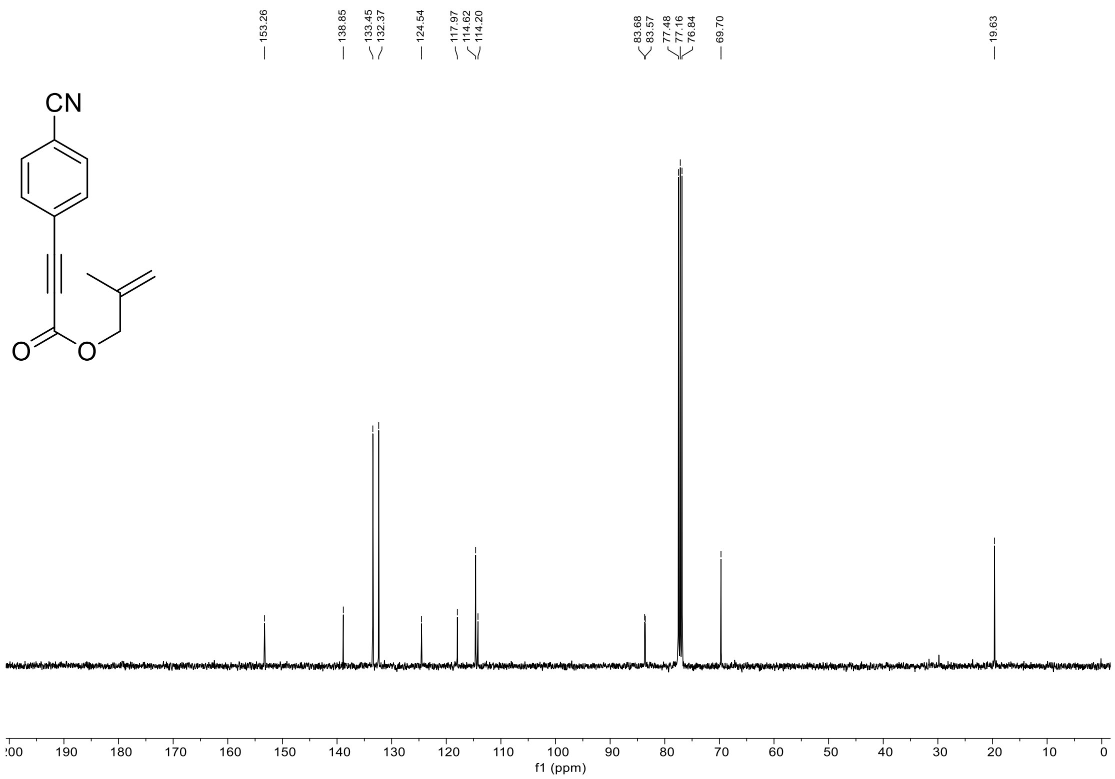

^{13}C NMR spectrum of **15a**

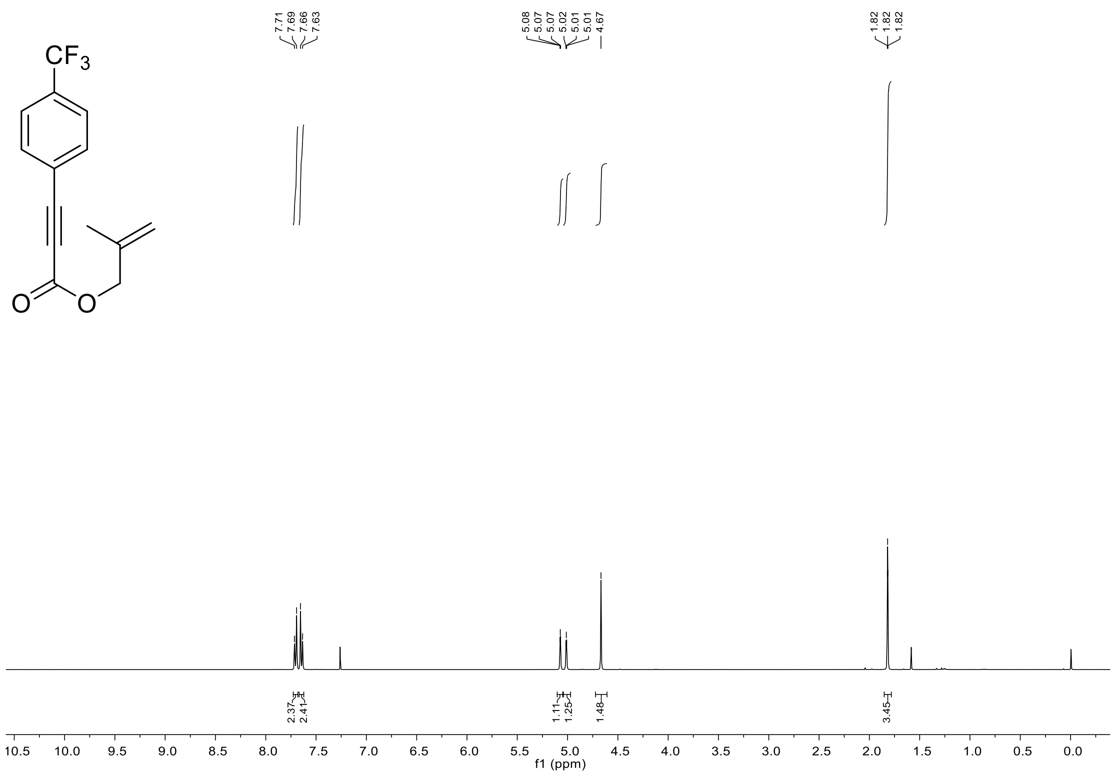

^{1}H NMR spectrum of **16a**

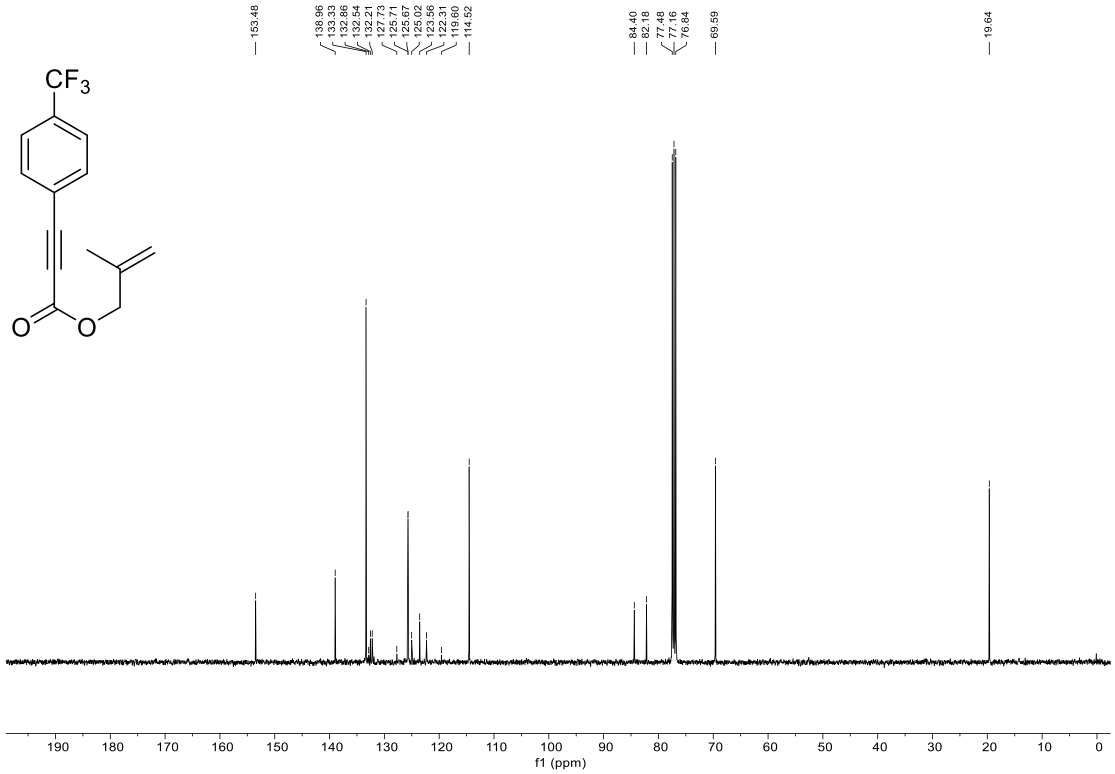

^{13}C NMR spectrum of **16a**

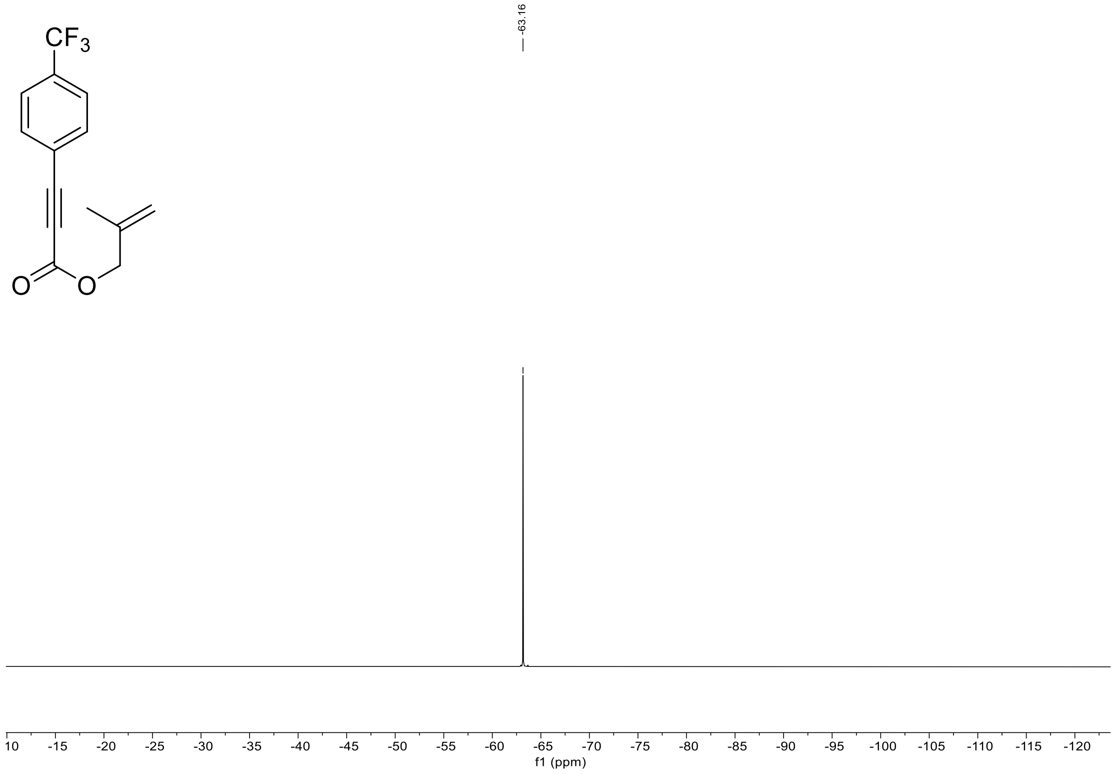

^{19}F NMR spectrum of **16a**

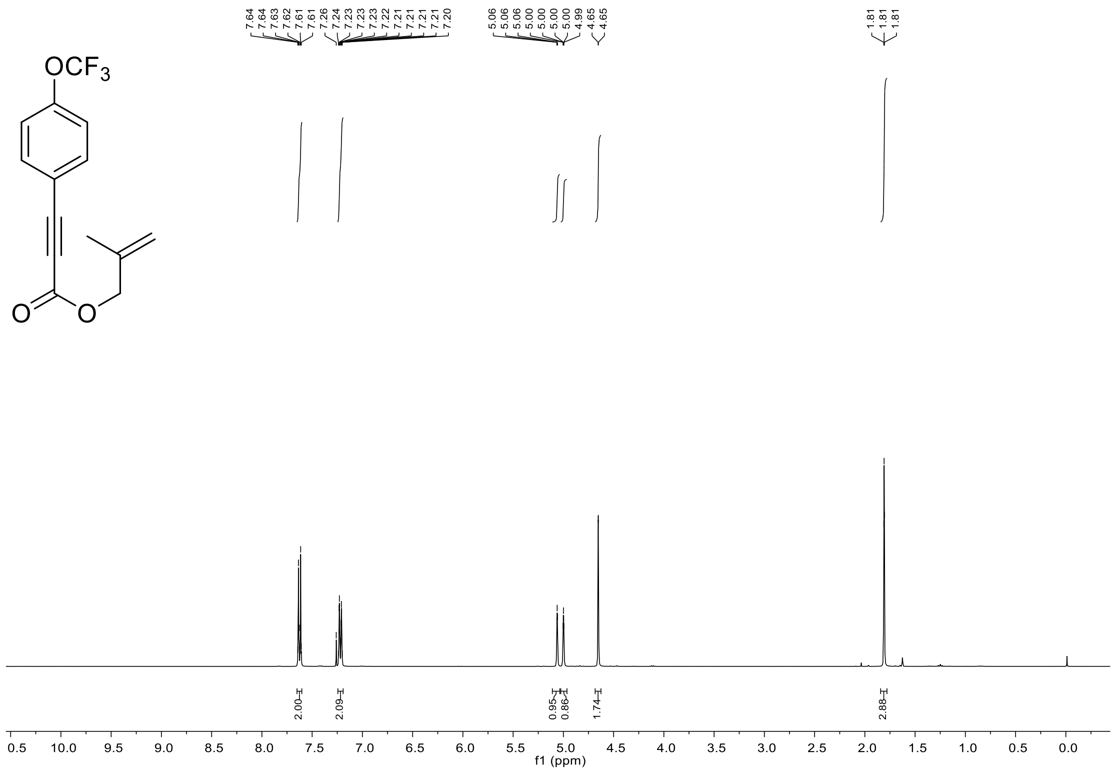

^{1}H NMR spectrum of **17a**

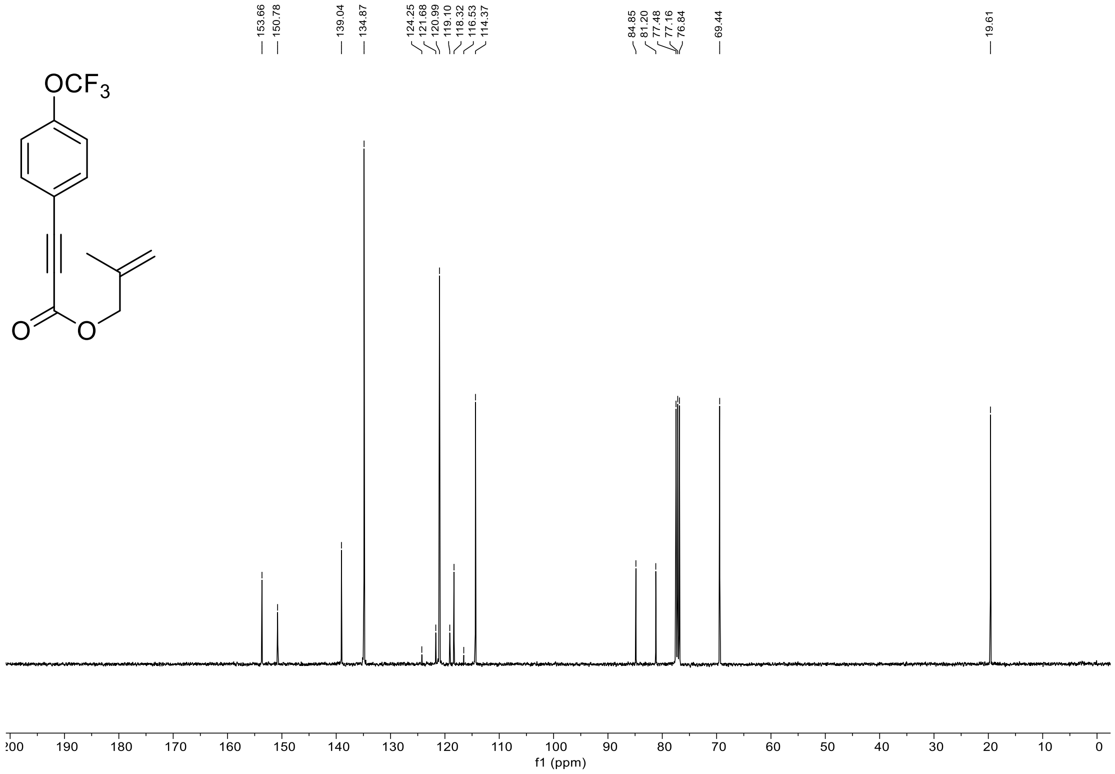

^{13}C NMR spectrum of 1**7a**

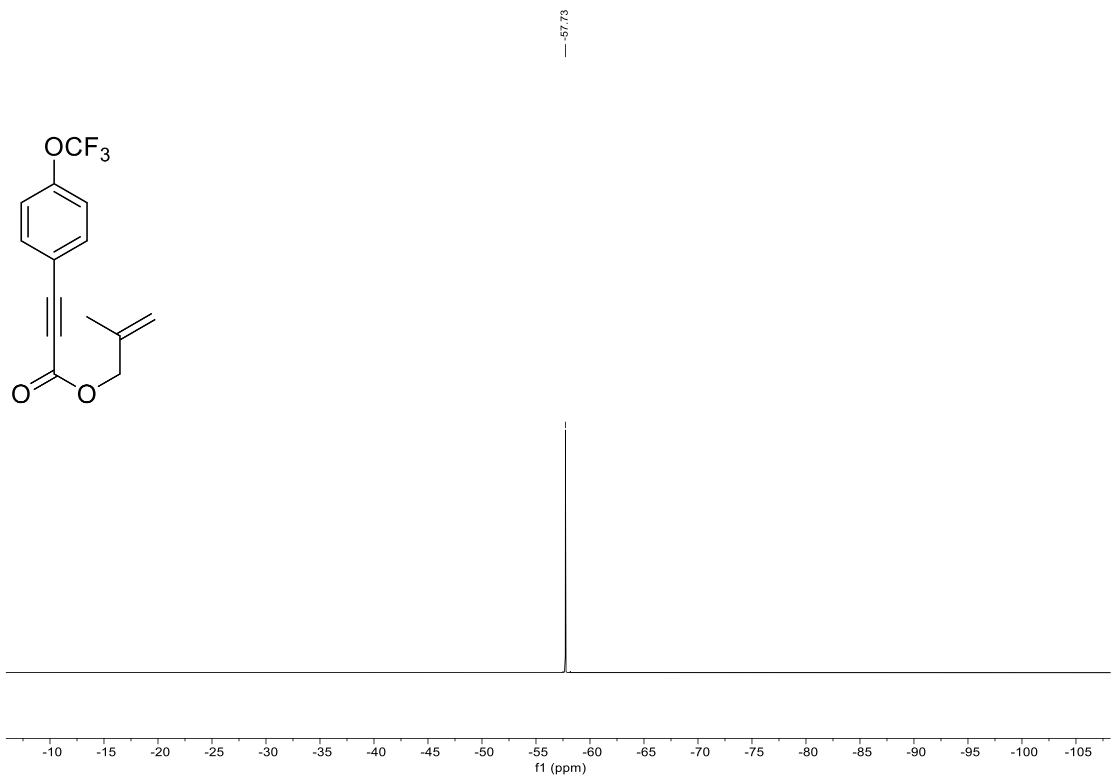

^{19}F NMR spectrum of **17a**

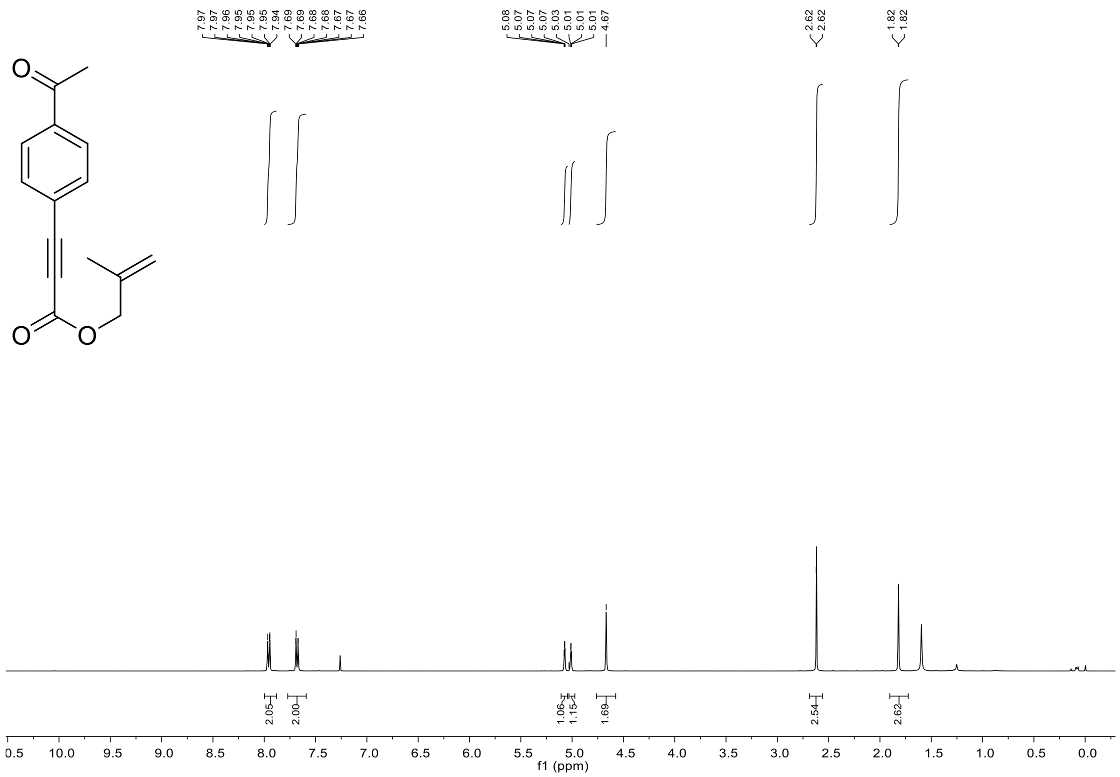

^{1}H NMR spectrum of **18a**

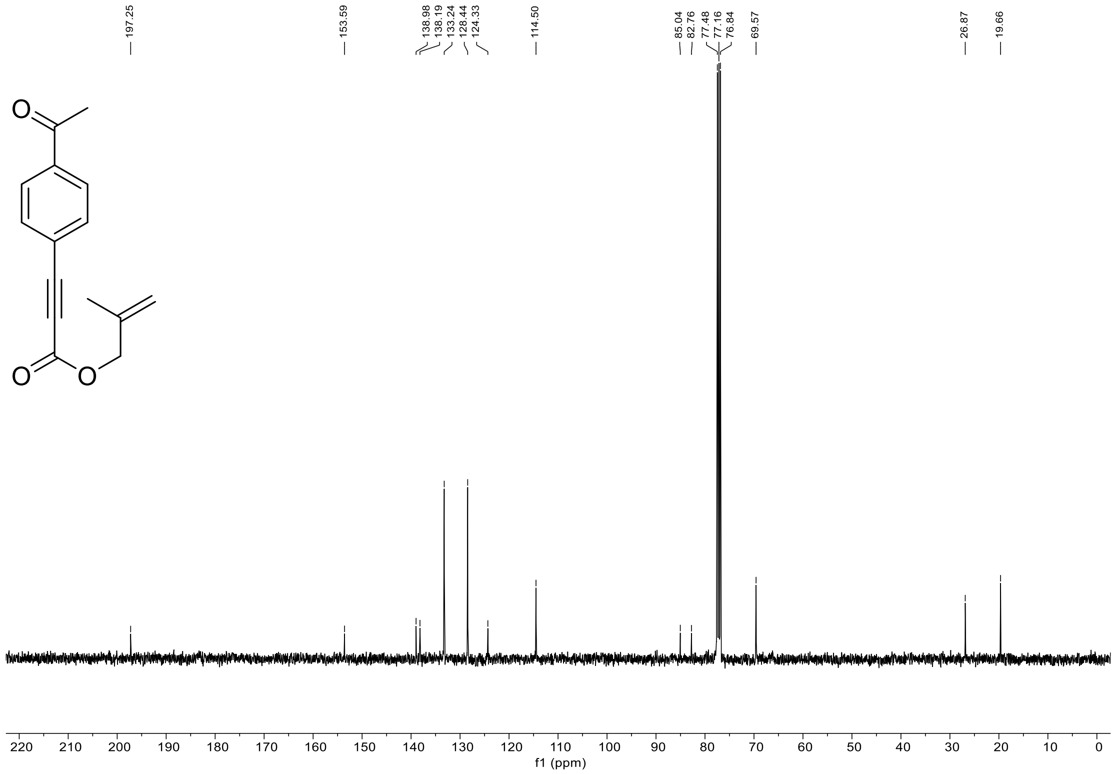

^{13}C NMR spectrum of **18a**

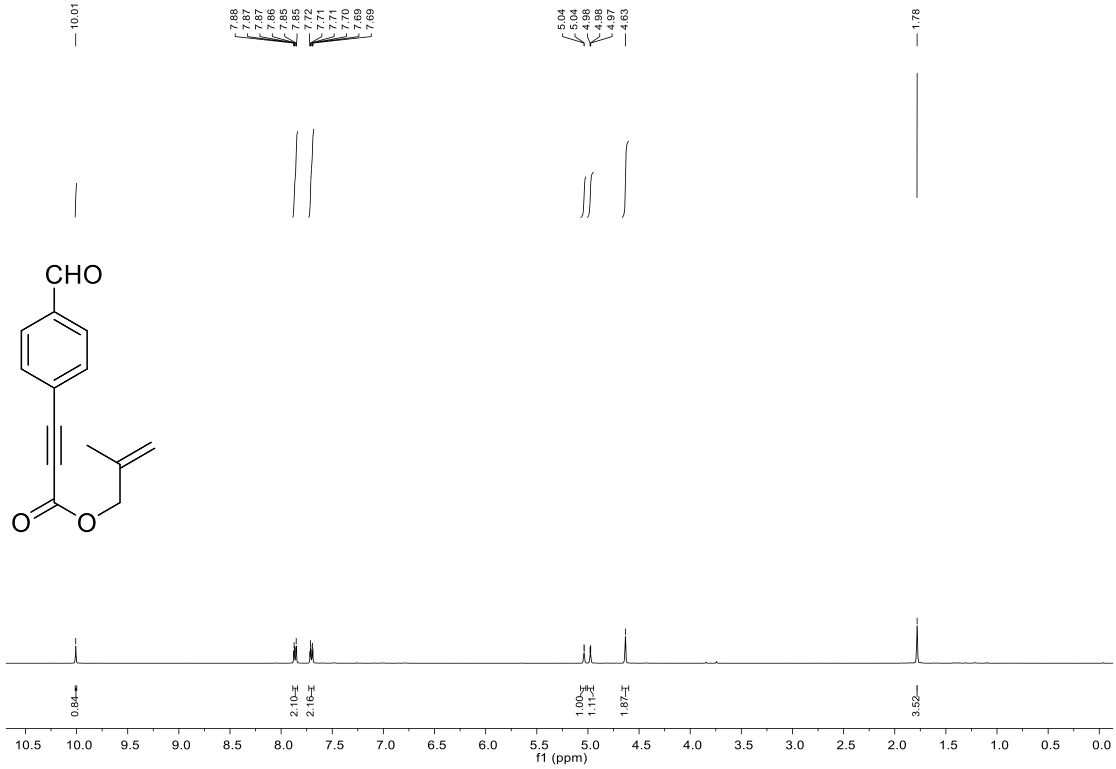

^{1}H NMR spectrum of **19a**

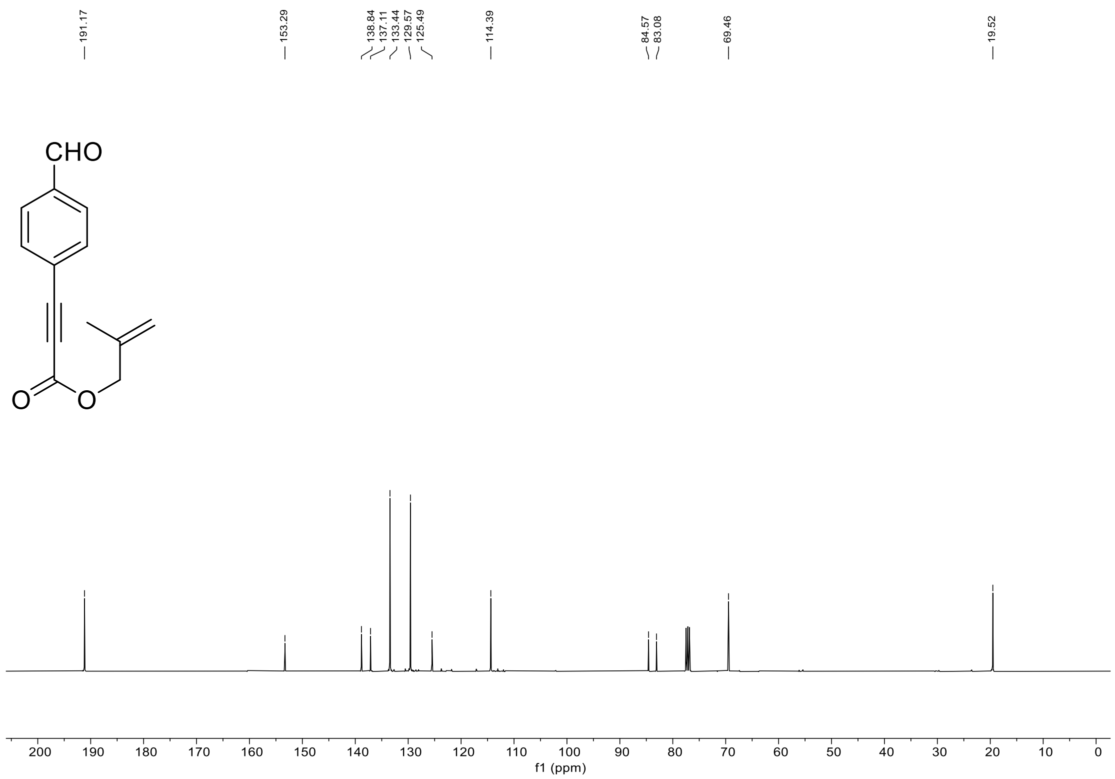

^{13}C NMR spectrum of **19a**

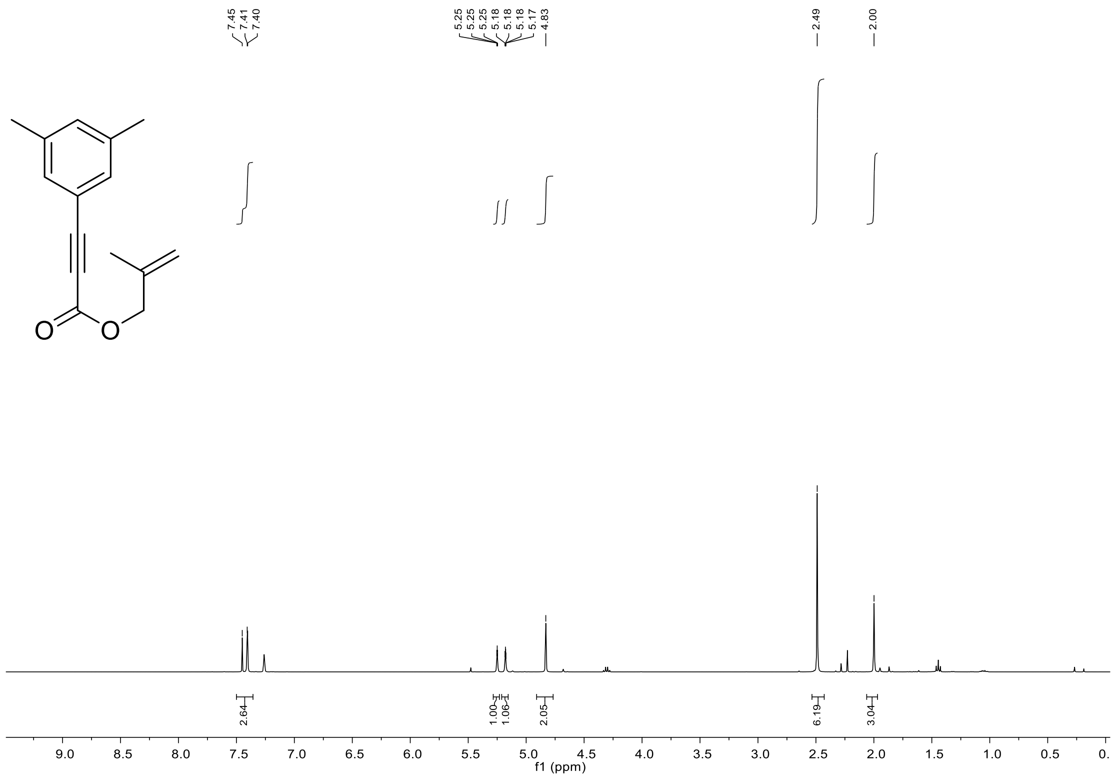

^{1}H NMR spectrum of **20a**

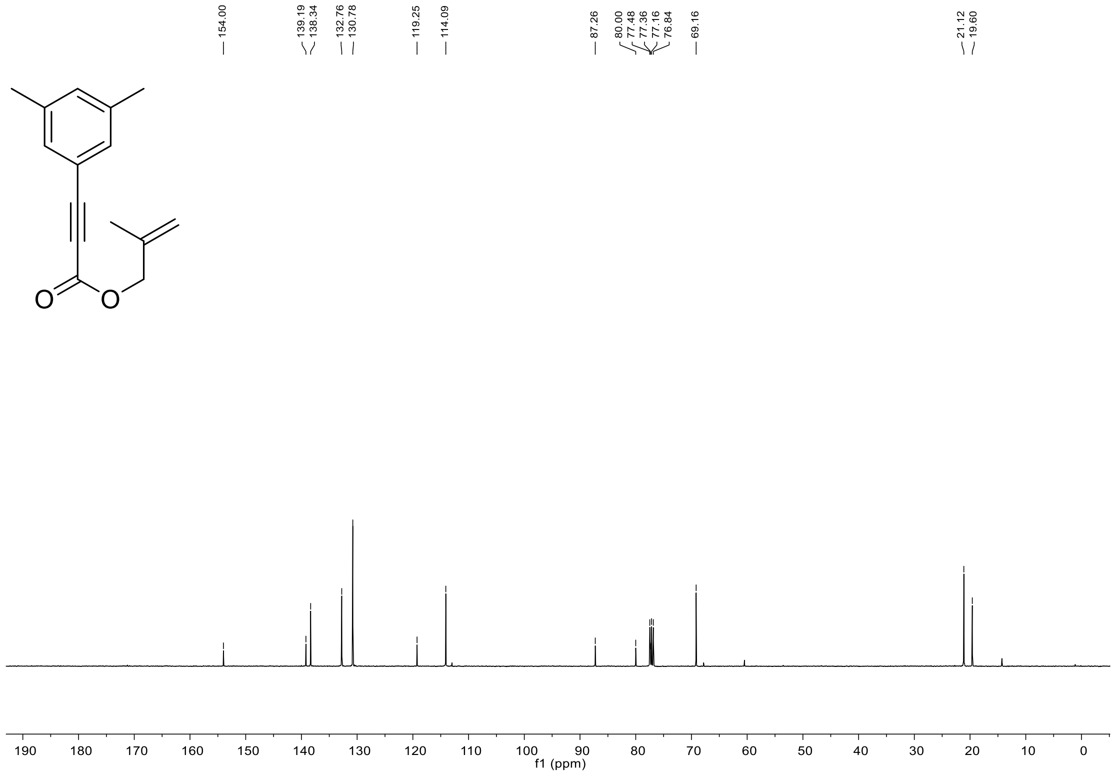

^{13}C NMR spectrum of **20a**

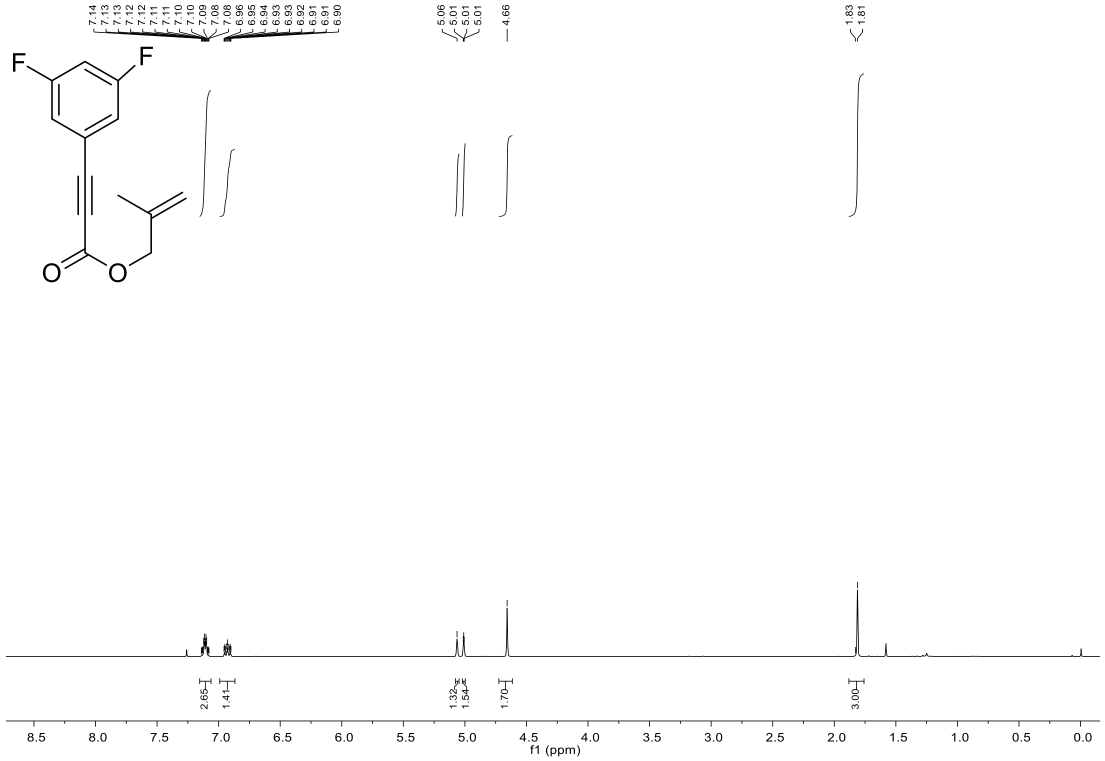

^{1}H NMR spectrum of **21a**

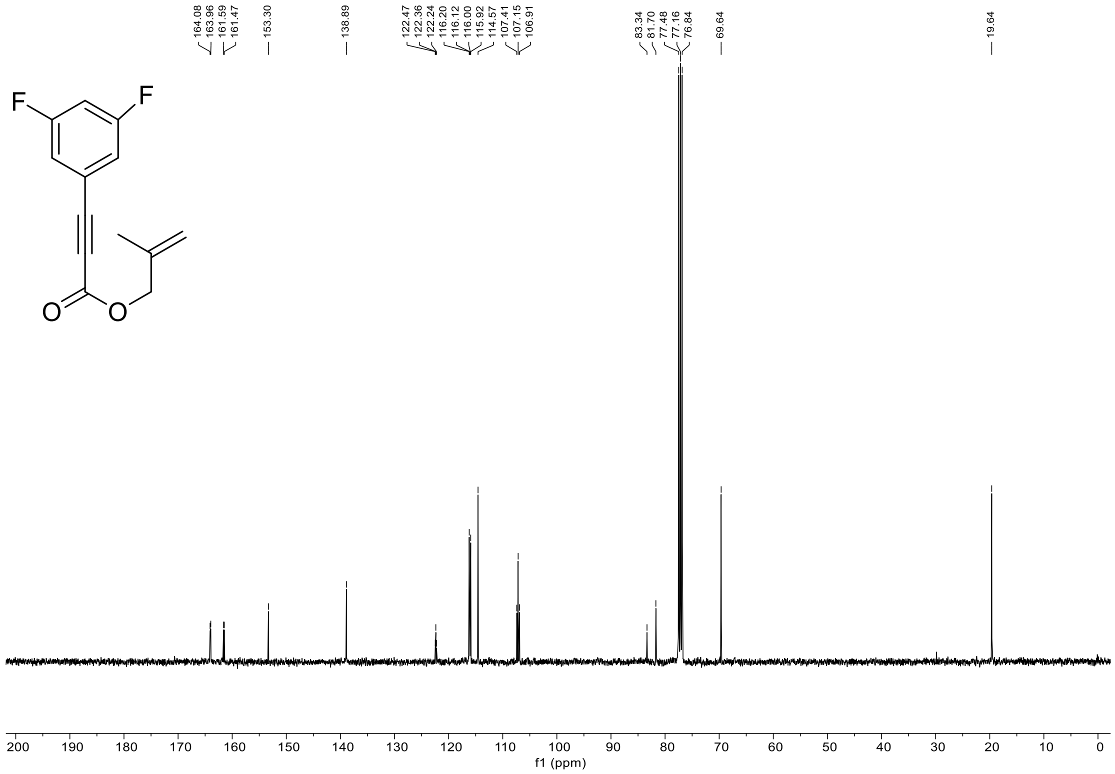

^{13}C NMR spectrum of **21a**

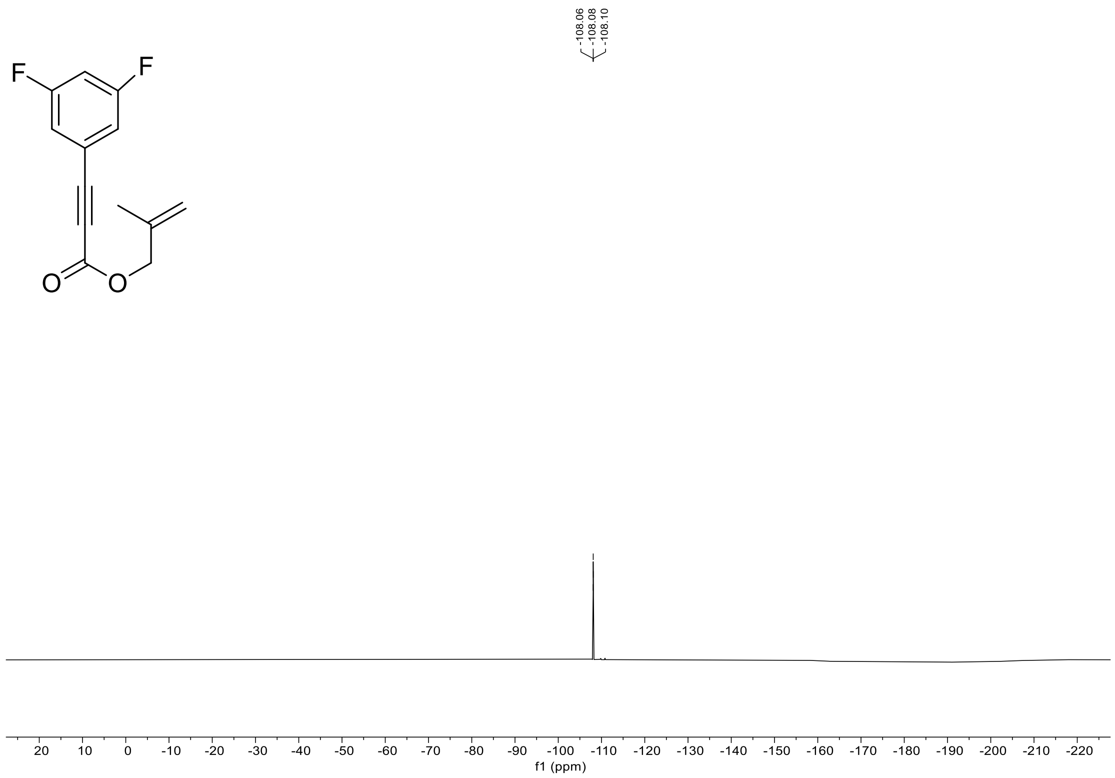

^{19}F NMR spectrum of **21a**

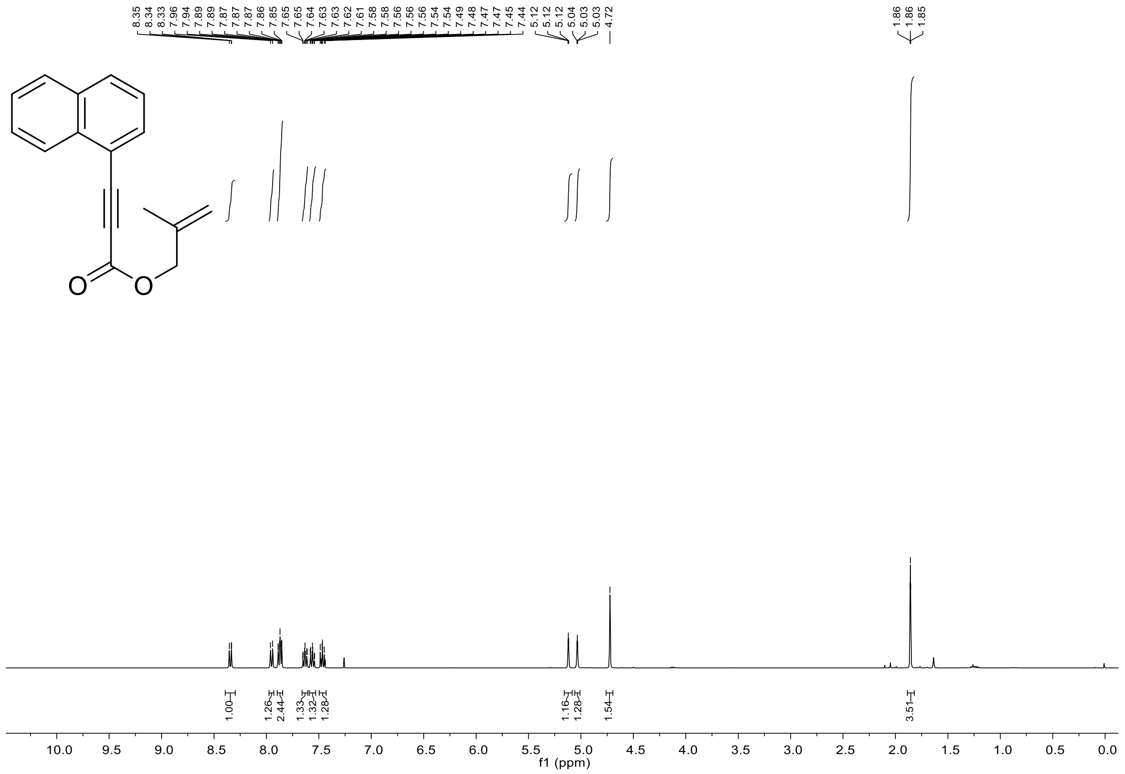

^{1}H NMR spectrum of **22a**

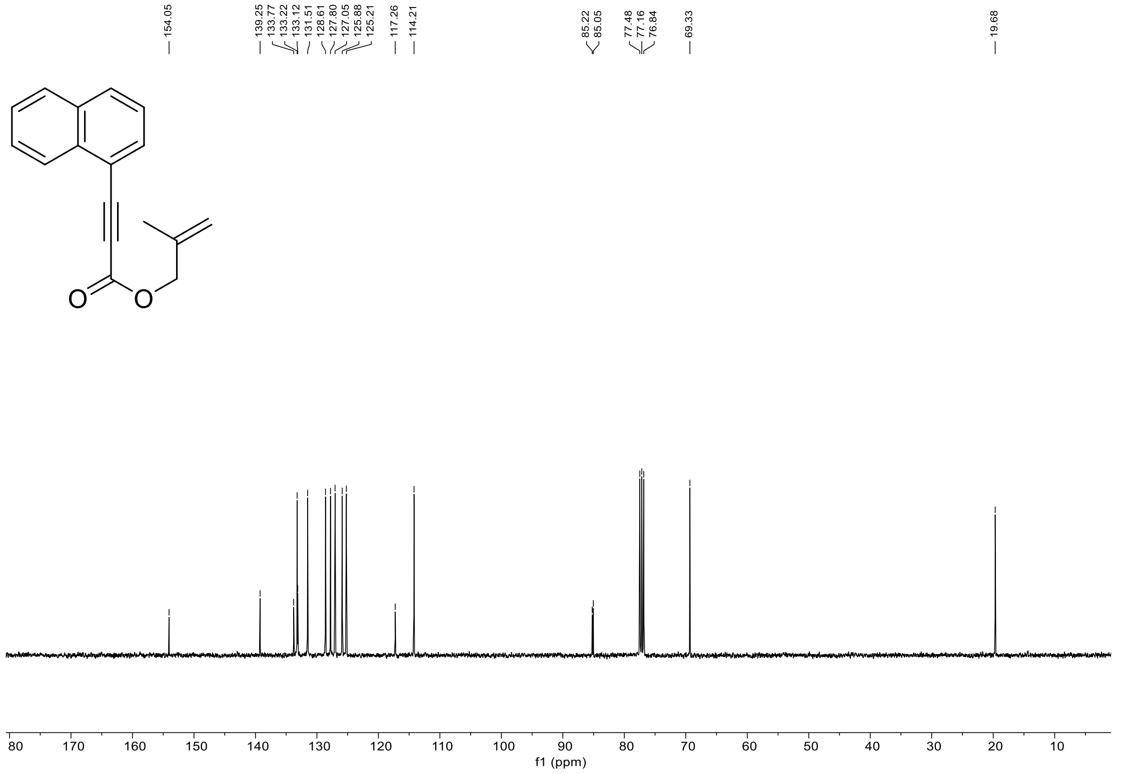

^{13}C NMR spectrum of **22a**

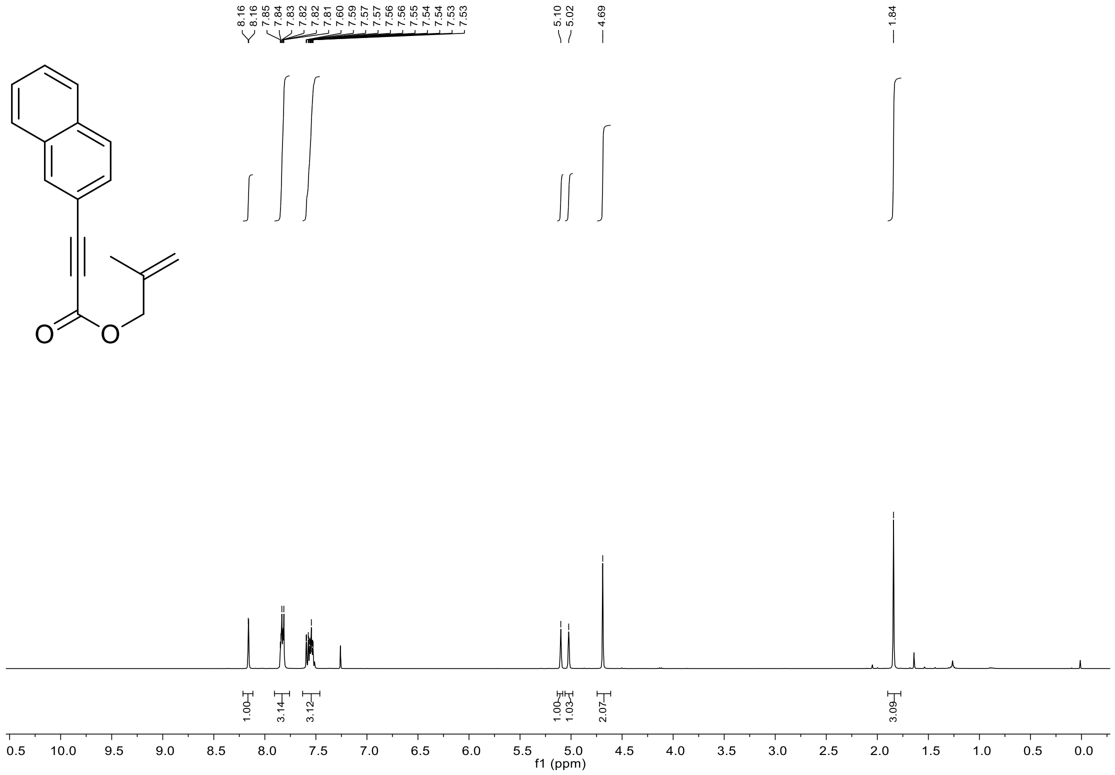

^{1}H NMR spectrum of **23a**

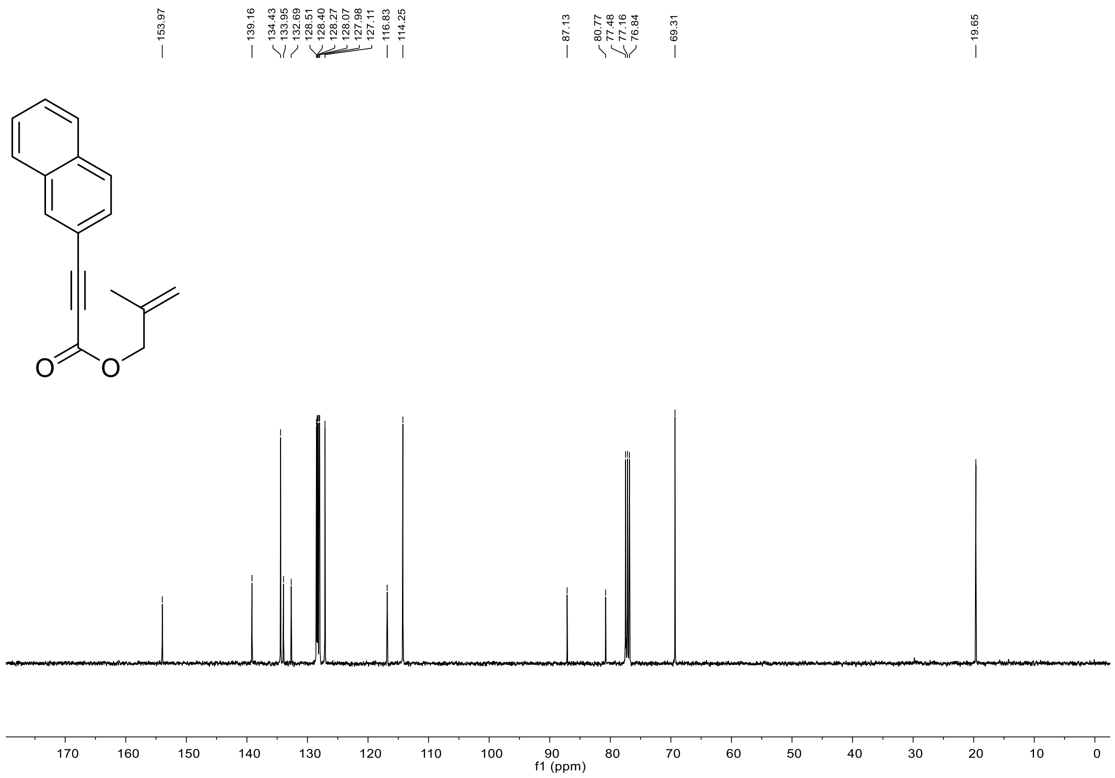

^{13}C NMR spectrum of **23a**

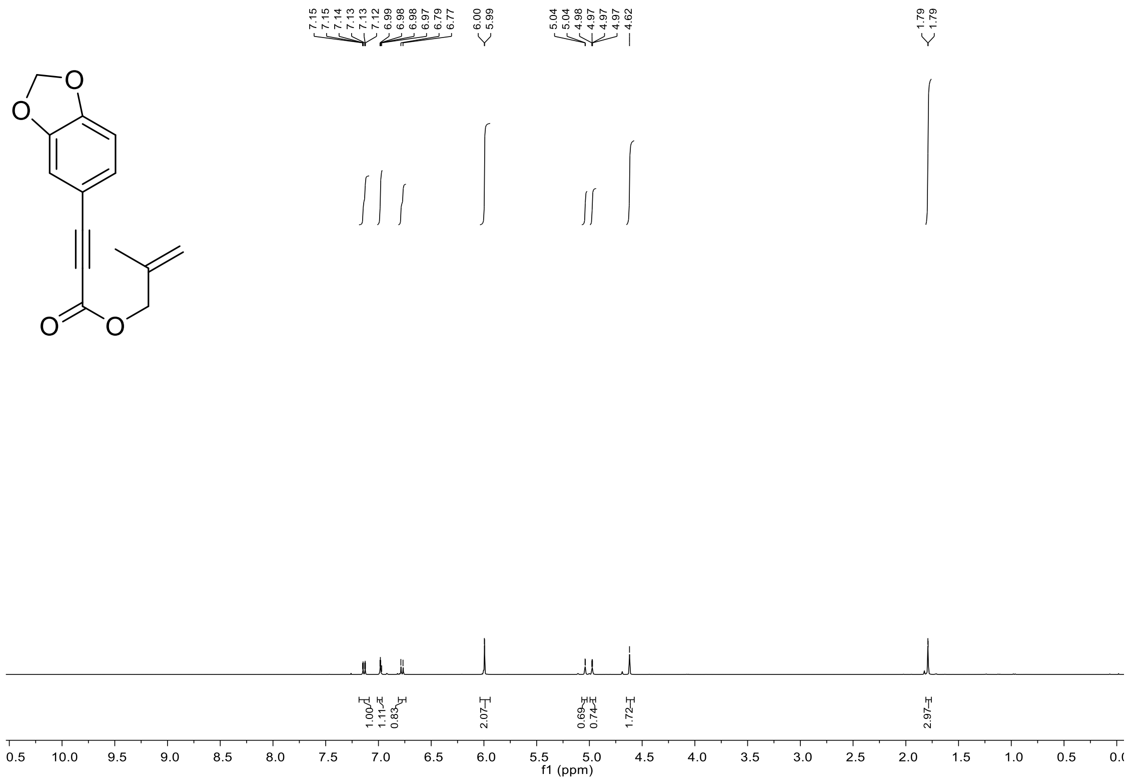

^{1}H NMR spectrum of **24a**

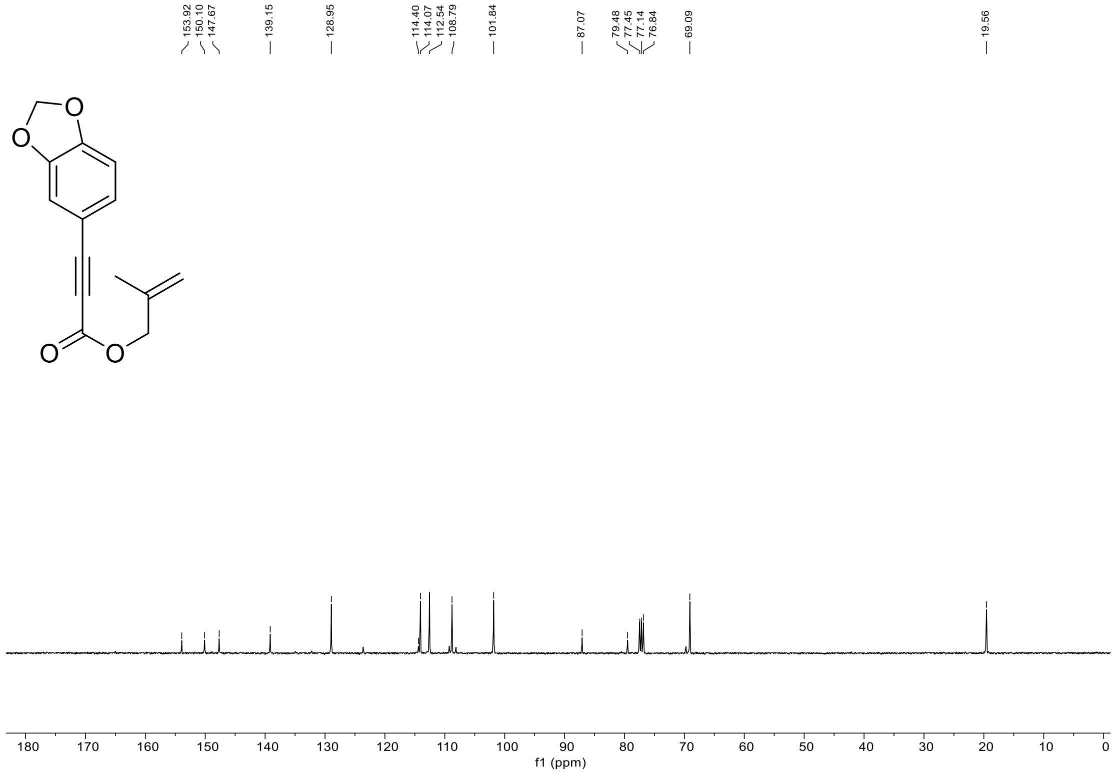

^{13}C NMR spectrum of **24a**

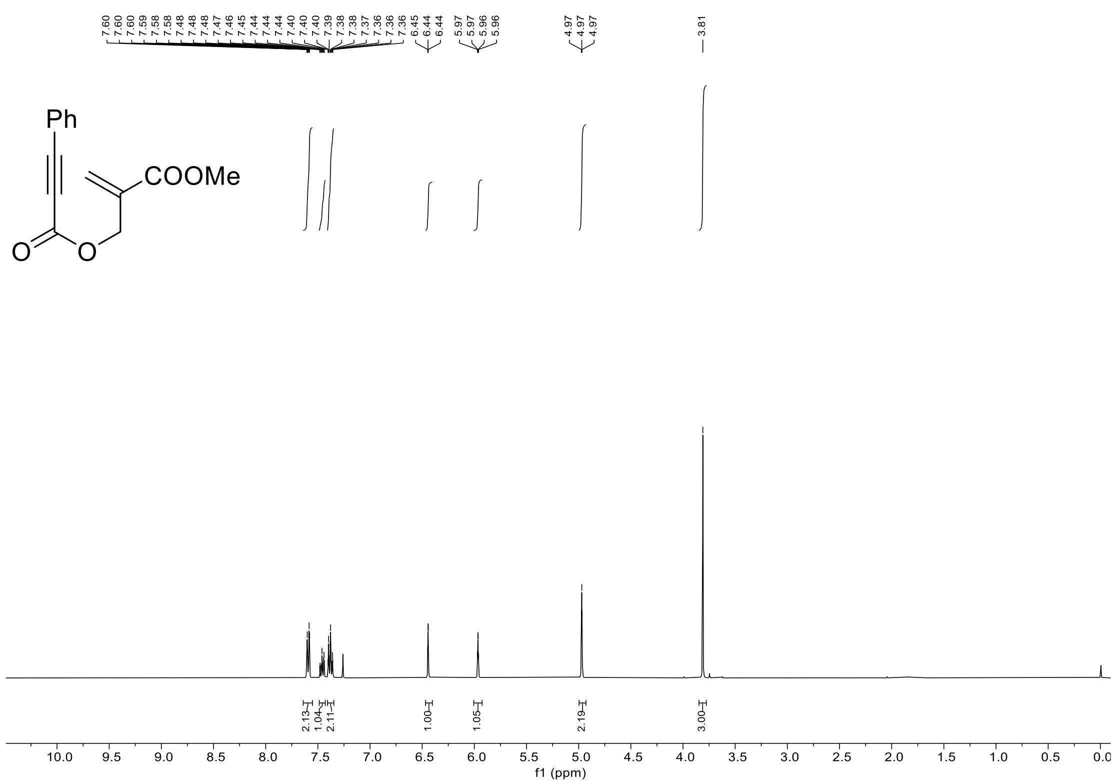

^{1}H NMR spectrum of **27a**

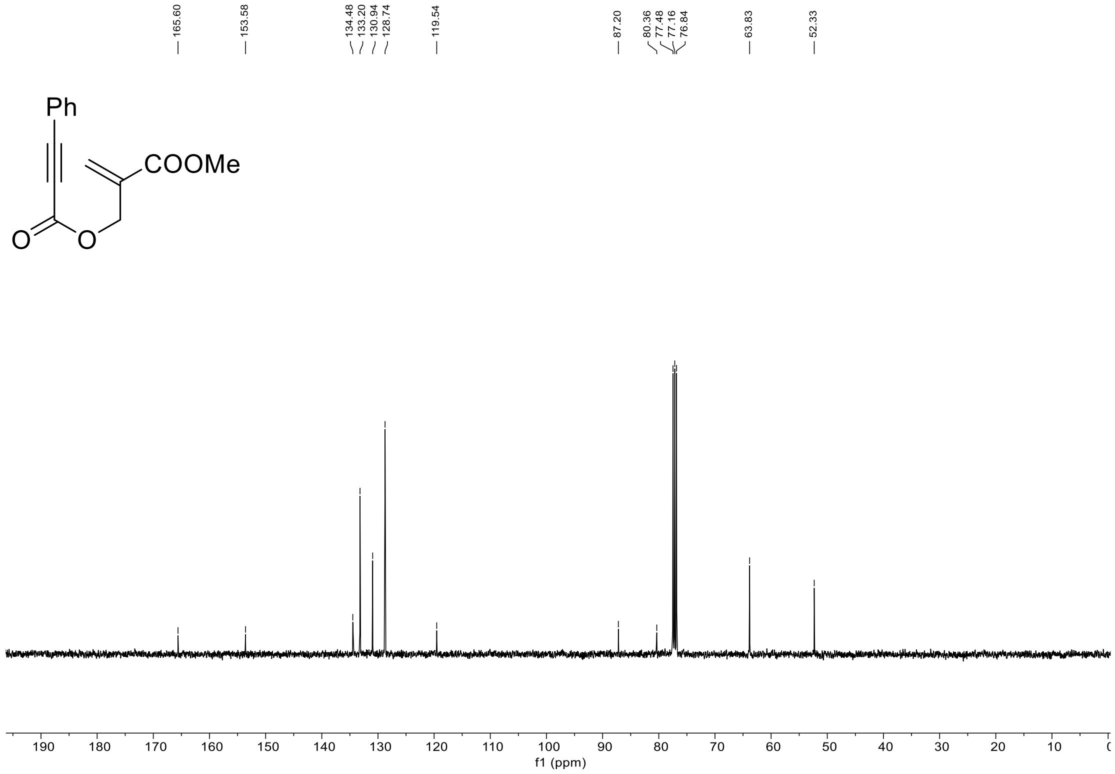

^{13}C NMR spectrum of 2**7a**

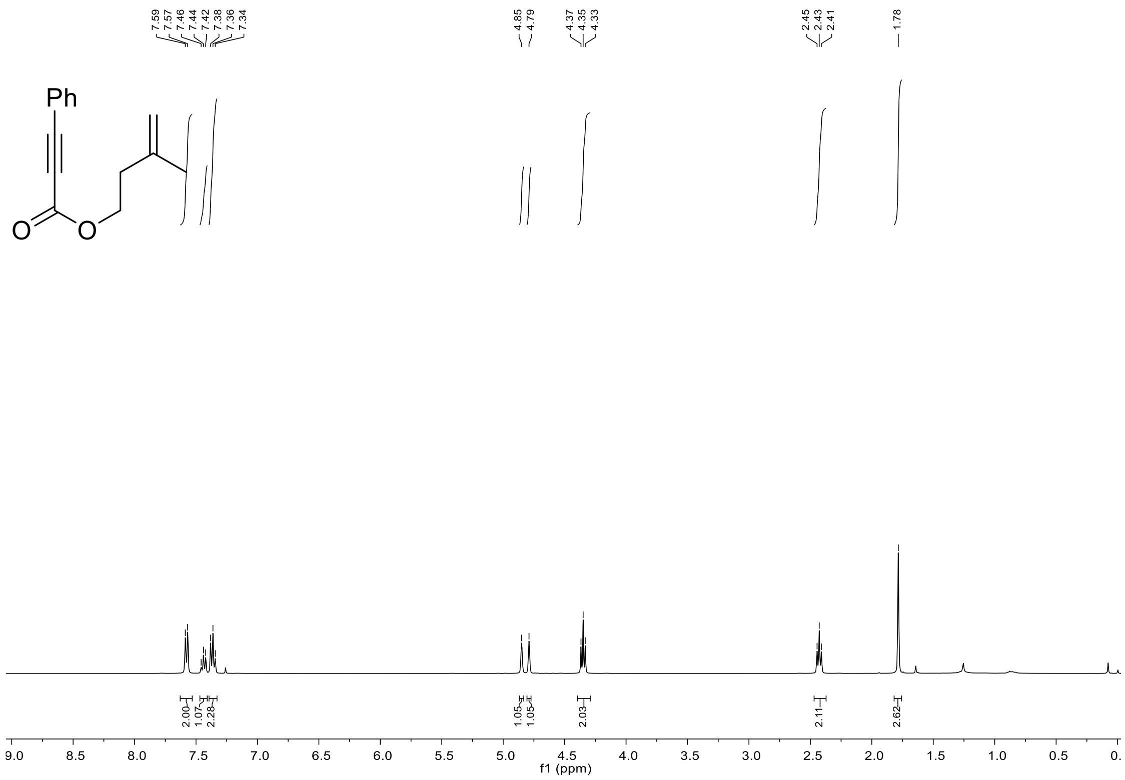

^{1}H NMR spectrum of **31a**

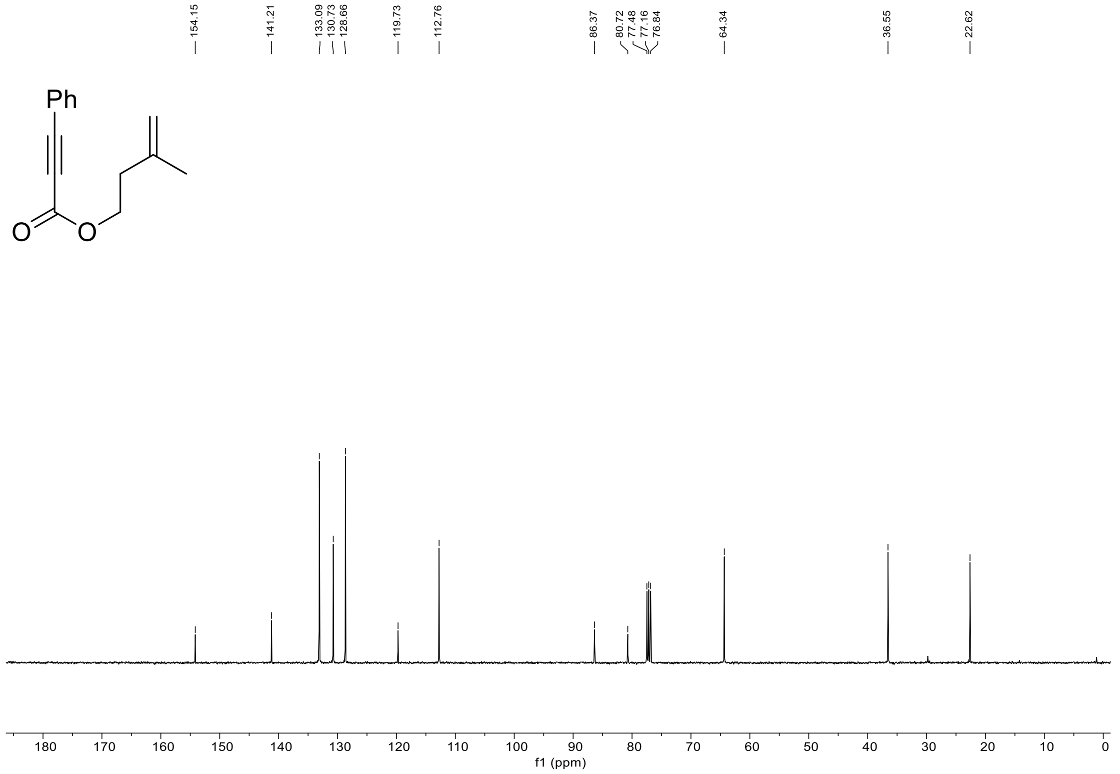

^{13}C NMR spectrum of **31a**

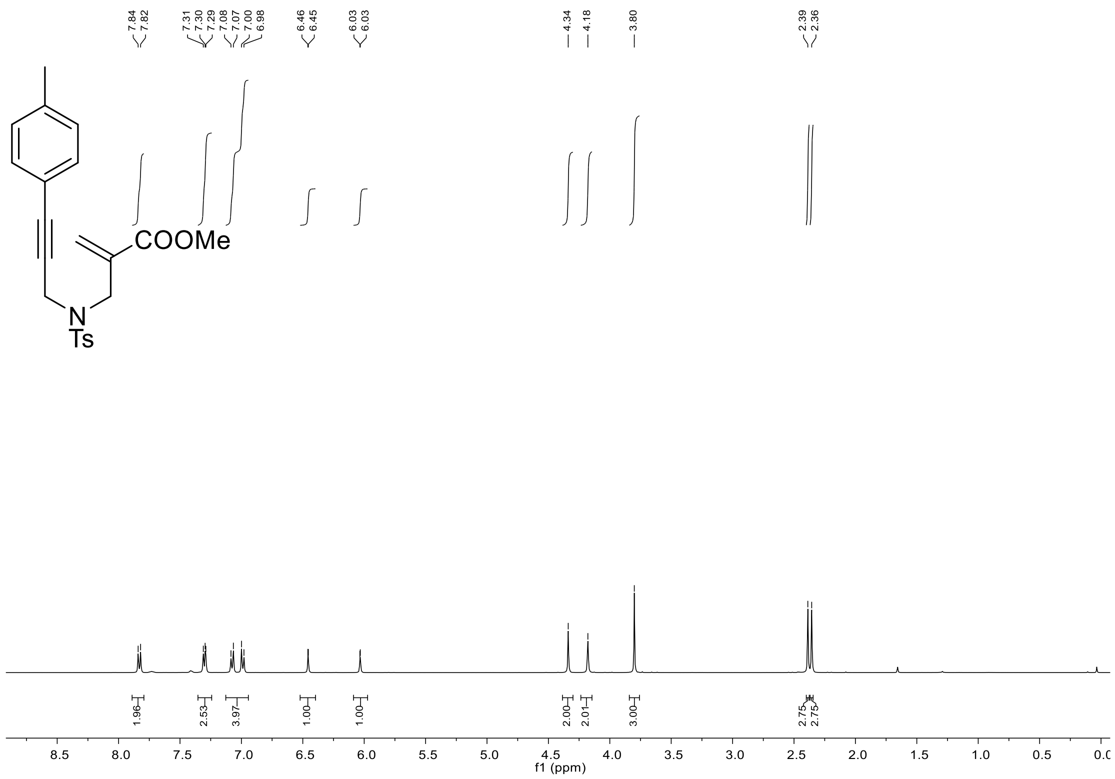

^{1}H NMR spectrum of **35a**

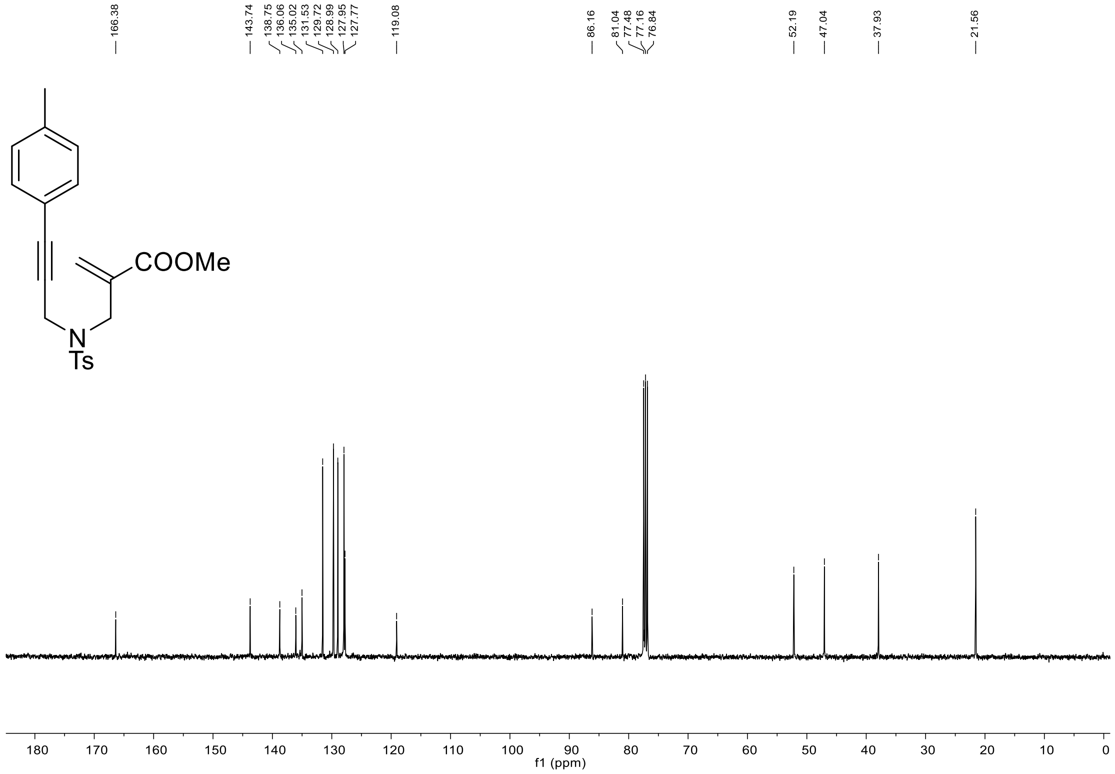

^{13}C NMR spectrum of **35a**

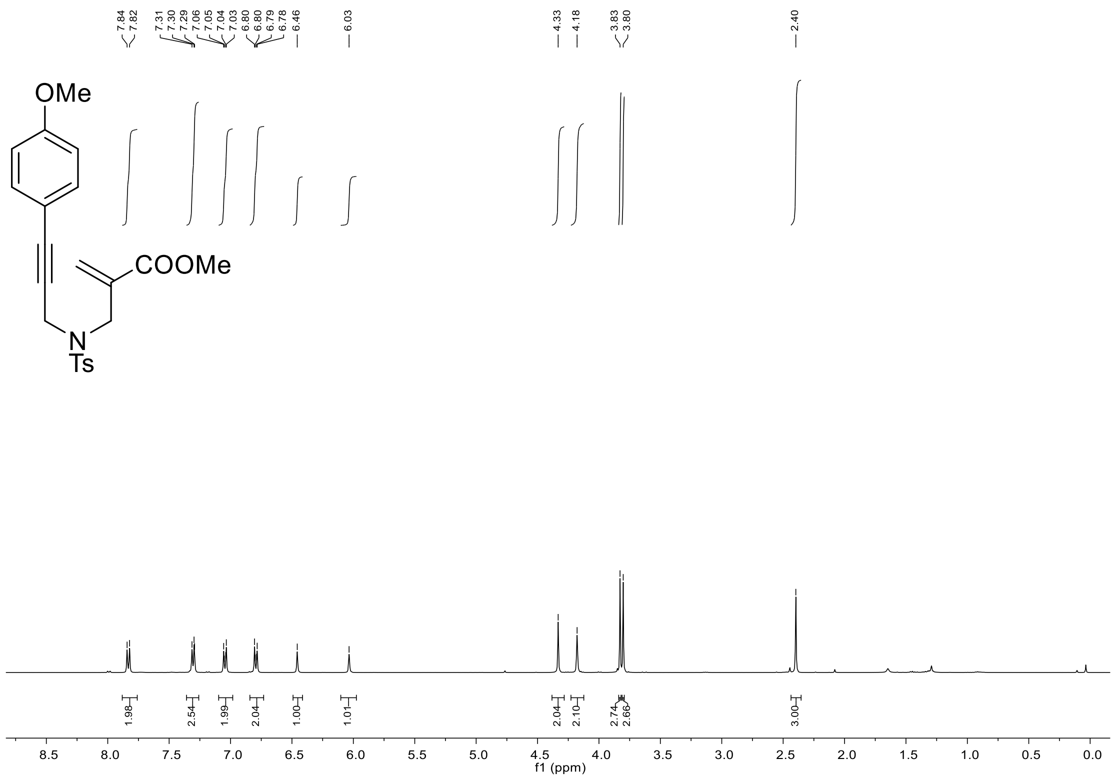

^{1}H NMR spectrum of **36a**

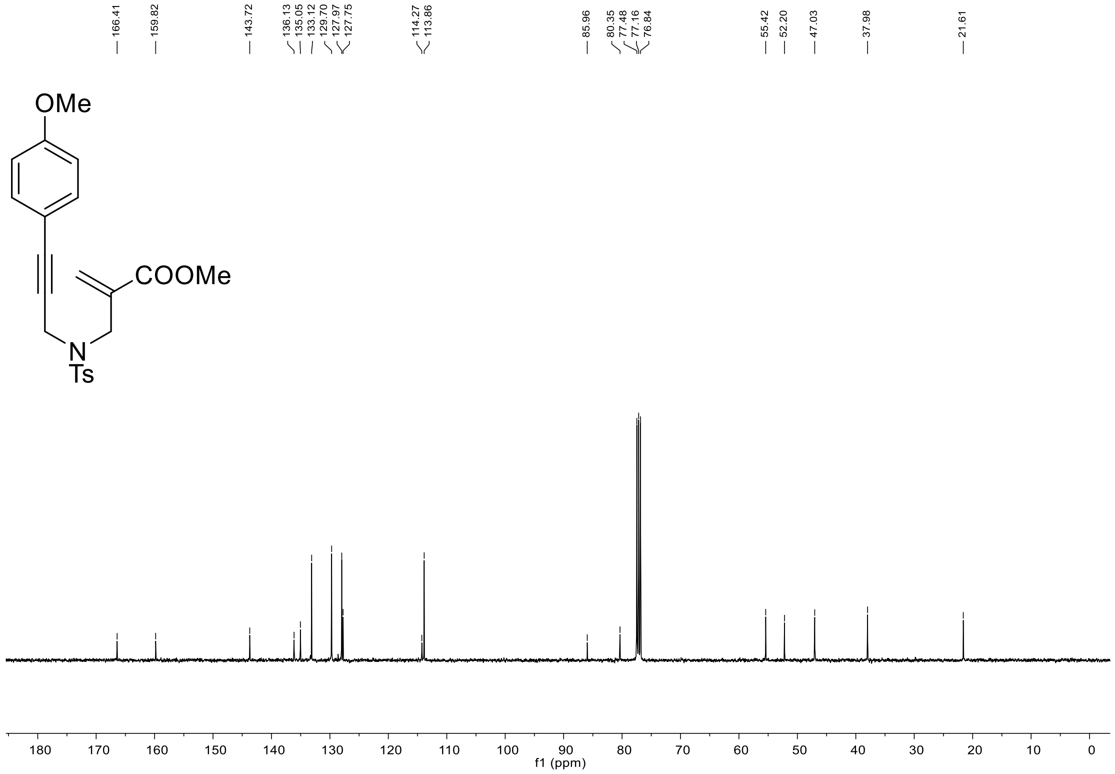

^{13}C NMR spectrum of **36a**

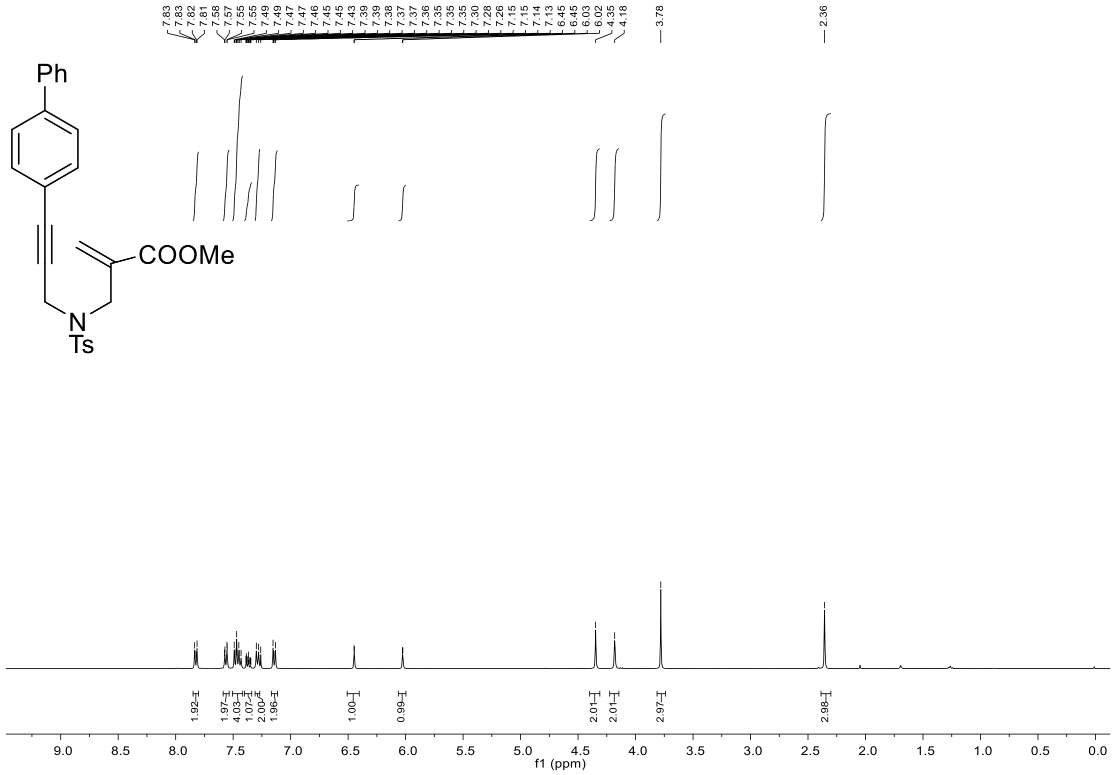

^{1}H NMR spectrum of **37a**

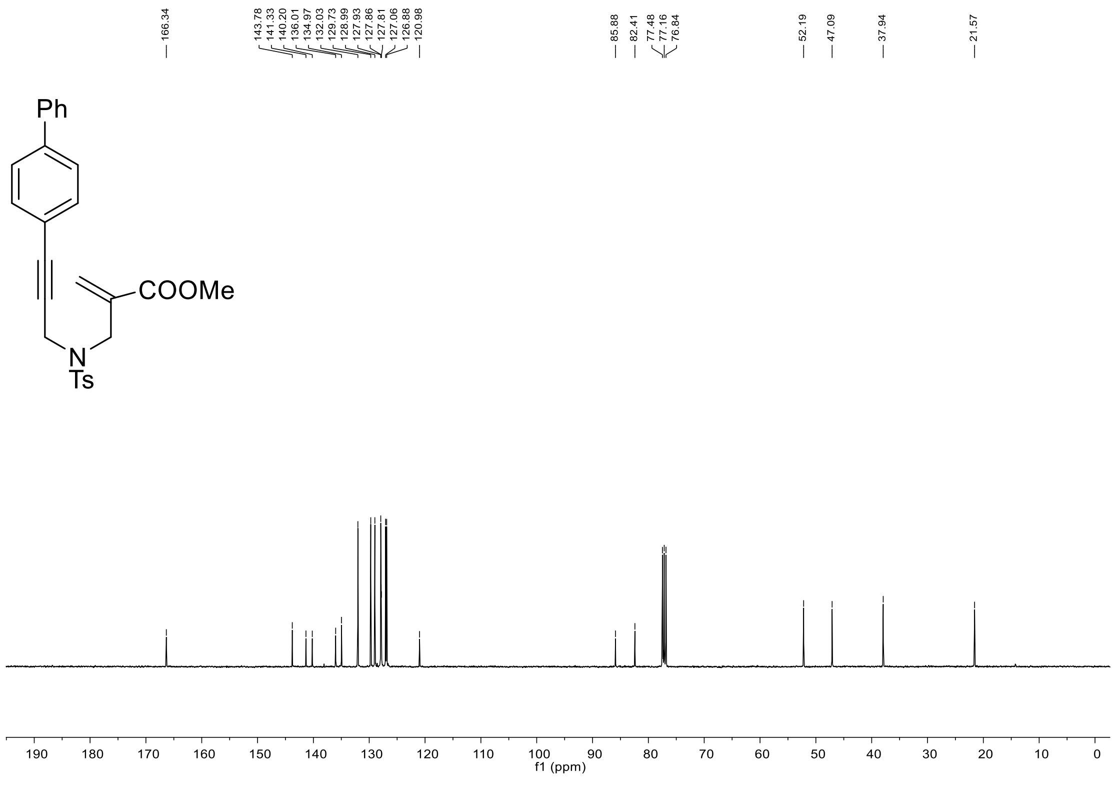

^{13}C NMR spectrum of 3**7a**

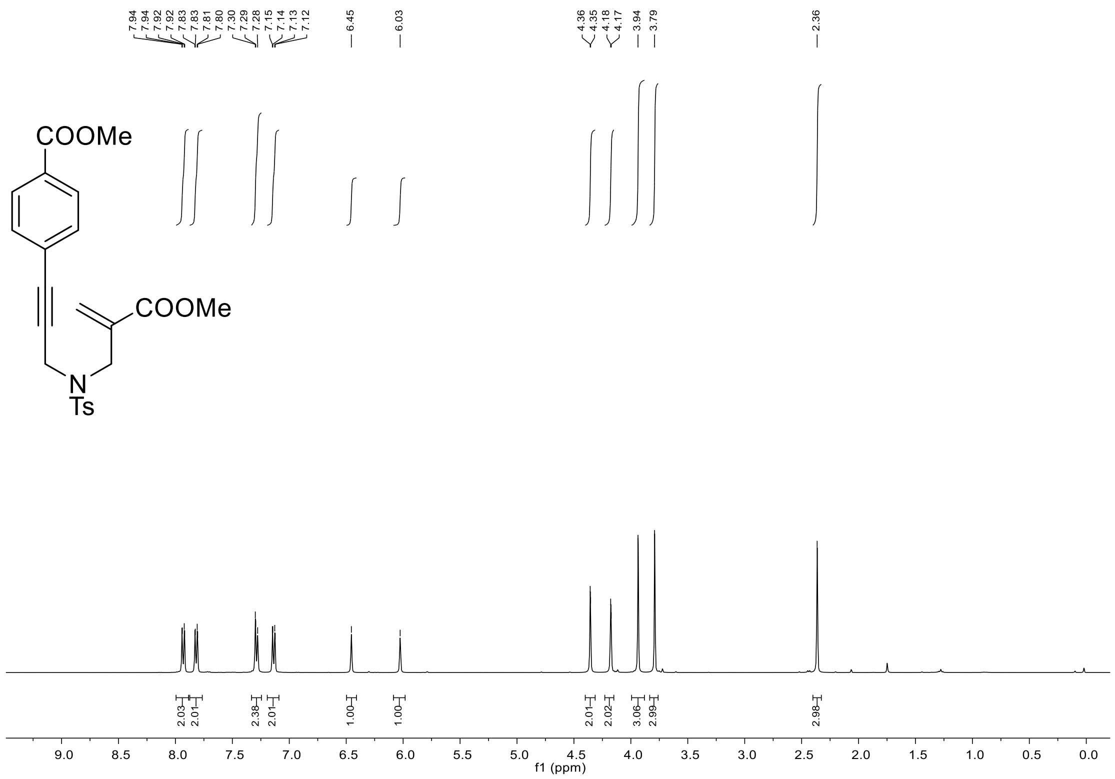

^{1}H NMR spectrum of **38a**

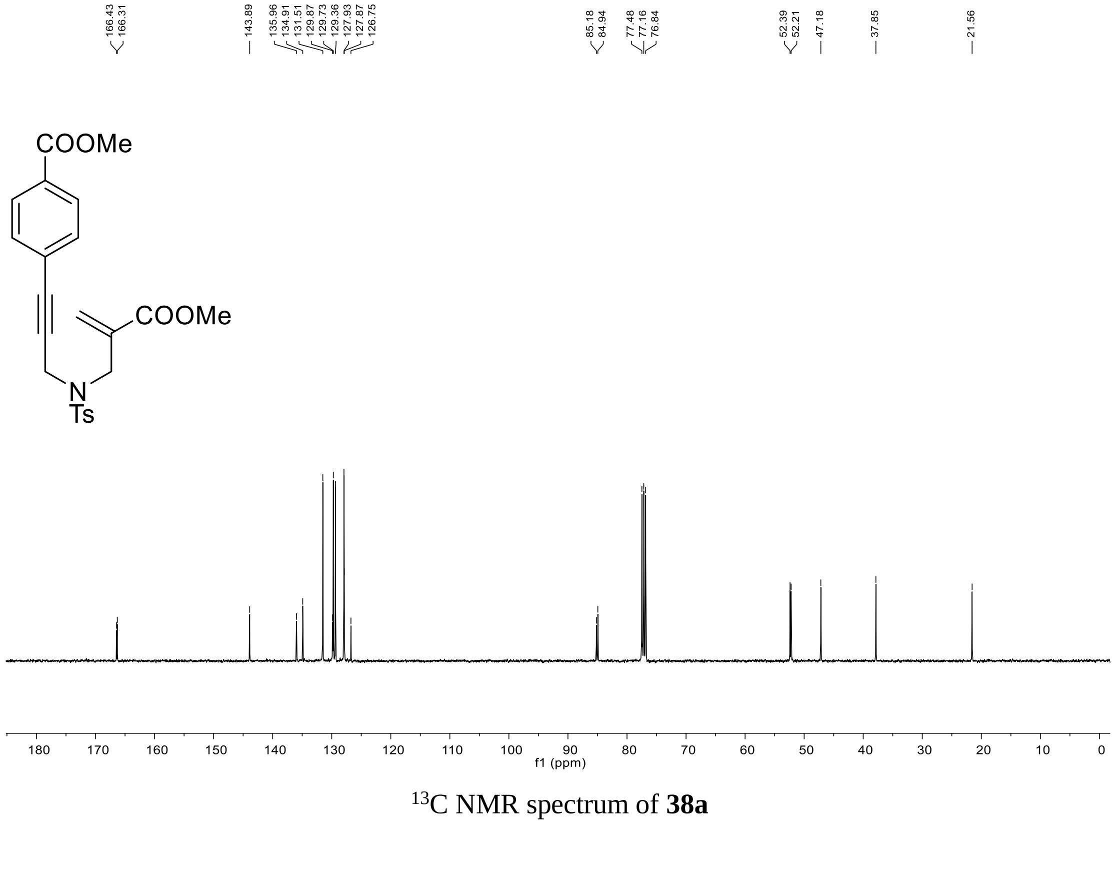

^{13}C NMR spectrum of **38a**

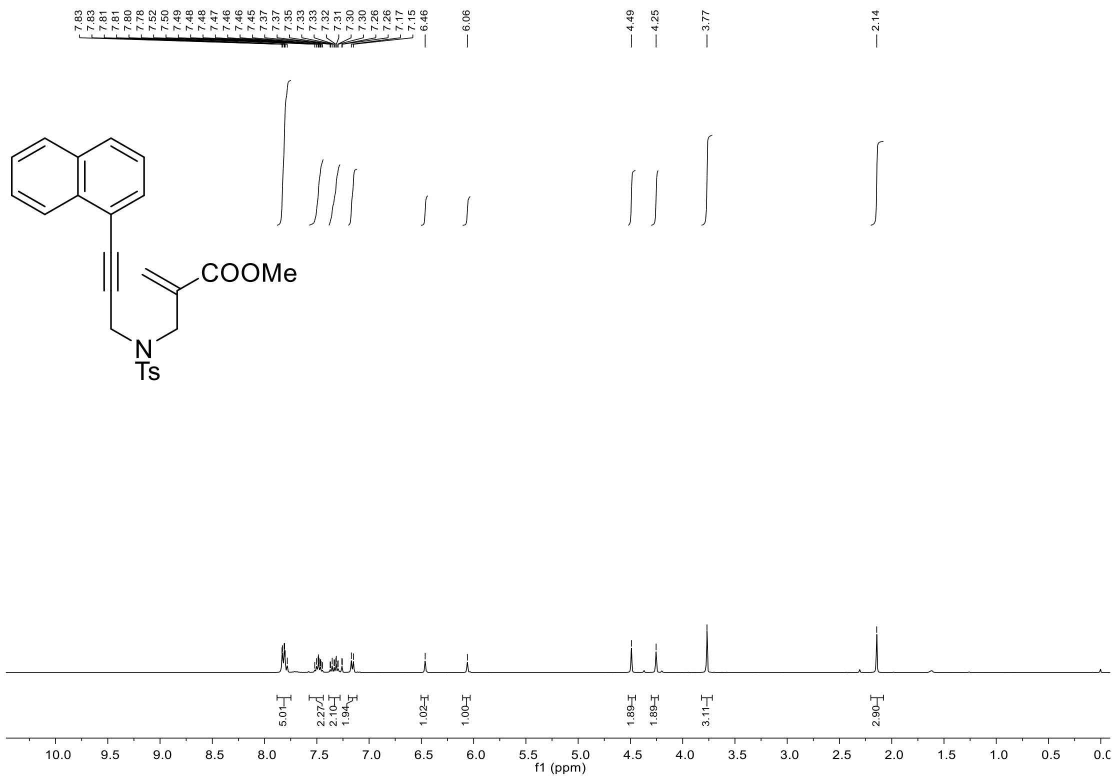

^{1}H NMR spectrum of **39a**

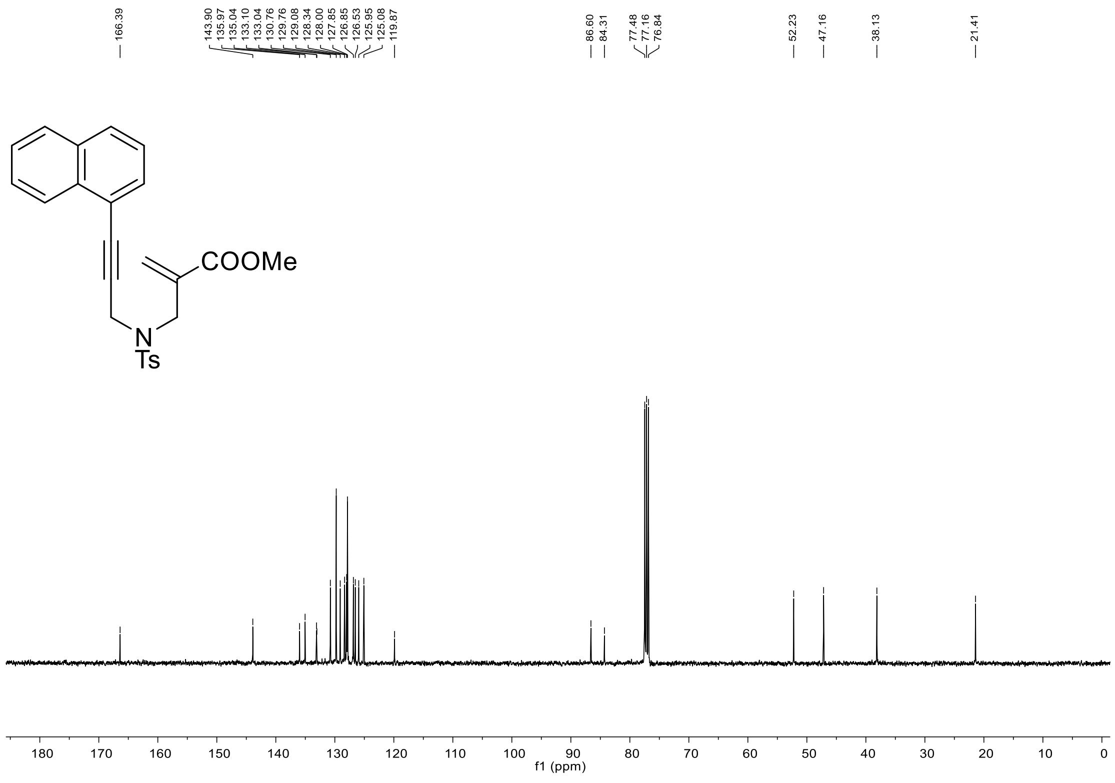

^{13}C NMR spectrum of **39a**

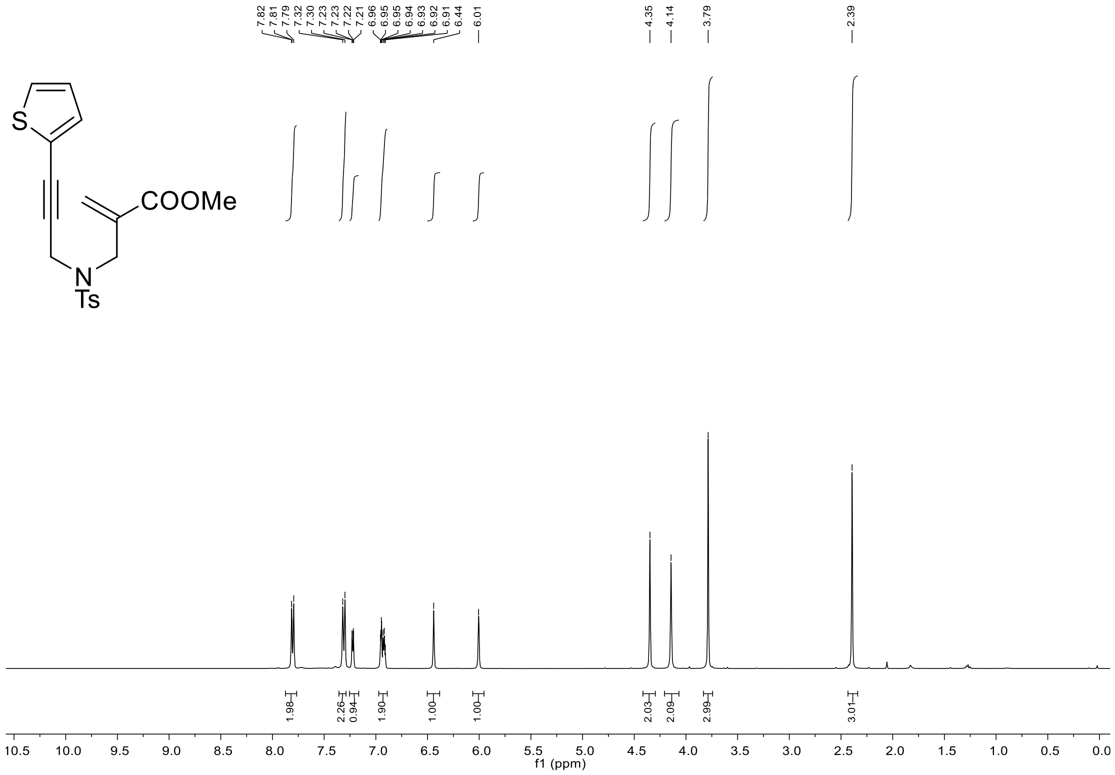

^{1}H NMR spectrum of **40a**

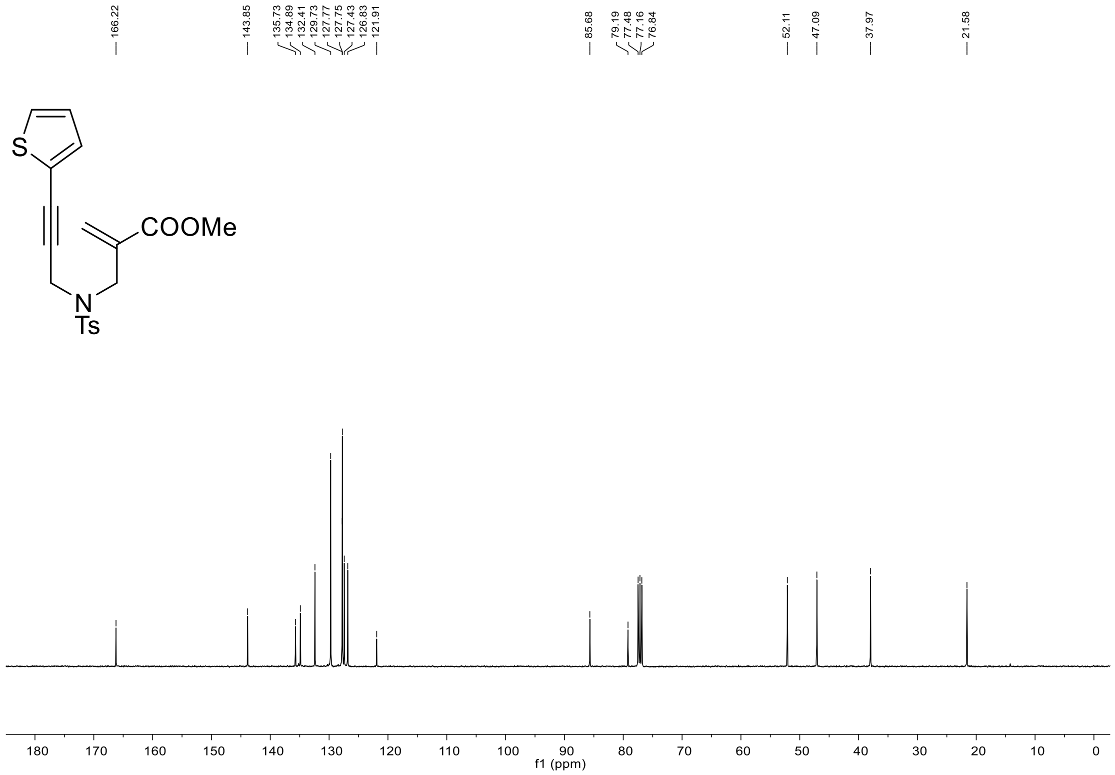

^{13}C NMR spectrum of **40a**

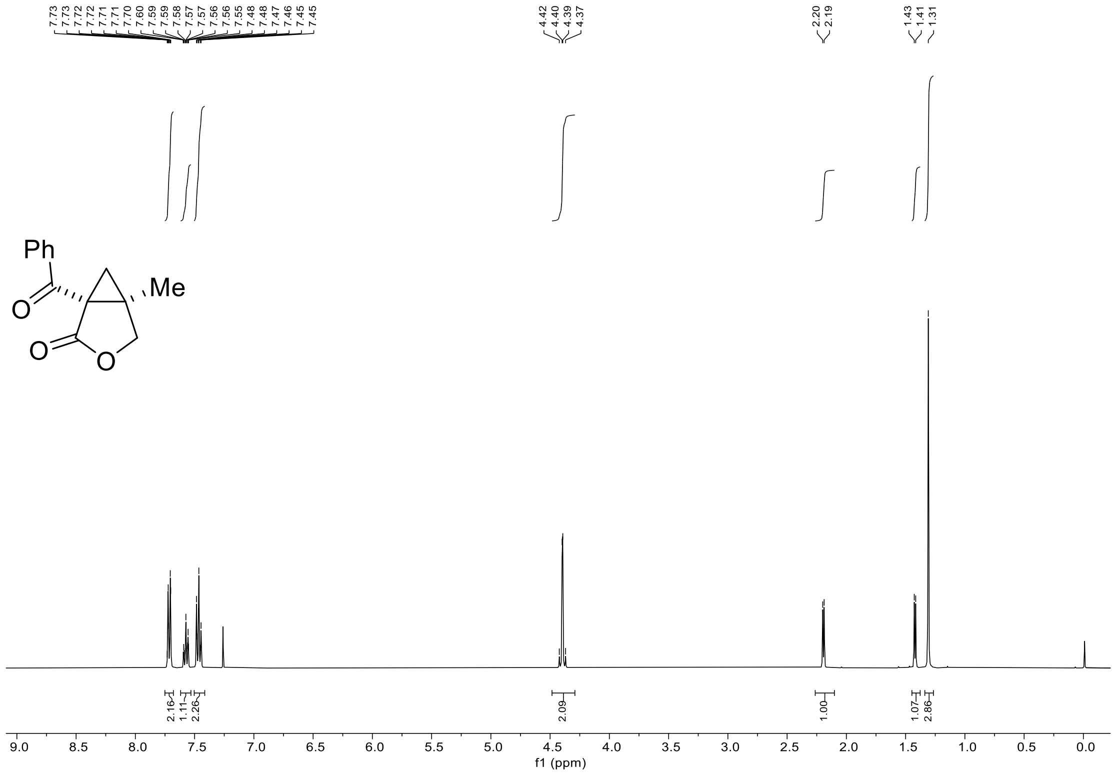

^{1}H NMR spectrum of **1**

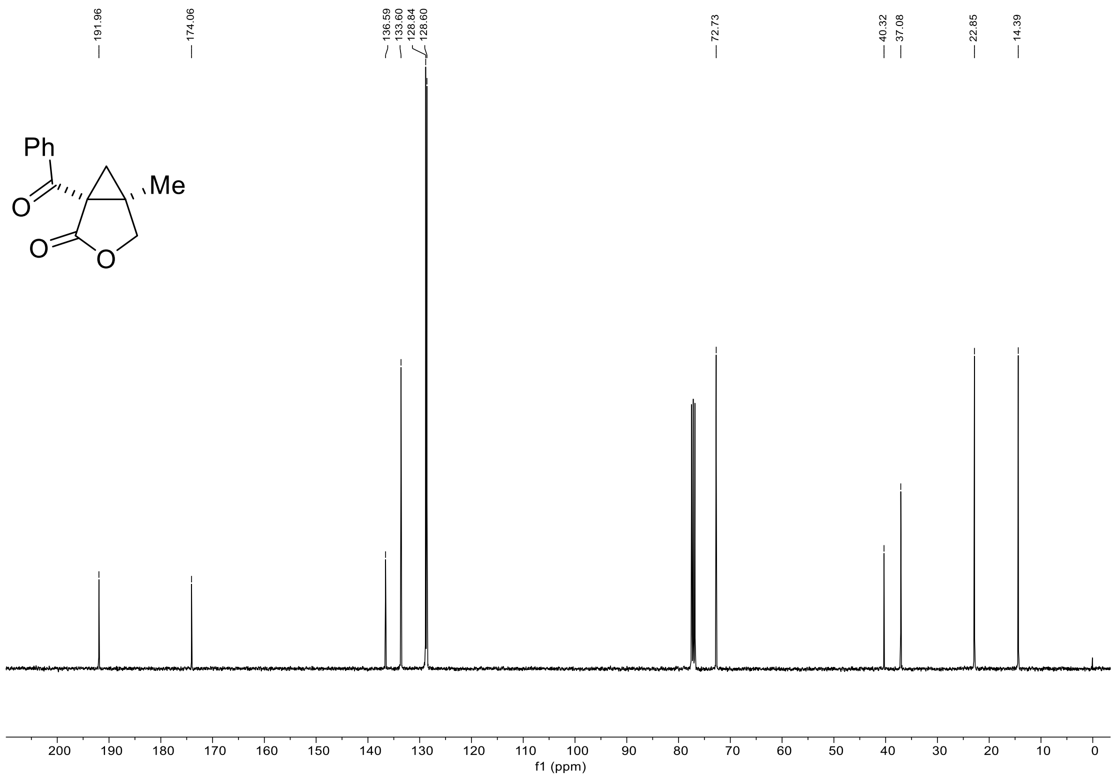

^{13}C NMR spectrum of **1**

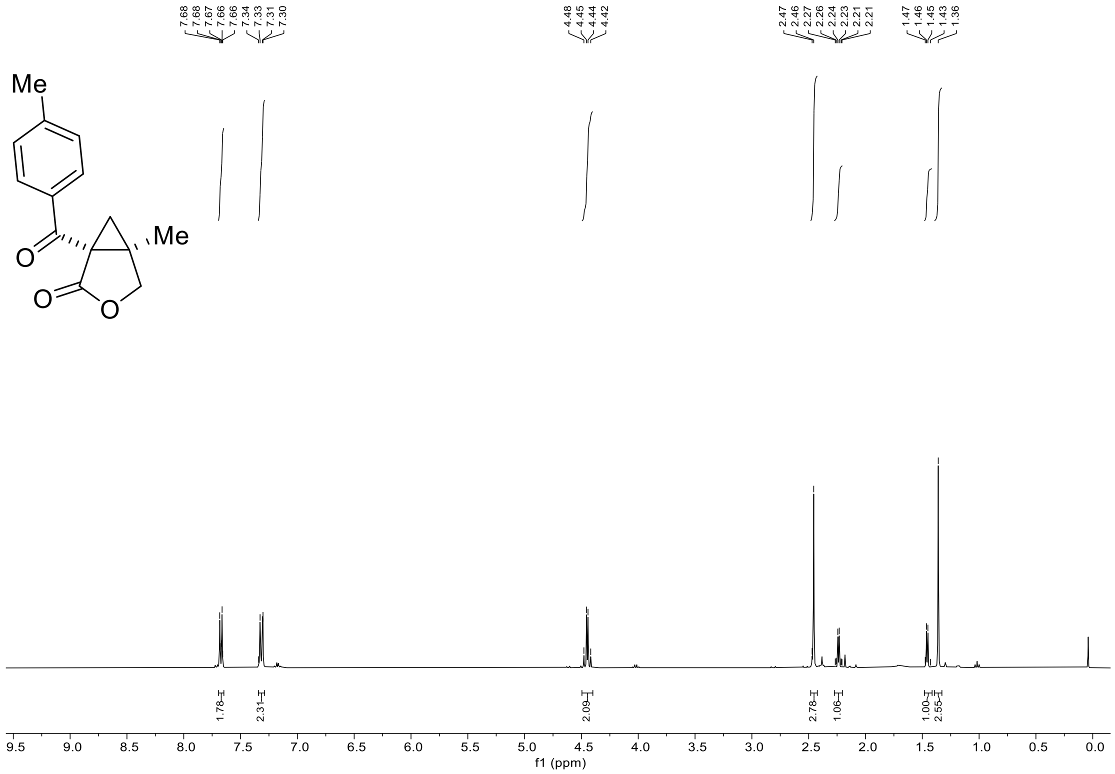

^{1}H NMR spectrum of **2**

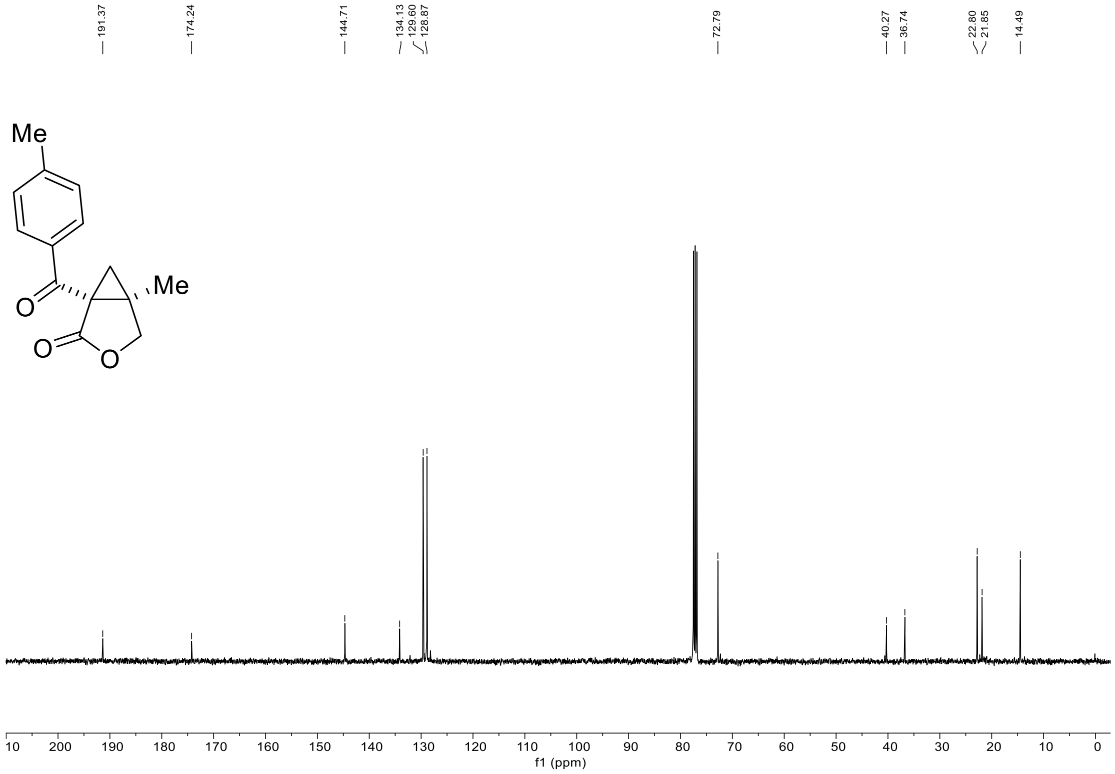

^{13}C NMR spectrum of **2**

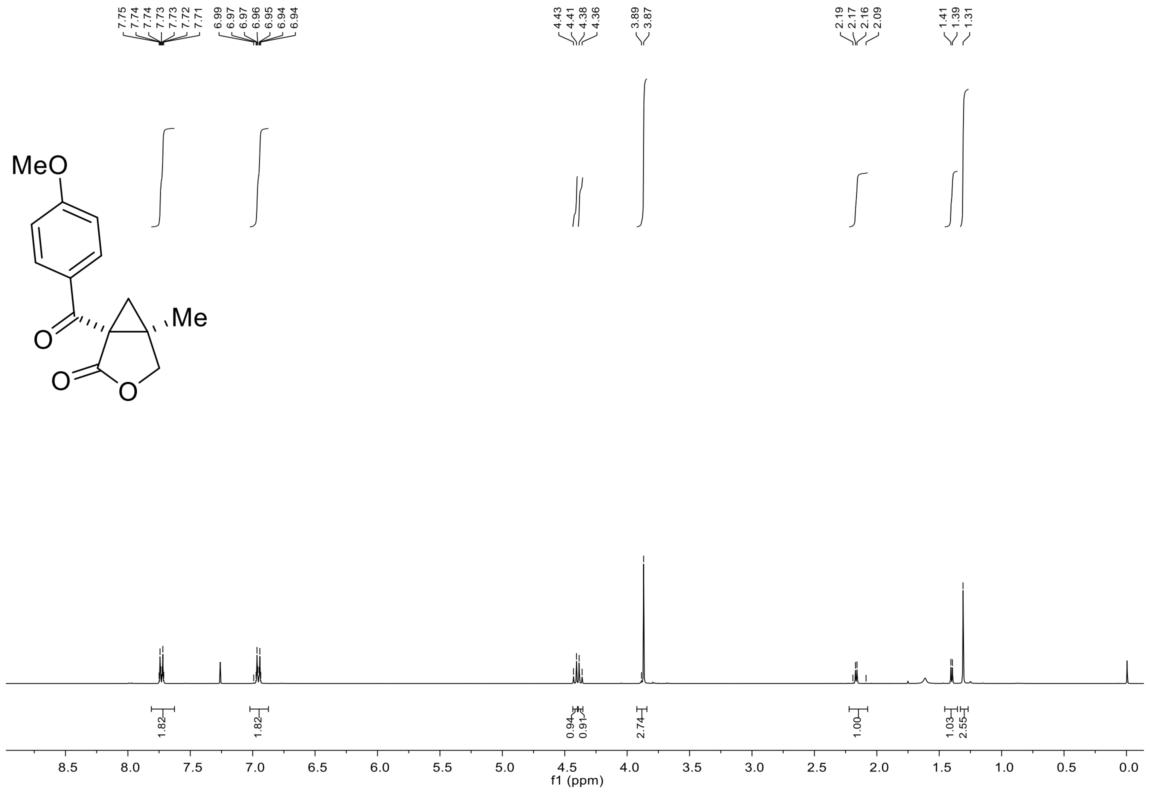

^{1}H NMR spectrum of **3**

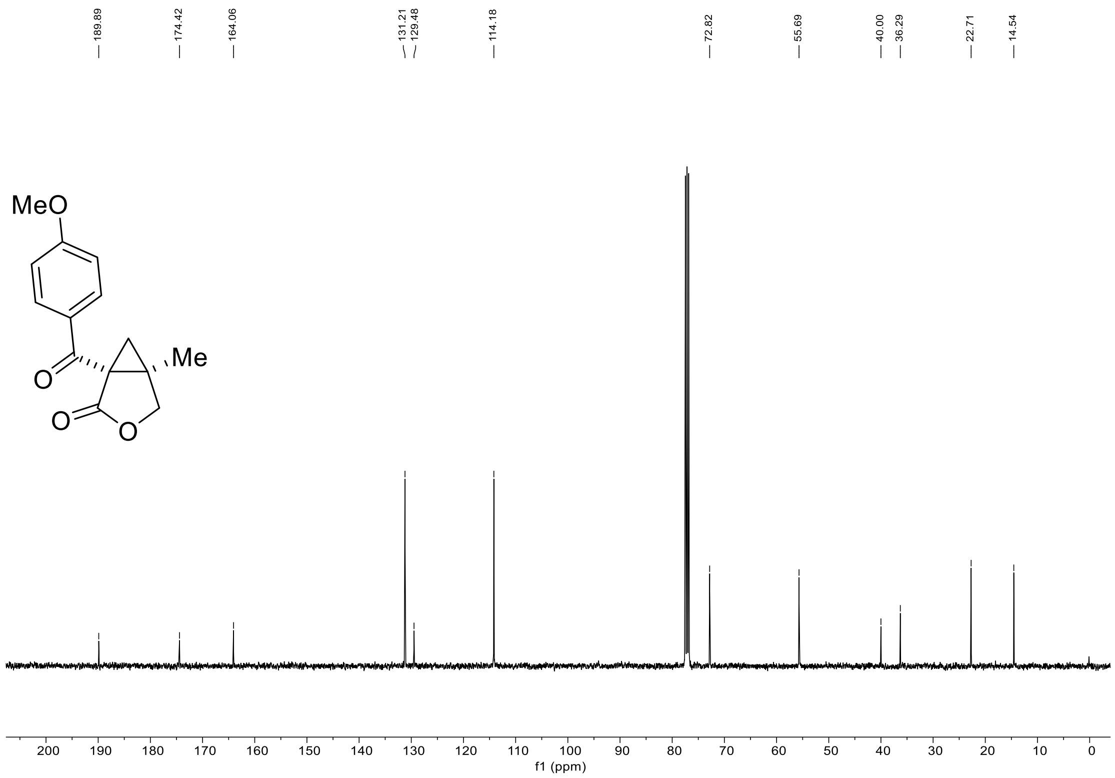

^{13}C NMR spectrum of **3**

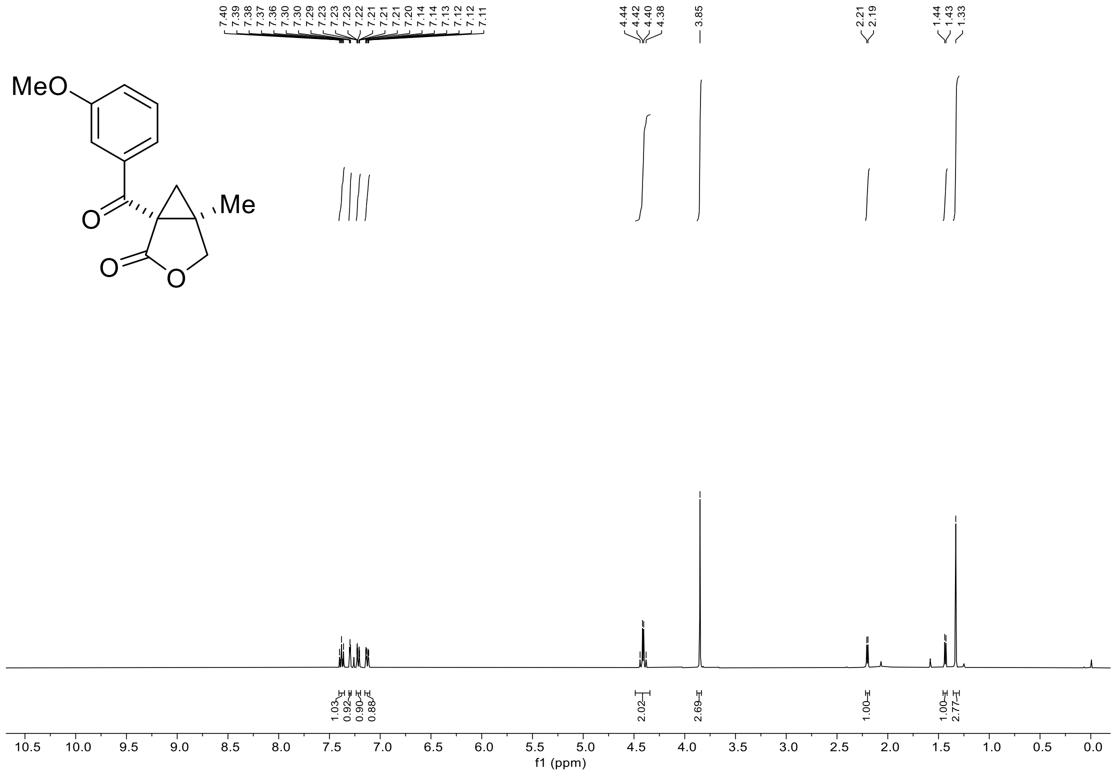

^{1}H NMR spectrum of **4**

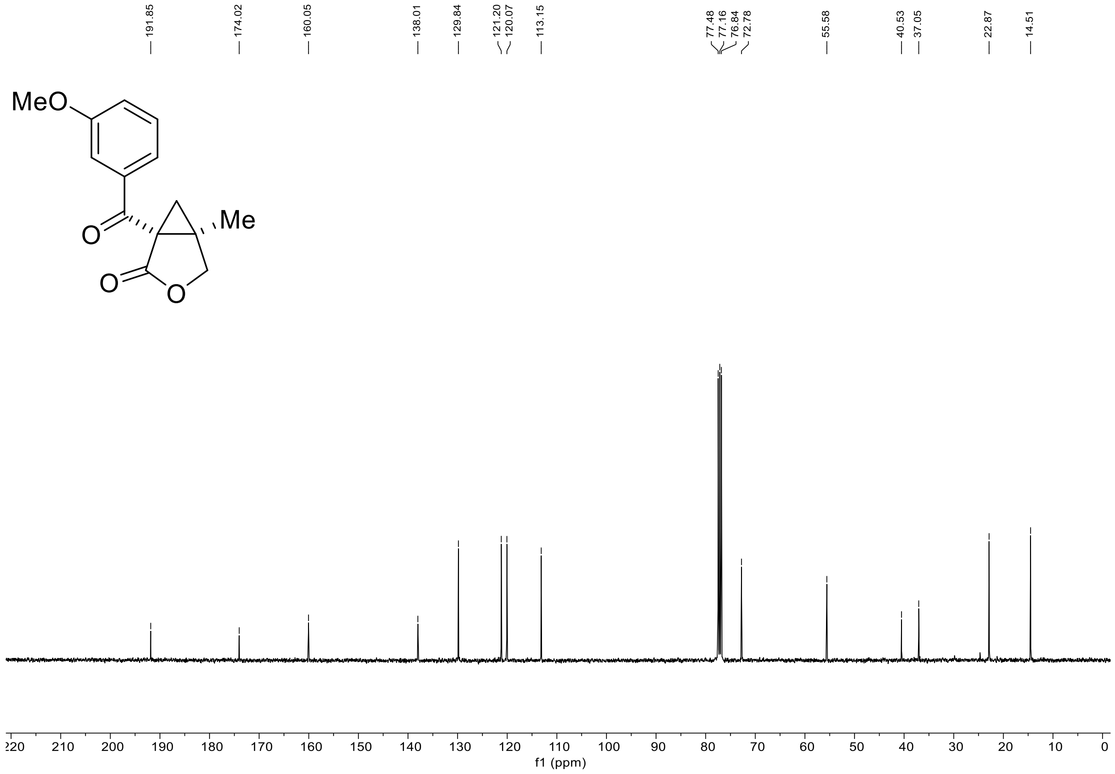

^{13}C NMR spectrum of **4**

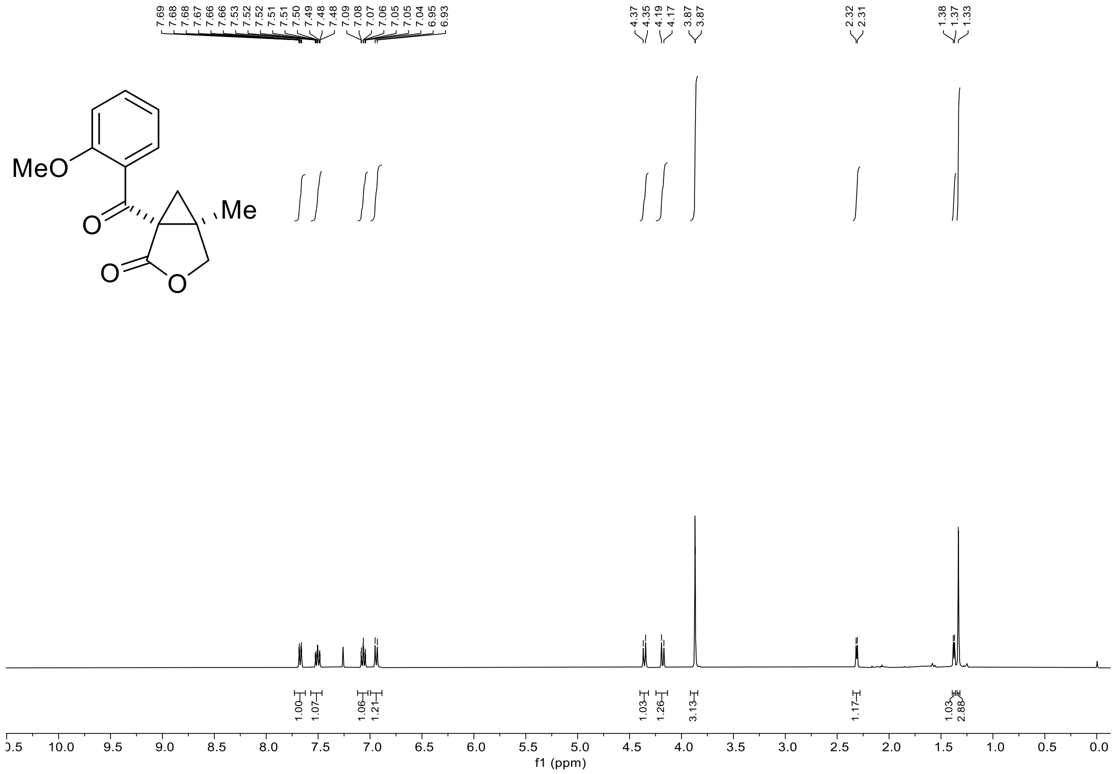

^{1}H NMR spectrum of **5**

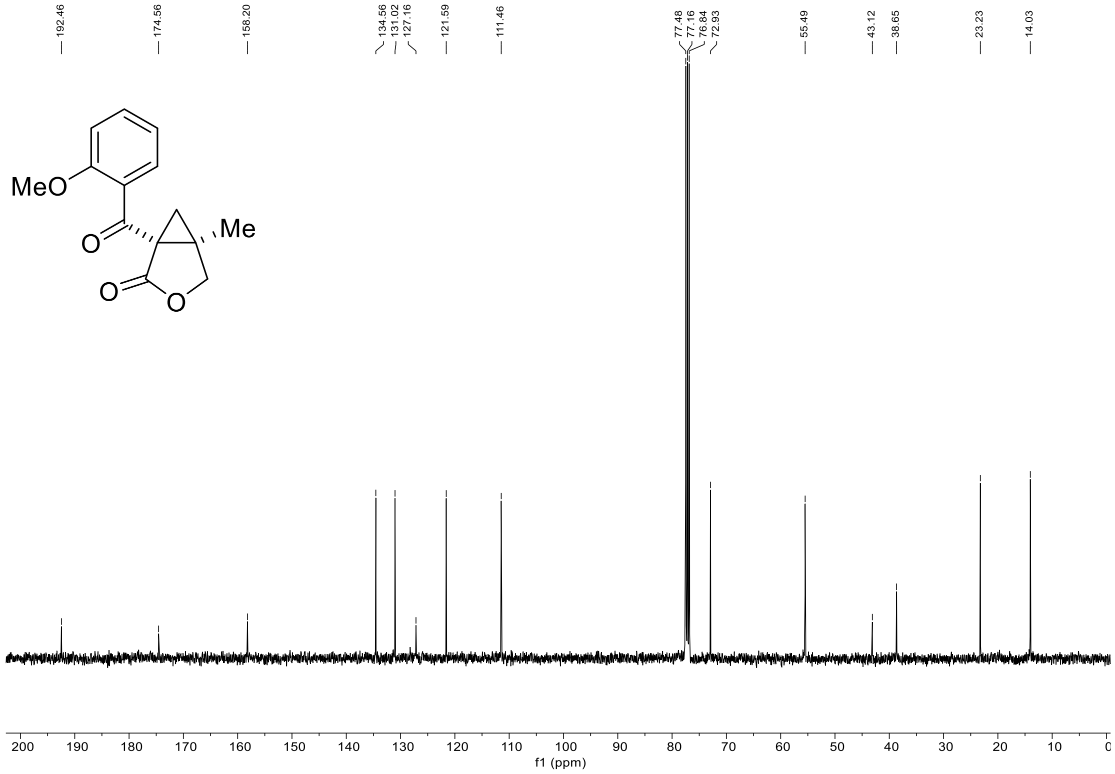

^{13}C NMR spectrum of **5**

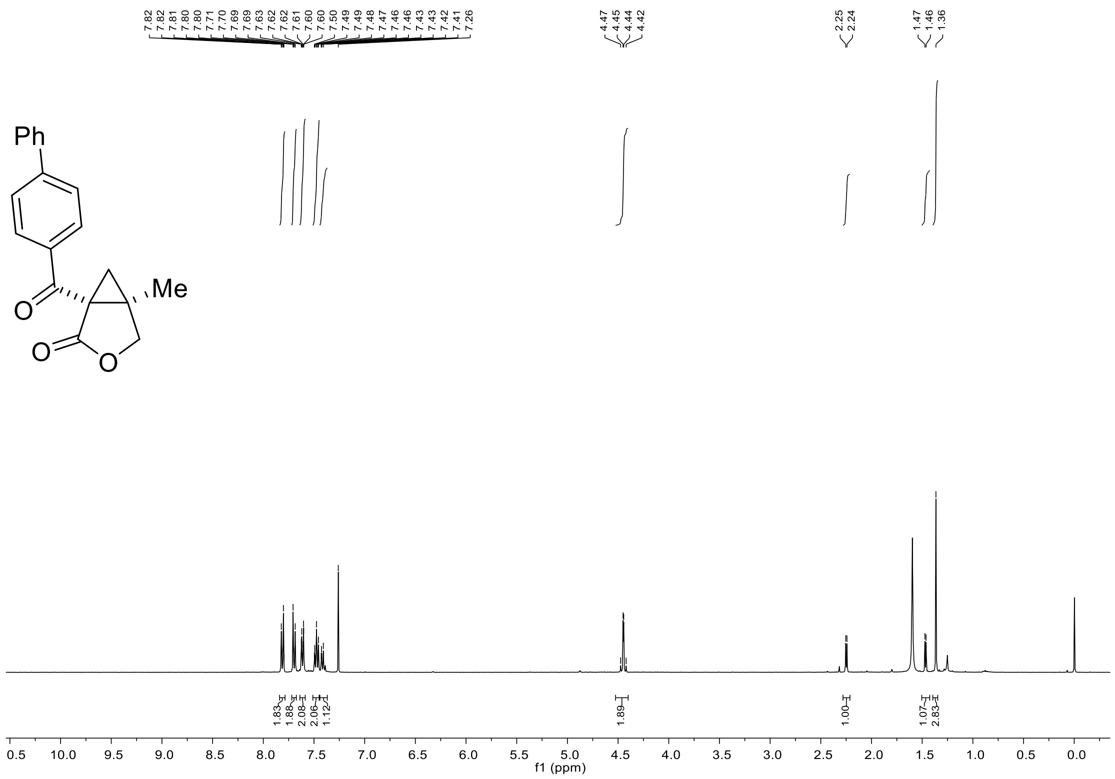

^{1}H NMR spectrum of **6**

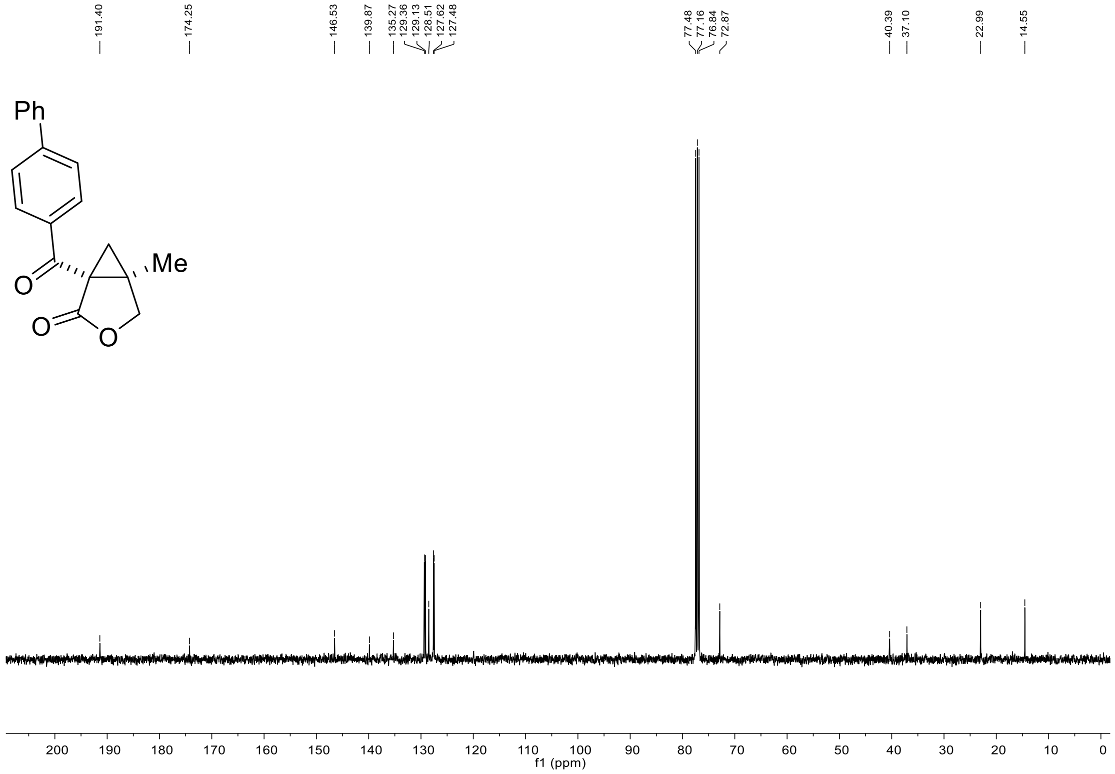

^{13}C NMR spectrum of **6**

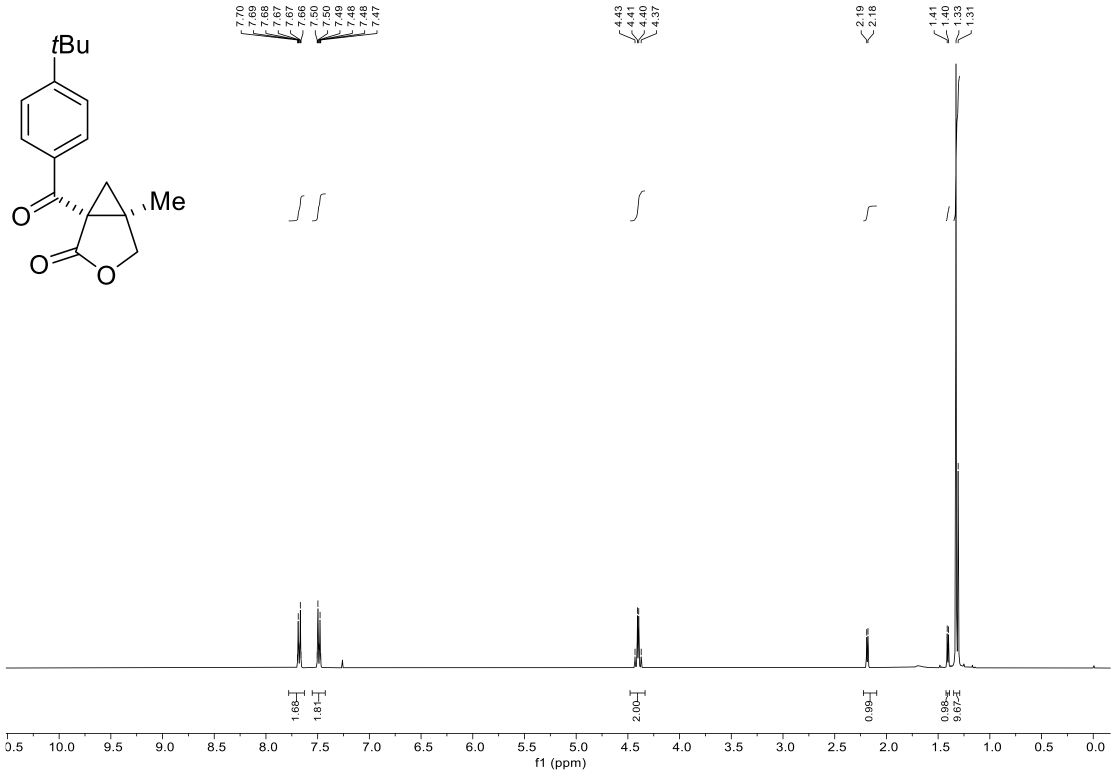

^{1}H NMR spectrum of **7**

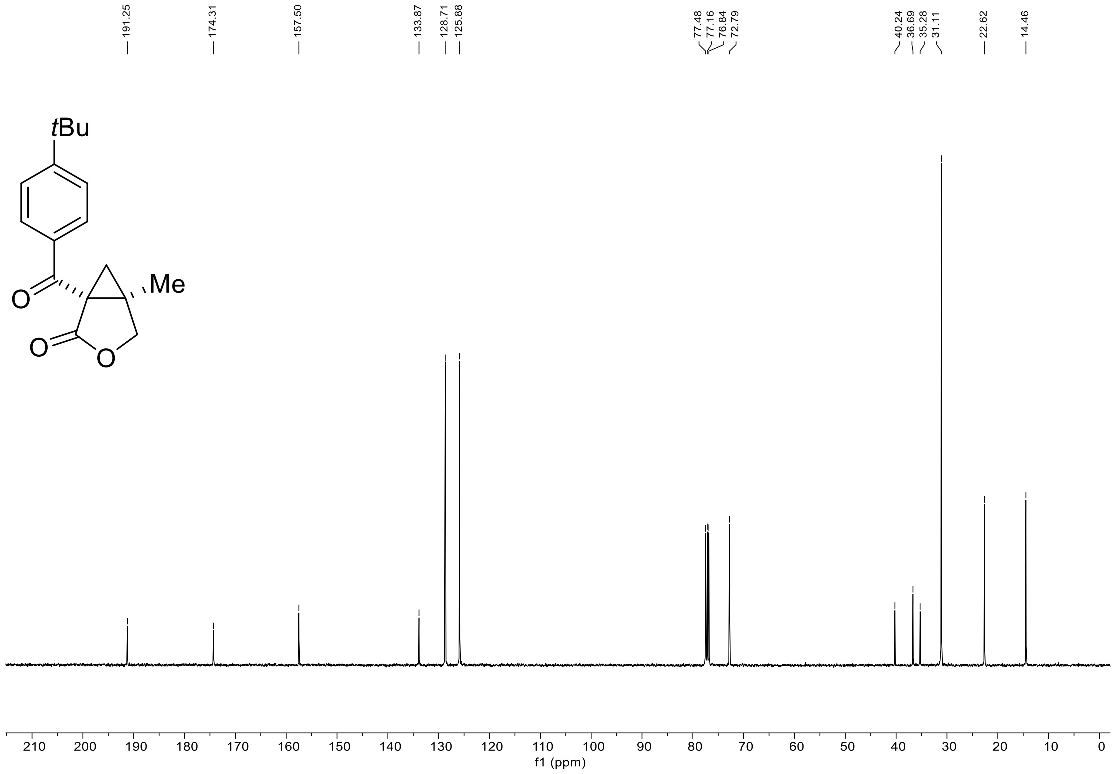

^{13}C NMR spectrum of **7**

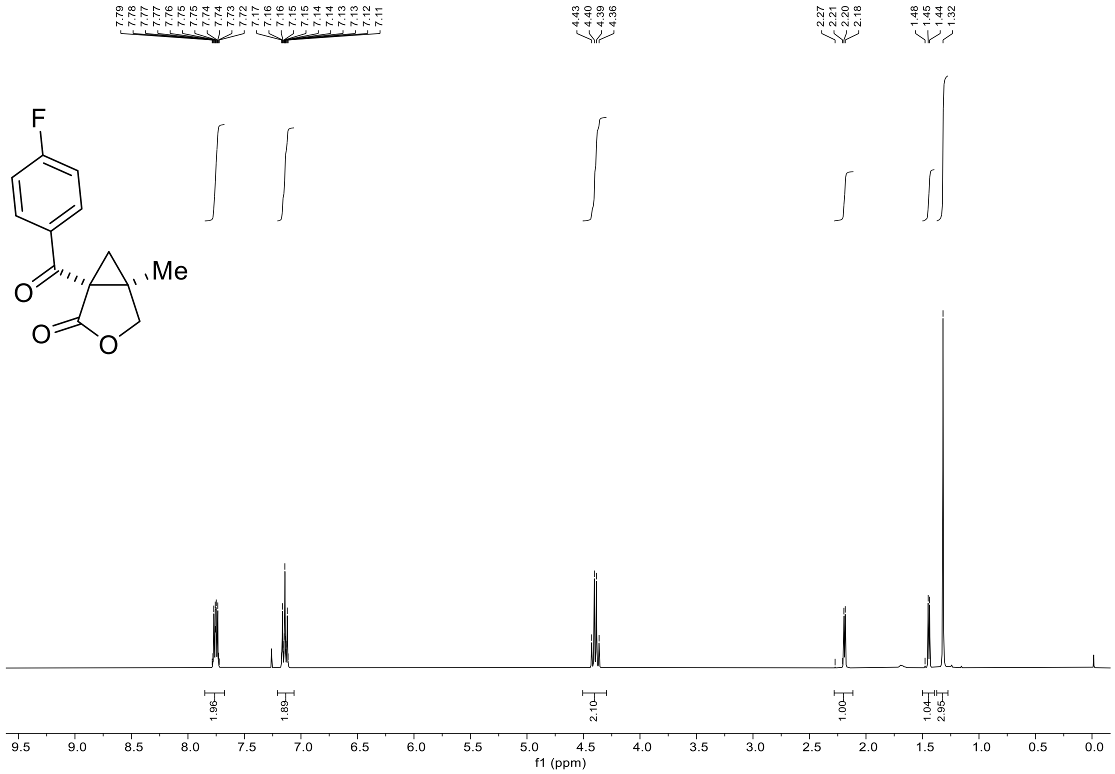

^{1}H NMR spectrum of **8**

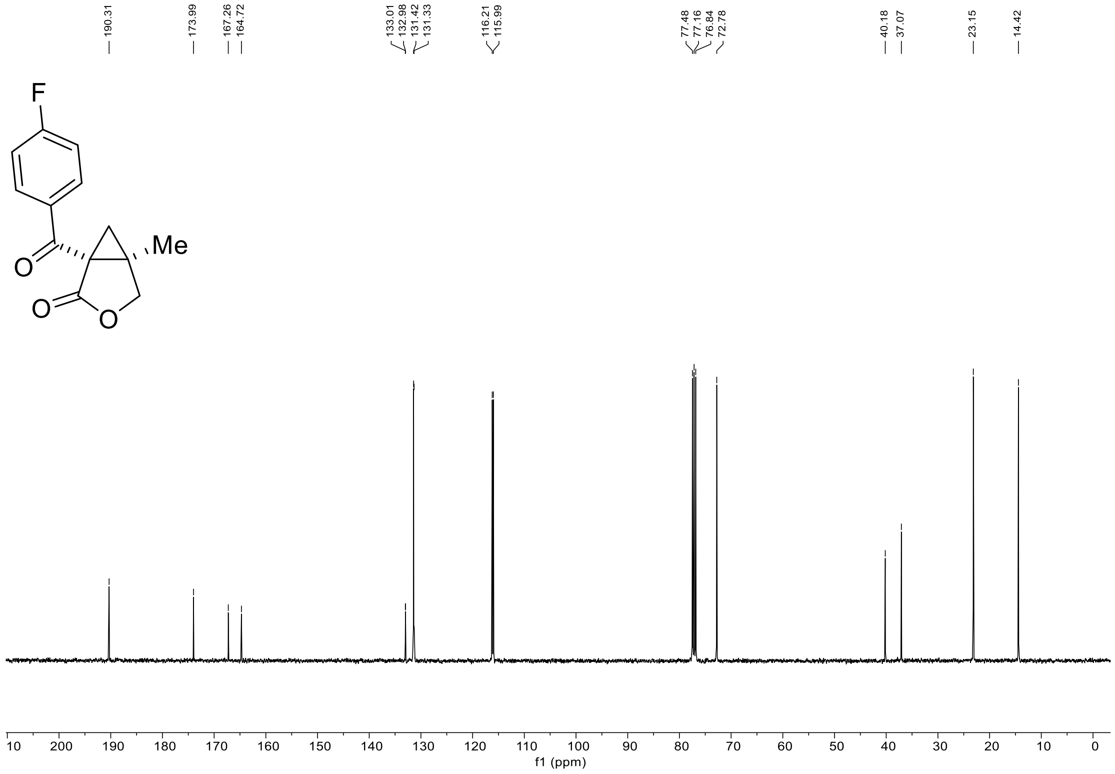

^{13}C NMR spectrum of **8**

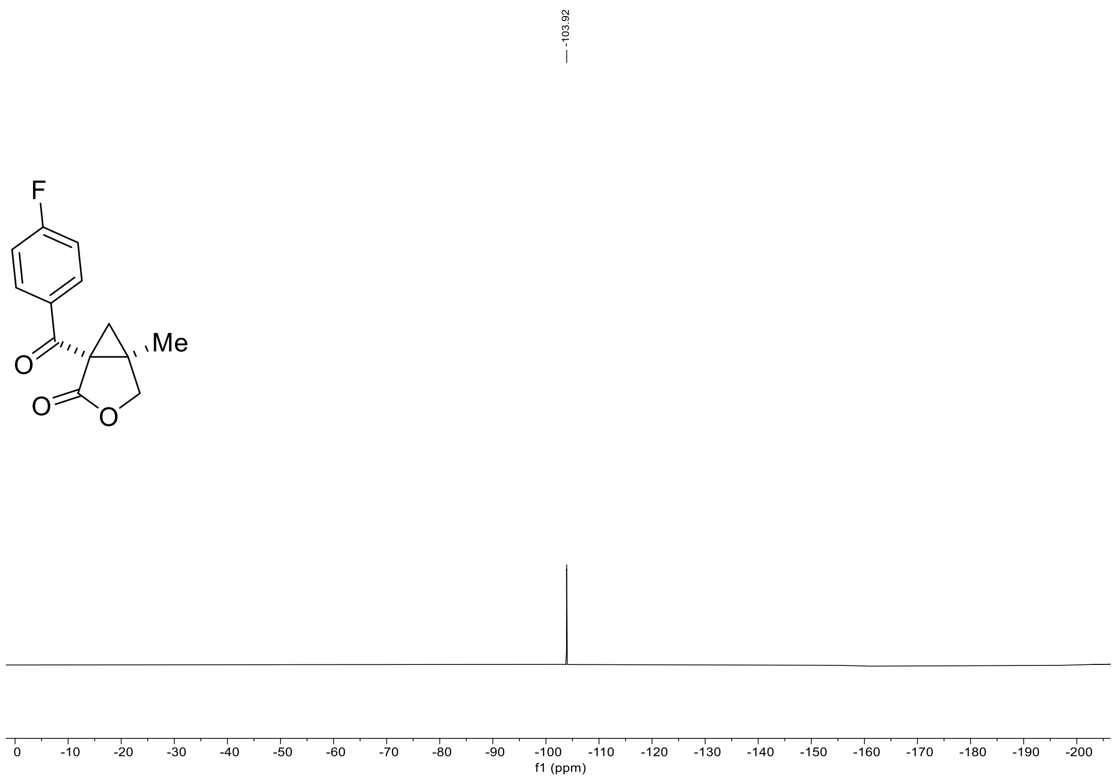

^{19}F NMR spectrum of **8**

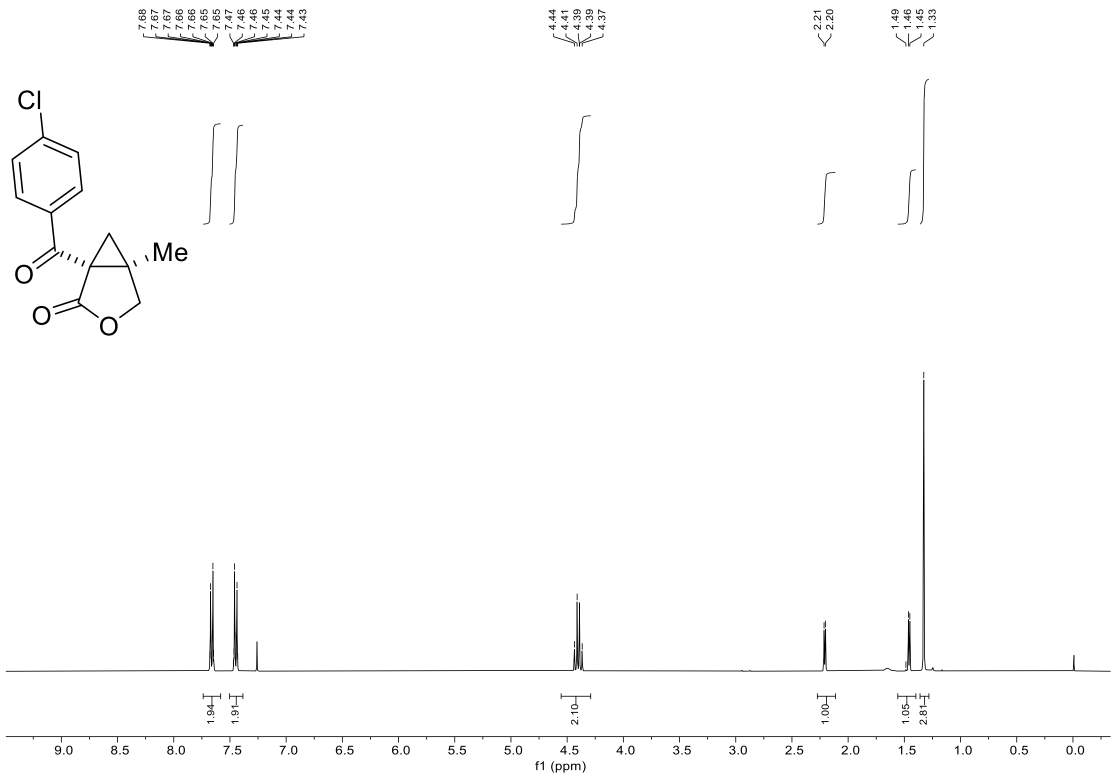

^{1}H NMR spectrum of **9**

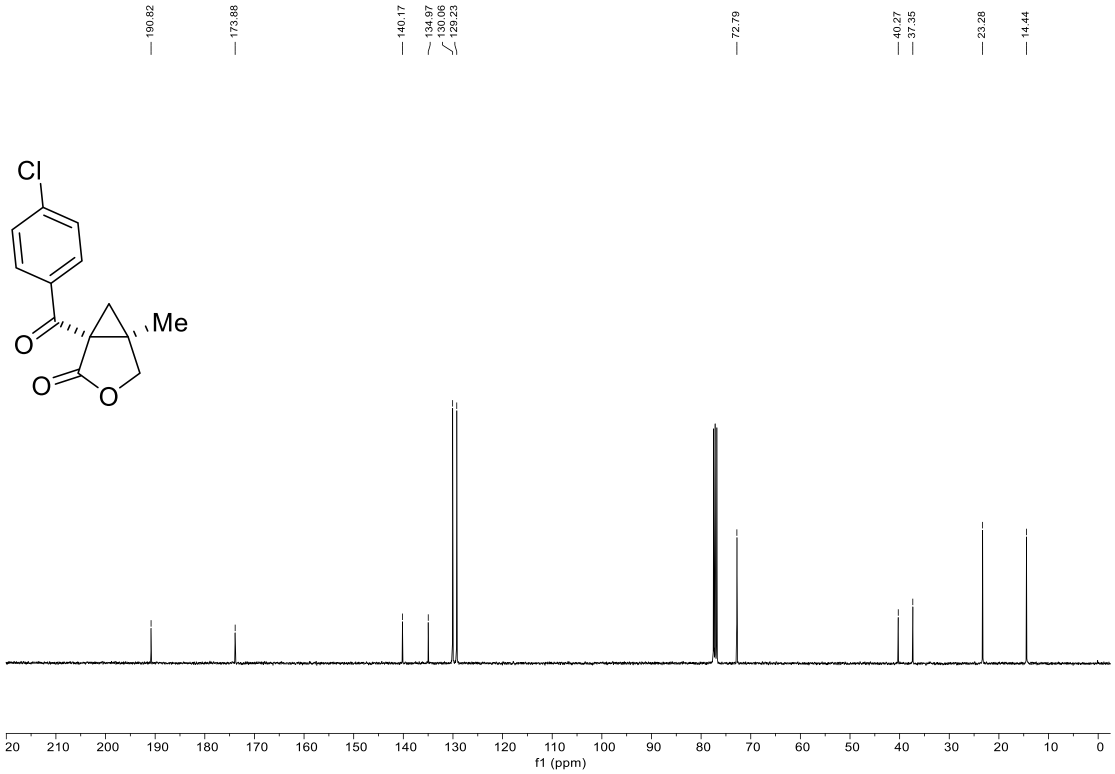

^{13}C NMR spectrum of **9**

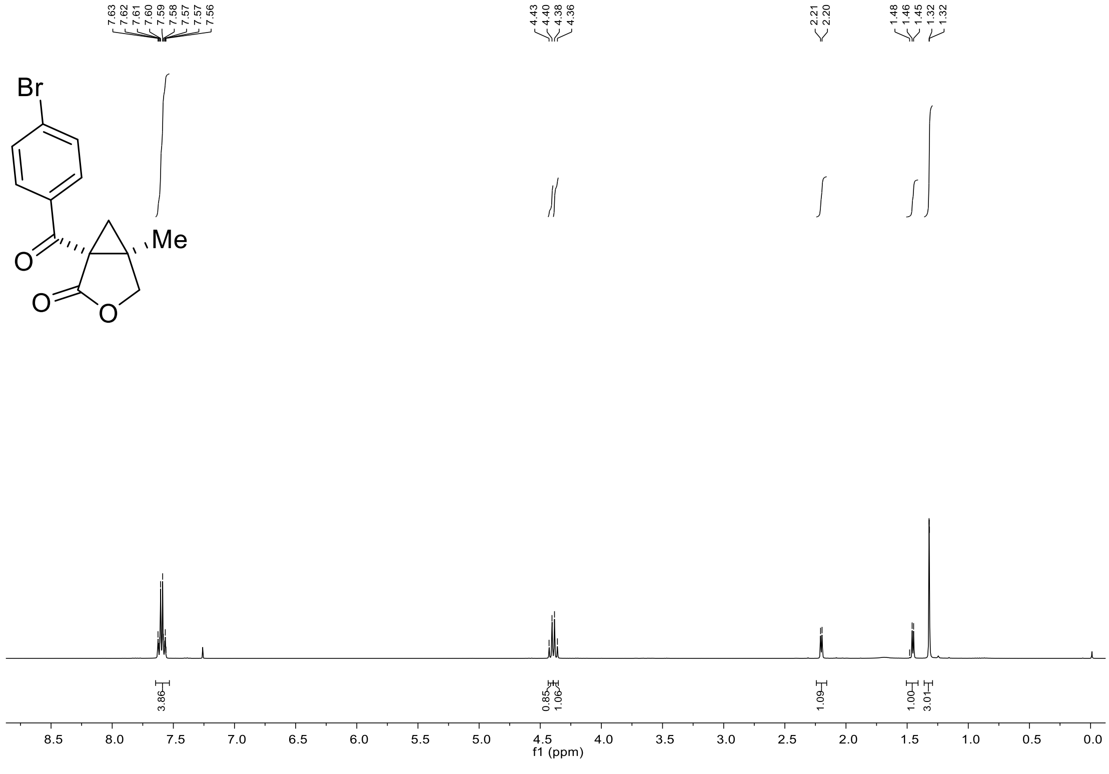

^{1}H NMR spectrum of **10**

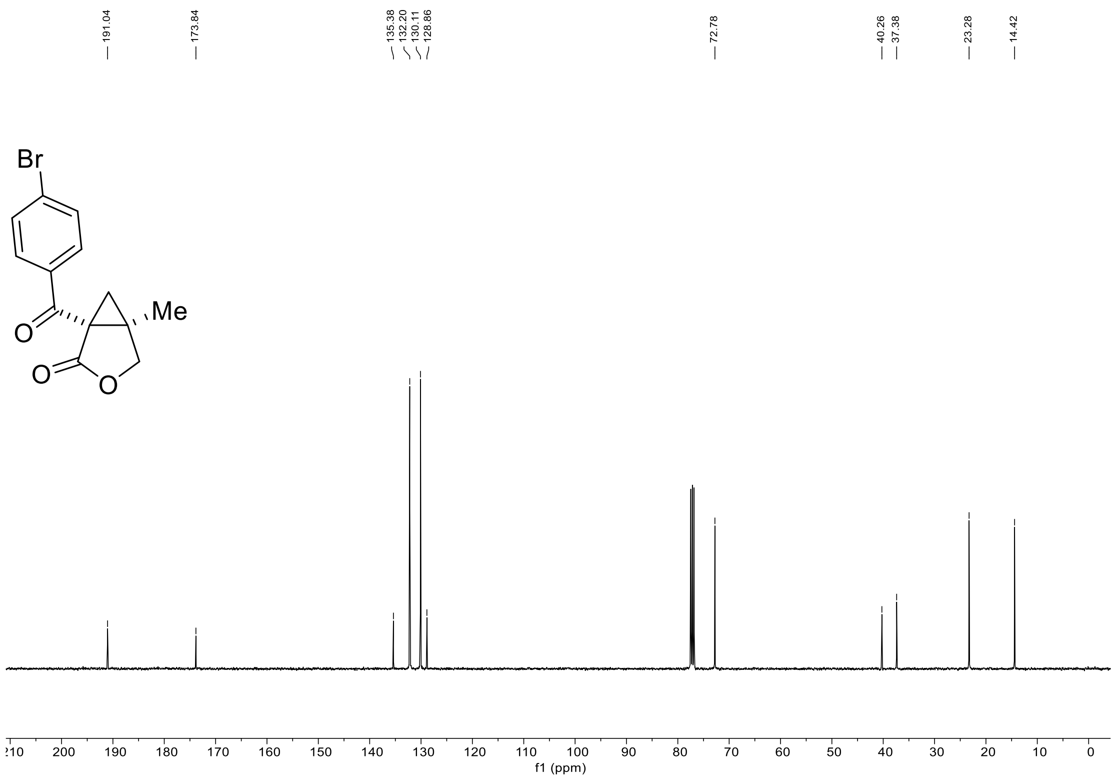

^{13}C NMR spectrum of **10**

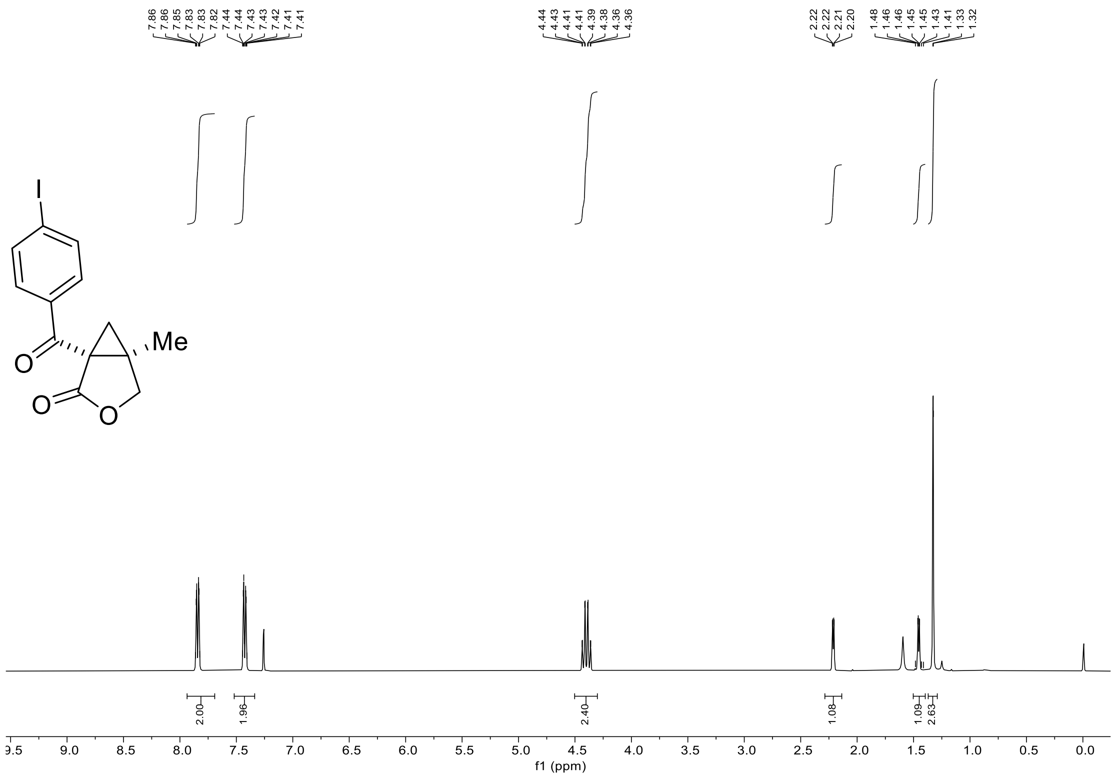

^{1}H NMR spectrum of **11**

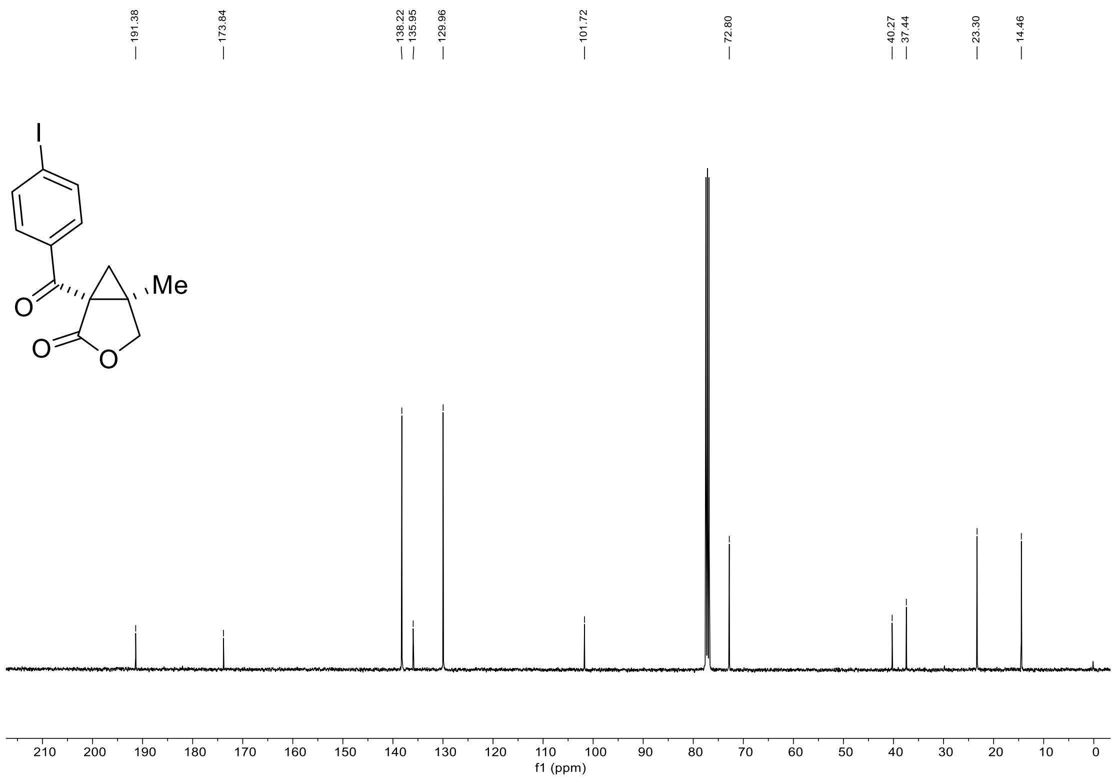

^{13}C NMR spectrum of **11**

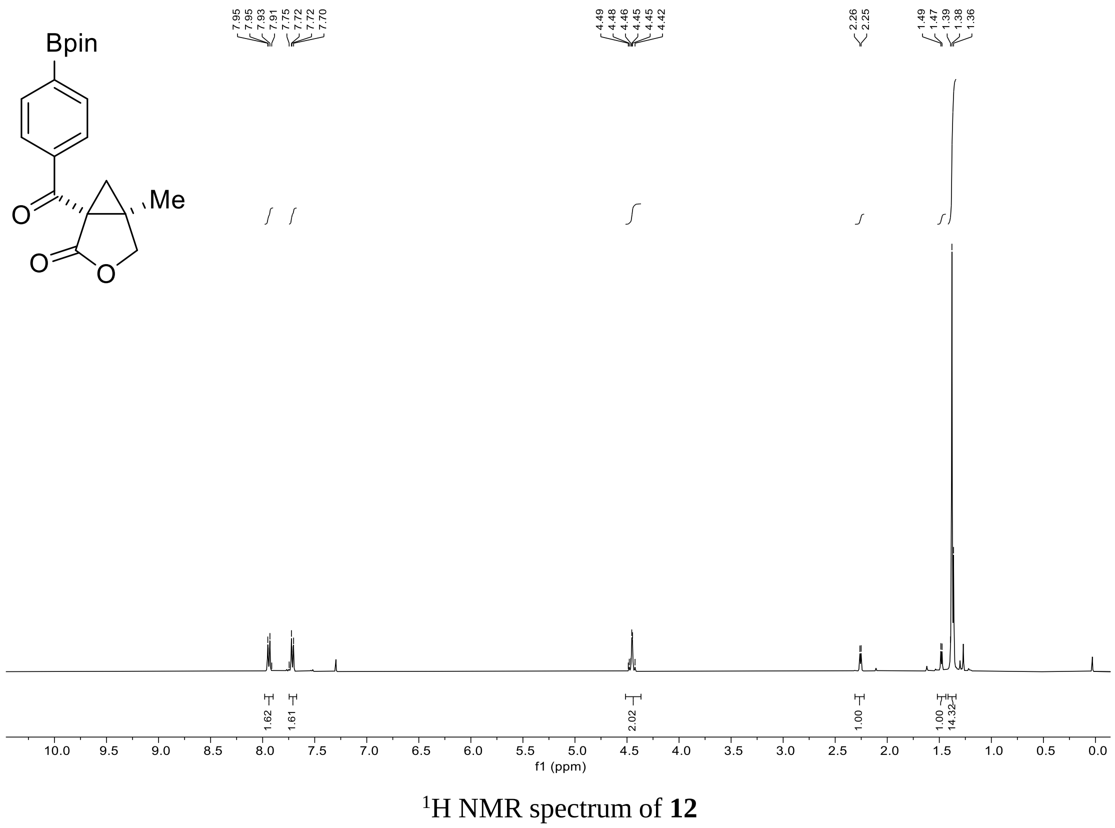

^{1}H NMR spectrum of **12**

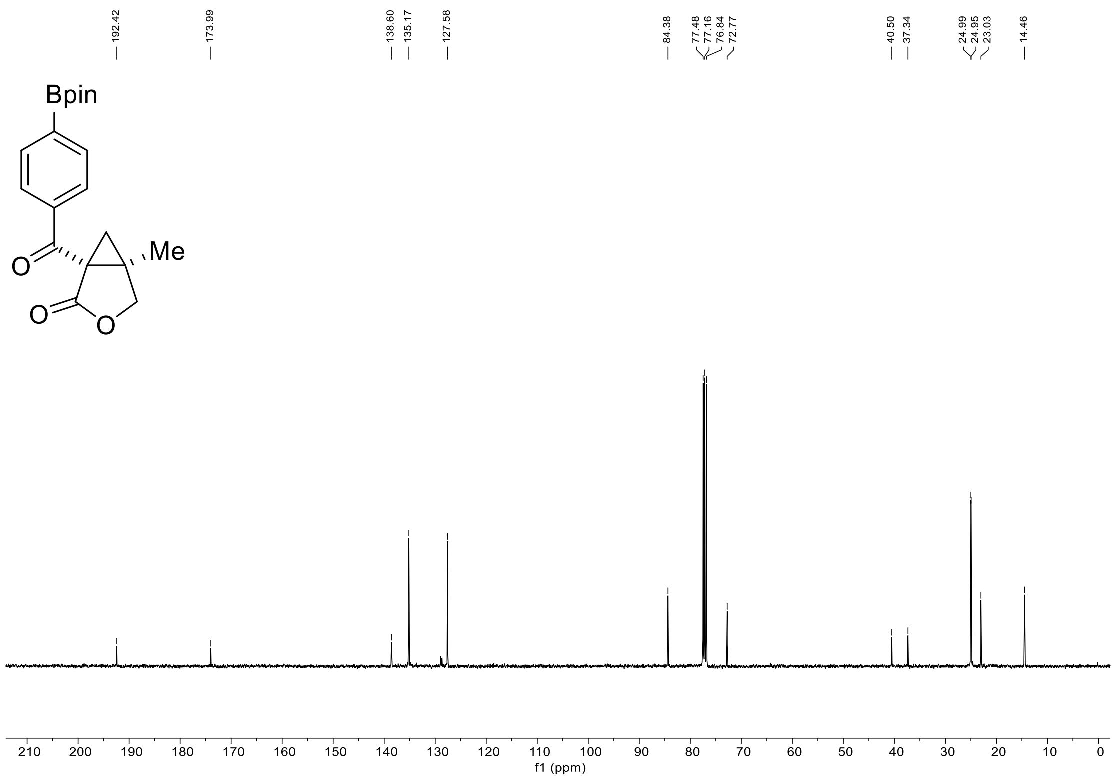

^{13}C NMR spectrum of **12**

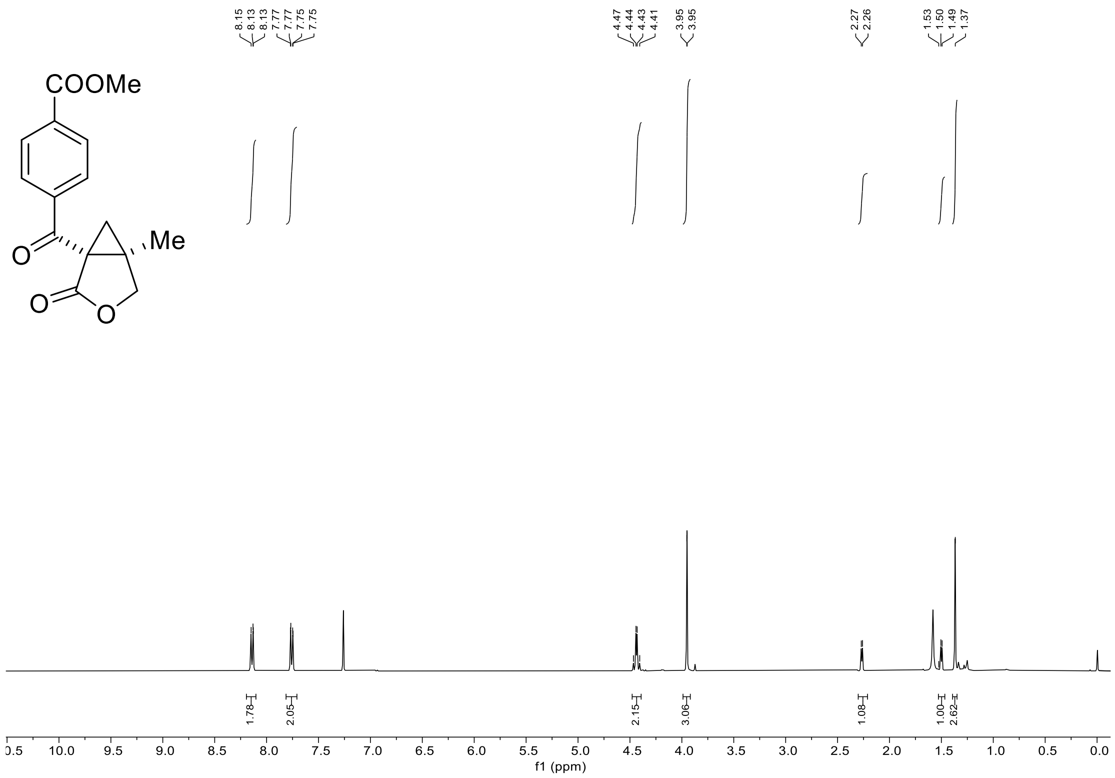

^{1}H NMR spectrum of **13**

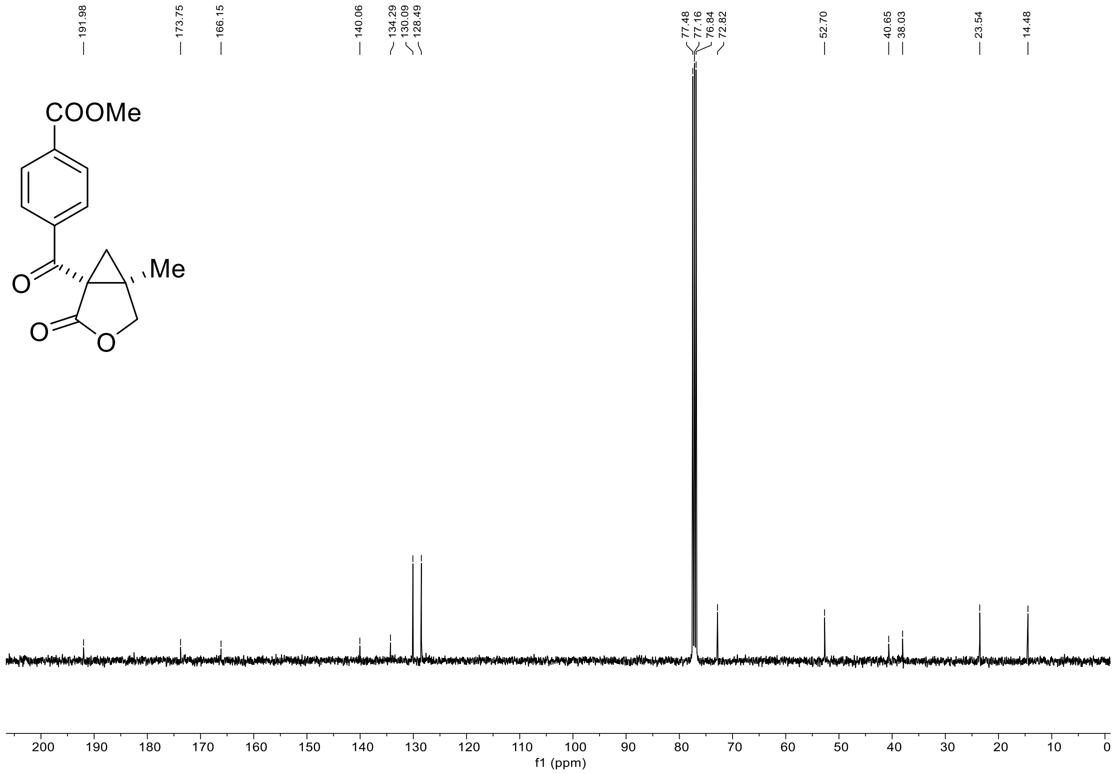

^{13}C NMR spectrum of **13**

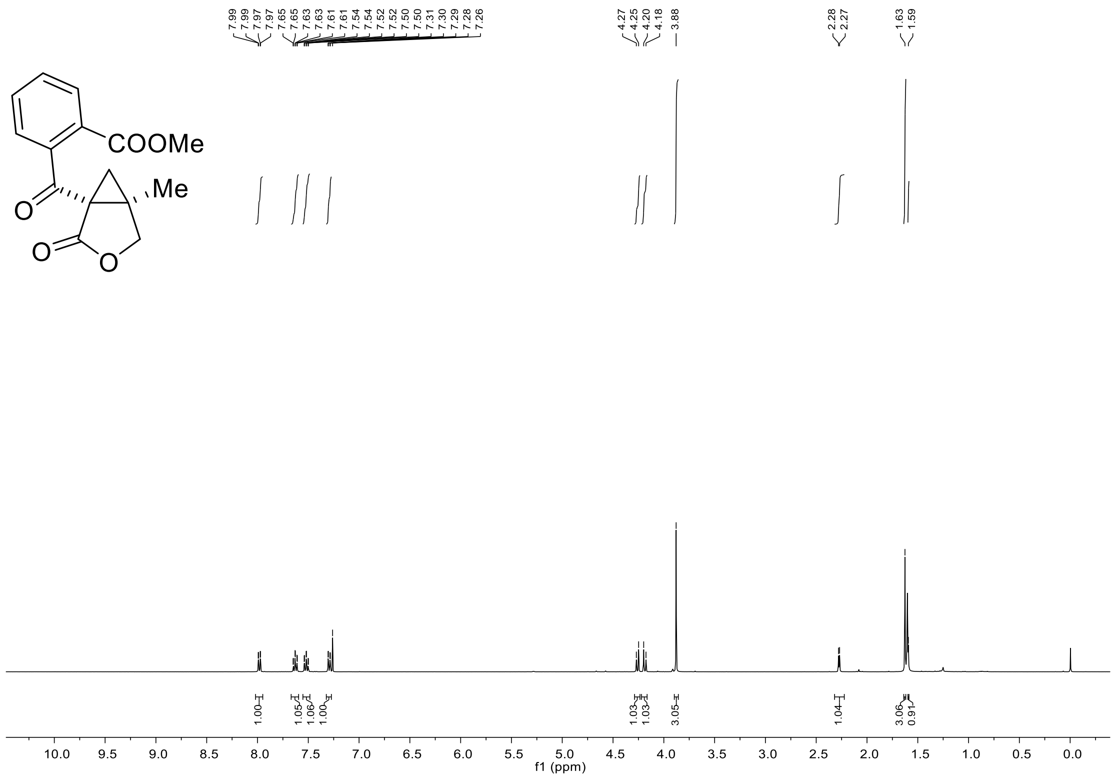

^{1}H NMR spectrum of **14**

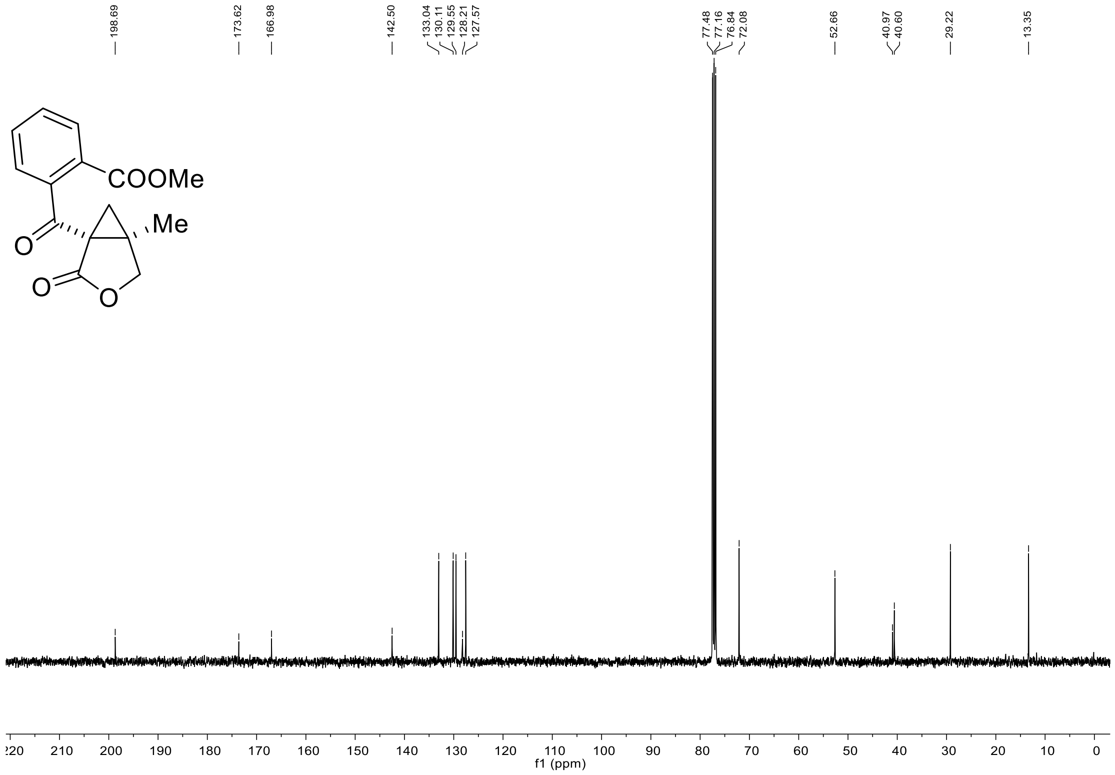

^{13}C NMR spectrum of **14**

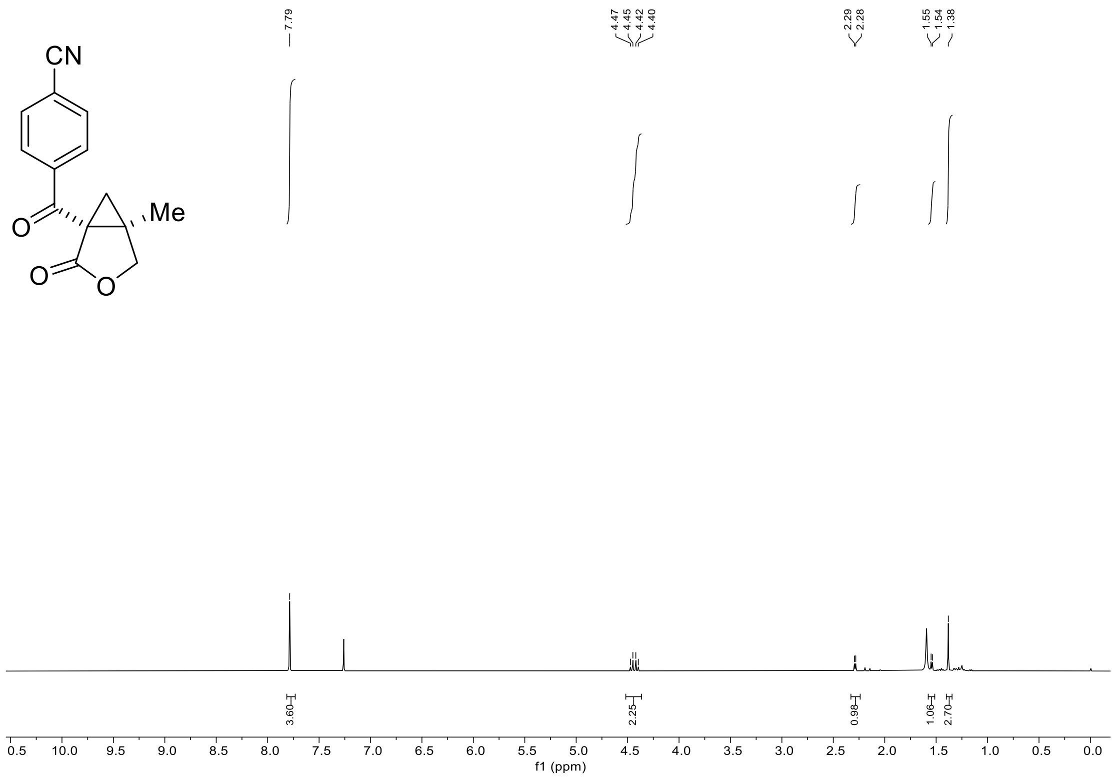

^{1}H NMR spectrum of **15**

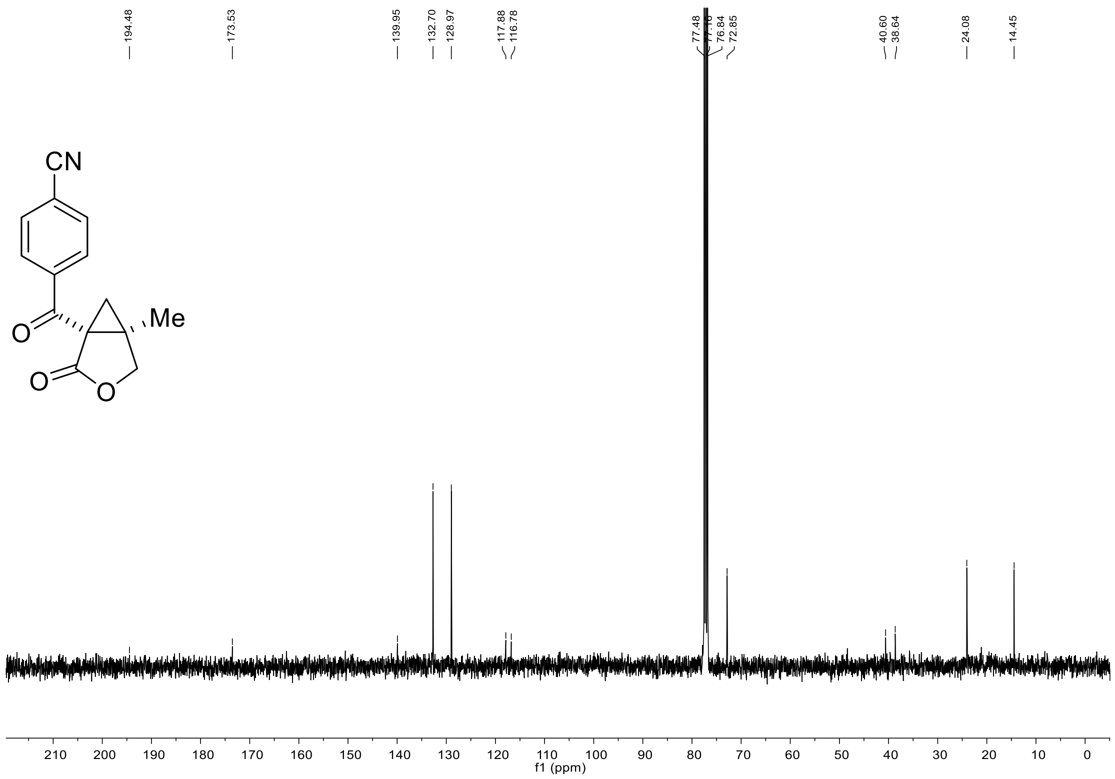

^{13}C NMR spectrum of **15**

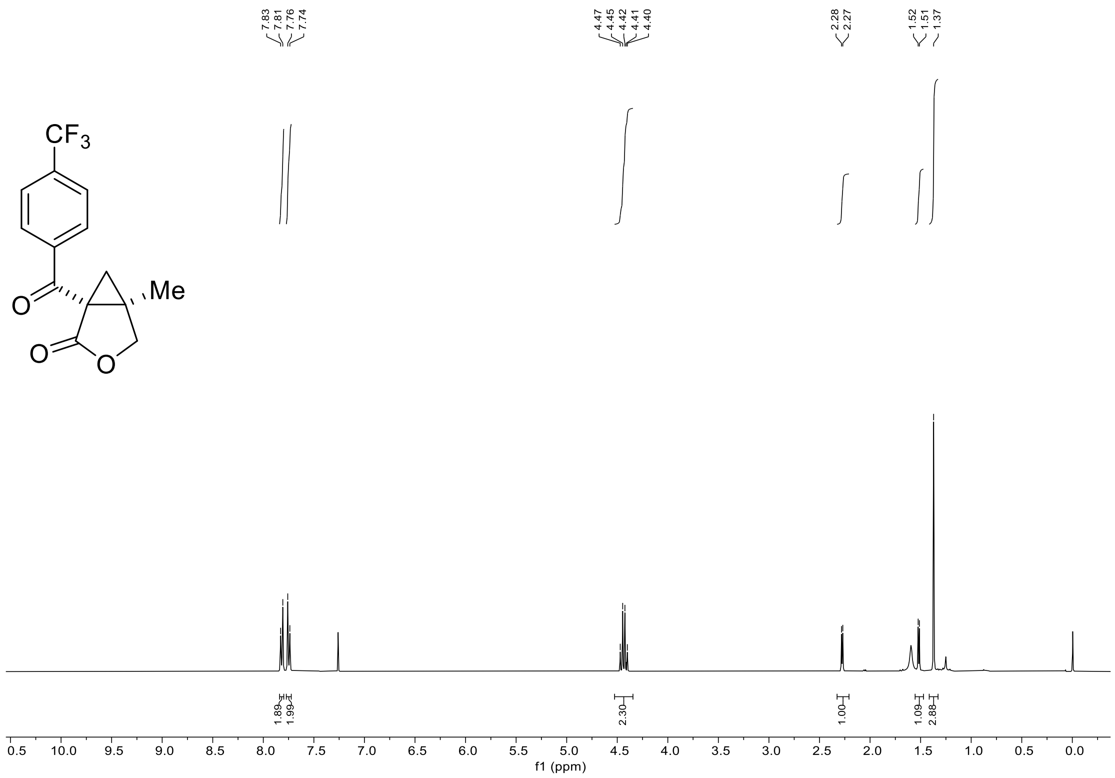

^{1}H NMR spectrum of **16**

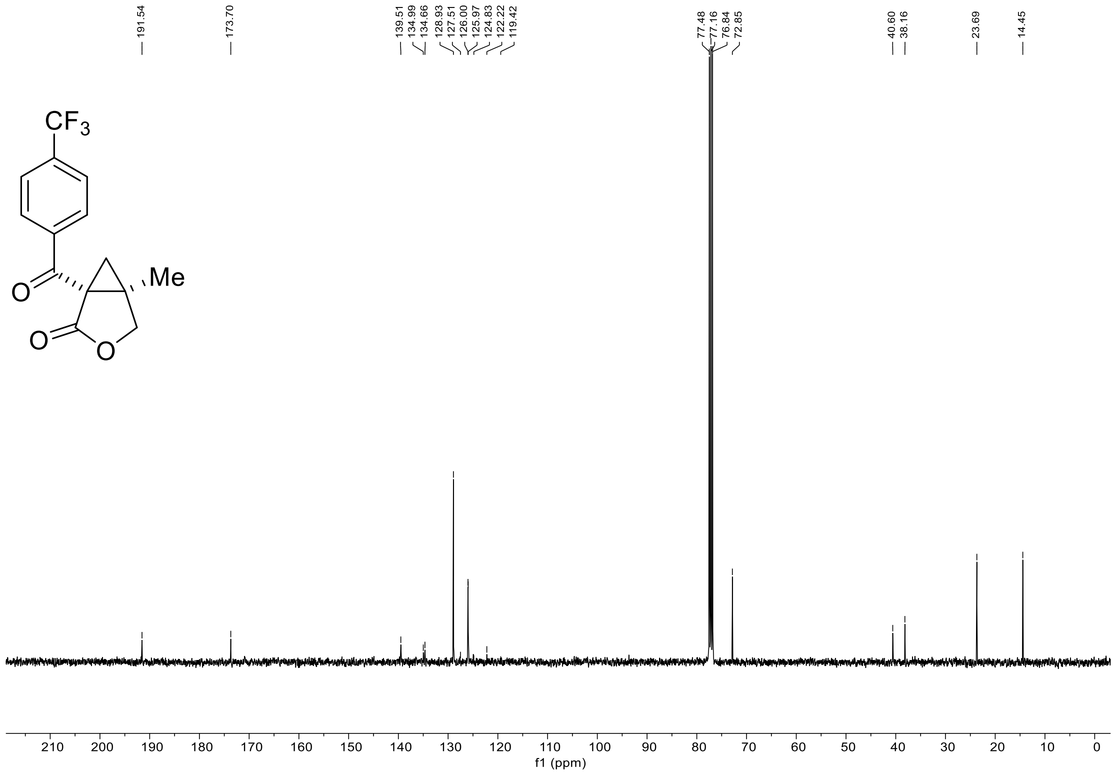

^{13}C NMR spectrum of **16**

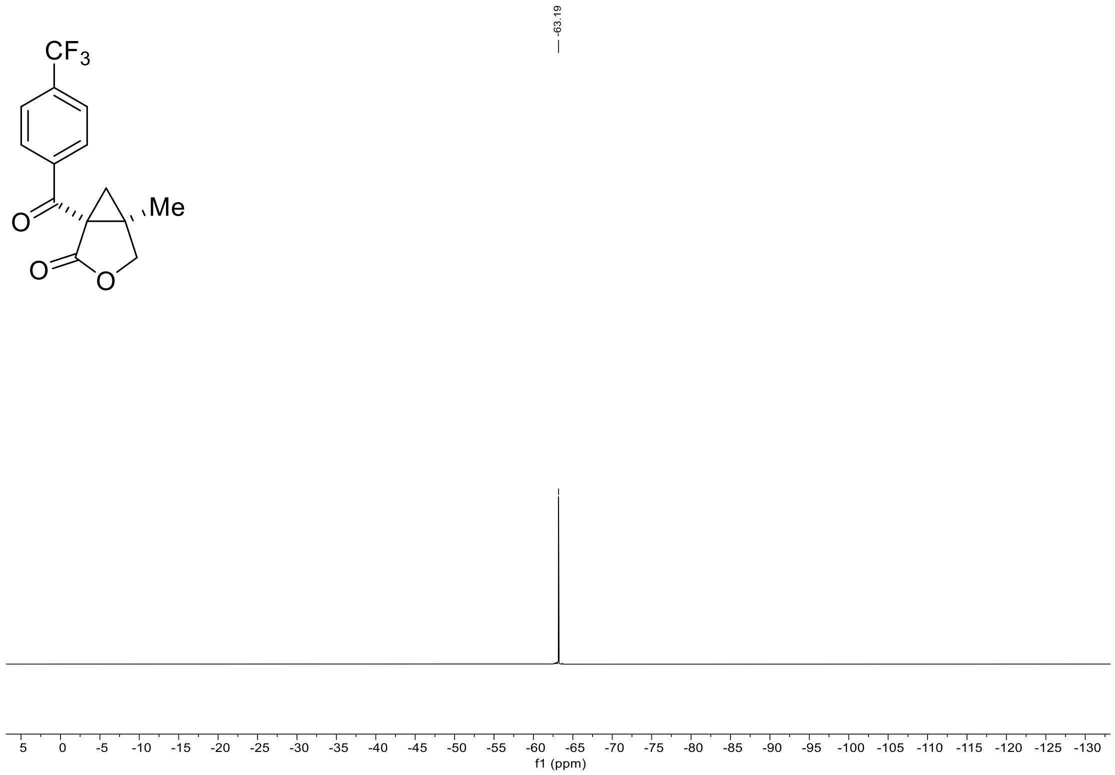

^{19}F NMR spectrum of **16**

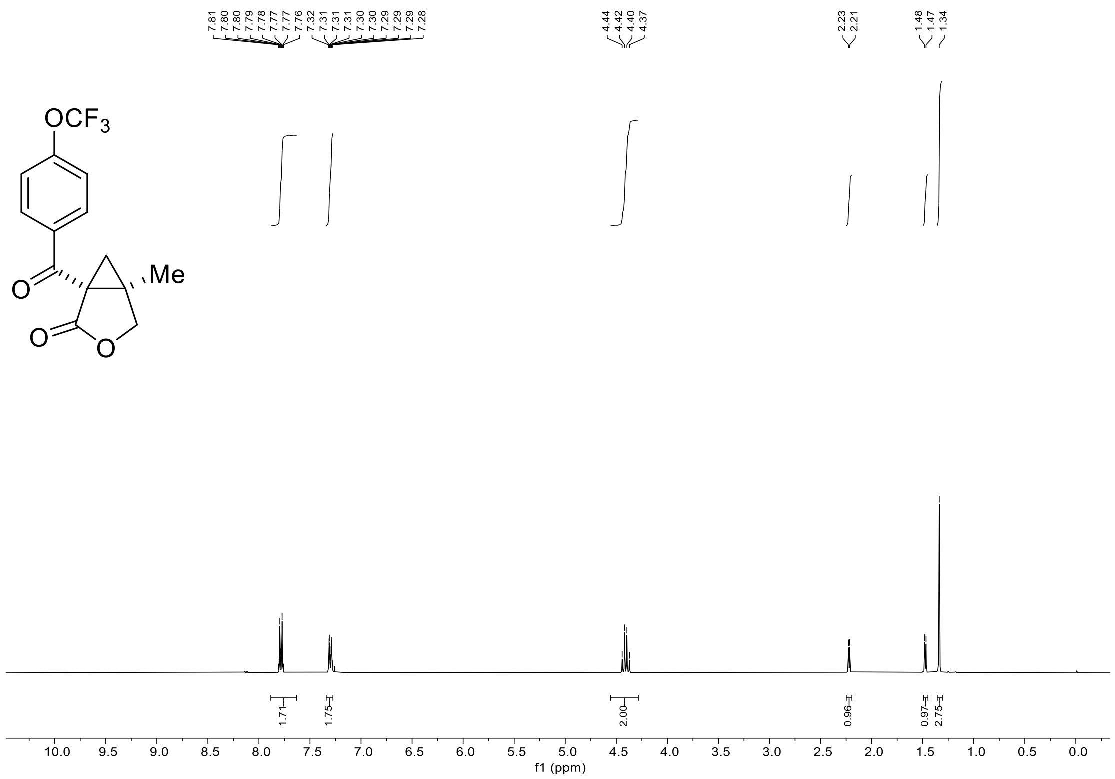

^{1}H NMR spectrum of **17**

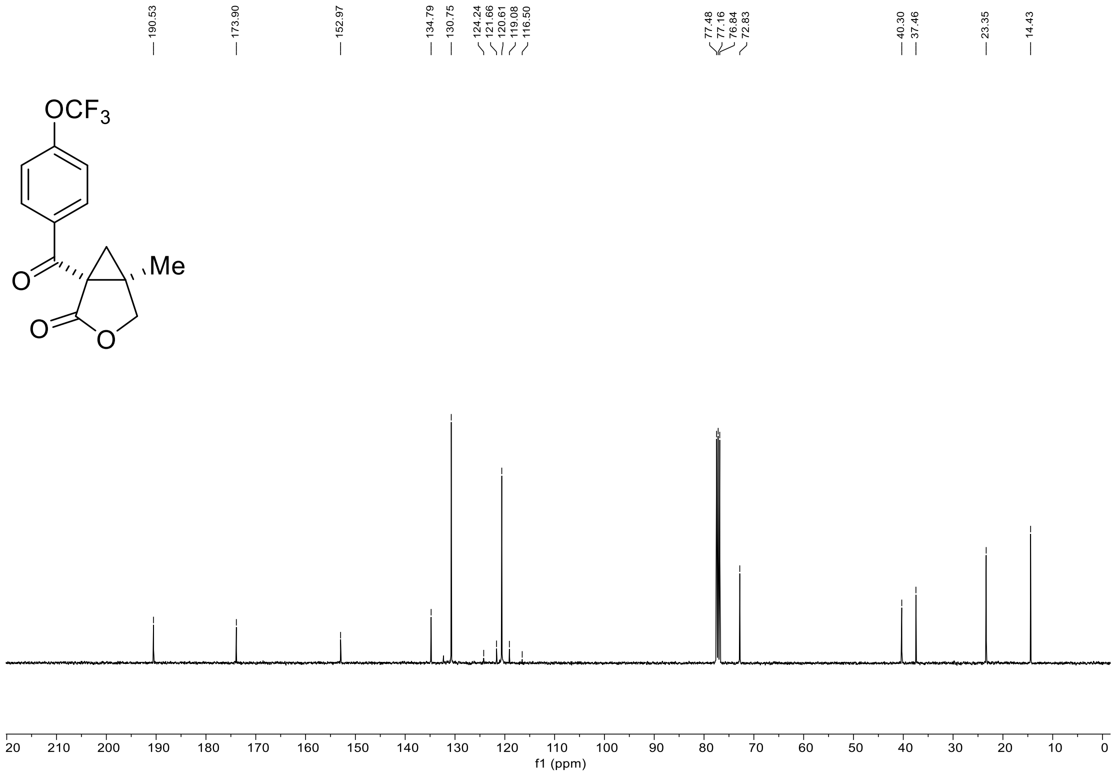

^{13}C NMR spectrum of **17**

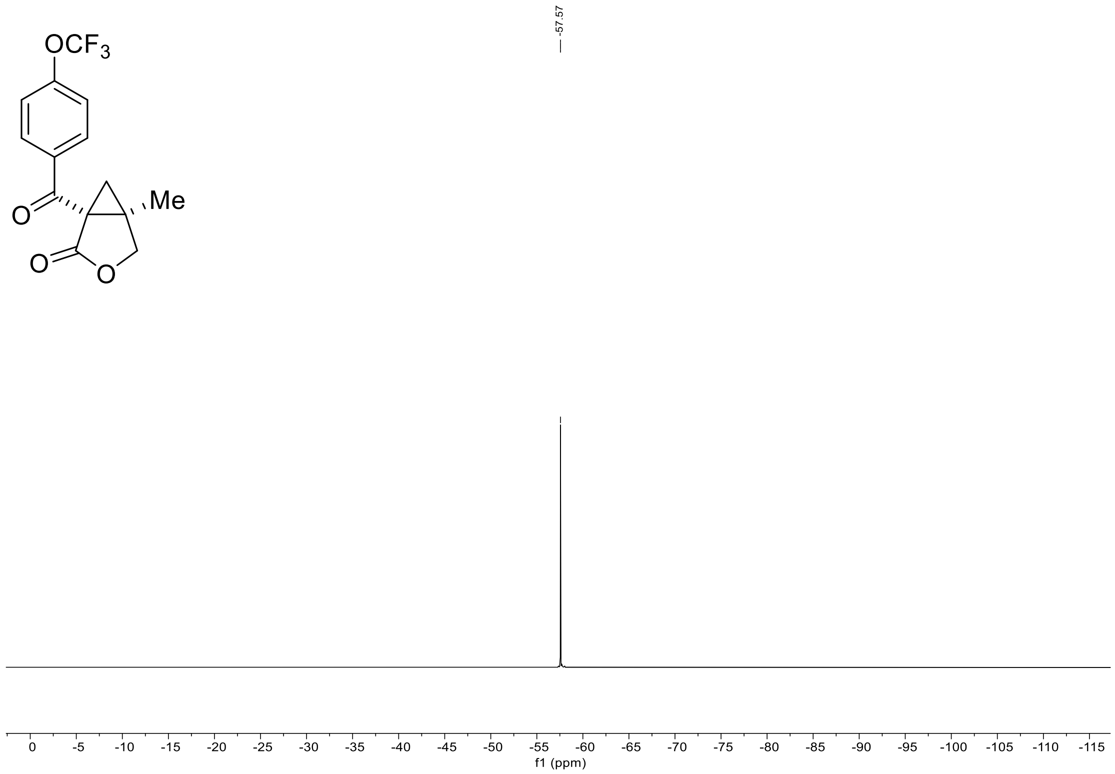

^{19}F NMR spectrum of **17**

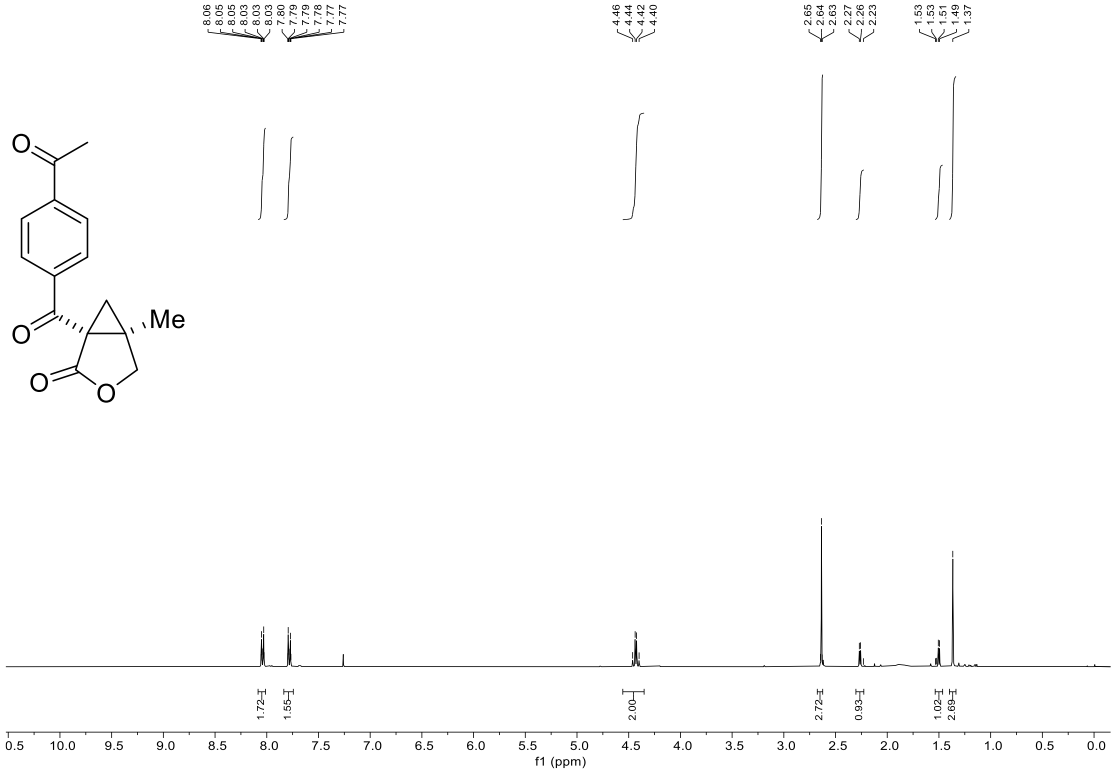

^{13}C NMR spectrum of **18**

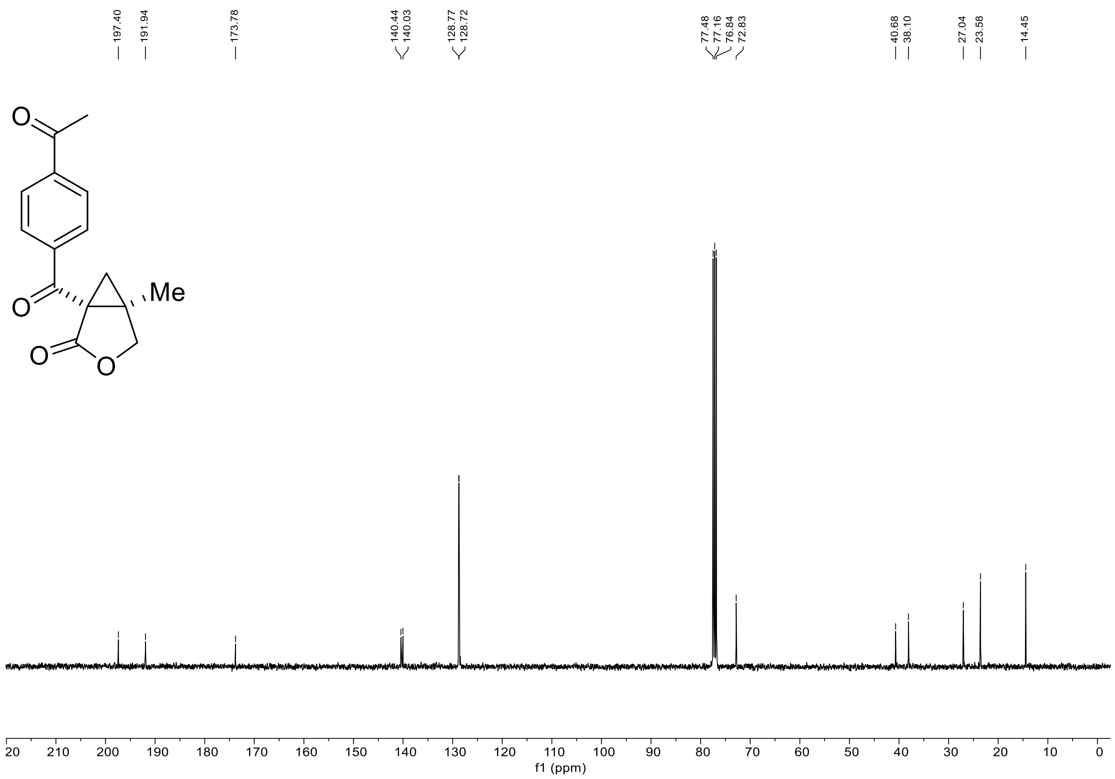

^{13}C NMR spectrum of **18**

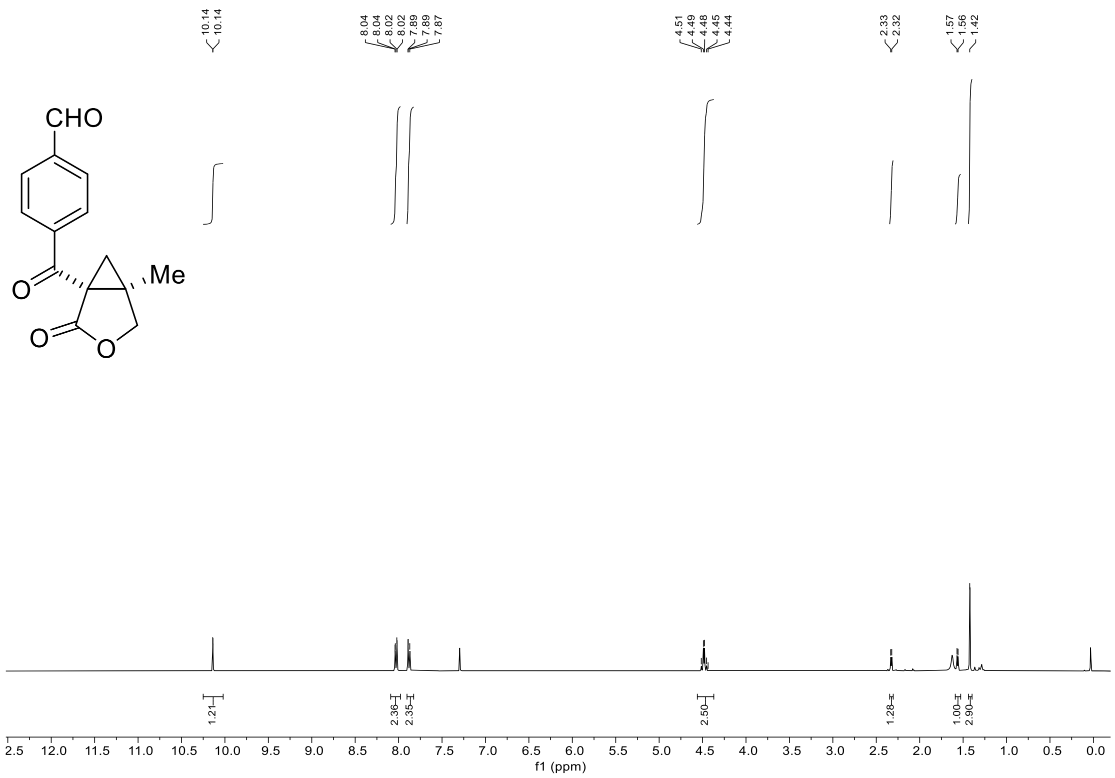

^{1}H NMR spectrum of **19**

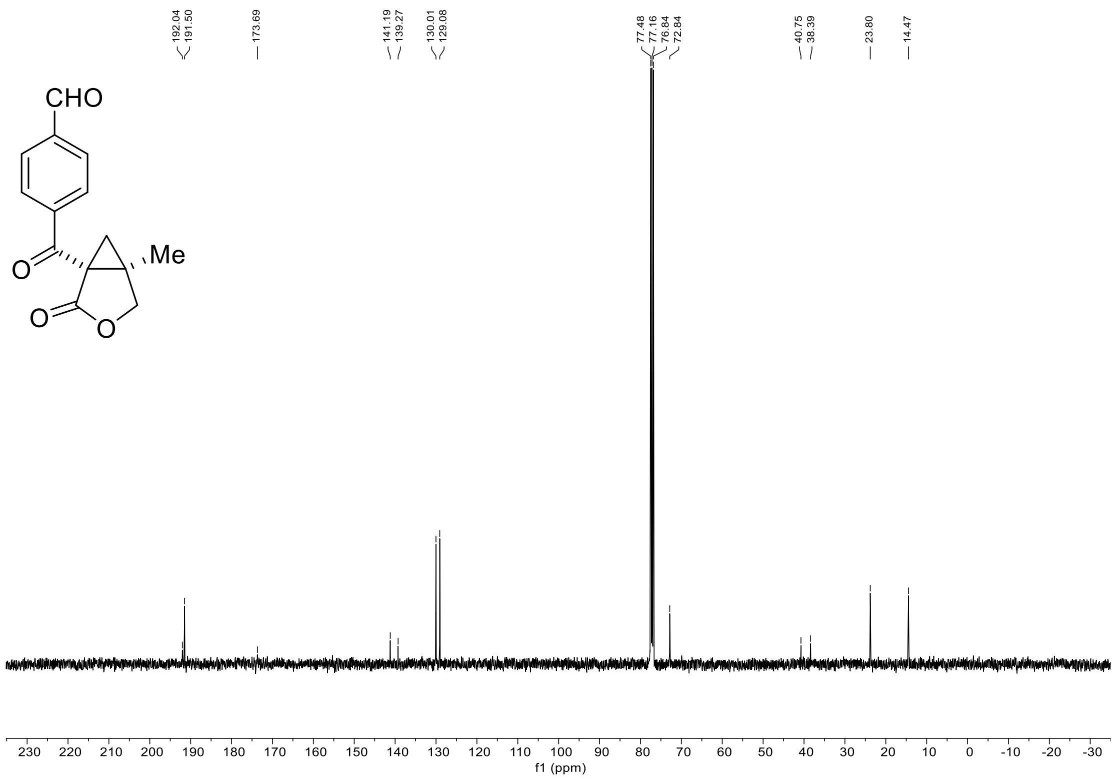

^{13}C NMR spectrum of **19**

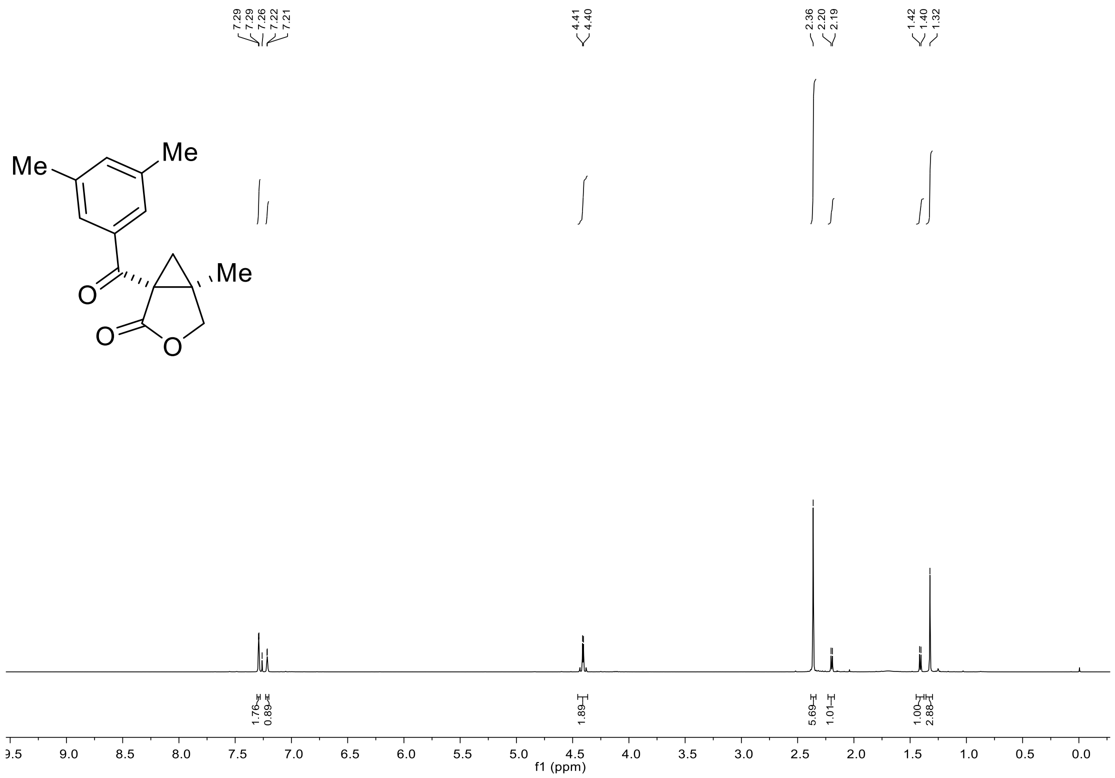

^{1}H NMR spectrum of **20**

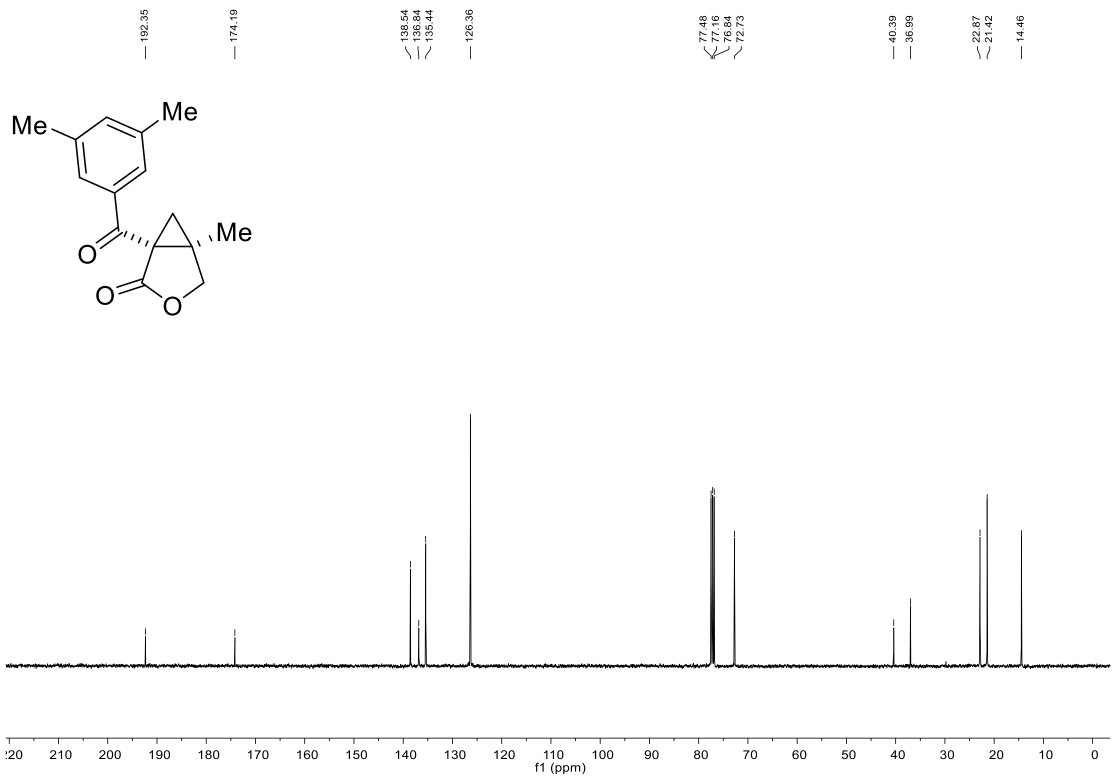

^{13}C NMR spectrum of **20**

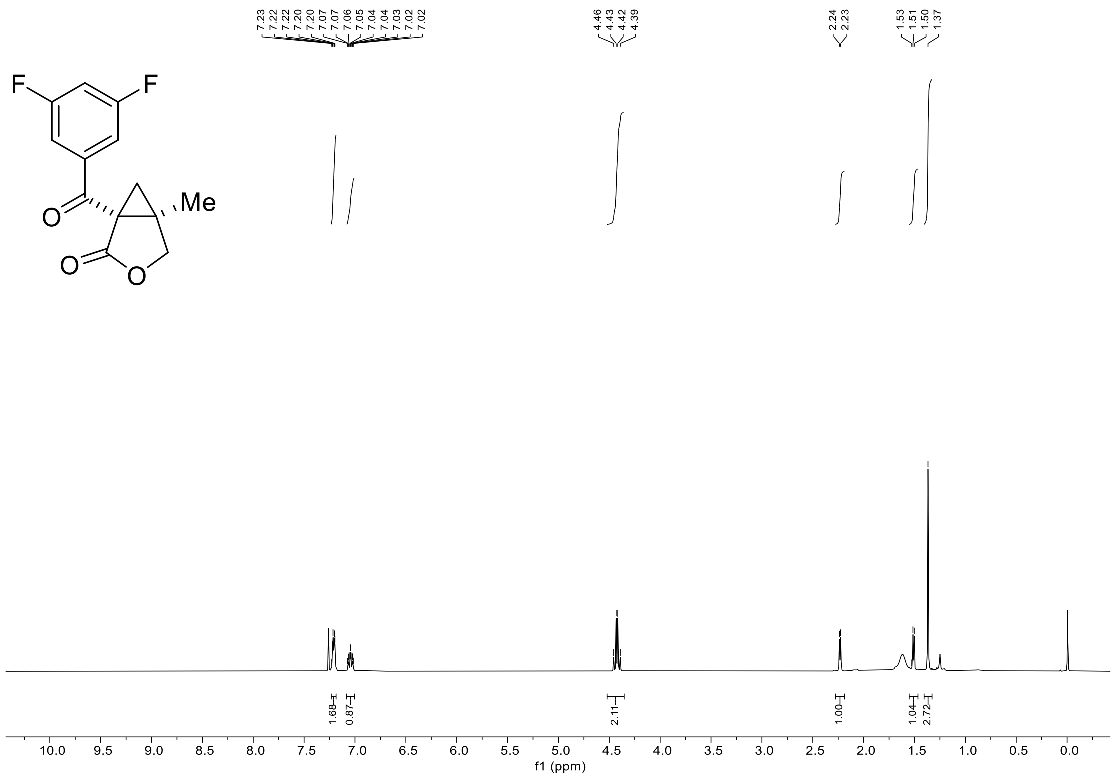

^{1}H NMR spectrum of **21**

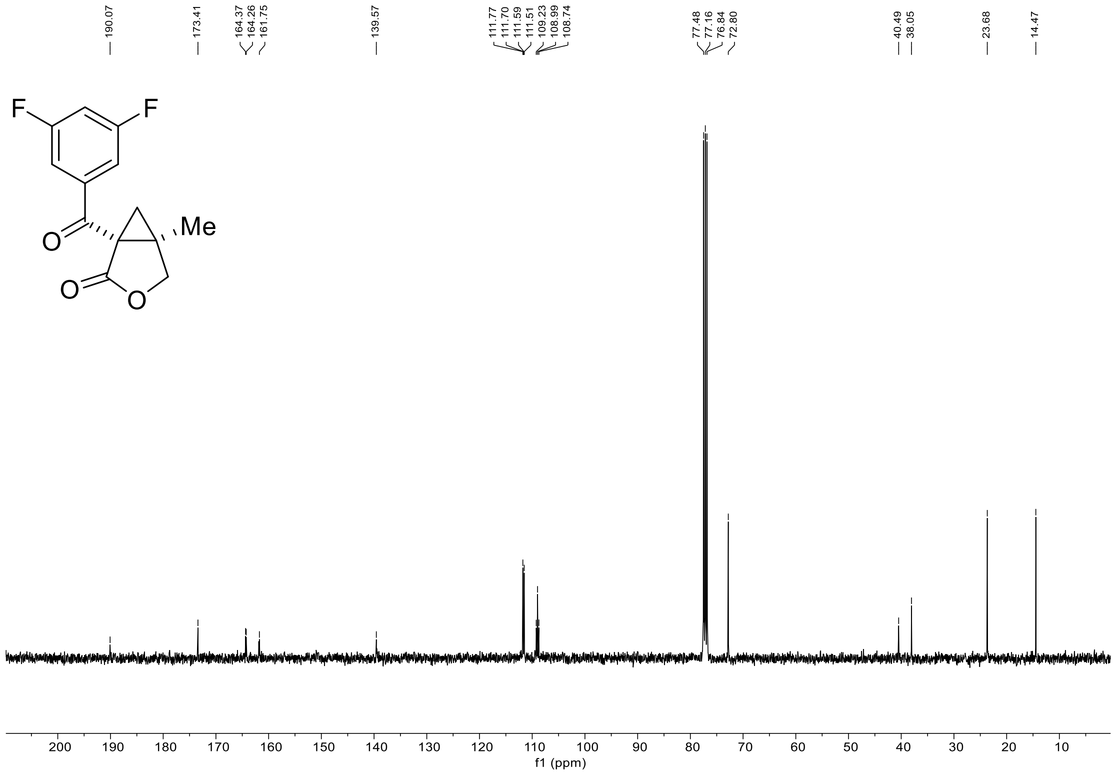

^{13}C NMR spectrum of **21**

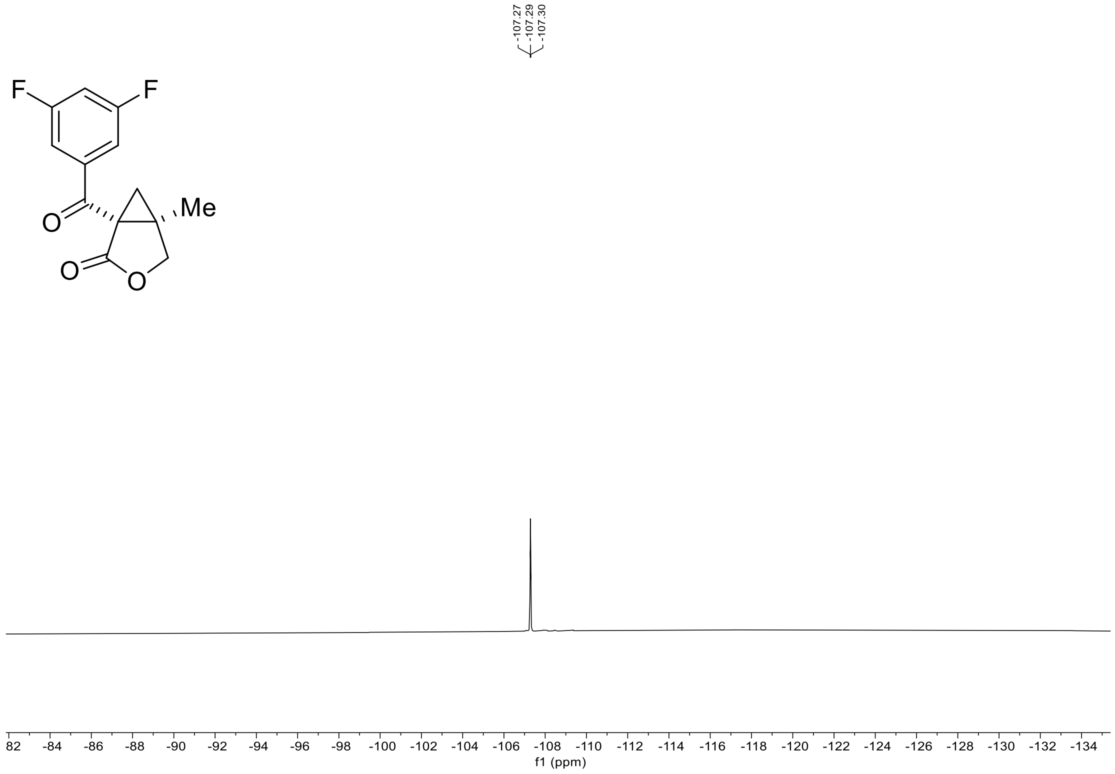

^{1}H NMR spectrum of **21**

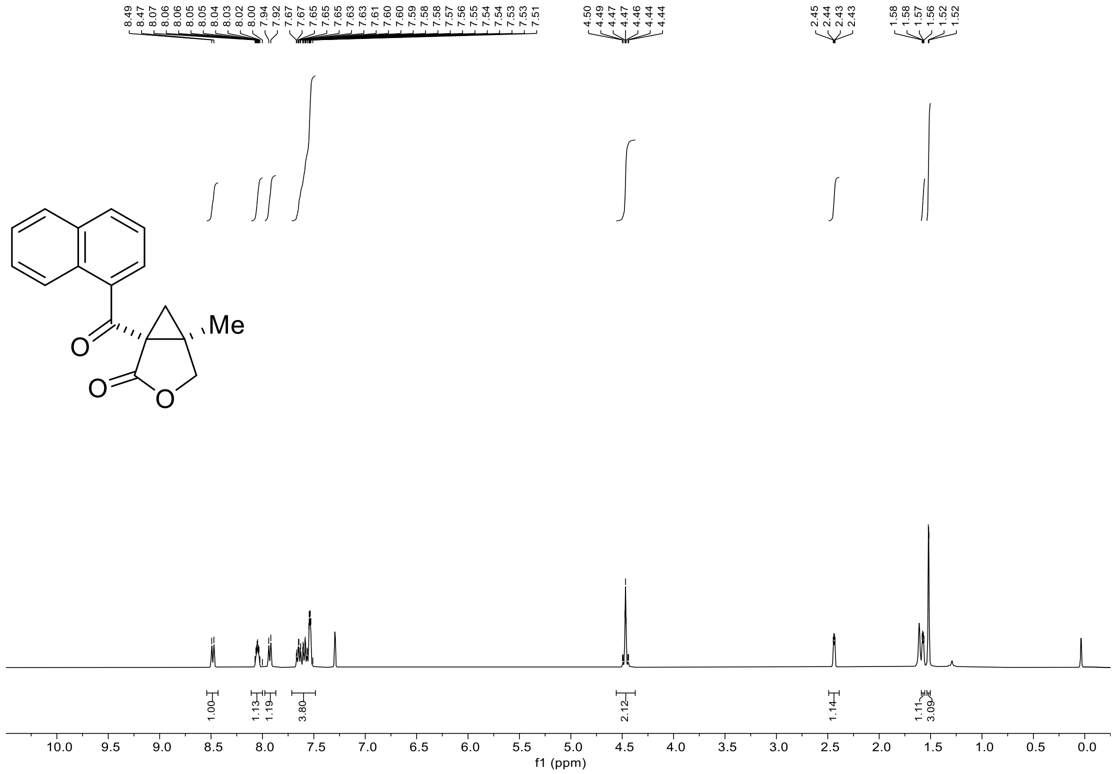

^{1}H NMR spectrum of **22**

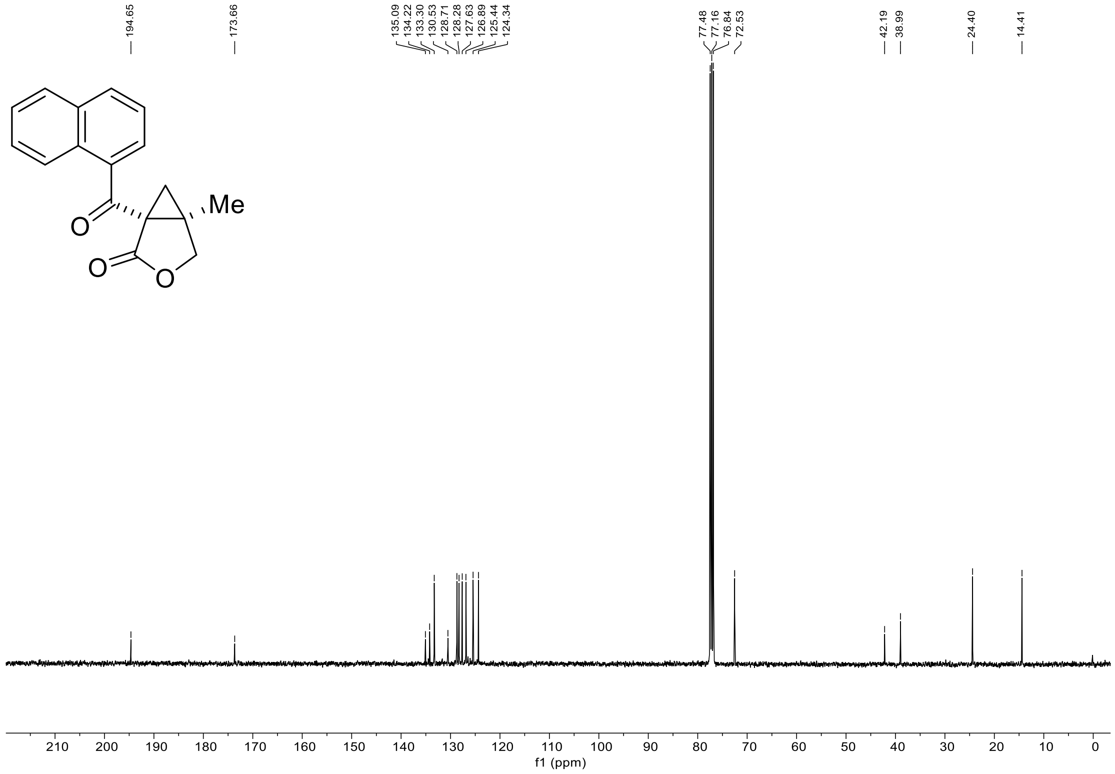

^{13}C NMR spectrum of **22**

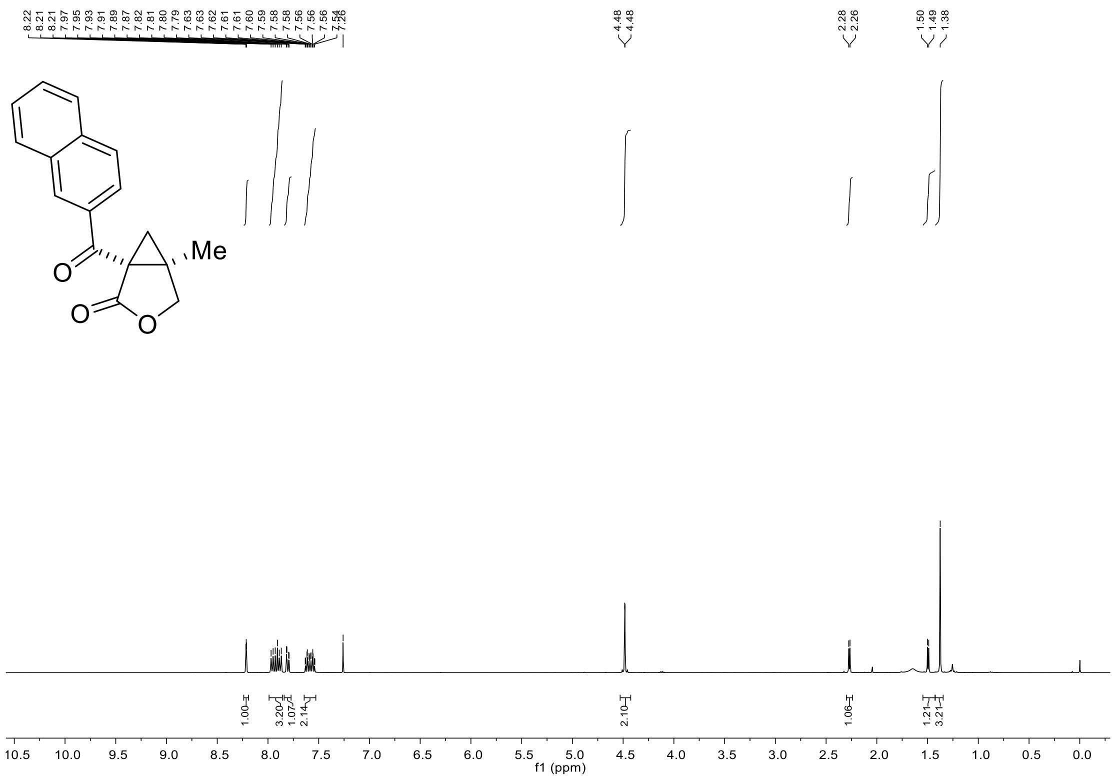

^{1}H NMR spectrum of **23**

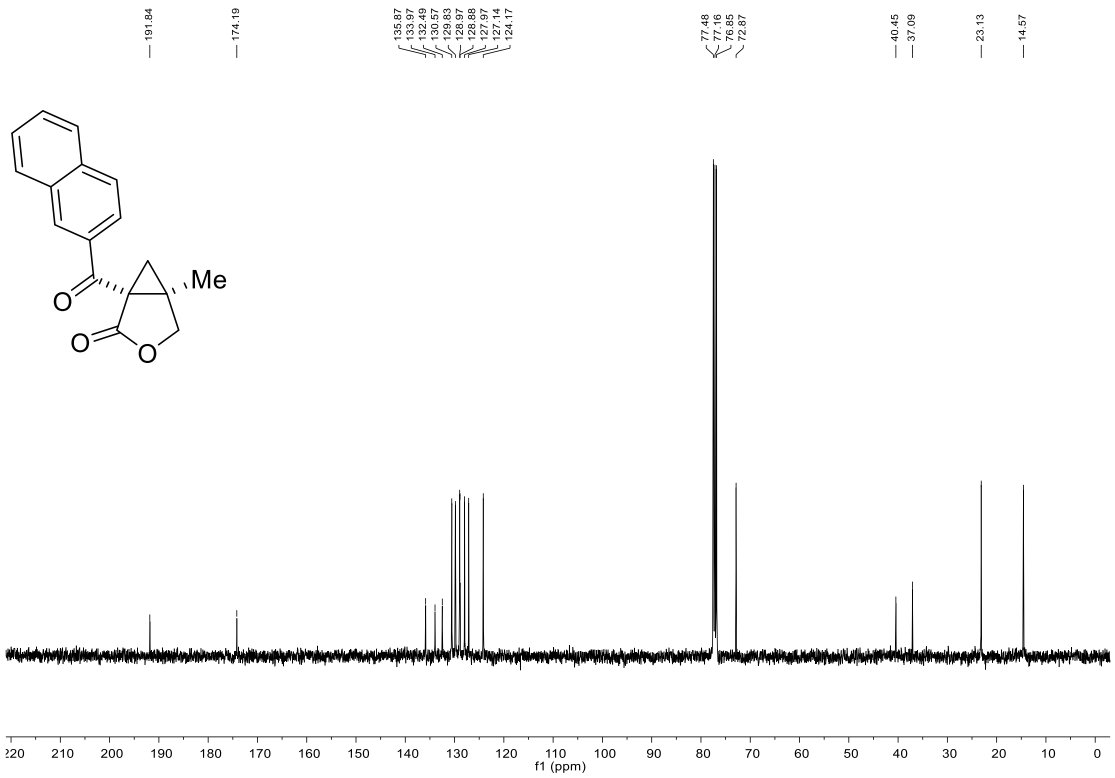

^{13}C NMR spectrum of **23**

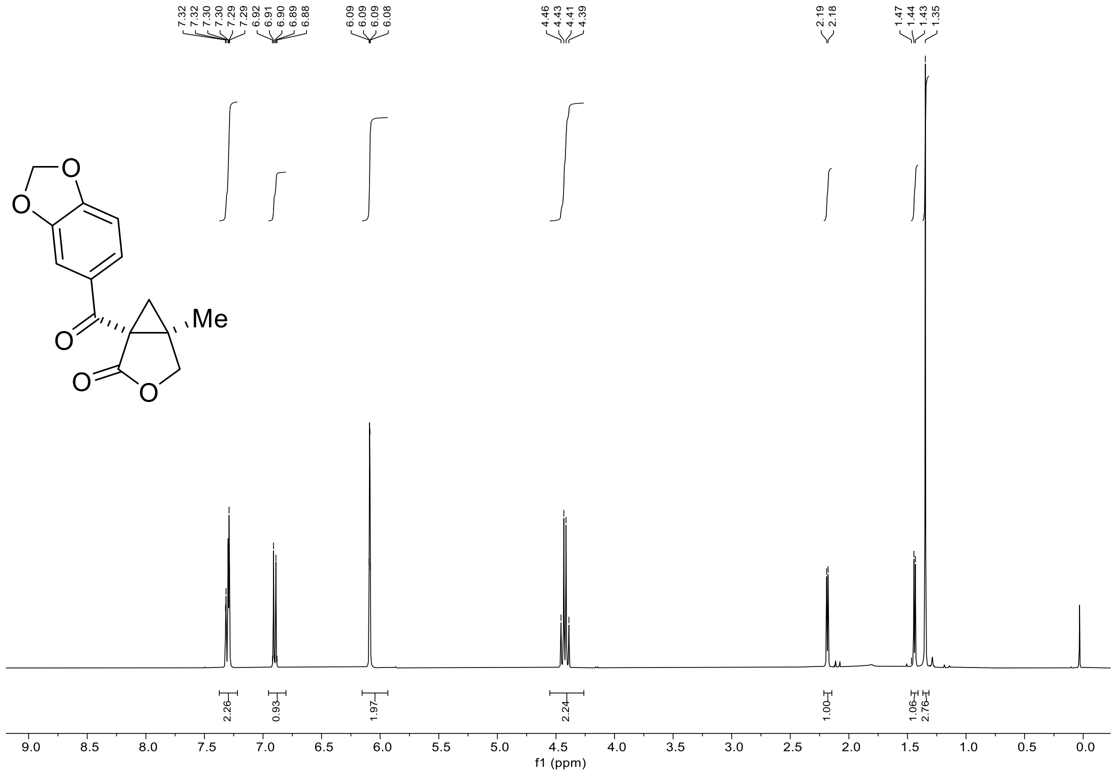

^{1}H NMR spectrum of **24**

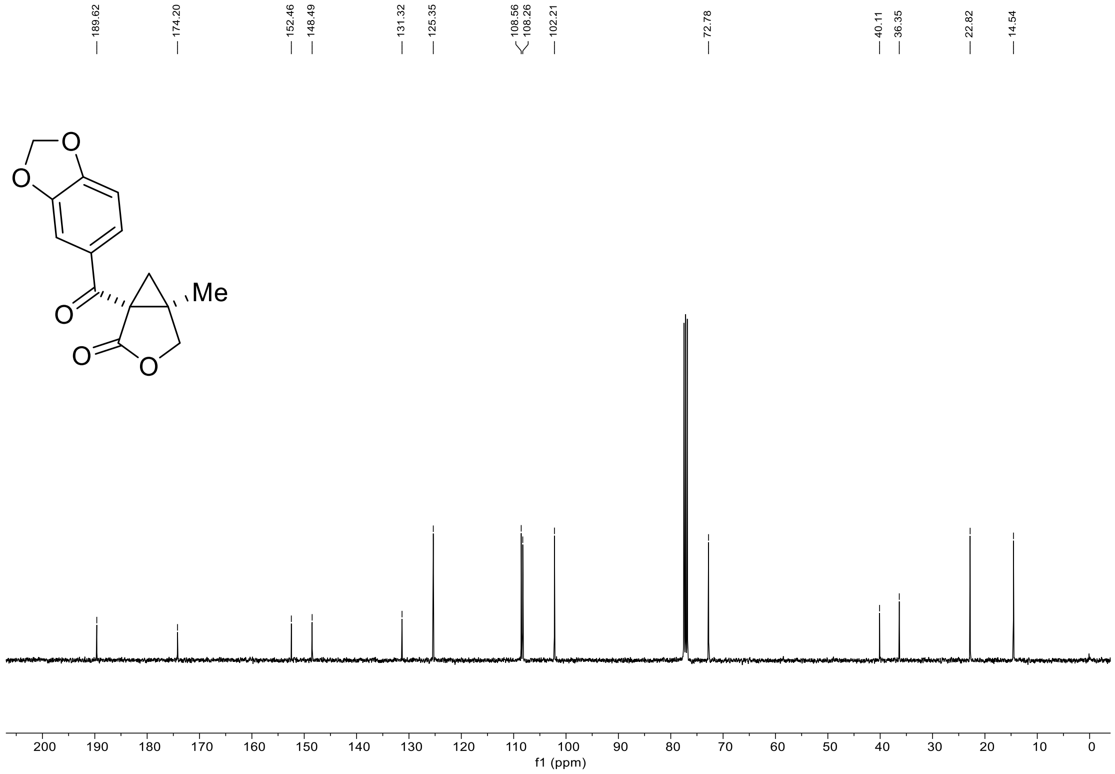

^{13}C NMR spectrum of **24**

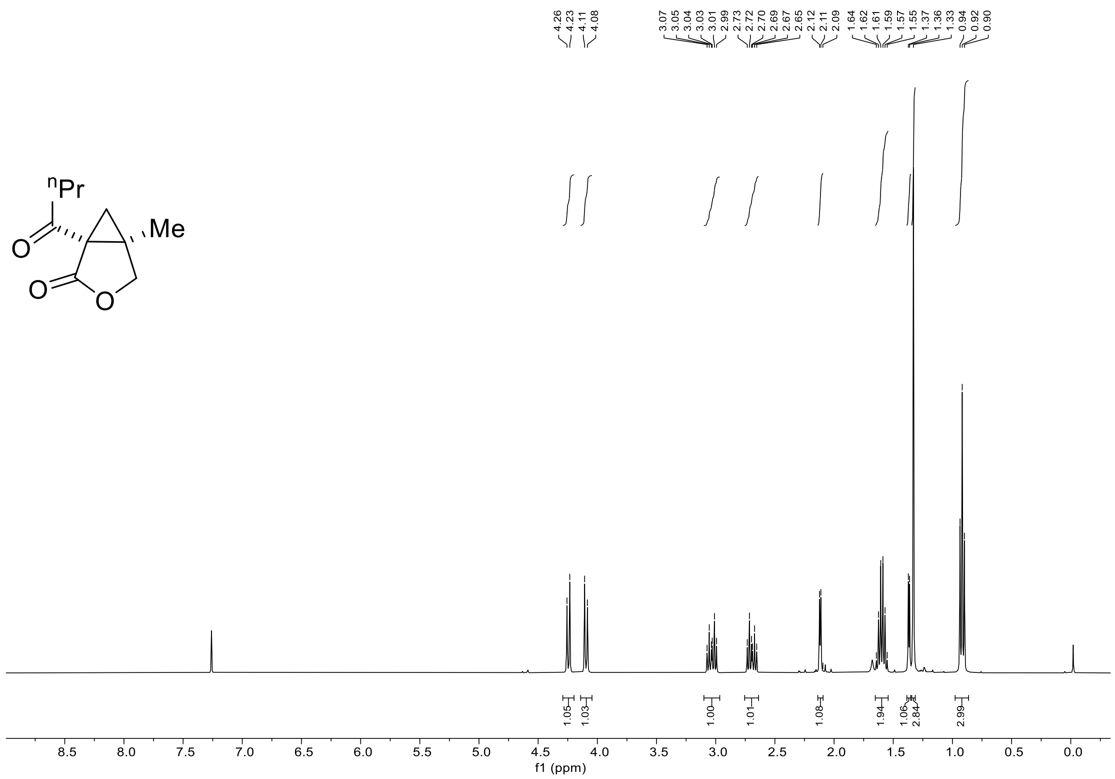

^{1}H NMR spectrum of **25**

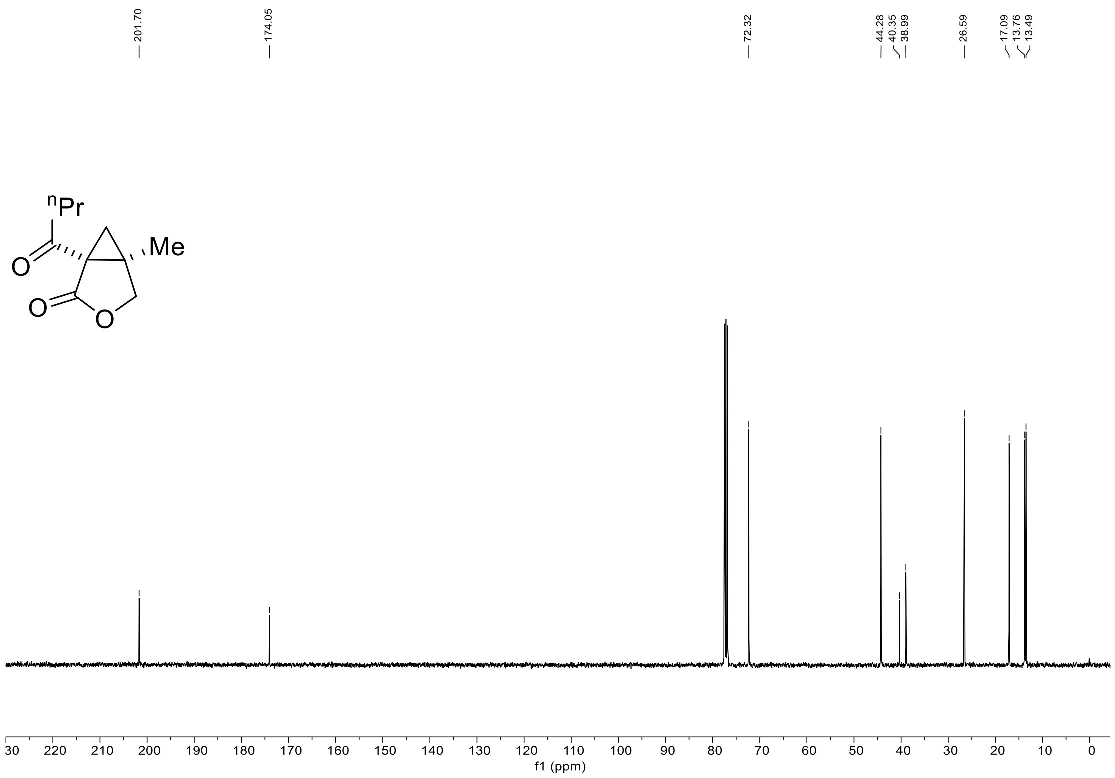

^{13}C NMR spectrum of **25**

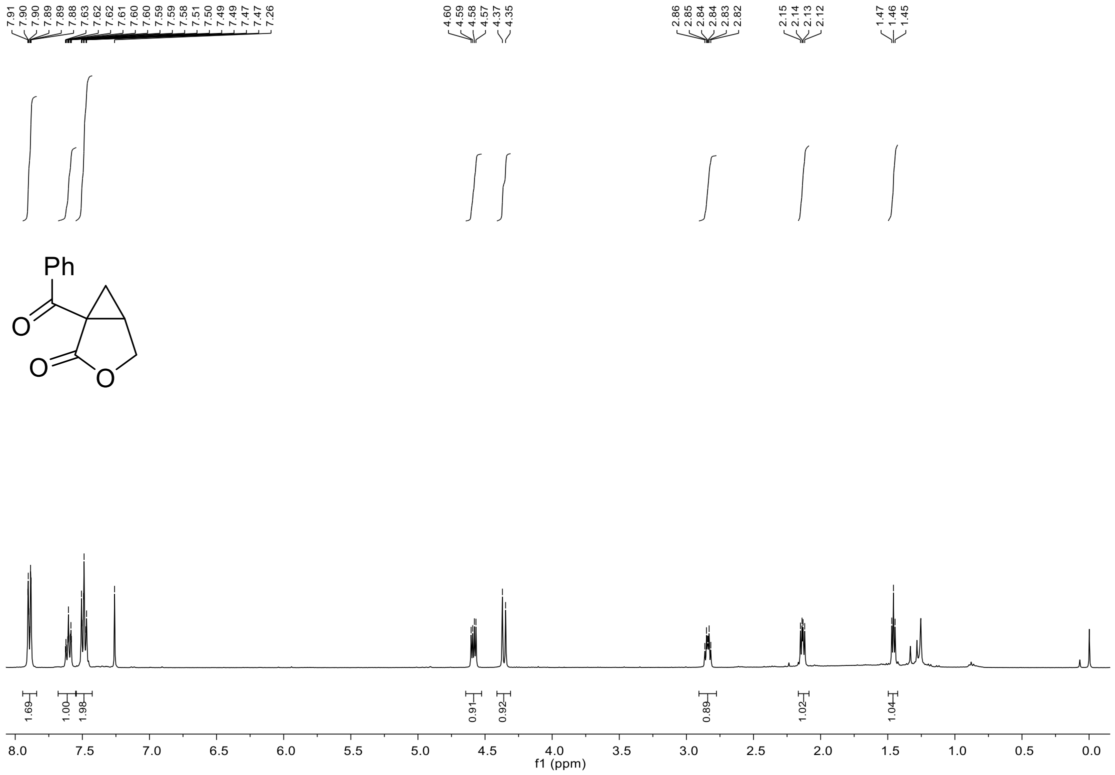

^{1}H NMR spectrum of **26**

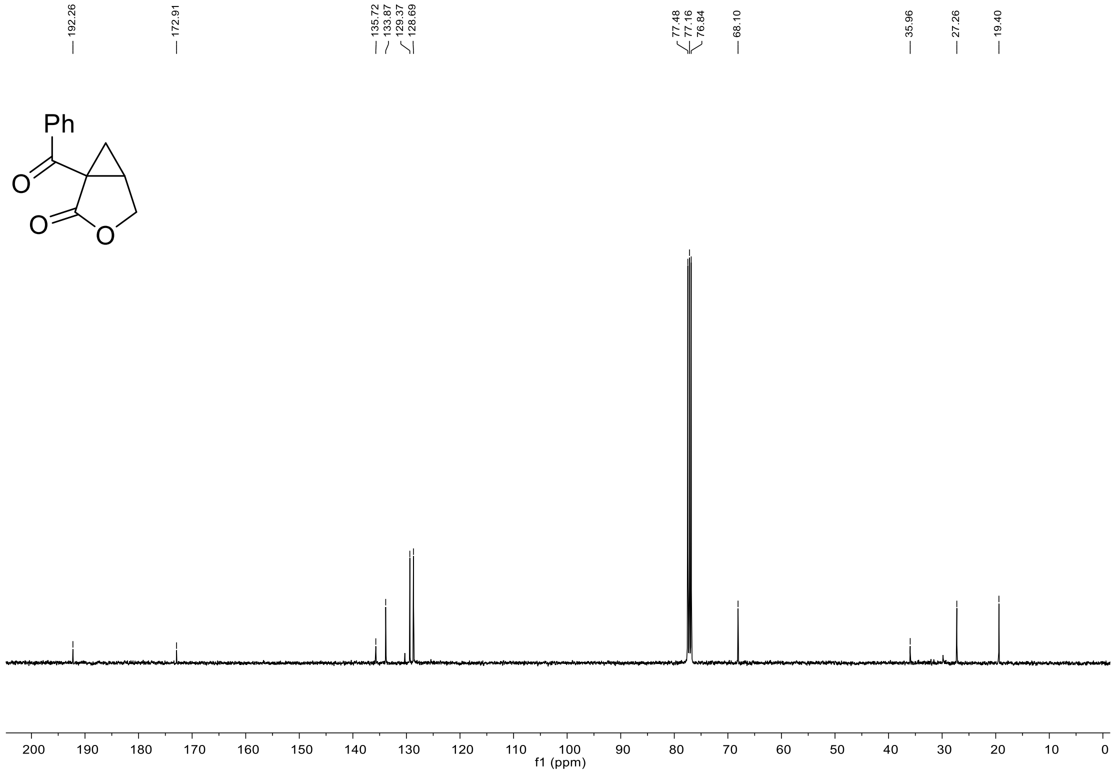

^{13}C NMR spectrum of **26**

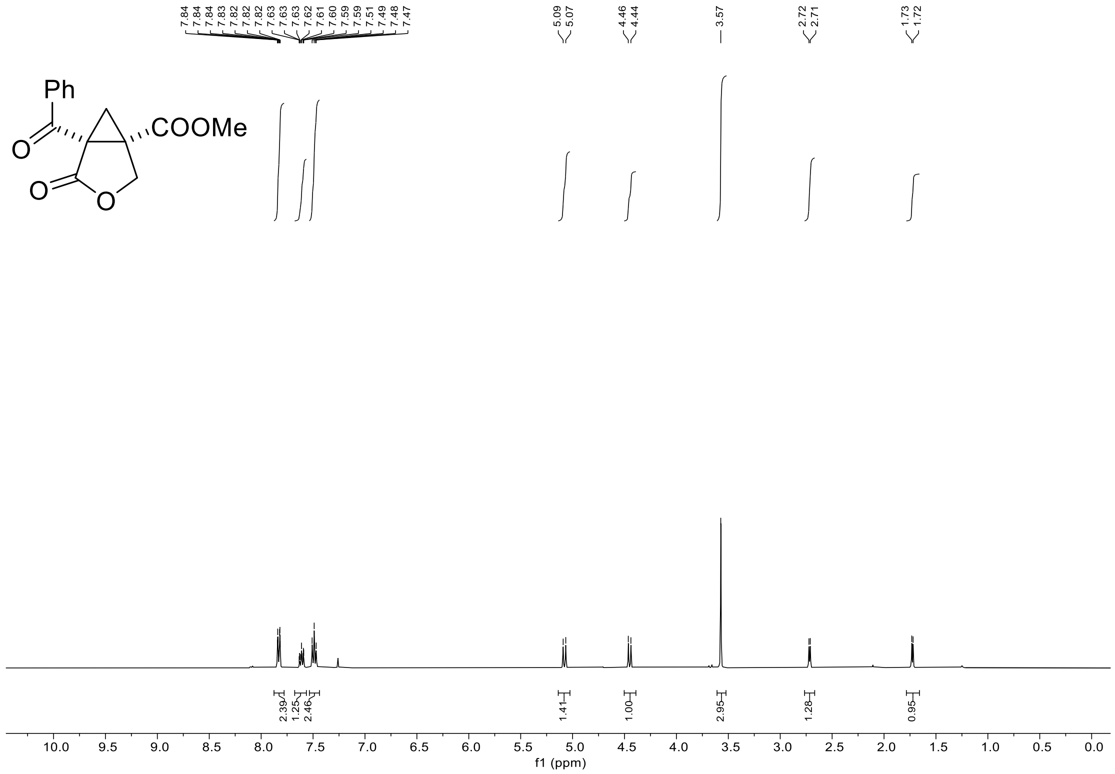

^{1}H NMR spectrum of **27**

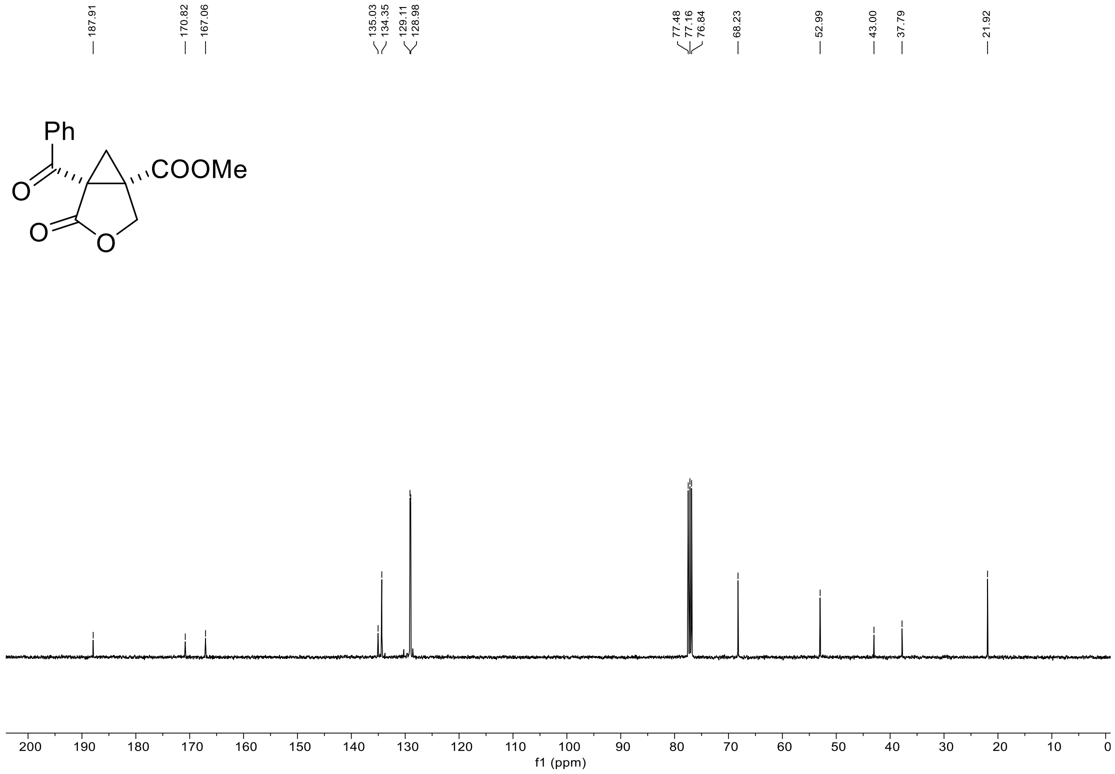

^{13}C NMR spectrum of **27**

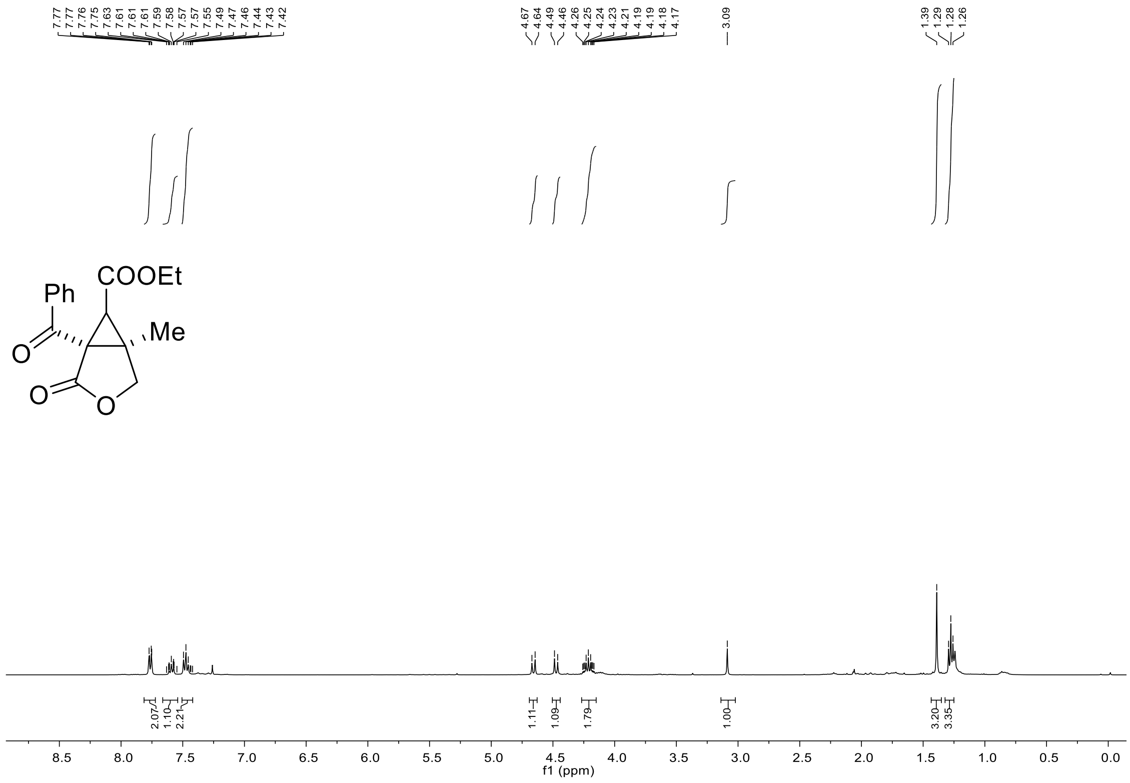

^{1}H NMR spectrum of **28**

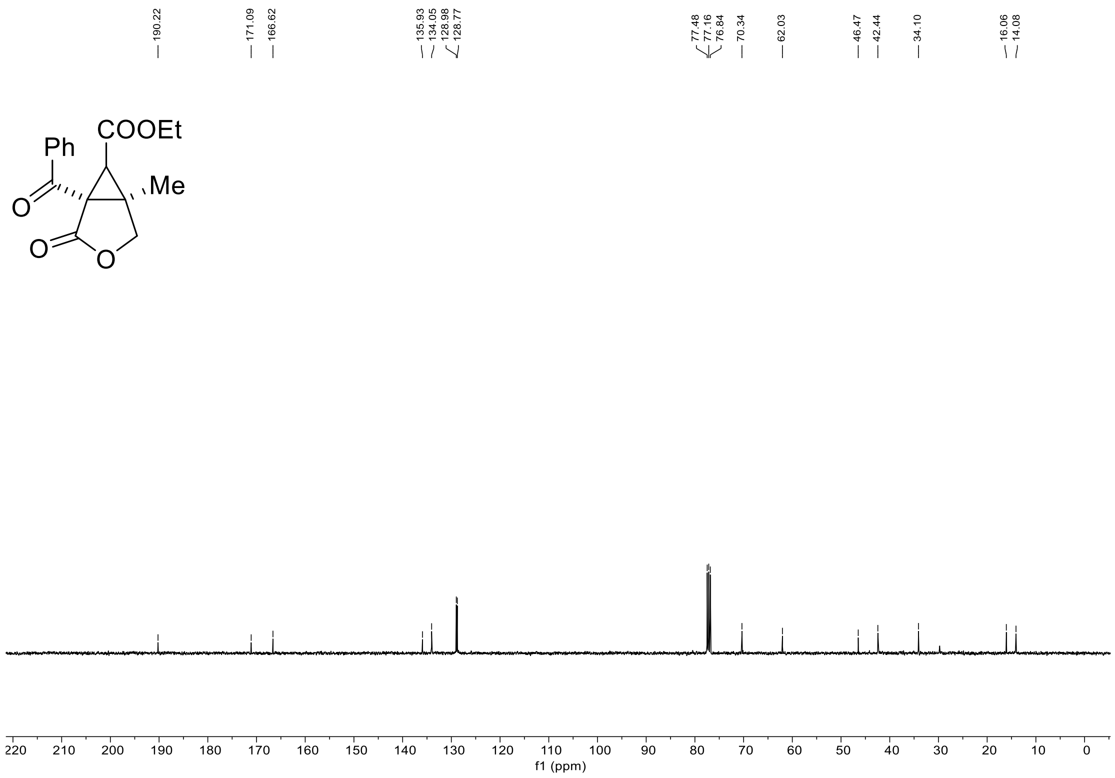

^{13}C NMR spectrum of **28**

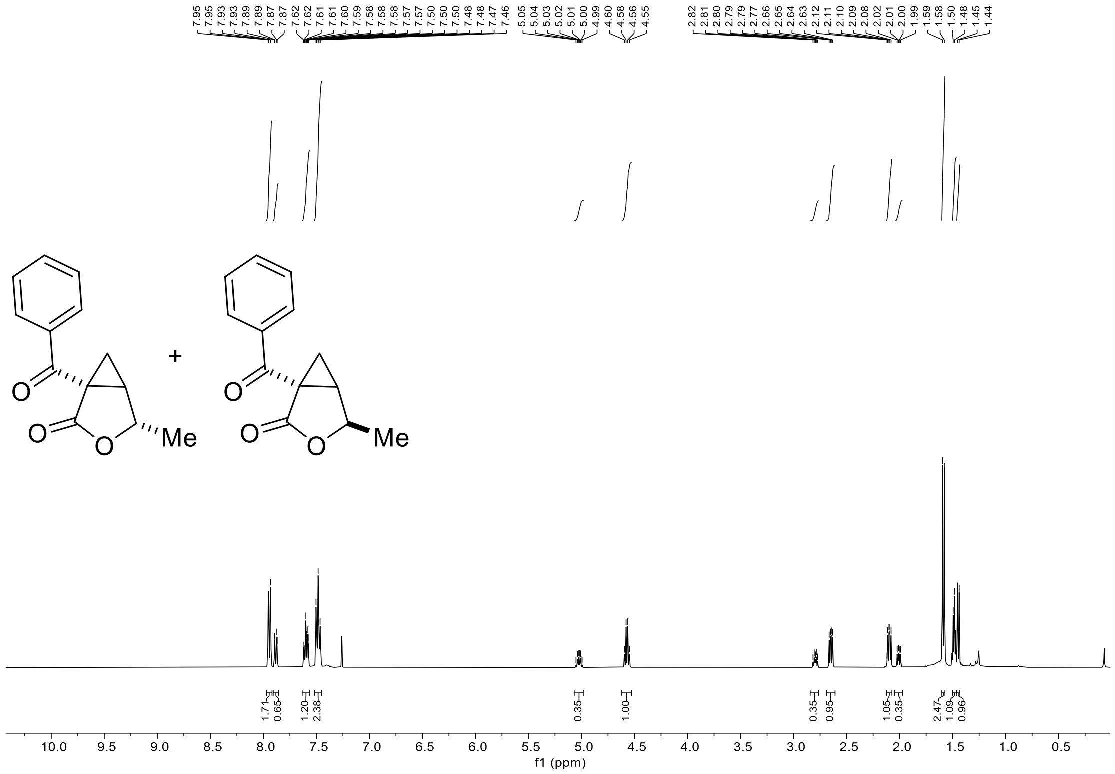

^{1}H NMR spectrum of **29**

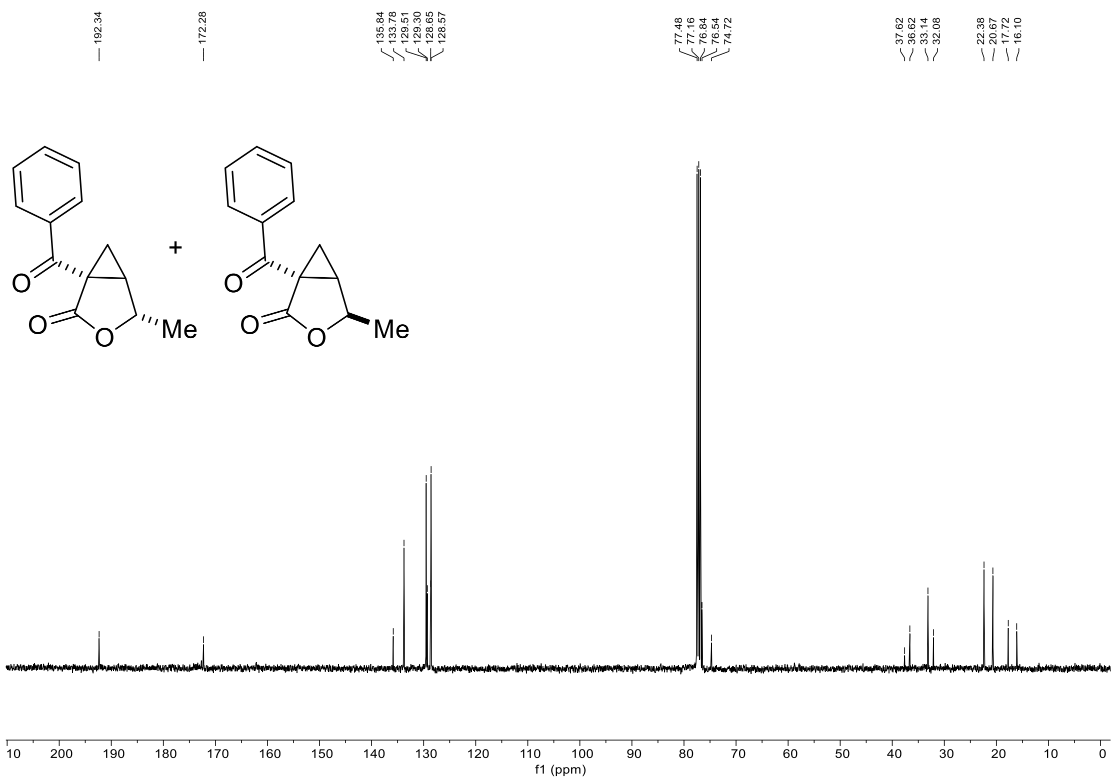

^{13}C NMR spectrum of **29**

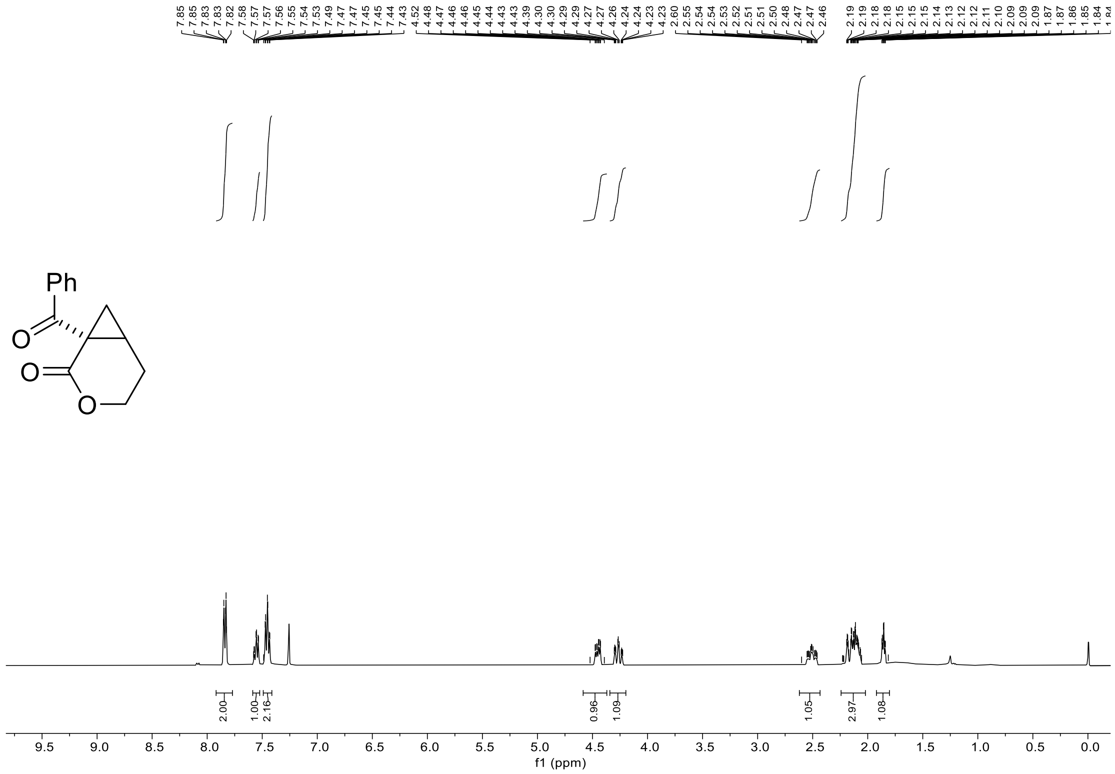

^{1}H NMR spectrum of **30**

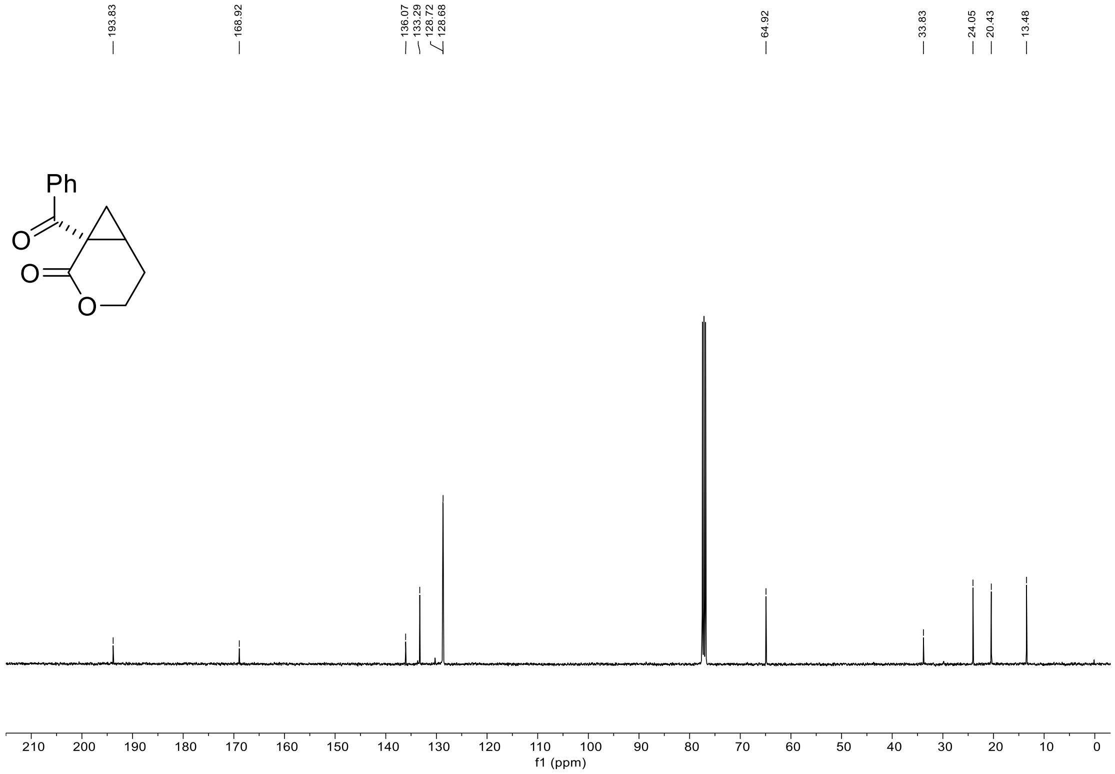

^{13}C NMR spectrum of **30**

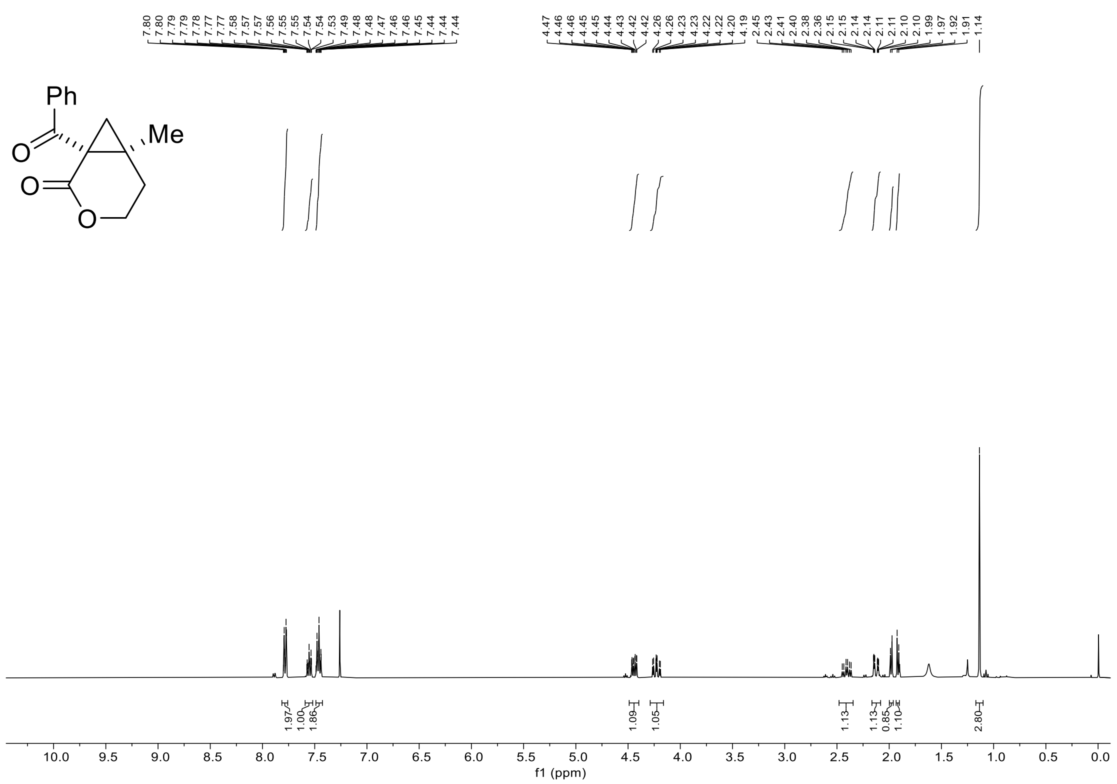

^{1}H NMR spectrum of **31**

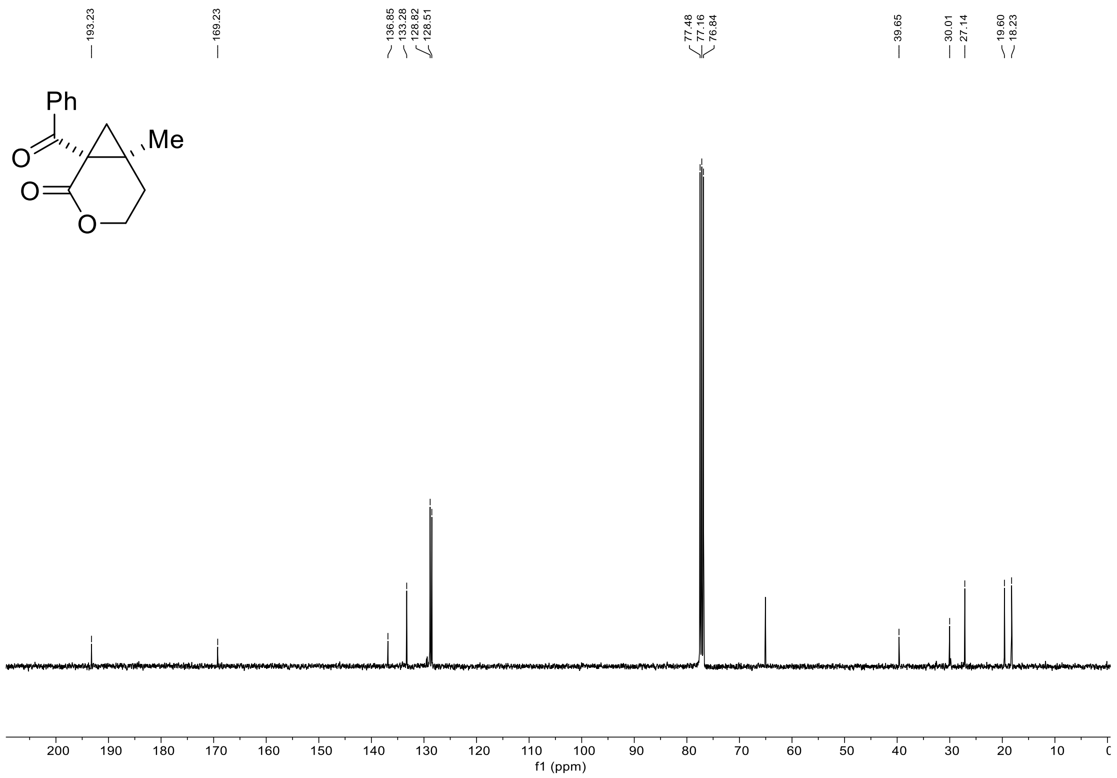

^{13}C NMR spectrum of **31**

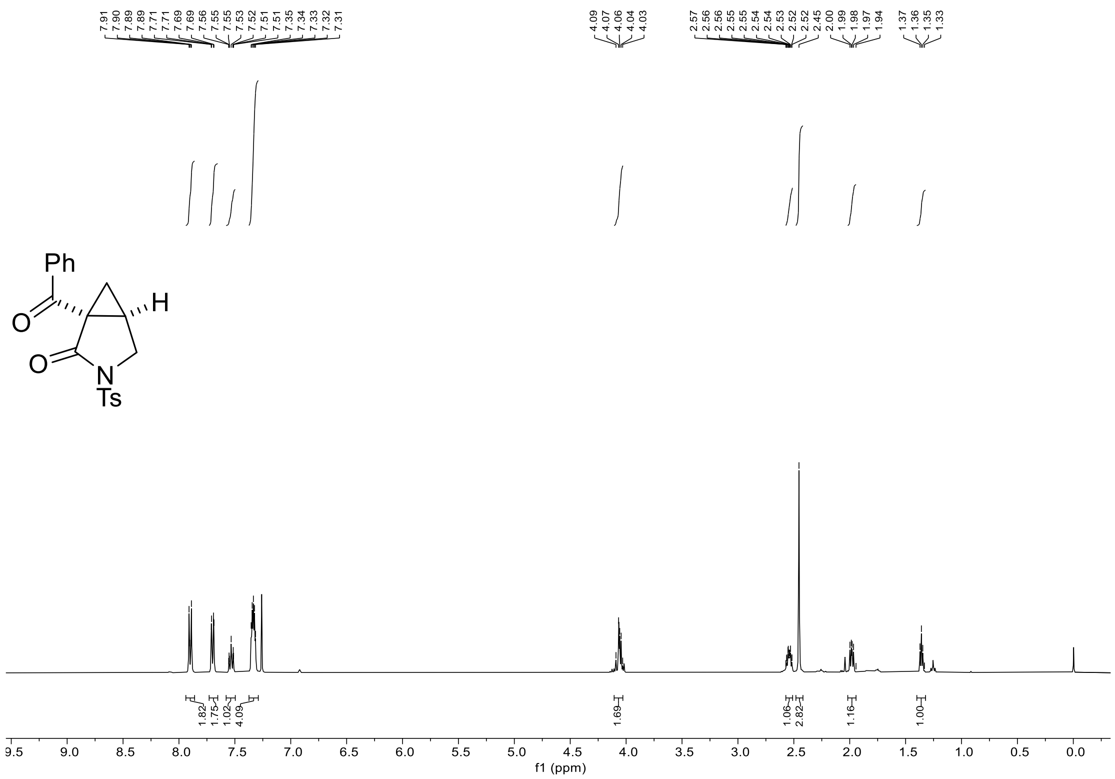

^{1}H NMR spectrum of **32**

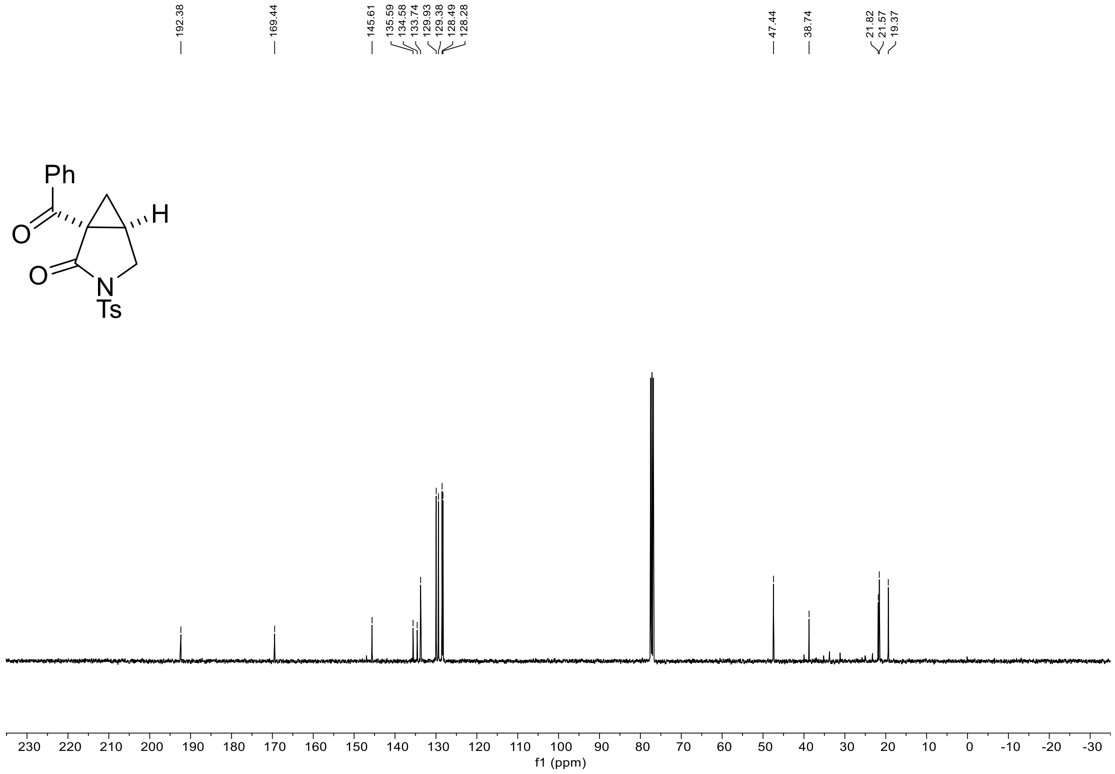

^{13}C NMR spectrum of **32**

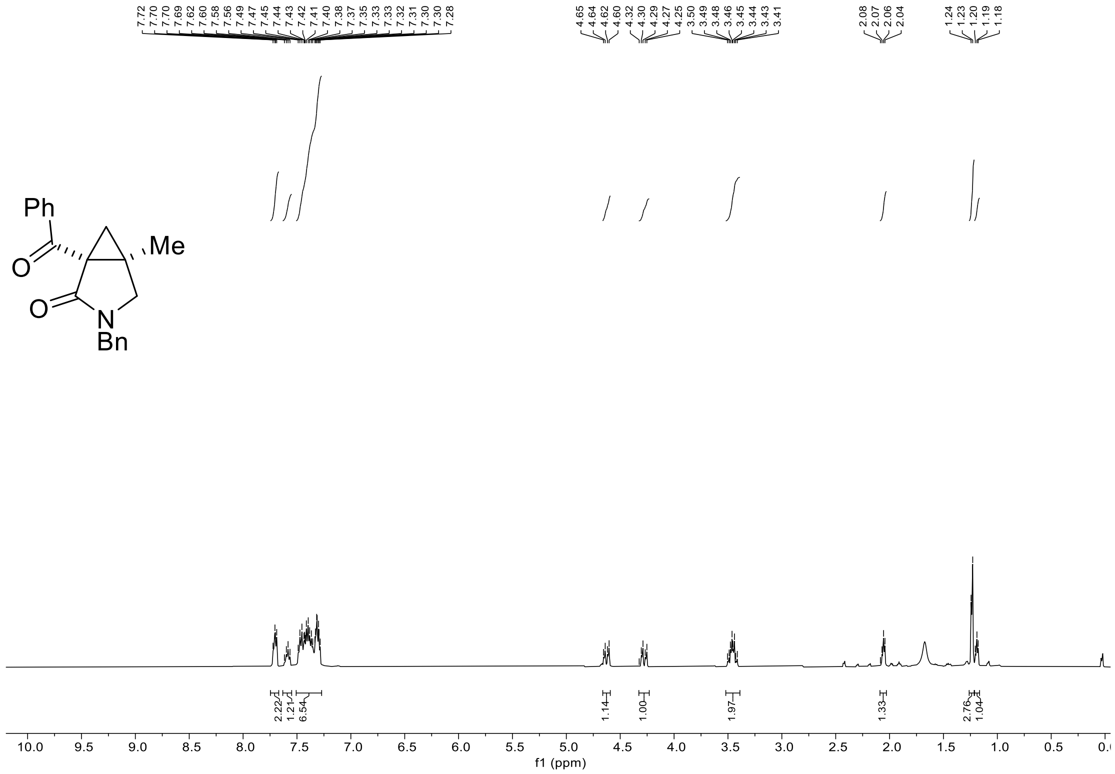

^{1}H NMR spectrum of **33**

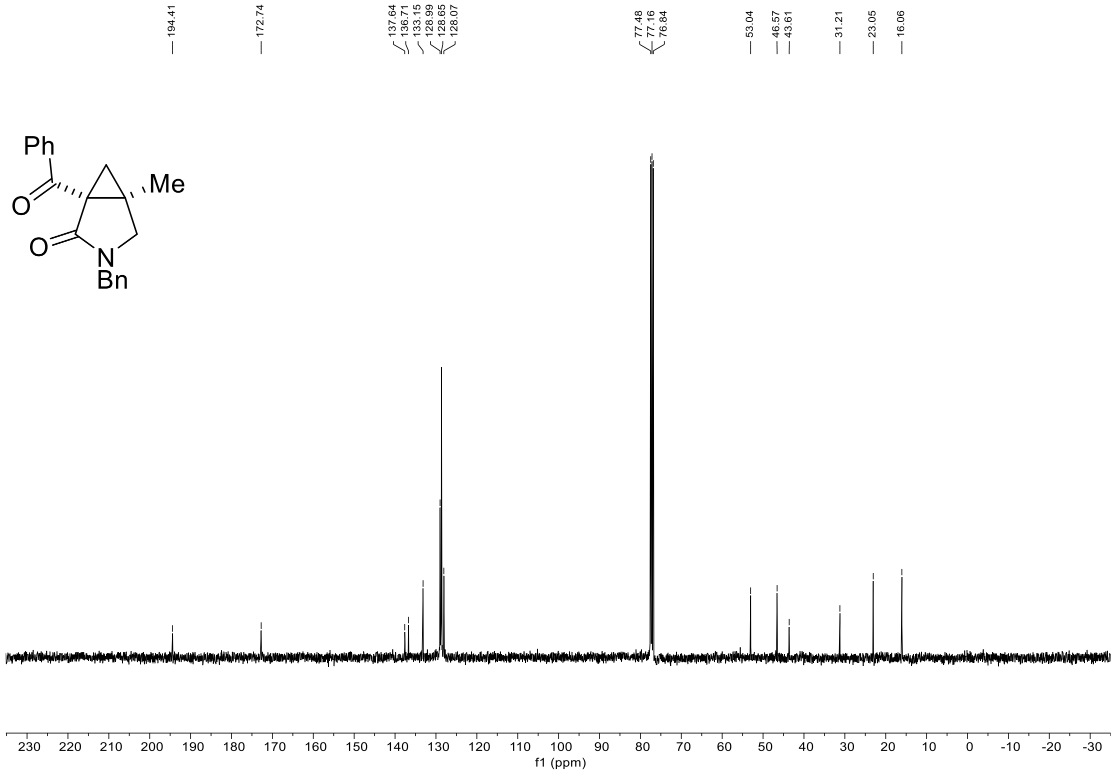

^{13}C NMR spectrum of **33**

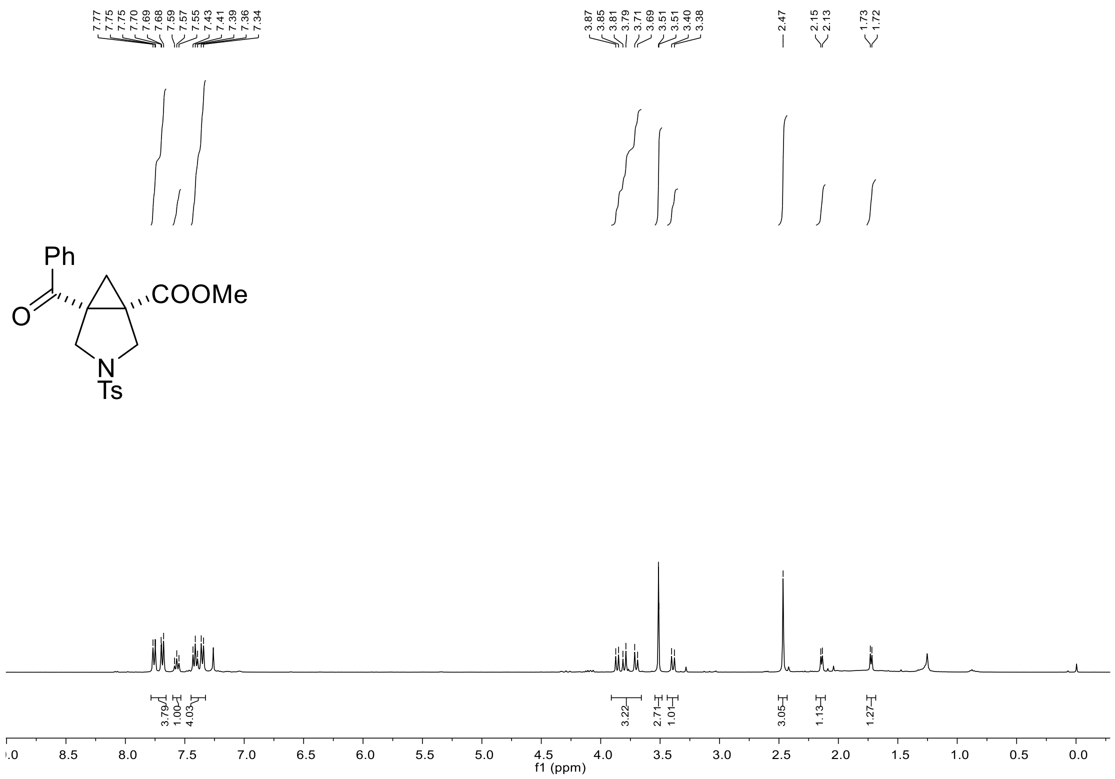

^{1}H NMR spectrum of **34**

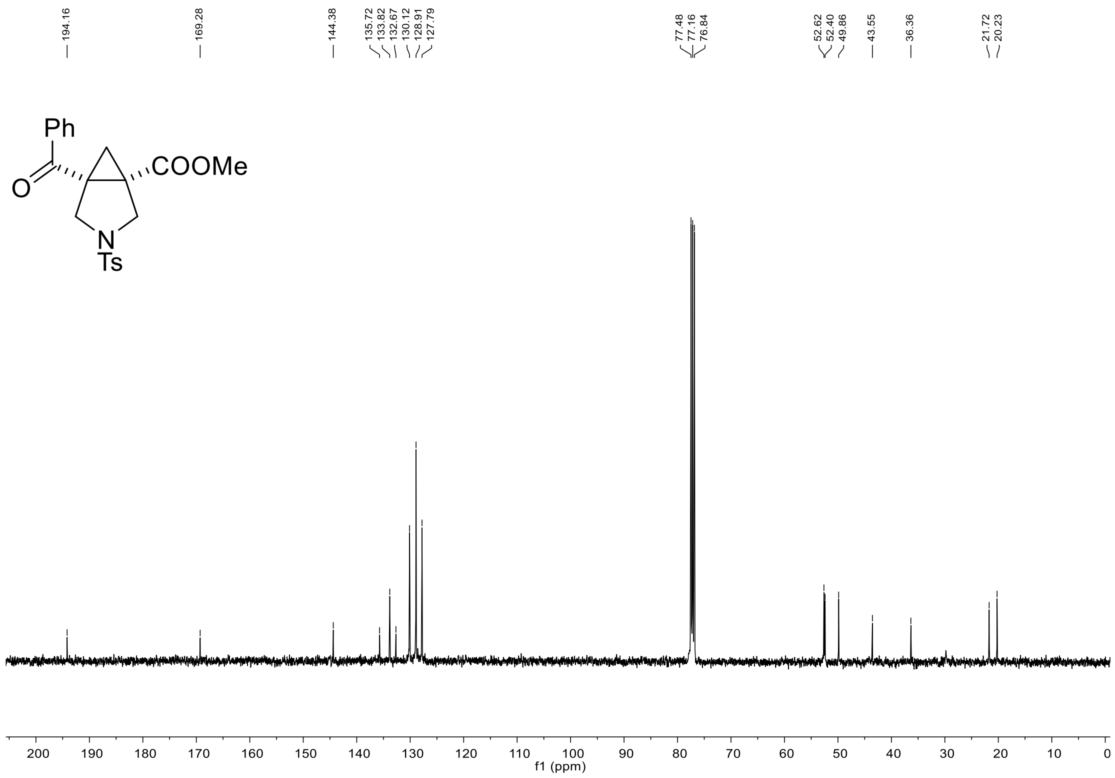

^{13}C NMR spectrum of **34**

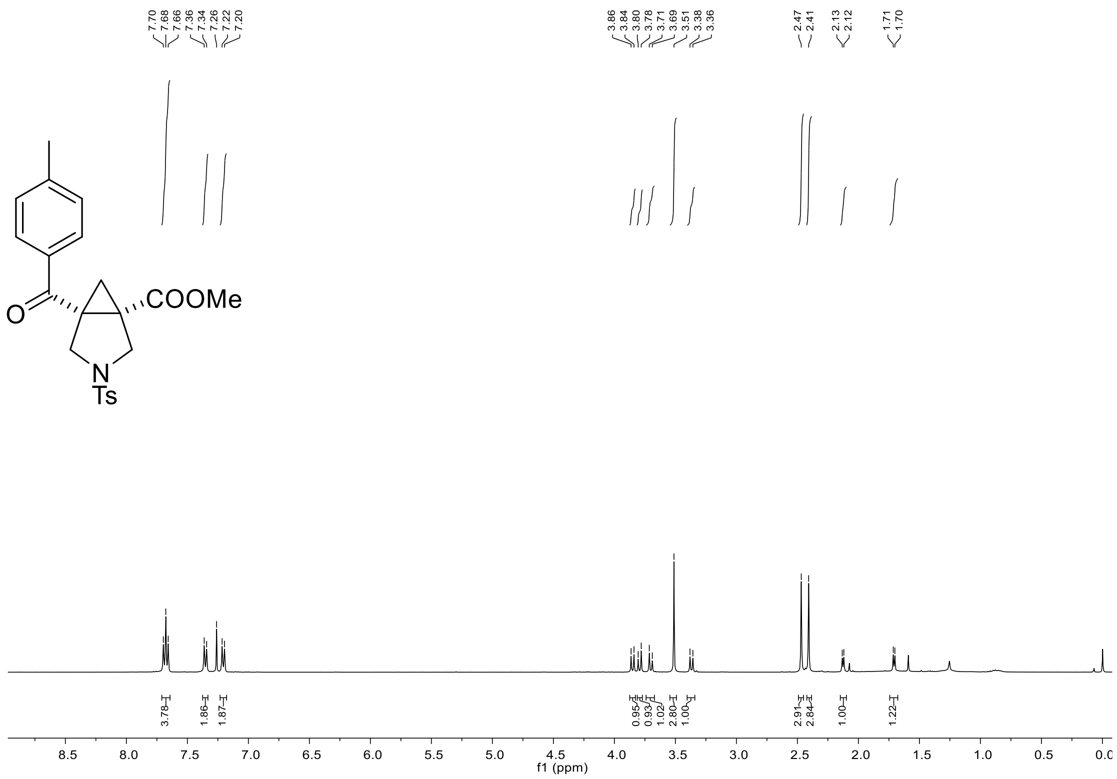

^{1}H NMR spectrum of **35**

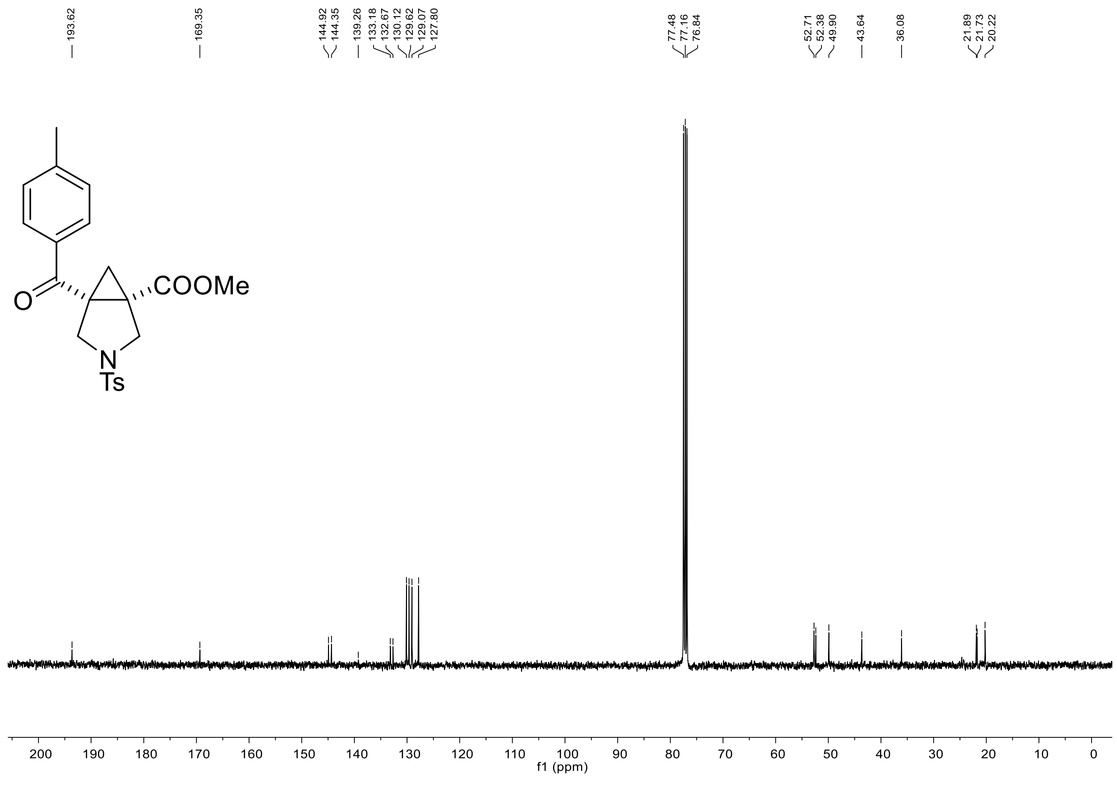

^{13}C NMR spectrum of **35**

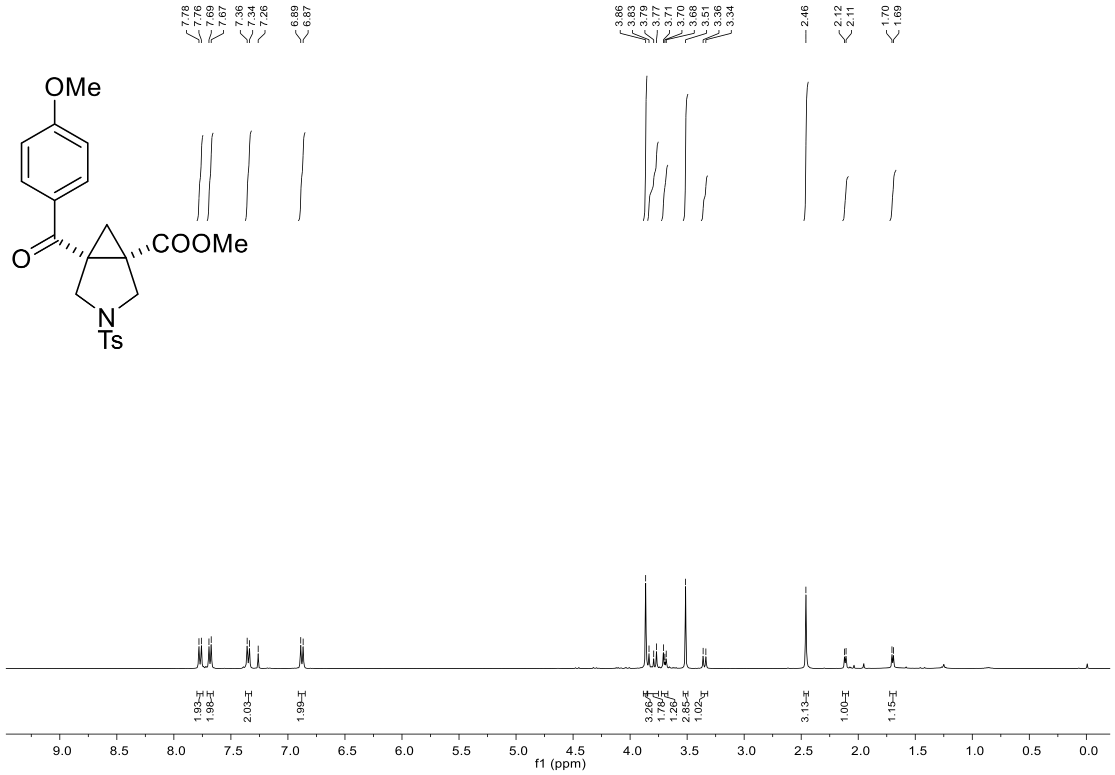

^{1}H NMR spectrum of **36**

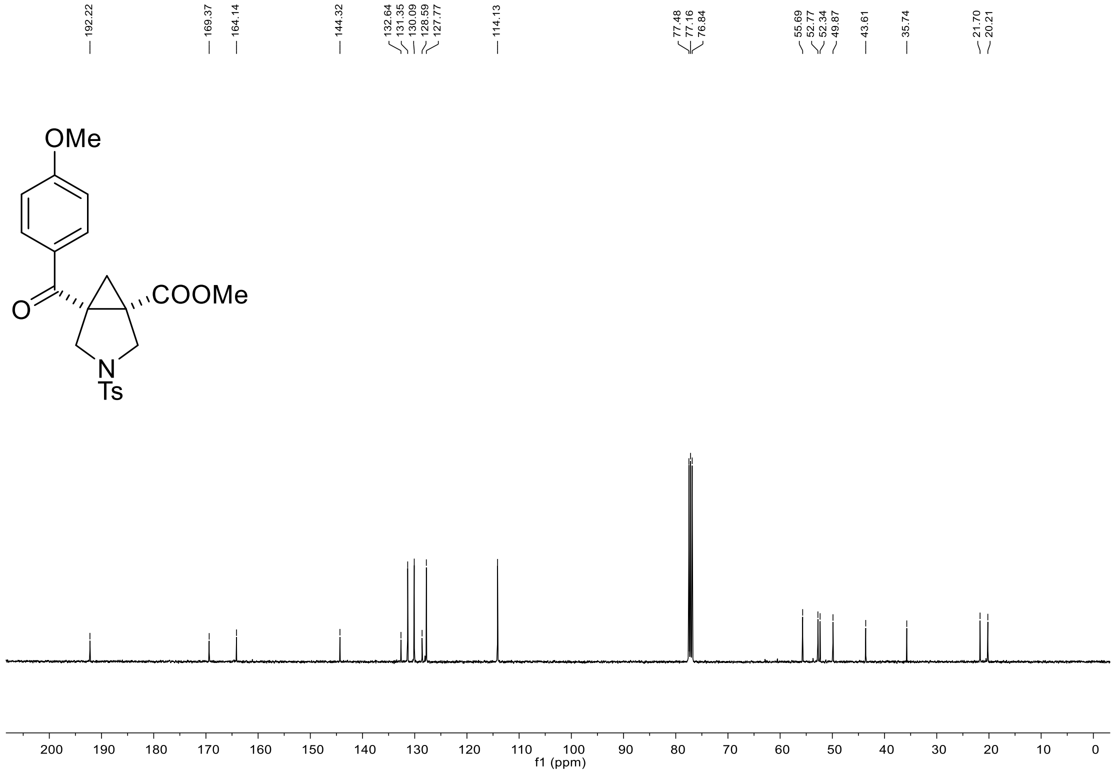

^{13}C NMR spectrum of **36**

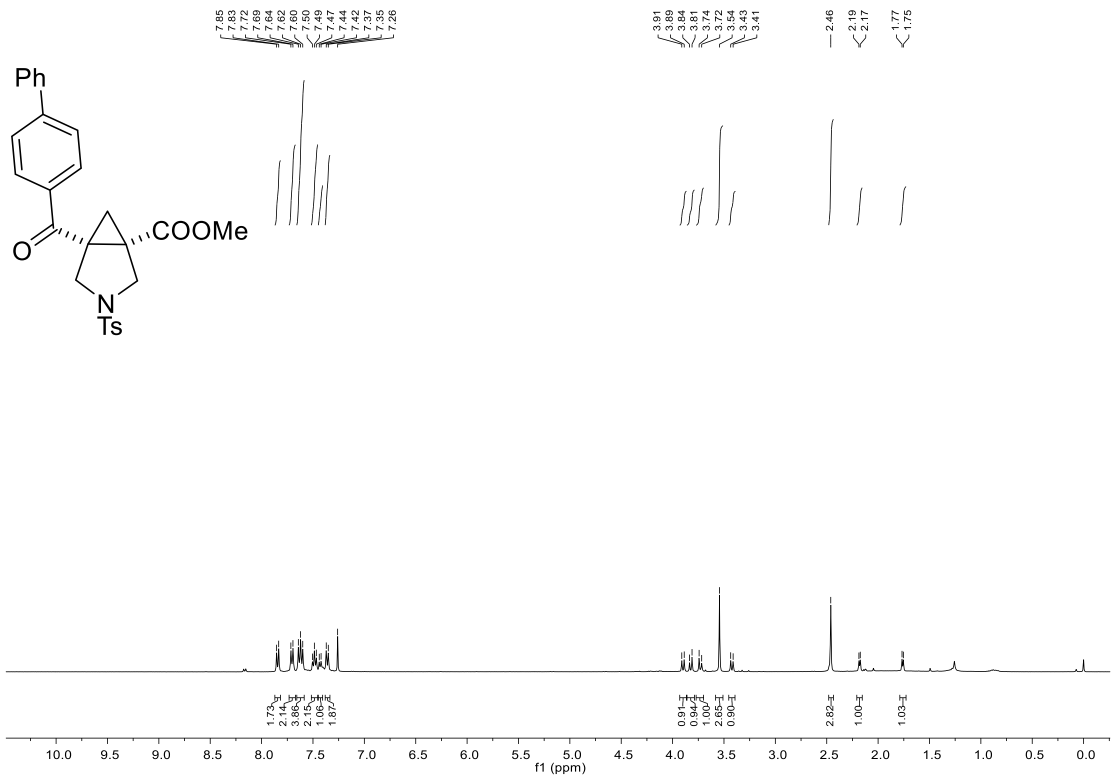

^{1}H NMR spectrum of **37**

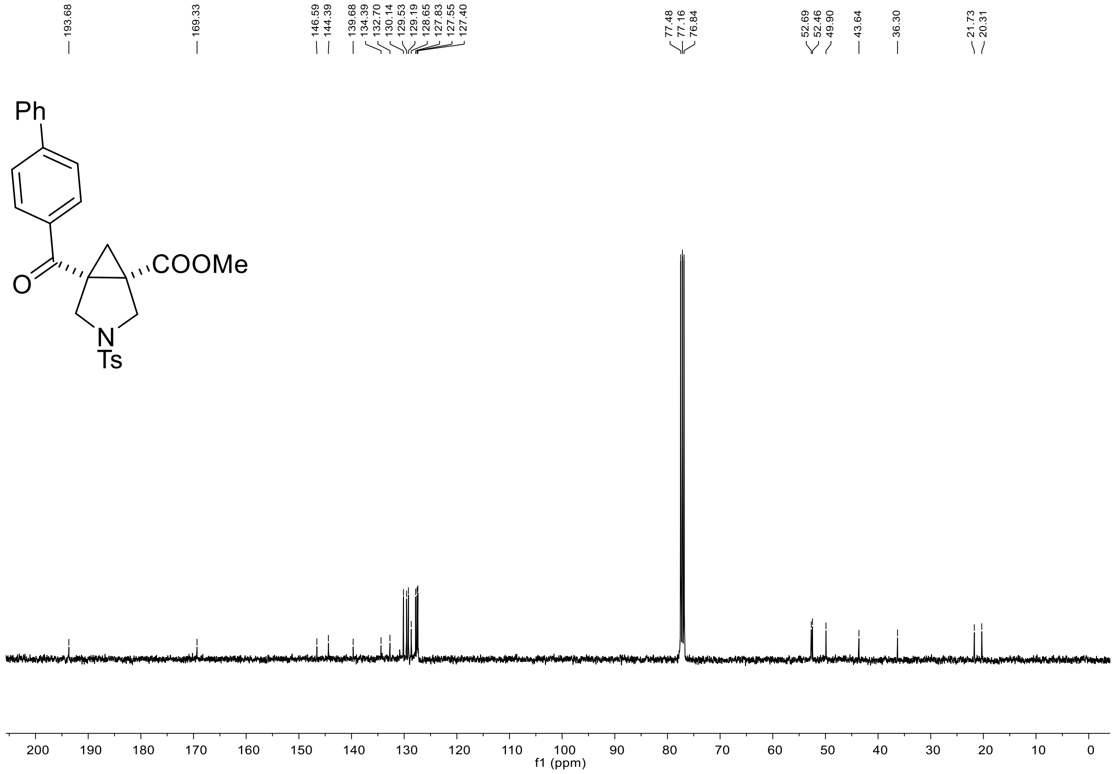

^{13}C NMR spectrum of **37**

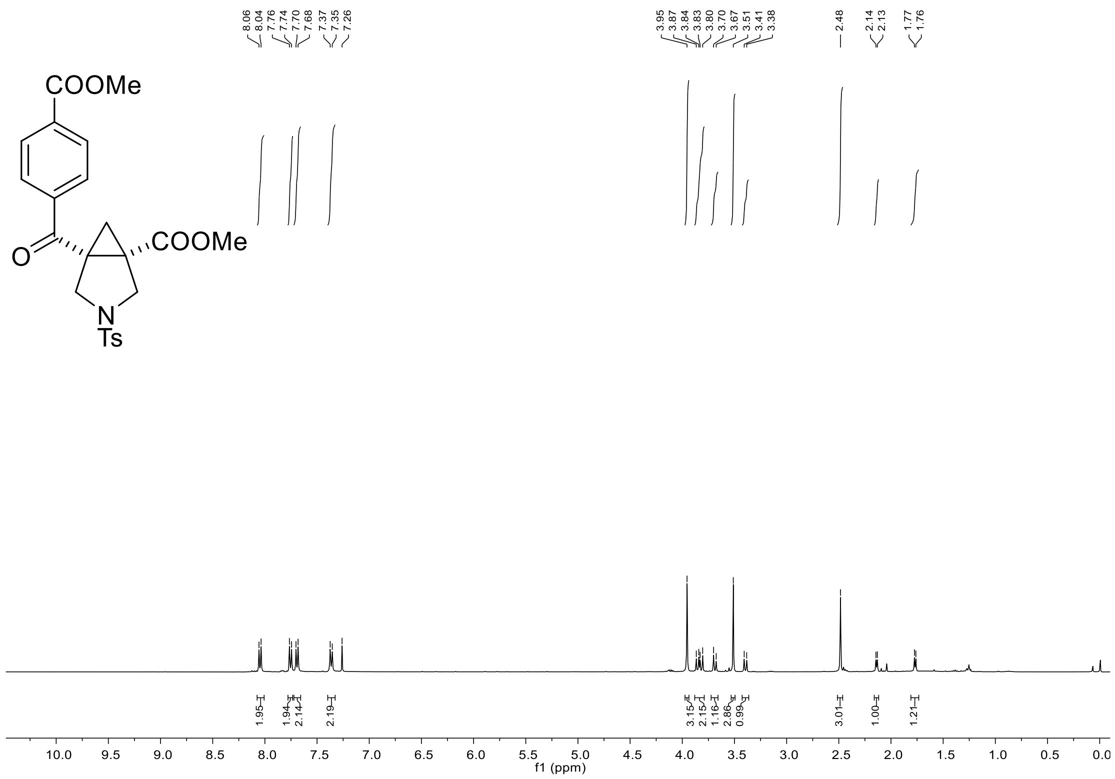

^{1}H NMR spectrum of **38**

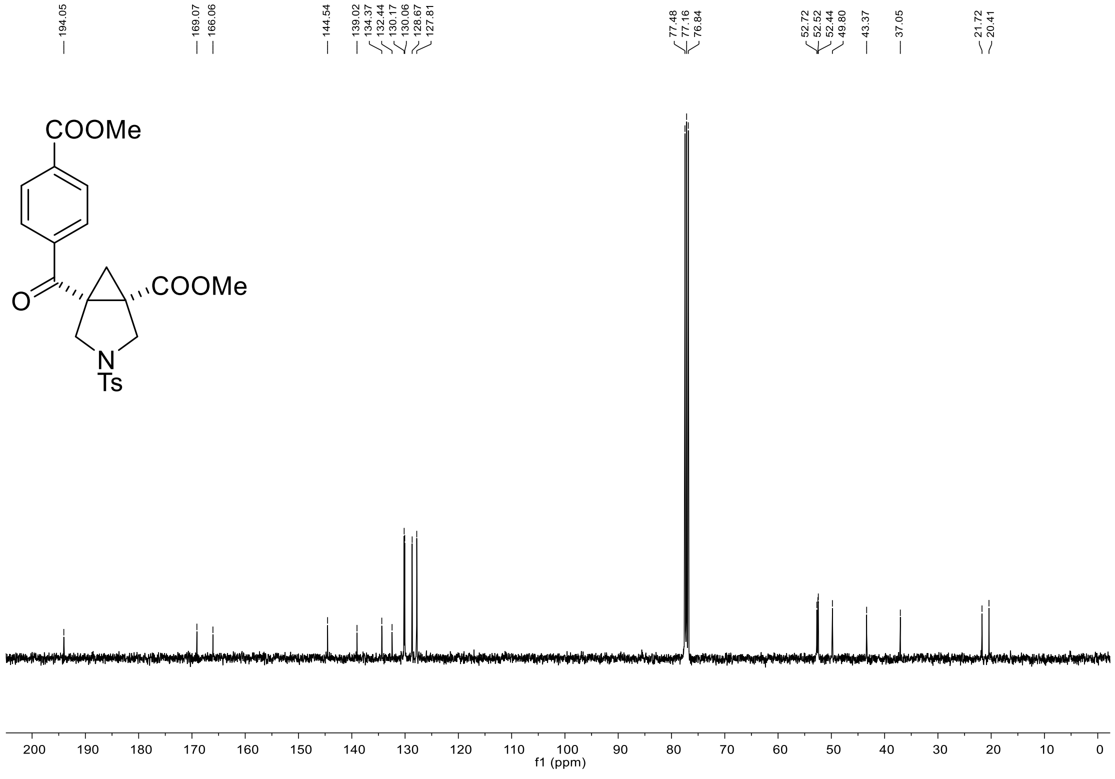

^{13}C NMR spectrum of **38**

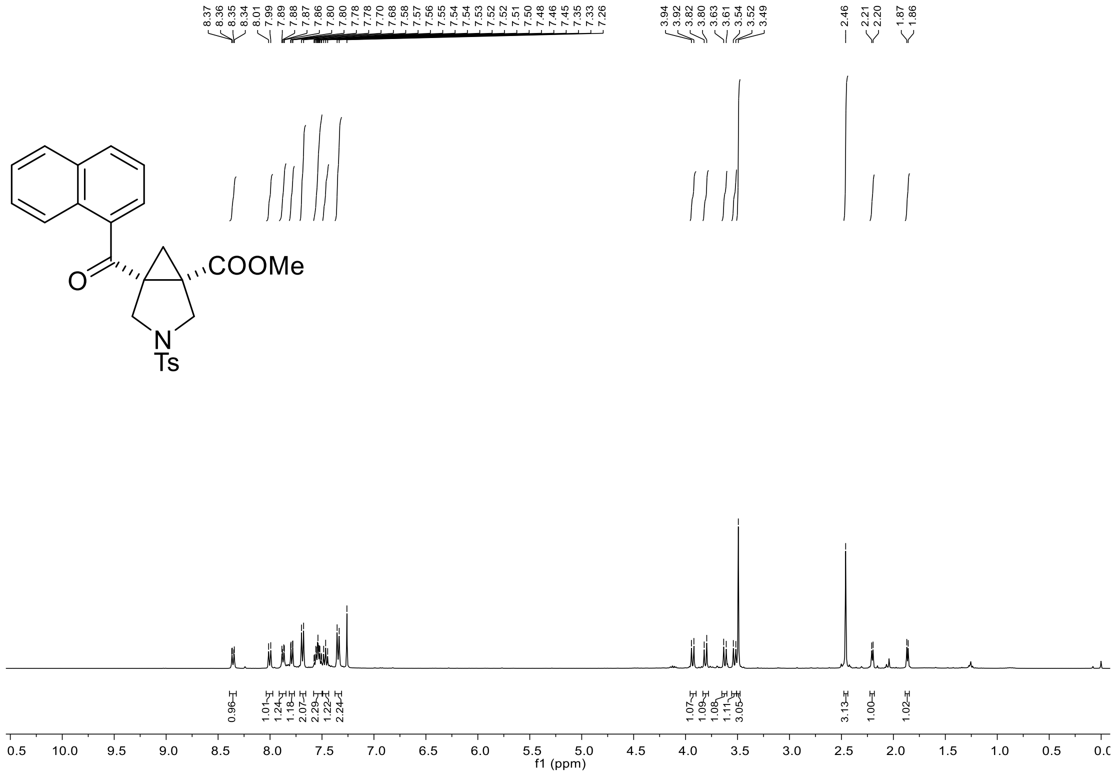

^{1}H NMR spectrum of **39**

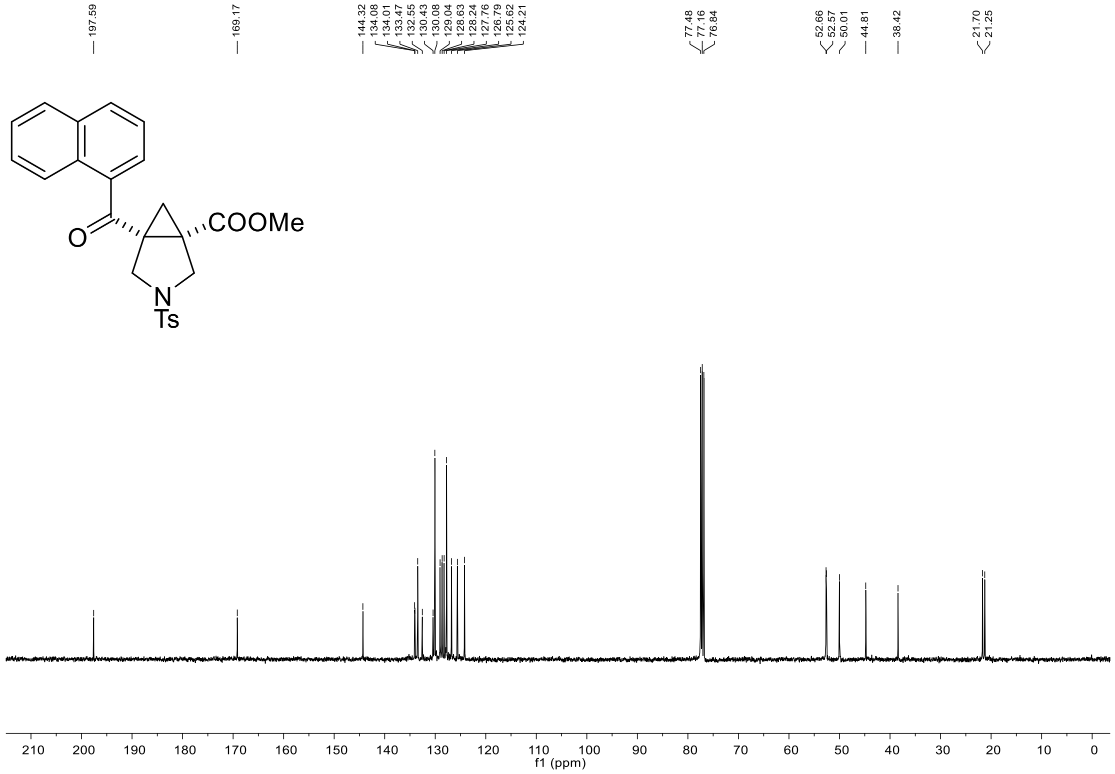

^{13}C NMR spectrum of **39**

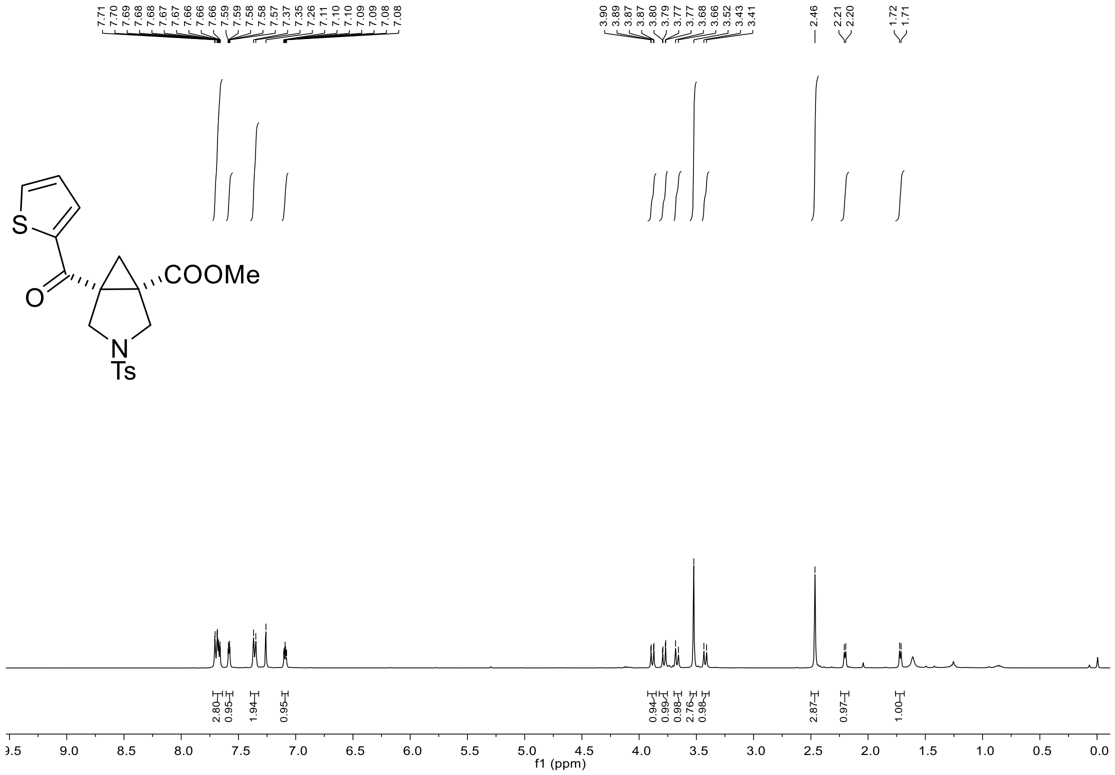

^{1}H NMR spectrum of **40**

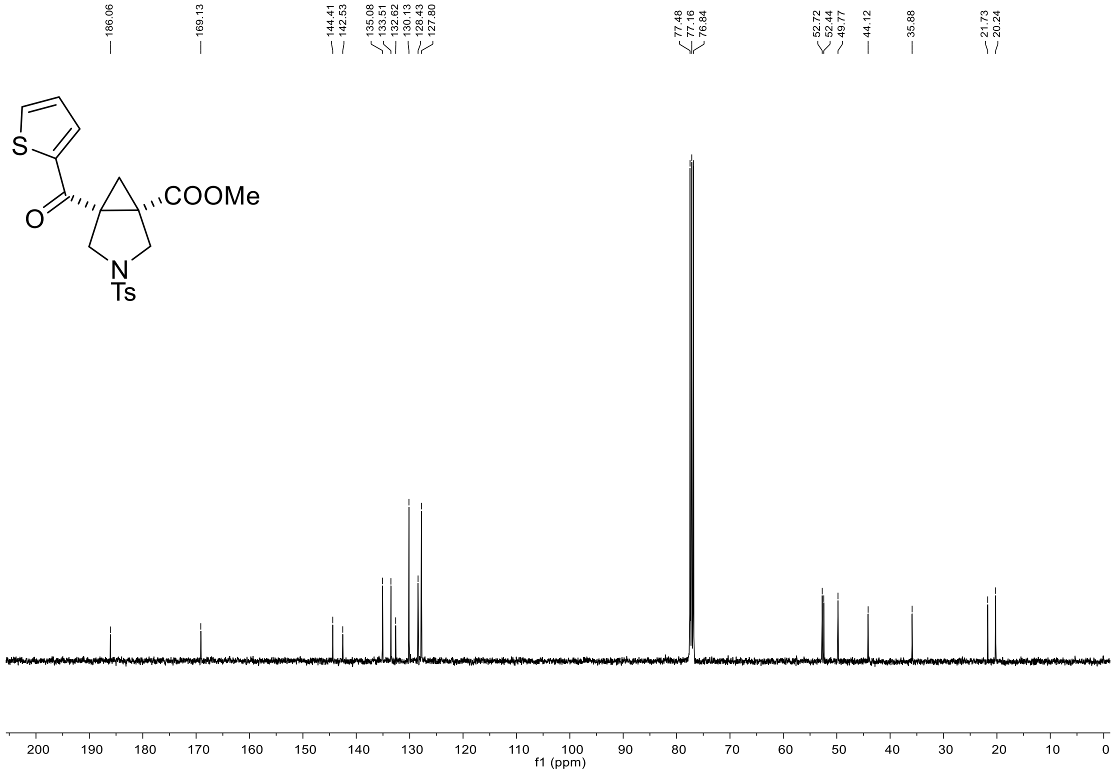

^{13}C NMR spectrum of **40**
